# Supplementary material for: Co-administration of AYUSH 64 as an adjunct to standard of care in mild and moderate COVID-19: A randomized, controlled, multicentric clinical trial
Source: PLoS One. 2023 Mar 16;18(3):e0282688. doi: 10.1371/journal.pone.0282688 (PMC10019690; doi:10.1371/journal.pone.0282688)
Supplement: S2 File — (DOCX) [file pone.0282688.s002.docx]

COVID 19/AYUSH-ICMR 2020/Adjunct Protocol/Version 3.3-F dated 11 May 2020

**FINAL PROTOCOL**

**A Randomized, Open Label, Parallel Efficacy, Active Control, Multi-Centre Exploratory Drug Trial to Evaluate Efficacy and Safety of an Ayurvedic Formulation as Adjunct Treatment to Standard of Care for the management of Mild to Moderate COVID-19 Patients**

**(AYUSH-CSIR-TRT-01)**

**Ministry of AYUSH and Council for Scientific and Industrial Research Collaborative Clinical Research Program**

With technical support from Indian Council of Medical Research

Prepared Under Supervision of

Arvind Chopra MD, DNB, FRCP (London), International Fellow (American College of Rheumatology), Director and Chief Rheumatologist,

Center for Rheumatic Diseases (CRD), Pune

**Authors**

Arvind Chopra, Girish Tillu, Manjit Saluja, Anuradha Venugopalan, Ashwinikumar Raut, Supriya Bhalerao, Tanuja Nesari, Ram Vishwakarma, KS Dhiman, Geetha Krishnan, G G Gangadharan, Sundeep Salvi, Bhushan Patwardhan

**Submitted to**

**Ministry of AYUSH, Government of India**

**Contact Person and Address:**

Dr Arvind Chopra, MD, DNB, FRCP (London)

Center for Rheumatic Diseases (CRD), Hermes Elegance, Convent Street,

Camp, Pune 411 001.

Tele: 020-26345624, 26332973 (M) 98220 39297 FAX: 020-26350084

EMAIL : [crdp5624@gmail.com](mailto:crdp5624@gmail.com)

Web site : [www.rheumatologyindia.org](http://www.rheumatologyindia.org)

**A Randomized, Open Label, Parallel Efficacy, Active Control, Multi-Centre Exploratory Drug Trial to Evaluate Efficacy and Safety of an Ayurvedic Formulation as Adjunct Treatment to Standard of Care for the management of Mild to Moderate COVID-19 Patients**

Contents

[DISCLAIMER 6](#_Toc40270945)

[PRINCIPAL EXECUTIVE STATEMENT ON SPONSORSHIP AND FUNDING 7](#_Toc40270946)

[PRINCIPAL INVESTIGATORS AND STUDY SITES 8](#_Toc40270947)

[STUDY SYNOPSIS 9](#_Toc40270948)

[1. INTRODUCTION 19](#_Toc40270949)

[2. COVID-19 CLINICAL PROFILE 20](#_Toc40270950)

[3. RATIONALE FOR PROPOSED STUDY 37](#_Toc40270951)

[4. OBJECTIVE OF THE STUDY 43](#_Toc40270952)

[5. STUDY DESIGN 43](#_Toc40270953)

[6. CONSENT, SELECTION, SCREENING, and ENROLLMENT 46](#_Toc40270954)

[7. CLINICAL EVALUATION INCLUDING AYURVEDIC ASSESSMENT 47](#_Toc40270955)

[8. STUDY DURATION, FOLLOW- UP SCHEDULE AND PROCEDURES 49](#_Toc40270956)

[9.WITHDRAWAL AND PREMATURE TERMINATION 49](#_Toc40270957)

[10. PATIENT POPULATION 50](#_Toc40270958)

[11. EFFECTIVENSS MEASURES 53](#_Toc40270959)

[12. AYURVEDIC MEASURES 54](#_Toc40270960)

[13. LABORATORY & OTHER INVESTIGATIONS 55](#_Toc40270961)

[14. SAFETY ISSUES AND ENDPOINTS, ADVERSE EVENTS 56](#_Toc40270962)

[15. STUDY PROCEDURES: TIMELINES AND EVENTS SCHEDULE 57](#_Toc40270963)

[16. INVESTIGATIONAL DRUGS AND DOSING 60](#_Toc40270964)

[17. STATISTICAL DESIGN, SAMPLE SIZE ANDANALYSIS, DATA BASE 62](#_Toc40270965)

[18. MANAGEMENT ISSUES 63](#_Toc40270966)

[19. ADMINISTERATIVE ISSUES 65](#_Toc40270967)

[20. ETHICAL CONSIDERATIONS 69](#_Toc40270968)

[21. FINANCING AND INSURANCE 70](#_Toc40270969)

[22. ACKNOWLEDGEMENT 71](#_Toc40270970)

[23. PROTOCOL ACCEPTANCE AND INVESTIGATORS SIGNATURE 73](#_Toc40270971)

[24. REFERENCES 74](#_Toc40270972)

[APPENDIX 81](#_Toc40270973)

[Appendix A: Schedule of Events 81](#_Toc40270974)

[Appendix B: Summary of SOLIDARITY Study 84](#_Toc40270975)

[Appendix C: Sanshamani Vati Plus: Ingredients and Related Research 86](#_Toc40270976)

[Appendix D: AYUSH 64: Ingredients and Related Research 91](#_Toc40270977)

[Appendix E: Yashtimadhu (*Glycyrrhiza glabra*) – A Research Update 98](#_Toc40270978)

[Appendix F: Patient Information Sheet 103](#_Toc40270979)

[Appendix G: Informed Consent Form 108](#_Toc40270980)

[Appendix H: Patient Case Record Form 108](#_Toc40270981)

[Appendix I: Ayurveda Case Record Form 120](#_Toc40270982)

[Appendix J: Daily Progress Report 126](#_Toc40270983)

[Appendix K: Study Completion and Outcome Report 128](#_Toc40270984)

[Appendix N: Self-Reported Questionnaire 131](#_Toc40270985)

[Appendix M: WHO Quality of Life Instrument 132](#_Toc40270986)

[Appendix N: Health Related - Behavior Habit Fitness 137](#_Toc40270987)

[Appendix O: Laboratory Diagnosis and Cytokine Assay 139](#_Toc40270988)

[Appendix P: Classification of Adverse Events 143](#_Toc40270989)

[Appendix Q: Adverse Event Form 145](#_Toc40270990)

[Appendix S: Patient Information Card 148](#_Toc40270991)

[Appendix T: Declartion of Helsinki 149](#_Toc40270992)

[Appendix U: List of Protocol Reviewers 154](#_Toc40270993)

**The Protocol Authors Affiliation and Designation**

1. Dr Arvind Chopra, Director, Center for Rheumatic Diseases, Pune11001
2. Dr Girish Tillu, Assistant Professor, AYUSH Center of Excellence, Interdisciplinary School of Health Sciences, Savitribai Phule Pune University, Pune 11007
3. Ms Manjit Saluja, Coordinator (Training, Research, Drug Trials), Center for Rheumatic Diseases, Pune 11001
4. Dr Anuradha Venugopalan, Deputy Director, Center for Rheumatic Diseases, Pune 411001
5. Dr Ashwinikumar Raut, Director, Clinical Research & Integrative Medicine, Medical Research Centre-Kasturba Health Society, Mumbai 400025
6. Dr Supriya Bhalerao, Scientist, Interactive Research School of Health Affairs, Bharati Vidyapeeth Deemed University, Pune 411 043
7. Dr Sanjiv Sarmukkadam, Consulting Biostatistician, Center for Rheumatic Diseases, Pune 411001
8. Prof Tanuja Nesari, Director, All India Institute of Ayurveda, New Delhi, 110076
9. Dr Ram Vishwakarma, Director, Indian Institute of Integrative Medicine (Council of Scientific & Industrial Research) Jammu 180001
10. Dr KS Dhiman, Director General, Central Council for Research in Ayurvedic Sciences, New Delhi - 110058
11. Dr Geetha Krishnan, Technical Officer, World Health Organization, Geneva
12. Dr G G Gangadharan, Director, M S Ramaiah Indic Centre for Ayurveda and Integrative Medicine, Bengaluru
13. Dr Sundeep Salvi, Director, Pulmocare Research and Education Foundation, Pune
14. Prof Bhushan Patwardhan, Distinguished Honorary Professor, Interdisciplinary School of Health Sciences, Savitribai Phule Pune University, Pune 411007

**Explanatory Note:**

This is a common protocol for three drug trials. Each drug trial is an independent controlled two arm study to evaluate a selected Ayurvedic formulation (AYUSH 64 or Yashtimadhu or Samshamani Vati Plus) as described in this protocol.

The protocol is registered with Clinical Trials Registy India (CTRI).(<https://ctri.nic.in/Clinicaltrials/login.php> ).

# DISCLAIMER

The protocol is based on standard medical and scientific knowledge and experience, and personal opinion of the authors. Scientific deliberations and discussions and comprehensive reference to suitable published medical literature were used to arrive at consensus and prepare this protocol. This is a research protocol and not a document to guide any kind of health or medical practice and all study personnel and patients in the study/drug trials are strictly advised not to use any kind of medication based on this protocol without the explicit knowledge and permission of a qualified physician. The authors claim no responsibility for any act of omission or commission that may arise from this protocol and adversely impact the study or its participants in any manner.

The draft protocol versions went through several cycles of peer review by representatives of Indian Council of Medical Research and independent reviewers. The protocol version 3.3-F is finalized after considering the comments and suggestions from the reviewers. Names and affiliations of reviewers are provided in Appendix U.

# PRINCIPAL EXECUTIVE STATEMENT ON SPONSORSHIP AND FUNDING

This study is part of inter-ministry collaborative initiative of Government of India (GOI) involving Ministry of Health and Family Welfare (MoHFW), Ministry of AYUSH (MoA), Ministry of Science and Technology (MS&T) through Council for Scientific and Industrial Research (CSIR) with technical support and guidance from Indian Council of Medical Research (ICMR). This collaborative study is sponsored and funded by Ministry MoA and CSIR.

A common mechanism (under progress) worked out under the collaborative effort of AYUSH and CSIR (GOI) will ensure that all the required obligations and permissions and all statute requirements of the current study are satisfactorily complied with by the various stakeholders. The latter mechanism will also look after all matters pertaining to funding of the study and speedy dispersal of study funds and payments to the study personnel, supervision and monitoring of study, co-ordination between different stakeholders for smooth and speedy conduct and completion of study, speedy and suitable procurement of all study materials, obtaining all ethics and other mandatory clearances from appropriate regulatory bodies for the study and any other administrative matter that may impact the study adversely. All patients and investigators and study personnel will be protected against any unintentional and inadvertent liability including any kind of financial or legal prosecution arising from any aspect of this protocol or subsequent conduct of the study in any form by the AYUSH and ICMR GOI. It is prudent to add that any allegation of ‘personal negligence’ arising out of this protocol or its subsequent conduct in any form will be thoroughly scrutinized and investigated by the appropriate authority and may call for further legal action and possible prosecution.

# **PRINCIPAL INVESTIGATORS AND STUDY SITES**

Principal Investigator: Dr D. Himanshu Reddy, Professor

Contact: Mobile No. +91 9839266822Email ID: [dr.himanshureddy@gmail.com](mailto:dr.himanshureddy@gmail.com)

Study Site: Department of Medicine, King George's Medical University, Lucknow, 226003

Principal Investigator: Dr Jaya Chakravarty, Professor

Contact: Mobile No.: +91 94530 03725; Email ID: [tapadar@gmail.com](mailto:tapadar@gmail.com)

Study Site: Department of General Medicine, IMS, Banaras Hindu University, Varanasi, 221005

Principal Investigator: Dr Pooja Sharma, MD (Medicine), Head

Contact: Mobile +91 9811535739; Email: [Pooja.Sharma@medanta.org](mailto:Pooja.Sharma@medanta.org)

Study site: Medanta Institute of Education and Research, Medanta, Delhi

Principal Investigator: Vaidya Manohar Gundeti MD (Ayurveda), Research Officer In-charge

Contact: Mobile No. +91 9004960133, Email: [drmanoharccras@gmail.com](mailto:drmanoharccras@gmail.com)

Study Site: RRAP Central Ayurveda Research Institute for Cancer (CCRAS), Podar Medical Campus, Dr A B Road, Worli, Mumbai, 400018

Principal Investigator: Dr Geeta Karambelkar, MD (Paediatrics) Professor and HOD.

Contact: Mobile No. +91 9890938837; Email ID: [hod.paediatrics@smcw.siu.edu.in](mailto:hod.paediatrics@smcw.siu.edu.in)

Study Site: Symbiosis University Hospital & Research Centre, Symbiosis International Deemed University, Lavale, Taluka: Mulshi, Dist: Pune, 412115

**Note:**

- Each study site shall have at least two Principal Investigators, one each from Ayurevda and modern medicine.
- Additional study sites and PIs may be added based on clearances from respective IECs

# STUDY SYNOPSIS

**a. Background:**

Coronavirus Disease-19 (COVID-19) pandemic has unleashed an unprecedented damage to life and livelihood. Over four Million people have contracted the disease globally and almost 2.7 lakh have died by the end of April 2020. There is no specific therapy and the vaccine is likely to be ready by early 2021 if not earlier. All the global strategies are focussed on stringent measures to contain the virus and mitigate the suffering of the people. India is under lockdown since early March 2020 and the situation though grim is fairly stable. There is an upward trend in number of cases but the health care system is suitably meeting the challenge. Clinical research is a vital part of such epidemics. There is a dire need to find newer more effective drugs or at least improve the current standard of care.

India has a rich tradition of Ayurveda since ancient times and several ‘Rasayana’ drugs are well known to enhance the immunity status. Though COVID 19 is an acute infectious disease with a predominant affliction for lungs and airways, the clinical experience so far has shown a rapidly progressive inflammation triggered by several exuberant immunological events. Therefore, there may be a potent role of immunity enhancing and or immunomodulators drugs in the medical management of COVID 19. It is against this perspective that the Ministry of AYUSH and Council of Scientific & Industrial Research (CSIR) have initiated an ambitious and comprehensive research program to discover Ayurveda formulations with proven value in the chemoprophylaxis and treatment of COVID 19.

In this protocol, the focus is on medical treatment.

Three Ayurveda herbal formulations are selected – AYUSH-64 (a proprietary multi plant formulation of CCRAS), Yashtimadhu (*Glycyrrhiza glabra extract* extensively researched by IIIM, Jammu) and ‘Samshamani Vati Plus’ a classical formulation (*Tinospora cordifolia* plus *Piper longum*).

This protocol is common to study all the three formulations; however, any one of them at a time will be individually evaluated for efficacy in a separate randomized two-arm controlled study.

**b. Study title:**

A Randomized, Open Label, Parallel Efficacy, Active Control, Multi-Centre Exploratory Drug Trial to Evaluate Efficacy and Safety of an Ayurvedic Formulation as Adjunct Treatment to Standard of Care for the Management of Mild to Moderate COVID-19 Patients.

**c. Objective:**

**Primary:** To compare the efficacy and safety of a combination regimen of standard of care (SOC) plus a selected standardized Ayurvedic drug (as adjuvant) in the management of mild to moderate cases of COVID-19 with that of standalone SOC control (active control).

**Secondary:** The secondary objectives are (i) To determine the effect of combined standard of care plus Ayurveda drug on the surrogate markers of disease severity and progression, and recovery (ii) To identify predictors of drug response (iii) To describe the clinical phenotype (with reference to characteristics, timelines of occurrence of complications and course of illness)

**d. The investigational products:**

Three formulations will be used in the study; those will be separately used as adjunct treatment. These formulations are –Samshamani Vati Plus, AYUSH 64 and Yashtimadhu.

**e. Study duration:**

The total duration of the study is 12 weeks – this includes a period of hospital based treatment followed by a post recovery period. The Ayurvedic drug will be prescribed as per protocol for a total duration of 12 weeks.

**f. Study design:**

This is a prospective, randomized, open label, blinded end point (PROBE), parallel efficacy, multicentric, two arm study to compare the efficacy of a combination of Standard of Care (SOC) plus a selected individual Ayurveda herbal drug (Samshamani Vati Plus or AYUSH 64 or Yashtimadhu) to SOC (active control). The Ayurveda drug is being considered as an adjunctive or an add-on therapy and each of the three drugs will be evaluated in separate two arm trial with a common study design. The drug trials are ‘exploratory’ in design. The Study Flow diagram summarizes the study design and schedule of events (Fig 1) (Appendix A).

**g. Sample size:** The total sample size is 140 patients per drug trial- 70 patients in each arm.

**h. Study Population:** All adult patients with laboratory-confirmed SARS-CoV2 infection with mild to moderate category will be selected from the outpatient or inpatient facility in COVID-19 medical centers/hospitals. Voluntary patients will be explained about the study and if willing will sign the informed consent and be screened for eligibility.

**Fig 1: Study Flow Diagram with Schedule of Important Study Events**

Screening of Voluntary and Consenting In-patients suffering from Mild to Moderately Severe COVID-19 for Eligibility as per Inclusion-Exclusion Criteria

3 Ayurveda formulation drug trial studies under a common protocol

(Separate study for each AYUSH 64 / Yashtimadhu / Samshamai Vati Plus)

Randomize Eligible Hospitalized Patients

(Open Label, Parallel Efficacy, Active Control Standard of Care (SOC), Two Arm Study)

Selected Ayurveda Formulation (Adjuvant) Versus SOC (Active Control)

Time Points

Clinical Assessment

Investigations

Baseline

Case Record Form (CRF), Medical History Clinical Examination, Ayurveda Assessment, Health Status (WHOQs), QoL (HR-BHF)

Diagnostic (PCR), Serology (IgM, IgG), Routine Hematology Profile, Special Assays, X-Ray Chest & Imaging, ECG

Week 4

Brief Clin Exam, Ayu Exam, Health Status (WHO- Qs) & QoL (HR-BHF Qs), Adverse Events (AEs)

Diagnostic (PCR), Serology (IgM, IgG), Routine haematology Body Profile, Special Assays

Week 8

Brief Clin Exam, Ayu Exam, Health Status (WHO- Qs) & QoL (HR-BHF Qs), Adverse Events (AEs)

Diagnostic (PCR), Serology (IgM, IgG), Routine haematology Body Profile, Special Assays

Week 12

Completion Outcome Assessment, CRF Clin Exam, Ayu Exam, WHO- Qs & QoL (HR-BHF Qs), AEs

Diagnostic (PCR), Serology (IgM, IgG), Routine haematology Body Profile, Special Assays

***Further Assessment Time Points**: (i) During Hospitalization: Daily monitored vital parameters, disease symptoms, disease progression and recovery, and investigations carried out as per protocol till DISCHARGE; Laboratory and Imaging investigations as per protocol but the study investigator and treating physician can do any additional test as per their judgment (ii)Post DISCHARGE: Daily Telephonic Contact using a special mobile phone application for any relapse or any other symptoms or complications or any other Issue (iii) Discharge: Complete all formalities as required for study completion (iv) Skip week 4 or week 8 end point evaluation within 2 weeks of DISCHARGE

The total study period is 12 weeks; Primary Efficacy is ‘Clinical Recovery’ as described in the protocol; Study participant developing severe COVID 19 or severe complication and requiring special critical support/ ICU/ ventilator support will be withdrawn from the study but followed up to record outcome;

Both Ayurvedic and Allopathic Physicians will jointly examine and monitor study participants in addition to the hospital treating physician

**i. Eligibility:**

1. **Inclusion Criteria:**
2. Typical clinical presentation of acute onset febrile illness with cough and a RT_PCR based laboratory confirmation test for COVID-19
3. Patients with either sex, 18 to 75 years age
4. Patients with mild to moderate patients
5. All patients must agree not to share medication
6. Patients willing to participate and sign an informed consent
7. Exclusion Criteria:
8. Patients suffering from severe COVID-19 Disease as judged by a physician and fulfilling at least two of the following three criteria* (i) Respiratory distress at room ambience (≥30 breaths per min) (ii) Oxygen saturation at rest ≤93% (peripheral digital arterial oxymetry) and requiring oxygen support for over one hour to normalize (iii) Any of the known COVID-19 complications and emergency procedures which may require shift/admission in intensive care unit such as respiratory failure, adult respiratory distress syndrome, requirement of oxygen support for over 1 hour, requirement of mechanical ventilation, septic shock, or severe non-respiratory organ dysfunction or failure.
   (Adapted and modified from the reference: Yang Liu et al. Lancet Infect Dis 2020, 2020 https://doi.org/10.1016/ S1473-3099(20)30232-2)
9. Chronic, Severe, Unstable, Uncontrolled co-existent medical illness such as Diabetes, Hypertension, Cardiac disorders, liver, kidney disorders and lung disorders or other disease of concern which may put the patient at increased risk during the study
10. History of immunosuppression: solid organ or bone marrow transplant, use of immunosuppressive antimetabolic and biologic agents, intrinsic immunodeficiencies, HIV infection.
11. Active cancer diagnosis, on palliative treatment or requiring current therapy with antimetabolic agents, immunotherapy or radiotherapy.
12. Patients on parenteral nutrition
13. Patients with known sensitivity or contraindication to any of the ingredients of study medication
14. History of bleeding haemorrhoids, haemoptysis, acid peptic diseases, ulcers and pulmonary diseases (tuberculosis, asthma, etc.)
15. Patients who are likely to worsen or planed ICU admission or ventilator support due to any reason
16. Pregnancy and lactation
17. Participation in a drug interventional clinical drug trial of any nature in the three month period preceding onset of COVID-19
18. Participation in any other clinical trial of an experimental agent treatment for COVID-19
19. Patients on any kind of Ayurveda treatment or any other alternative and complementary medicinal systems such as Homeopathy, Unani, Siddha and in particular requiring oral therapy of any kind.
20. Physician decision that involvement in the study is not in the patient´s best interest

**j. Efficacy:**

**A)** **Primary Outcomes:**

1. Mean time (days) for clinical recovery [Day of randomization to the day of clinical recovery (see criteria below)]
2. Proportion of patients showing ‘clinical recovery’

Criteria of ‘Clinical Recovery’:

1. Normal body temperature (≤36.6°C axilla or ≤37.2 °C oral)
2. Absence of cough or mild cough (infrequent, short episodic, non-wheezy, relieved by minimal or no medication, not interfering with routine speech and not related to lying in bed, mild sore throat or nasal congestion)
3. Absence of breathlessness on routine daily self-care chore or respiratory rate less than 30 breaths per minute without supplemental oxygen
4. Absence of any other symptom/sign attributed to COVID-19 illness
5. Normalization of SpO2 by standard peripheral oximetry device (above 95 percent)
6. Recovery should be sustained for at least 48 hours under physician observation
7. Assessed by physician blinded to treatment allocation (blinded end-point assessment)
8. All of the above criteria ought to be fulfilled

Note: Clinical recovery would be deemed from the first day of satisfying the above criteria

**B)** **Secondary Outcomes:**

1) Rate of patients with negative SARS-CoV-2 on nasal or throat swab in a 2 day continuous real time RT-PCR test beginning from ‘first day of clinical recovery’ or ‘Day 10 after onset of symptoms depending on whichever of the two time points is first achieved

2) Timelines (days counted from onset of illness)- normal body temperature, absence or minimal cough (see ‘clinical recovery’ for the definition), absence of dyspnoea, onset of clinical pneumonia, pneumonia diagnosed on chest X-Ray or CT scan, time to supplemental oxygen, admit in intensive care unit, mechanical ventilation (non-invasive), mechanical ventilation (invasive), steroid use, respiratory failure, adult respiratory distress syndrome, cytokine storm syndrome, secondary infection, shock, septicaemia shock, hospital discharge, negative nose or throat swab confirmatory test, and all-cause mortality.

3) Proportion of patients developing an event that reflects clinical or otherwise improvement of worsening (the events are similar to those listed under Timelines, see above)

4) Improvement on pulmonary function tests using simple ‘home expiratory spirometer’ device and peripheral pulse oximetry

5) Improvement in selected laboratory parameters: blood haemoglobin, differential and total leukocyte counts, liver enzymes, renal functions, acute phase reactants, serum IL-6 and other selected cytokines, serum muscle enzymes (CK, CPK), serum ferritin, serum d-Dimer, anti-oxidant markers, serum BNP (cardiac function)

6) Serological Protective Antibody Assay (IgM and IgG)

7) Radiological Improvement on digital chest X - ray and HRCT chest

8) Drug related: side effects and toxicity, and tolerability (Safety criteria: All adverse events occurring during the study will be recorded and monitored as per GCP-ICH guidelines. Safety would also be assessed in case of all withdrawals.)

9) Health status: WHO QOL brief, health related behaviour habit and fitness questionnaire based on visual analogue scale

**10) Ayurvedic Measures: (Appendix I)**

BASELINE:

a) Prakriti

b) Clinical Features

c) Ayurvedic Disease Subsets / Stages

FOLLOW UP / MONITORING:

a) Clinical Features

b) Ayurveda examination

c) Ayurvedic Disease Subsets / Stages

**k. Medication*:**

Interventional Experimental:

i)AYUSH-64: 500 mg tablet, 2 tablets bid (twice daily)

ii)Yashtimadhu: 300 mg tablet, 2 tablets bid (twice daily)

iii)Sanshamani Vati Plus: Each tablet to contain 300 mg Guduchi plus 75 mg Pippali, 2 tablets bid (twice daily) (*Considering the *Dosha* status Ayurveda physician may change the dose.)

Concomitant:

Medication prescribed for other co-existent disease and permitted as per inclusion criteria will continue under medical supervision of the primary care physician and this will be recorded in the CRF.

**l. Laboratory investigations:**

These are common to the each of the three Ayurveda formulation drug trials:

1. Haemogram, Platelet count, Total leukocyte differential count, Hemoglobin and ESR,
2. Liver function test - Serum Bilirubin, ALT, AST, Alkaline phosphatase,
3. Kidney Function Test ( Serum creatinine, Blood Urea Nitrogen)
4. Lipid profile (Total cholesterol, HDL cholesterol, LDL cholesterol, Triglycerides, VLDL)
5. Blood Sugar Level
6. Urine Routine
7. C-Reactive protein titer
8. LDH, Ferritin
9. Pro-Cal
10. CK, B-type natriuretic peptide (BNP), Troponin, D-Dimer
11. Serum Electrolytes (Sodium, Potassium, Chloride, Iron, Zinc, Manganese) Vitamin D, B12
12. Oxidation Biomarkers-Superoxide Dismutase (SOD), Glutathoine (GSH)
13. Cytokine Panel (Interleukin-2, Interleukin l - 4, Interleukin-6, Interleukin-10, TNF-α, Interleukin-1β, Interleukin-13), Monocyte Chemotactic Protein (MCP), Gamma Interferon. RATIONALE FOR CYTOKINE ASSAY: Cytokine assay can be used to study important anti-viral effects (gamma interferon), immune mediated inflammation (IL-6, anti-TNF, IL-17), TH 1 and TH 2 immune response and antibody producing B cell activity (IL4, IL13), activation of immune cells such as macrophage activation (MCP). Intense up regulation and elevation of IL 6 and several other cytokines has been reported by several clinical case series and research in COVID-19 and can guide specific therapy (as in case of use of monoclonal antibody to IL-6 receptor being used to treat Cytokine storm in COVID 19)
14. Serum Immune Response tests (IgG and IgM) for COVID-19
15. Urine Pregnancy Test for women of child bearing potential

Other Investigations:

1. USG Abdomen and Pelvis
2. Color Doppler and 12 Lead ECG
3. Chest X-ray
4. HRCT Chest

**m. Withdrawal Criteria**

The patient can withdraw at any time during the study without assigning any reason. The investigator may also withdraw the patient due to reasons connected with the severity of disease, interventional drug (adverse event) or some protocol deviation. Detailed description is provided in the protocol.

**n. Statistical statement:**

The design of the drug trial is exploratory in nature. Though a randomized selection of patients will be done to either of the two arms, the sample size is that of convenience (expert opinion) and the same is not statistically powered however, sample size (70 patients in each arm) sufficient to draw meaningful conclusions. The statistical significance p<0.05(two sided) is predefined for analysis. Statistical analysis will be performed using standard tests to compare the two interventional arms for primary and secondary efficacy measures. Safety events will be also analyzed. Both intent-to-treat and per protocol completer analysis will be performed. Regression analysis will be done to identify predictors of response.

**o. Regulatory clearances:**

The protocol will be submitted for all regulatory and other relevant approvals including that of the ethics committee prior to beginning the clinical component of the study. The trial protocol will be registered under Clinical Trial Registry of India before enrolling the first patient.

**p. Funding agency:**

COVID 19/AYUSH-CSIR 2020/Adjunct Protocol/Version 3.3-F dated 10 May 2020 is being funded by Ministry of AYUSH and CSIR Government of India.

**q. Guidelines:**

The study will be conducted in accordance with the principles of Good Clinical Practice (GCP) of the WHO and the current version of the Declaration of Helsinki, ICMR (Indian Council of Medical Research) Ethical Guidelines for Biomedical Research on Human patients (2017), and AYUSH/CCRAS Guidelines for clinical research in Ayurveda and GCP (2018).

# 1. INTRODUCTION

The on-going COVID-19 (Corona virus disease) pandemic is unprecedented and has caused a global disaster of immeasurable magnitude in terms of human morbidity and mortality and loss to economy and livelihood. According to the Coronavirus Resource Center of Johns Hopkins University, over 4 million confirmed cases with over 0.20 million deaths were reported from all over the world as of the first week of May 2020 (1). This Corona virus disease, akin to its predecessor SARS epidemic originated in China and rapidly engulfed large stretches of the World (2). Early identification of the genomic sequence and molecular structure of this novel Corona virus (named SARS - CoV-2) has led to sensitive and specific diagnostic test (RT-PCR technology) and an understanding of the core pathophysiology mechanism of the aggressive nature of the disease.

The situation in India, though grim, is certainly reassuring so far with much lesser number of cases and deaths as compared to China, European countries and USA. The Government of India has accorded health matters and saving people’s lives as top most priority and initiated stringent measures of containment all over the country since early March 2020. There is a steady increase in the number of cases to about 62,000 and about 2,100 deaths by 10^th^ May 2020. (1)

Though the World has witnessed several Influenza and Coronavirus epidemics in the recent decade, the World does not seem to be prepared to combat COVID-19 pandemic (3)(4)(5). Despite several action plans, the COVID-19 has been a bolt from the blue. Several countries are desperately clinging on to a strategy to contain the virus and delay its spread (6). Non-pharmaceutical interventions (NPIs) are pivotal to protect and safeguard the community till such time that vaccine becomes available. NPIs are also called community mitigation measures and include several personal protection measures (7). Comparatively, the nationwide program of lockdown in India has been useful and the success is largely due to a massive and largely voluntary community participation to enforce social distancing, hand and self-hygiene and using nose and mouth physical barriers like masks (8). This was also necessitated by the fact that the medical care facilities in India are ill-equipped to handle a huge burden of patients suffering from severe COVID-19 illness and requiring critical care support including ventilators.

The pandemic is still on and the medical community needs to be cautious and ever prepared to help the community pull through the severe phase. Also there is a dire need to identify the gaps in the knowledge about COVID-19. Despite the severe losses to finance and a precarious economy, India too will need a medical research agenda. There is no specific modern medicine to prevent or cure the disease. Despite the on-going intense modern science search for better drugs and an effective vaccine in rest of the developed World, it is worthwhile to search Ayurveda for some medical solutions to improve the standard of care (SOC).

It is against this perspective that the Ministry of AYUSH GOI has laid a foundation of an ambitious and comprehensive clinical research program for studying Ayurveda derived interventions in the prophylaxis and management of COVID-19 and critically test some of the most potential candidates using standard clinical drug trials.

Here we present a protocol to evaluate the efficacy and safety of Ayurveda based botanical formulations as an add-on or adjunct therapy to the standard of care in the medical management of COVID 19 in a randomized study design. The protocol is based on literature review of several recent research publications and clinical experience for treating flue like illness following principles and practices of Ayurveda. Much of the rationale of the timelines for study events is based on critical observations gleaned from these research publications. It is prudent to add that Indian data on COVID 19 is sparse at the moment.

# 2. COVID-19 CLINICAL PROFILE

Important published research studies on COVID-19 were selected to tabulate the data on symptoms (Table 1), timelines of the course of disease (Table 2), complications (Table 3) and co-morbidity (Table 4).

COVID-19 is an acute respiratory tract infection. Though the clinical phenotype is similar to influenza, the spread and severity of illness is much more in case of COVID-19. The overall mortality of COVID-19, albeit due to respiratory complications, seems to be several folds higher than that of influenza. And unlike influenza, there is no vaccine or specific drugs to kill the virus.

Viral RNA levels have been demonstrated from upper respiratory and lung specimens. In over 90% cases, the viral RNA tests on nasopharyngeal swabs was found to be repeatedly negative at about 10 days after the onset of symptoms but the viral shedding may continue well beyond clinical recovery; median duration 20 days (range of 8 to 37 days) (9,10). In mild infections, the virus may not be detected especially if the viral RNA level in respiratory specimen is less than 106 copies/mL. Some recent studies have also shown prolonged virus shedding in stool samples.

The incubation time of COVID-19 is about 5-7 days and the infectivity is often high much before the onset of symptomatic illness (11). The infection remains asymptomatic in a substantial proportion of community and a predominant proportion of symptomatic cases will suffer from mild to moderate cases. However, asymptomatic individuals may show abnormalities in the lungs on computed tomography (CT) scans (12). It is estimated that less than 20 % cases will rapidly progress to severe stage and develop life threatening lung and systemic complications requiring critical care and often life support systems like ventilators. The overall mortality rate is about 2-3%. According to the WHO, the recovery time appears to be around two weeks for mild infections and three to six weeks for severe disease.

**2.1 Clinical features (Table 1, Table 2, Table 3, Table 4):**

There are no specific clinical features of COVID-19 that can distinguish it from other viral respiratory infections (13). Men are more commonly affected for reason not clear. No age has been found exempt, but the infection is more prone, severe and fatal in elderly. Pneumonia like disease is the single most important feature (2). A comprehensive list of symptoms described in the selected published literature is shown in Table 1; published data from India is sparse so far an a small sample size case series is also included. Patients generally present with fever, sore throat, cough, dyspnea, and may show early bilateral infiltrates on chest imaging. Myalgia and fatigue is not uncommon. But several other unusual features are described (14). An early loss of sense of smell and taste has been described and may be specific for COVID-19 (15)(). Severe and fatal cases are reported to have several complications and are associated with co-existent morbidity especially hypertension, cardiac disorders, diabetes and obesity(16). Compared with non-ICU patients, ICU patients had higher plasma levels of several cytokines and in particular interleukin-6 (IL-6); a cytokine storm may precede rapid deterioration and death. Rarely, thromboembolic complications, including pulmonary embolism and acute stroke, have also been reported (17).

**Table 1: COVID-19 SYMPTOMS (LITERATURE REVIEW*):**

| **Study** | **Wang et al.^1^** | **Guan et al.^2^** | **Zhou et al.^3^** | **Goyal et al.^4^** | **Bhandari et al.^5^** |
| --- | --- | --- | --- | --- | --- |
| **Site** | China | China | China | USA | India |
| **Design** | Retrospective  Observational  Single center | Retrospective  Observational  Multicenter | Retrospective  Observational  Multicenter | Retrospective  Observational  Multicenter | Retrospective  Observational  Single center |
| **Nature of site** | Hospital | Hospital | Hospital | Hospital | Hospital |
| **Sample** | 138 | 1099 | 191 | 393 | 21 |
| **Age, median (IQR), y** | 56 (42–68) | 47 (35-58) | 56 (46-67) | 62 (49-74) | 43 (2-85) |
| **Male (%)** | 54 | 58 | 62 | 61 | 67 |
| **Features/Symptoms (%)** | | | | | |
| **Fever** | 99 | 89 | 94 | 77 | 52 |
| **Cough** | 59 | 68 | 79 | 79 | 57 |
| **Myalgia** | 35 | 15 | 15 | 23.8 | 43 |
| **Dyspnea** | 31 | 19 | - | 56.5 | 19 |
| **Sputum production/**  **Expectoration** | 27 | 34 | 23 | - | 24 |
| **Diarrhea** | 10 | 4 | 5 | 24 | 38 |
| **Fatigue** | 70 | 38 | 23 | - | - |
| **Anorexia** | 40 | - | - | - | - |
| **Headache** | - | - | - | - | 19 |
| **Nausea,**  **Vomiting,**  **Abdominal pain.** | ≤10 | ≤10 | ≤10 | 19 | - |

IQR: Interquartile range.

***References:**

1. Dawei Wang, Bo Hu, Chang Hu, et al. Clinical Characteristics of 138 Hospitalized Patients With 2019 Novel Coronavirus–Infected Pneumonia in Wuhan, China. JAMA. 2020;323(11):1061-1069. DOI:10.1001/jama.2020.1585.
2. Wei-jie Guan, Zheng-yi, Yu Hu, Wen-hua Liang, Chun-quanOu, Jian-xing He, et.al. Clinical Characteristics of Coronavirus Disease 2019 in China. N Engl J Med. 2020. DOI: 10.1056/NEJMoa2002032.
3. Fei Zhou, Ting Yu, Ronghui Du, Guohui Fan, Ying Li, Zhibo Liu, et al. Clinical course and risk factors for mortality of adult inpatients with COVID-19 in Wuhan, China: a retrospective cohort study. The Lancet. 2020;395:1054-1062. DOI:https://doi.org/10.1016/S0140-6736(20)30566-3.
4. Parag Goyal, Justin J. Choi, Laura C. Pinheiro, Edward J. Schenck, Ruijun Chen, Assem Jabri. Clinical characterisitics of COVID-19 in New York City. N Engl J Med. 2020 April. DOI: 10.1056/NEJMc2010419
5. Bhandari S, Bhargava A, Sharma S, Keshwani P, Sharma R, Banerjee S. Clinical Profile of COVID-19 Infected Patients Admitted in a Tertiary Care Hospital in North India. 2020. Downloaded from <http://apiindia.org/wp-content/uploads/pdf/corona-virus/covid-19-publication-03.pd>

**Table 2: COVID-19 TIMELINES OF THE COURSE OF DISEASE (LITERATURE REVIEW#):**

| **Study** | **Wang et al.^1^** | **Li et al.^2^** | **Huang et al.^3^** | **Zhou et al.^4^** |
| --- | --- | --- | --- | --- |
| **Site** | China | China | China | China |
| **Study type** | Retrospective  Observational  Single center | Prospective  Observational  Multicenter | Prospective  Observational  Single center | Retrospective  Observational  Multi center |
| **Nature of Site** | Hospital | Hospital | Hospital | Hospital |
| **Sample size** | 138 | 425 | 41 | 191 |
| **Time to symptoms- Median days (IQR)** | | | | |
| **Fever** | -- | -- | -- | 1 (1-1) |
| **Cough** | -- | -- | -- | 1 (1-3) |
| **Dyspnoea** | 5 (1-10) | -- | 8 (5-13) | 7 (4-9) |
| **1^st^ Hospital visit** |  | 4.6 (95%CI 4.1-5.1) | 8 (5-13) | -- |
| **Hospital admission** | 7 (4-8) | 9.1 (95%CI 8.6-9.7) | 7 (4-8) | -- |
| **Time to complications- Median days (IQR)** | | | | |
| **ICU admission** | -- | -- | 10.5 (8-17) | 12 (8-15) |
| **Mechanical Ventilation** | -- | -- | 10.5 (7-14) | 14.5 (12-19) |
| **ARDS** | 8 (6-12) | -- | 9 (8-14) | 12 (8-15) |
| **Sepsis/ Shock** | -- | -- | -- | 9 (7-13) |
| **Acute Cardiac injury** | -- | -- | -- | 15 (10-17) |
| **Acute Kidney injury** | -- | -- | -- | 15 (13-19.5) |
| **Time to outcome- Median days (IQR)** | | | | |
| **Discharge** | -- | -- | -- | 22 (18-25) |
| **Death** | -- | -- | -- | 18.5 (15-22) |
| **Recovery** | 10 (7-14) | -- | -- | -- |
| **Others** | -- | -- | -- | Viral shedding:  20 (17-24)  Severe disease:  19 (17-22)  Critical disease:  24 (22-30) |

ARDS: Acute respiratory distress syndrome. IQR: Interquartile range, CI: Confidence interval.

**#References:**

1. Dawei Wang, MD1; Bo Hu, MD1; Chang Hu, MD1; et al. Clinical Characteristics of 138 Hospitalized Patients With 2019 Novel Coronavirus–Infected Pneumonia in Wuhan, China. JAMA. 2020;323(11):1061-1069. DOI:10.1001/jama.2020.1585.
2. Qun Li, Xuhua Guan, Peng Wu, Xiaoye Wang, Lei Zhou, Yeqing Tong, et al. Early Transmission Dynamics in Wuhan, China, of Novel Coronavirus–Infected Pneumonia. N Engl J Med 2020; 382:1199-1207 DOI:10.1056/NEJMoa2001316.
3. Chaolin Huang, Yeming Wang, Xingwang Li, Lili Ren, Jianping Zhao, Yi Hu, et al. Clinical features of patients infected with 2019 novel coronavirus in Wuhan, China. 2020; 395:467-536. DOI:https://doi.org/10.1016/S0140-6736(20)30183-5.
4. Fei Zhou, Ting Yu, Ronghui Du, Guohui Fan, Ying Li, Zhibo Liu, et al. Clinical course and risk factors for mortality of adult inpatients with COVID-19 in Wuhan, China: a retrospective cohort study. The Lancet. 2020;395:1054-1062. DOI:https://doi.org/10.1016/S0140-6736(20)30566-3

**Table 3: COVID-19 COMPLICATIONS (LITERATURE REVIEW**):**

| **Study** | **Wang et al.^1^** | **Nanshan et al.^2^** | **Huang C et al.^3^** | **Guan et al.^4^** | **Zhou et al.^5^** |
| --- | --- | --- | --- | --- | --- |
| **Site** | China | China | China | China | China |
| **Design** | Retrospective,  Observational,  Single center | Retrospective,  Observational,  Single center | Retrospective,  Observational,  Single Center | Retrospective,  Observational,  Multicenter | Retrospective, Observational, Multicenter |
| **Nature of Site** | Hospital | Hospital | Hospital | Hospital | Hospital |
| **Sample size** | 138 | 99 | 41 | 1099 | 191 |
| **Age, median (IQR), y** | 56 (42-68) | 55.5 | 49 (41-58) | 47 (35-58) | 56 (46-67) |
| **Male (%)** | 54 | 68 | 73 | 58 | 62 |
| **Complications (%)** | | | | | |
| **ARDS** | 20 | 17 | 29 | 3 | 31 |
| **Shock** | 9 | 4 | 7 | 1 | 59 |
| **Acute Kidney Injury** | 4 | 3 | 7 | 0.5 | 15 |
| **Pneumonia** | -- | -- | -- | 91 | -- |
| **Acute respiratory injury/failure** | -- | 8 | - | -- | 54 |
| **Arrhythmia** | 17 | -- | -- | -- | -- |
| **Acute Cardiac Injury** | 7 | -- | 12 | -- | 17 |
| **Heart failure** | -- | -- | -- | -- | 23 |
| **VAP** | -- | 1 | -- | -- | -- |
| **Secondary infection** | -- | -- | 10 | -- | 15 |
| **DIC / coagulopathy** | -- | -- | -- | 0.1 | 19 |
| **Rhabdomyolysis** | -- | -- | -- | 0.2 | -- |
| **Acidosis** | -- | -- | -- | -- | 9 |
| **Hypoproteinaemia** | -- | -- | -- | -- | 12 |

ARDS: Acute respiratory distress syndrome, VAP: Ventilator associated pneumonia, DIC: Disseminated Intravascular Coagulopathy. IQR: Interquartile range.

****References:**

1. Dawei Wang, Bo Hu, Chang Hu, et al. Clinical Characteristics of 138 Hospitalized Patients With 2019 Novel Coronavirus–Infected Pneumonia in Wuhan, China. JAMA. 2020;323(11):1061-1069. DOI:10.1001/jama.2020.1585.
2. Nanshan Chen,MinZhou,Xuan Dong, Jieming Qu, Fengyun Gong, Yang Han, et al.Epidemiological and clinical characteristics of 99 cases of 2019 novel coronavirus pneumonia in Wuhan, China: a descriptive study. Lancet. 2020;395;507-513. DOI:https://doi.org/10.1016/S0140-6736(20)30211.
3. ChaolinHuang,Yeming Wang, Xingwang Li, Lili Ren, Jianping Zhao, Yi Hu, et al. Clinical features of patients infected with 2019 novel coronavirus in Wuhan, China. 2020; 395:467-536. DOI:https://doi.org/10.1016/S0140-6736(20)30183-5.
4. Wei-jie Guan, Zheng-yi, Yu Hu, Wen-hua Liang, Chun-quanOu, Jian-xing He, et.al. Clinical Characteristics of Coronavirus Disease 2019 in China. N Engl J Med. 2020. DOI: 10.1056/NEJMoa2002032.
5. Fei Zhou, Ting Yu, Ronghui Du, Guohui Fan, Ying Li, Zhibo Liu, et al. Clinical course and risk factors for mortality of adult inpatients with COVID-19 in Wuhan, China: a retrospective cohort study. The Lancet. 2020;395:1054-1062. DOI:https://doi.org/10.1016/S0140-6736(20)30566-3.

**Table 4: COVID-19 CO-MORBIDITIES (LITERATURE REVIEW^# #^)**

| **Study** | **Guan et al.^1^** | **Wang et al.^2^** | **Zhou et al.^3^** | **Richardson et al.^4^** | **Goyal et al.^5^** |
| --- | --- | --- | --- | --- | --- |
| **Site** | China | China | China | USA | USA |
| **Design** | Retrospective  Observational  Multicenter | Retrospective  Observational  Single center | Retrospective  Observational  Multicenter | Retrospective  Observational  Multicenter | Retrospective  Observational  Multicenter |
| **Nature of site** | Hospital | Hospital | Hospital | Hospital | Hospital |
| **Sample** | 1099 | 138 | 191 | 5700 | 393 |
| **Age, median (IQR), y** | 47 (35-58) | 56 (42–68) | 56 (46-67) | 63 (52-75) | 62 (49-74) |
| **Male (%)** | 58 | 54 | 62 | 60 | 61 |
| **Co-morbidities (%)** | | | | | |
| **Any** | 24 | 46 | 48 | 94 |  |
| **Hypertension** | 15 | 31 | 30 | 57 | 50 |
| **Coronary Artery Disease** | 2.5 | 14.5 | 8 | 11 | 14 |
| **Cerebrovascular disease** | 1 | 5 | - | - | - |
| **COPD** | 1 | 3 | 3 | 5 | 5 |
| **Asthma** | - | - | - | 9 | 12.5 |
| **Diabetes** | 7 | 10 | 19 | 34 | 25 |
| **Obesity^#^** | - | - | - | 42 | 36 |
| **Malignancy** | 1 | 7 | 1 | 6 | - |
| **Chronic renal disease** | 1 | 3 | 1 | 5 | - |
| **Chronic liver disease^*^** | 2 | 3 | - | 1 | - |
| **Immunodeficiency^^^** | 0.2 | 1 | - | 2 | - |

# Body mass index > 30 kg/m^2^. * Chronic liver diseases include Hepatitis B, hepatitis C infection, cirrhosis. ^ Immunodeficiency includes HIV, history of solid organ transplant. IQR: Interquartile range.

**# # References:**

1. Wei-jie Guan, Zheng-yi, Yu Hu, Wen-hua Liang, Chun-quanOu, Jian-xing He, et.al. Clinical Characteristics of Coronavirus Disease 2019 in China. N Engl J Med. 2020. DOI: 10.1056/NEJMoa2002032.
2. Dawei Wang, Bo Hu, Chang Hu, et al. Clinical Characteristics of 138 Hospitalized Patients With 2019 Novel Coronavirus–Infected Pneumonia in Wuhan, China. JAMA. 2020;323 (11):1061-1069. DOI:10.1001/jama.2020.1585.
3. Fei Zhou, Ting Yu, Ronghui Du, Guohui Fan, Ying Li, Zhibo Liu, et al. Clinical course and risk factors for mortality of adult inpatients with COVID-19 in Wuhan, China: a retrospective cohort study. The Lancet. 2020;395:1054-1062. DOI:https://doi.org/10.1016/S0140-6736(20)30566-3.
4. Safiya Richardson, Jamie S. Hirsch; MangalaNarasimhan, et al. Presenting Characteristics, Comorbidities, and Outcomes Among 5700 Patients Hospitalized With COVID-19 in the New York City Area. 2020. DOI:10.1001/jama.2020.6775.
5. ParagGoyal, Justin J. Choi, Laura C. Pinheiro, Edward J. Schenck, Ruijun Chen, AssemJabri. Clinical characterisitics of COVID-19 in New York City. N Engl J Med. 2020 April. DOI: 10.1056/NEJMc2010419

**2.2 Laboratory findings (Table 5):** Several laboratory abnormalities are described. Blood cultures were usually sterile unless complicated by secondary infection and septicemia. Despite a dominant pneumonia like clinical picture, serum pro-calcitonin levels are usually normal. Ferritin is often elevated in presence of severe anemia (11). High D-dimer level have been associated with severe and fatal disease and may be due to subclinical process in micro-circulation of widespread hemolysis and/or thromboembolism.

**Table 5: COVID-19 LABORATORY FINDINGS (LITERATURE REVIEW^$^)**

| **Study** | **Guan et al.^1^** | **Chen et al.^2^** | **Zhou et al.^3^** | **Richardson et al.^4^** | **Huang et al.^5^** |
| --- | --- | --- | --- | --- | --- |
| **Site** | China | China | China | USA | China |
| **Design** | Retrospective  Observational  Multicenter | Retrospective  Observational  Single center | Retrospective  Observational  Multicenter | Retrospective  Observational  Multicenter | Retrospective  Observational  Single center |
| **Nature of Site** | Hospital | Hospital | Hospital | Hospital | Hospital |
| **Sample** | 1099 | 99 | 191 | 5700 | 41 |
| **Age, median (IQR), y** | 47 (35-58) | 55 (R, 21-82) | 56 (46-67) | 63 (52-75) | 49 (41-58) |
| **Male (%)** | 58 | 68 | 62 | 60 | 73 |
| **Laboratory findings (%)** | | | | | |
| **Leucopenia**  **(<4000/mm^3^)** | 38 | 9 | 17 | - | 25 |
| **Leucocytosis**  **(>10000/mm^3^)** | 6 | 24 | 21 | - | 30 |
| **Lymphocytopenia**  **(< x10^9^/L)** | 83 | 35 | 40 | 60 | 63 |
| **Thrombocytopenia**  **(< 1.5L /mm^3^)** | 36 | 12 | 7 | - | 5 |
| **CRP**  **(> 5 mg/L)** | 61 | 86 | - | 79 | - |
| **Procalcitonin**  **(≥0.5ng/ml)** | 5 | 6 | 9 | 73 | 8 |
| **LDH**  **(≥250 U/L)** | 41 | 76 | 67 | 70 | 73 |
| **D-dimer**  **(≥0.5 mg/L)** | 46 | 36 | 42 | 56 | - |
| **Interlukin-6**  **(>7 pg/mL)** | - | 52 | - | - |  |
| **Ferritin**  **(≥400 ng/mL)** | - | 63 | 80 | 76 | - |
| **ALT**  **(≥40 U/L)** | 21 | 28 | 31 | 39 | - |
| **AST**  **(≥40 U/L)** | 22 | 35 | - | 58 | 37 |
| **Albumin**  **(<3.5 g/dL)** | - | 98 | - | - | - |
| **Creatine kinase**  **(> 185 U/L)** | 14 | 13 | 13 | - | 33 |
| **Creatinine**  **(≥ 133 mmol/L)** | 2 | 3 | 4 | - | 10 |
| **Troponin I**  **(> 28 pg/mL)** | - | - | 17 | 23 | 12 |
| **Brain-type natriuretic peptide**  **(> 99 pg/mL)** | - | - | - | 32 | - |

CRP: C-reactive protein, LDH: lactate dehydrogenase, ALT: alanine transaminases, AST: aspartate transaminases, IQR: interquartile range, R: range

**$ References:**

1. Wei-jie Guan, Zheng-yi, Yu Hu, Wen-hua Liang, Chun-quanOu, Jian-xing He, et.al. Clinical Characteristics of Coronavirus Disease 2019 in China. N Engl J Med. 2020. DOI: 10.1056/NEJMoa2002032.
2. Nanshan Chen, Min Zhou, Xuan Dong, Jieming Qu, Fengyun Gong, Yang Han, et al. Epidemiological and clinical characteristics of 99 cases of novel coronavirus pneumonia in Wuhan, China: a descriptive study. The Lancet. 2020. Downloaded from <https://doi.org/10.1016/> S0140-6736(20)30211-7
3. Fei Zhou, Ting Yu, Ronghui Du, Guohui Fan, Ying Li, Zhibo Liu, et al. Clinical course and risk factors for mortality of adult inpatients with COVID-19 in Wuhan, China: a retrospective cohort study. The Lancet. 2020;395:1054-1062. DOI:https://doi.org/10.1016/S0140-6736(20)30566-3.
4. Safiya Richardson, Jamie S. Hirsch; Mangala Narasimhan, et al. Presenting Characteristics, Comorbidities, and Outcomes Among 5700 Patients Hospitalized With COVID-19 in the New York City Area. 2020. DOI:10.1001/jama.2020.6775.
5. Chaolin Huang, Yeming Wang, Xingwang Li, Lili Ren, Jianping Zhao, Yi Hu, et al. Clinical features of patients infected with 2019 novel coronavirus in Wuhan, China. 2020; 395:467-536. DOI:https://doi.org/10.1016/S0140-6736(20)30183-5.
   1. **Imaging:**

Chest radiographs may be normal in early or mild disease. However, 50% patients in an asymptomatic cohort showed several abnormal findings on chest computed tomography (CT)- typical ground-glass opacities or patchy shadowing and some atypical abnormalities (12)*.* Patchy lung parenchymal consolidation and ground glass opacities, often bilateral, peripheral, and lower lung zone distributions are often demonstrated (18). Less common findings include pleural thickening, pleural effusion, vascular thickening and lymphadenopathy. Though the overall picture appears non-specific, certain characteristic features can distinguish COVID-19 from other pulmonary disorders and in particular viral pneumonitis, in a patient with a high index of clinical suspicion.

- 1. **Diagnosis**

A confirmatory diagnosis is obtained when a nose/throat swab tests positive for the COVID-19 using real time RT-PCR technology. The details of specific lab diagnosis are appended to this protocol.

- 1. **Classification and Staging of Disease;**

There is no universally accepted system to classify or stage the disease into various categories like mild, moderate or severe at onset of symptoms or during the early part of the disease. A large proportion of patients remain asymptomatic or with mild symptoms and recover completely as described above. It is also easier to comprehend the severe stage characterized by progressive respiratory failure or ARDS invariably necessitates need for supplemental oxygen or a ventilation assist device. Pneumonia is often an early occurrence along with fever, cough and breathlessness in the large majority and patients are expected to recover without any severe progression of respiratory status or any other complication. So the presence of pneumonia does not necessarily mean severe COVID-19. It may be more suitable to label COVID-19 severe if there is a low arterial oxygen saturation (as evident by pulse oxymetry) and a requirement of oxygen and or ventilation device and some of these patients will progress to a life threatening situation which needs critical ICU support in an ‘emergency situation’. Objective measures of respiration and cardiovascular system can define the severity of the disease in the few patients who rapidly worsen. As mentioned above (See Table 3), there are several other complications which are life threatening and can turn the balance towards a severe or an emergency status; most important being septicemia and septicemic shock. It must be remembered that patients with mild disease at onset can infrequently rapidly progress to severe disease and that some patients may die within few days of onset of illness.

An expert group has described 4 stages as follows; Mild (without any risk factors/ comorbidities and managed as outpatient), Moderate (dyspnoea, hypoeximia, and infiltrates/ consolidation on chest on X-Ray/ CT scan, and needs to be hospitalized for treatment), Severe (severe respiratory illness , respiratory rate > 30/min, SPo2- <93%, PaO2/FiO2 <300, and lung infiltrates >50% within 24- 48 hours and requiring ICU care ), Critically Ill (ICU care , respiratory failure and/or ARDS with need of mechanical ventilation, Septic shock and Multiple organ dysfunction syndrome and cytokine storm syndrome). (International Pulmonologist’s Consensus On Covid-19.Available at [https://www.unah.edu.hn/ dmsdocument/](https://www.unah.edu.hn/%20dmsdocument/)9674-consenso-internacional-de-neumologos-sobre-covid-19-version-ingle. Accessed at 03 May 2020).

The WHO has classified COVID-19 into Mild, Pneumonia, and Severe Pneumonia Acute respiratory distress syndrome, sepsis and septicemic shock. Mild disease is characterized byuncomplicated upper respiratory tract viral infection with nonspecific symptoms such as fever, fatigue, cough (with or without sputum production), anorexia, malaise, muscle pain, sore throat, dyspnea, nasal congestion, or headache; rarely, patients may also present with diarrhea, nausea, and vomiting (Older and/or immunosuppressed patients may present with atypical symptoms). In the pneumonia stage, the patients have typical features of pneumonia on clinical examination and imaging) X-Ray, CT Scan) but do not have any of the features of severe pneumonia or need supplemental oxygen as described for severe pneumonia. In the Severe Pneumonia stage, patients have severe respiratory disorder with respiratory rate >30 breaths/minute, severe respiratory distress and SpO₂ ≤93% on room air along with critical support of supplement oxygen and ICU, and may worsen to need mechanical ventilation and other life support systems (World Health Organization. Clinical management of severe acute respiratory infection (SARI) whenCOVID-19 disease is suspected.(Available at <https://www.who.int/publications-detail/clinical-management-of-severe-acute-respiratory-infection-when-novel-coronavirus-(ncov)-infection-is-suspected>. Accessed on 05052020).

The Table below shows the clinical syndromes associated with COVID-19 as described by WHO.


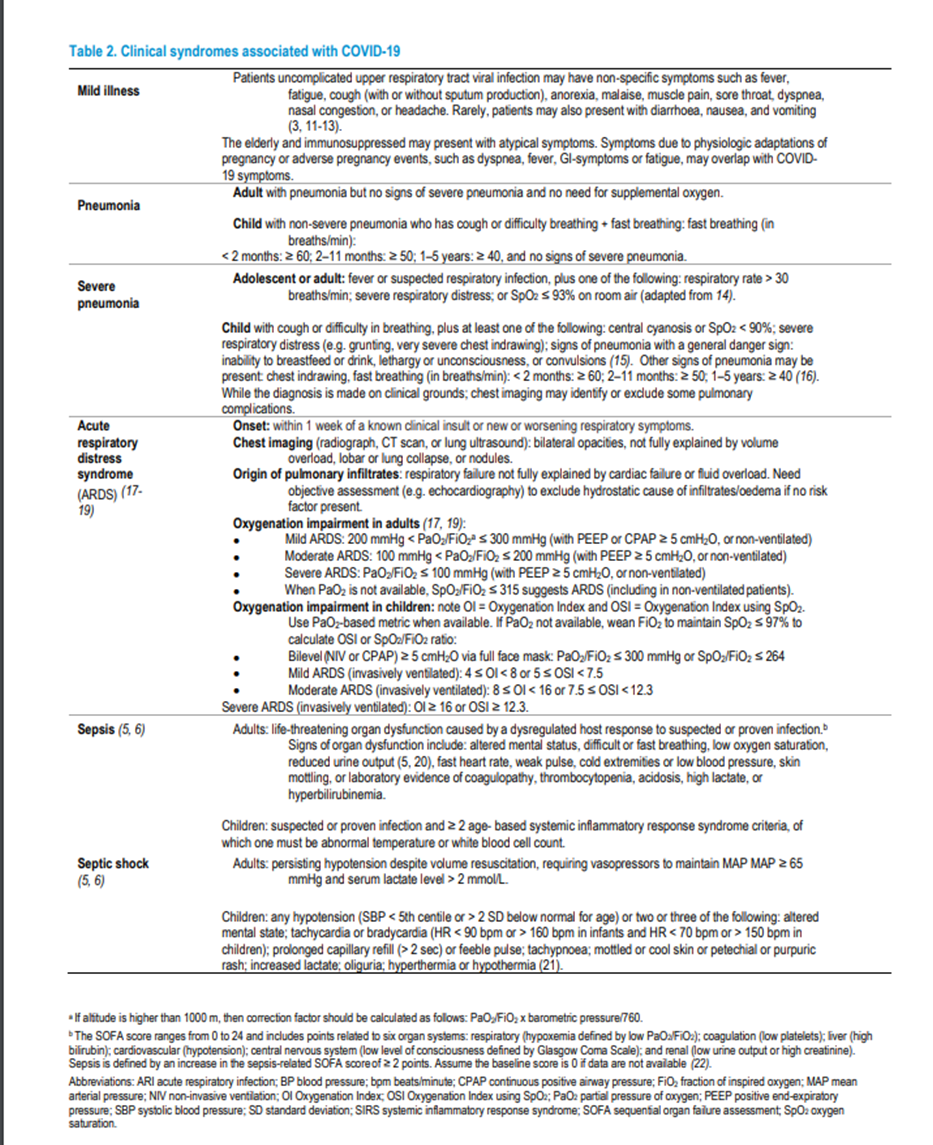


**2.6 Management (Table 6)**

There is no specific drug management of proven value in the treatment of COVID-19. The emphasis is on symptomatic and timely supportive management.

Patients with mild disease do not require hospital-based interventions and can be managed with at home with some isolation to contain the virus transmission. Patients with severe disease and complication like severe acute respiratory infection (SARI), acute respiratory distress syndrome (ARDS), sepsis and septic shock are best managed in an intensive care unit (ICU) and may require oxygen supplement, mechanical ventilation, intravenous antibiotics and other critical hemodynamic and respiratory support. Use of corticosteroids is reserved for progressive severe disease and is not routinely recommended. However, of late, steroids have been observed to contribute to relief of symptoms and promote early recovery when initiated early in moderately severe disease but evidence is still lacking in this regard (19).

The Ministry of Health and Family Welfare GOI has issued guidelines on the clinical management of COVID-19 on 31^st^ March 2020 (20). These guidelines also mention an empirical short use of Hydroxychloroquine (HCQ) in conjunction with azithromycin in the management of COVID 19. The latter is not supported by management guidelines issued by WHO (19). US FDA recently issued a warning against routine use of HCQ in COVID-19.

**Table 6: COVID-19 MANAGEMENT (LITERATURE REVIEW^@^):**

| **Study** | **Zhou et.al^1^** | **Wang et.al^2^** | **Huang et.al^3^** | **Guan et.al.^4^** | **Richardson et.al.^5^** |
| --- | --- | --- | --- | --- | --- |
| **Site** | China | China | China | China | USA |
| **Study type** | Retrospective  Observational  Multicenter | Retrospective  Observational  Single center | Prospective  Observational  Single center | Retrospective  Observational  Multicenter | Prospective  Observational  Multicenter |
| **Nature of Site** | Hospital | Hospital | Hospital | Hospital | Hospital |
| **Sample Size** | 191 | 138 | 41 | 1099 | 5700 |
| **Pharmacologic treatment (%)** | | | | | |
| **Antiviral** | 21 (lopinavir/  retonavir) | 90  (Oseltamivir) | 93  (Oseltamivir) | 36  (Oseltamivir) | -- |
| **Antibiotic** | 95 | 64 (moxifloxacin)  25(ceftriaxone)  18  (azithromycin) | 100 | 58 | -- |
| **Steroids** | 30 | 45 | 22 | 19 | -- |
| **Iv IG** | 24 | -- | -- | 13 | -- |
| **Supportive treatment (%)** | | | | | |
| **Nasal cannula O2** | 21 | 77 | 66 | 41 | -- |
| **Non invasive mechanical ventilation** | 14 | 11 | 24 | 5 | -- |
| **Invasive mechanical ventilation** | 17 | 12 | 5 | 2 | 12 |
| **ECMO** | 2 | 3 | 5 | 0.5 | -- |
| **Renal replacement therapy** | 5 | 1 | 3 | 1 | 3 |
| **Outcome (%)** | | | | | |
| **Still hospitalized** | -- | -- | 17 | 94 | 57.5 |
| **Recovery/discharge** | 28 | -- | 68 | 1/5 | 36.5 |
| **Death** | 72 | 4.3 | 15 | 1 | 21 |

Iv IG- Intravenous immunoglobulin, ECMO- Extra corporeal membrane oxygenation,

**@ References:**

1. Fei Zhou, Ting Yu, Ronghui Du, Guohui Fan, Ying Li, Zhibo Liu, et al. Clinical course and risk factors for mortality of adult inpatients with COVID-19 in Wuhan, China: a retrospective cohort study. The Lancet. 2020;395:1054-1062. DOI:https://doi.org/10.1016/S0140-6736(20)30566-3.
2. Dawei Wang, MD1; Bo Hu, MD1; Chang Hu, MD1; et al. Clinical Characteristics of 138 Hospitalized Patients With 2019 Novel Coronavirus–Infected Pneumonia in Wuhan, China. JAMA. 2020;323(11):1061-1069. DOI:10.1001/jama.2020.1585.
3. ChaolinHuang,Yeming Wang, Xingwang Li, Lili Ren, Jianping Zhao, Yi Hu, et al. Clinical features of patients infected with 2019 novel coronavirus in Wuhan, China. 2020; 395:467-536. DOI:https://doi.org/10.1016/S0140-6736(20)30183-5.
4. Wei-jie Guan, Zheng-yi, Yu Hu, Wen-hua Liang, Chun-quanOu, Jian-xing He, et.al. Clinical Characteristics of Coronavirus Disease 2019 in China. N Engl J Med. 2020. DOI: 10.1056/NEJMoa2002032.
5. Safiya Richardson, Jamie S. Hirsch; Mangala Narasimhan, et al. Presenting Characteristics, Comorbidities, and Outcomes Among 5700 Patients Hospitalized With COVID-19 in the New York City Area. 2020. DOI:10.1001/jama.2020.6775.

**2.6 Experimental Drugs:**

HCQ has been extensively used for over several decades to treat rheumatoid arthritis and lupus. HCQ predominantly effects the immune processing of the self-antigen or other pathogenic molecules by effecting glycosylation on cell surface and pH changes in the lysosomes through a physicochemical process (21). Chloroquine (CQ) analogs are shown to exhibit *in vitro* nonspecific antiviral activity at high micro molar concentration against a broad range of emerging virus (HIV, dengue, hepatitis C, Chikungunya, influenza, Ebola, severe acute respiratory syndrome (SARS)-associated coronavirus and MERS (Middle East-SARS) viruses and more recently COVID-19 (22). CQ also affects glycosylation of ACE-2, the receptor that SARS-CoV-2 uses to enter cells (23). HCQ was extensively used to treat acute Chikungunya illness during the epidemic in India in 2006 and later all over the Asia without any concrete clinical evidence. However, it failed to show efficacy in a randomized controlled study (24).

The rationale for clinical use of HCQ to treat COVID-19 is not conclusive and there is insufficient data from relatively small sample size drug trials (25, 26, 27, 28). Another quasi-randomized design observational study (not yet published) from USA reported an adverse outcome in hospitalized patients of COVID-19 who received HCQ as compared to only standard of care (no HCQ) when confounders were controlled (29). However HCQ may be used in the current pandemic situation as a Monitored Emergency Use of Unregistered Interventions (MEURI) (30).

There is an urgent need to investigate newer therapeutic agents but HCQ does not seem to be a priority (31). There are several concerns regarding empirical use of drugs in the management of COVID-19 in absence of good evidence generated from controlled clinical studies (32). Table 7 shows some of the important features of recently concluded drug trials to treat COVID 19. Several more ongoing drug trials are investigating the use of experimental drugs and to name some- Remdesivir, Favipiravir, Lopinavir/Ritonavir, Ivermectin, Niclosamide, Tocilizumab (anti IL-6), Baricitinib, Tofacitinib, Gimsilumab, and Eculizumab (33).

WHO has initiated a global drug trial (called the SOLIDARITY study) to evaluate the efficacy of four investigational interventions in the medical management of COVID 19 (34). A synopsis of the ‘Solidarity’ study is enclosed with this protocol (see Appendix B).

- 1. **Current state of prophylaxis**

There is no vaccine available at this point of time although several candidates are in different stages of development all over the world and it is expected to have some potent and safe and approved vaccine by early 2021 (35). The only effective prophylaxis is personal protection. The overarching principal is containing virus spread through social distancing and minimizes man-to-man contact, stringent personal and hand hygiene and protection by wearing face masks. Health Care providers closely involved with the management of COVID-19 cases are advised to wear special personal protection gear to avoid contamination from close andextensive contact (8). Hospitals are expected to observe a higher extent of discipline and caution because COVID-19 has demonstrated extensive nosocomial spread in several reports.

An advisory has been issued by ICMR on the empirical chemoprophylaxis use of HCQ in COVID-19; a weekly course for seven weeks is described after a single day loading dose for high risk health care workers but no clinical evidence or rationale is provided (36). These ICMR guidelines very appropriately target high risk health care providers and community contacts of COVID-19 positive cases. The advisory very rightly warns against the known toxicity of HCQ and with special reference to cardiac. The ICMR guideline on HCQ prophylaxis has come in for sharp criticism in a recent report (37).

**Table 7: DRUG TRIALS TO TREAT COVID-19 (LITERATURE REVIEW***)**

| **Study** | **Investigational Drug** | **Design**  **Arms**  **Control**  **(n)** | **Duration**(days) | **Outcome** | **Adverse events** | **Remark** |
| --- | --- | --- | --- | --- | --- | --- |
| **Tang et al.^1^** | Hydroxychloroquine (HCQ)  1200mg x 3 days f/b  800mg for 2 weeks in Mild/Moderate and for 3 weeks in severe disease | Randomised, open label  2 arms,  75 SOC plus HCQ  75 SOC  (150) | 28 | 1. No difference in seroconversion rate at 28 days.  2. Significant normalisation of CRP from baseline (Absolute change 6.986 v/s 2.71, p=0.045).  3. Modest improvement in lymphopenia (p=0.546) | Diarrhoea (10% vs 0%, p=0.004) | 1.Anti virals can be confouding factors |
| **Gautret et al.^2^** | Hydroxychloroquine plus Azithromycin  HCQ 200md TID  Azithromycin 500mg on day 1 f/b 250mg day 2-5 | Retrospective observational  (80) | 10 | Beneficial effect of adding azithromycin to HCQ viral load reduction/disappearance |  | 1.No data on cardiac monitoring.  2.Small sample size.  3.Observational. |
| **Grein et al.^3^** | Remdesivir  200mg IV on day 1 f/b 100mg IV daily for 10 days | Open label, Non-randomized, Phase III  1 arm (53) | 10 | Clinical improvement seen in 68%. (36/53) | Hepatic enzyme dysfunction(23%), Diarrhoea(9), Rash(8), Renal impairment (8), Hypotension(8) | 1.Shoer duration and follow up.  2.Small size cohort  3.Not blinded or randomised |
| **Cao et al.^4^** | Lopinavir/Ritonavir  400+100mg BD | Randomised controlled, open label SOC  2 arms, 99(Lopinavir/Ritonavir+ SOC)  100(SOC) (199) | 28 | 1.Not difference in clinical improvement and mortality at the end of 28 days.  2.Do not decrease viral load/ RNA detectability | Lymphopenia(17%), Nausea(9.5%), Leucopenia(7%) | 1.Not blinded.  2. Use of steroids confounding factor |
| **Zha et al.^5^** | Corticosteroids  Methylprednisolone (MP) 40mg OD or BD for 5 days | Observational, Phase III  2 arms  11 (MP+SOC)  20(SOC) (31) |  | No difference in outcomes in patients with ARDS. Delay Viral clearance. | Liver injury (46% vs. 35%) | 1.Small size.  2.Young age group (Median age 39y) |
| **Luo et al.^6^** | Tocilizumab | Retrospective, observational | - | Significant fall in CRP and IL 6 level from baseline.  IL6 can be used for prognosis |  | 1.Use of steroids- confounding  2.Small sample  No placebo |
| **Cia et al.^7^** | Favipiravir  1600mg on day 1 f/b 600mg day 2-14 | Open label, Non-randomized  Phase III  2 arms  35 favipiravir  45 lopinavir/ritonavir (80) | 14 | Better effects in terms of disease progression and viral clearance | Diarrhoea (6% vs. 11%)  Nausea ( 0 vs. 13%), vomiting (0 vs. 11%) | 1.No randomisation or blinded study |

SOC: standard of care.

*****References**:

1. Tang W, Cao Z, Han M, Wang Z, Chen J, Sun W, et al. Hydroxychloroquine in patients with COVID-19:an open label, randomized, controlled trial. BMJ. 2020.

doi: <https://doi.org/10.1101/2020.04.10.20060558>

1. Philippe Gautret, Jean-Christophe Lagier, Philippe Parola, Van Thuan Hoang, Line Meddeb, MorganeMailhe, et al. Hydroxychloroquine and azithromycin as a treatment of COVID-19: results of an open-label non-randomized clinical trial. Int J Antimicrob Agents. 2020 Mar 20 : 105949. doi: 10.1016/j.ijantimicag.2020.105949 .
2. Jonathan Grein, Norio Ohmagari, Daniel Shin, George Diaz, Erika Asperges, Antonella Castagna, et al. Compassionate Use of Remdesivir for Patients with Severe Covid-19. N Engl J Med. 2020.

DOI: 10.1056/NEJMoa2007016

1. Bin Cao, YemingWang, Danning Wen, Wen Liu, Jingli Wang, Guohui Fan, et al. A Trial of Lopinavir–Ritonavir in Adults Hospitalized with Severe Covid-19. N Engl J Med. 2020. DOI: 10.1056/NEJMoa2001282.
2. Lei Zha, Shirong Li, Lingling Pan, Boris Tefsen, Yeshan Li, Neil French, et al. Corticosteroid treatment of patients with coronavirus disease 2019 (COVID‐19). Med J Aust 2020. doi: 10.5694/mja2.50577
3. Luo P, Liu Y, Qiu L, Liu X, Liu D, Li J.Tocilizumab treatment in COVID‐19: A single centerexperience. J Med Virol. 2020;1–5.https://doi.org/10.1002/jmv.25801LUO.
4. Qingxian Cai, Minghui Yanga, DongjingLiua, Jun Chena, Dan Shua, Junxia Xia, et al. Experimental Treatment with Favipiravir for COVID-19: An Open-Label Control Study. Engineering. 2020. <https://doi.org/10.1016/j.eng.2020.03.007>

# 3. RATIONALE FOR PROPOSED STUDY

The absence of specific drug treatment and lack of vaccine is a critical gap in the knowledge of COVID-19 pandemic (38). And in this regard it is important to refer to a seminal paper published on the ‘responsibility and accountability of conducting standard research including randomized clinical drug trials by the medical community’ in times of epidemics, and the authors have used the recent Ebola virus epidemic in Africa to highlight their contention (39).

The Ministry of AYUSH, GOI has recommended the self-care guidelines for preventive health measures and boosting immunity with special reference to respiratory health based on Ayurvedic literature and scientific publications. However, this advisory does not claim to be treatment for COVID-19. The role of Ayurveda Rasayana as an immunomodulator is well known and should be investigated in COVID-19 (40). In this protocol, we describe the rationale for using three Ayurveda herbal formulations individually to treat COVID-19 as an add-on/adjunct therapy to standard of care. The three Ayurveda formulations are AYUSH-64, Sanshamani Vati Plus and Yashtimadhu. In India the guidelines issues by the Ministry of Health and Family Welfare are generally followed as standard of care (See above, Section 2.6 (20). It is difficult if not impossible to expect a uniform nature of standard of care in the study site hospitals in this study.

Patients suffering from symptomatic Mild to Moderate COVID-19 will be selected for this study. By ’Mild to Moderate’ it would essentially mean that patients do not require any of the emergency measures that are advocated for severe and life-threatening cases of COVID-19. The latter would include complications of progressive lung disease, septicemia, shock and other complications (see Table 3). The mild to moderate cases of COVID-19 will belong to the categories of ‘Mild’ and ‘Pneumonia’ as described by WHO (See WHO Table on Syndromes associated with COVID-19 shown in Section 2.6 above). Mere need for supplement oxygen using non-invasive means will not negate the classification of mild to moderate COVID-19.

**3.1 AYURVEDA RATIONALE**:

The rationale is based on elaborate information and understanding of these formulations in classic Ayurveda texts and published literature and experimental data.

Ayurveda, an Indian traditional system focuses on a holistic approach to health promotion and disease prevention (41, 42). In clinical practice this is a whole system approach which individualizes the treatment to meet the requirements of an individual’s complaint pertaining to health or illness. Lifestyle management, which includes diet, daily routine and behavior, is intrinsic to the holistic approach. The holistic interventions of Ayurveda employ several non-pharmacological measures, which are somewhat difficult to standardize in a clinical research setting such as a drug trial.

The salient general features of Ayurveda that may be relevant to manage COVID-19 are summarized as follows:

- Ayurveda offers public health approach of health promotion and disease prevention
- Health promotion of Ayurveda has a potential role in COVID-19 prevention
- Ayurveda interventions aim at boosting immune status urgently needed in present crisis
- Home based measures (gargles, medicated water, oil pulling, nasya etc.) may control virus entry
- Rasayana approach is crucial for pre-infection and post-infection prophylaxis
- Reports on immunomodulation, adaptogenic potential of Ayurveda suggests potential in prophylaxis treatment (43).

Studies on pre-infection and post-infection prophylaxis are need of the hour. Ayurveda describes a special class of medicinal botanicals used as Rasayana therapy, which includes medicines, diet and behavior. Though used in Ayurvedic practices since many centuries, several clinical and biological investigations have demonstrated the Rasayana to be effective in immunomodulation, rejuvenation, regeneration, adaptogenic and restoration of immune homeostasis. Several research reports on the immunomodulation and adaptogenic properties of Rasayana lend credence to its immense therapeutic potential both for prevention and treatment of a large spectrum of medical disorders irrespective of etiology, chronicity and pathogenesis (44). The immunity status of the host is critical to fighting against any illness but more so in case of infectious disorders, autoimmune disorders, malignancy and any illness characterized by immunosuppressive state.

Here we describe the rationale of the three Ayurveda formulations namely AYUSH-64, Sanshamani Vati Plus and Madhuyasthi which, will be evaluated for their individual effectiveness and related health benefits in the management of COVID-19 in this protocol.

**A.** **Sanshamani Vati Plus (SV +):**

The proposed study includes a combination of *Tinospora cordifolia* (Guduchi) and *Piper longum* (Pippali) as one of the Ayurvedic formulations*.* Both these botanicals are very well studied and documented for their anti-viral, anti-inflammatory and immunomodulatory potential (Rasayana). The details of ingredients and related research are annexed as Appendix C.

The ingredients are used in Ayurveda practice for various indications including respiratory (*Shvasa, Kasa, Jvara*) and infectious diseases (45). This particular combination has been described in the treatment of ‘*Vata Kapha Pradhana Sannipatika Jwara’* (VKJ) which shares symptomatology similar to COVID-19. The proposed formulation is indicated in the VKJ, as described in Ashtanga Hridaya (a text of 8^th^ century, which is referred as one of the three important Ayurveda classics known as ‘*Brihadtrayee’*)

कफवाते .. पिप्पलीचूर्णयुक्तोवाक्वाथछिन्नोद्भवोद्भवा (अष्टांगहृदयज्वरचिकित्सा६०)

Sanshamani Vati Plus is a dried aqueous extract of *Tinospora cordifoila* (TC) used to treat VKJ and immunocompromised conditions in various disorders. Both the botanicals are abundantly available in the Indian sub-continent. Its immune modulating function has been effectively used in treatment of HIV and Chikungunya (46, 47). The clinical profile of COVID-19 is similar to the Ayurvedic description of VKJ and ‘Shvasa Kasa Jwara’ and thus the stated formulation has a potential to treat COVID-19. While TC will target the immune system, Pippali will improve the respiratory component. The advisory by the AYUSH Ministry for COVID-19 has also recommended *Sanshamani Vati* for improving immunity (48).

Dosage: *Samshamani Vati* Plus will be given as two tablets (each containing 300 mg aqueous extracts of *Tinospora cordifolia* and 75 mg aqueous extracts of *Piper longum*) twice daily with warm water. However, the Ayurveda clinician can increase the dose (up to four tablets per day) considering the symptoms, dosha dominance and patient’s need. In such a case the attending Ayurveda physician will document every change in dose everyday with its reason.

**B. AYUSH – 64:** This is a poly-herbal formulation developed by Central Council for Research in Ayurvedic Sciences (CCRAS), Ministry of AYUSH, Govt. of India that targets VKJ (49) and has also been shown to be useful in the management of malaria (50, 51). The AYUSH-64 has been used in more than 13,000 patients during the malaria epidemic of 1984 (52). The advisory by the AYUSH Ministry for COVID-19 has recommended AYUSH – 64 for symptomatic management. The formulation consists of- *Saptaparna* (*Alstonia scholaris* R. Br.) bark aqueous extract 100 mg., *Katuki* (*Picrorhiza kurroa* Royle ex. Benth) root extract 100 mg., *Kiratatikta* (*Swertia Chirata* Pexbex. Karst) whole-plant extract 100 mg. and *Kuberaksha* (*Caesalpinia crista* L.) seed powder 200 mg. (Appendix D).

The experimental studies of AYUSH 64 have shown that it was safe and non-toxic in the dose of 500 mg/kg of body weight for 12 weeks**.** It is found to be effective in fevers of unknown etiology, filarial lymphangitis and derangement of liver function besides its anti-malarial activity (53, 54, 55).

The ingredients of AYUSH 64 are *Saptaparna* (*Alstonia scholaris* R. Br.) *Katuki* (*Picrorhiza kurroa*Royle ex. Benth), *Kiratatikta* (*Swertia chirata* Pexbex. Karst) and *Kuberaksha* (*Caesalpinia crista* Linn.). Various Ayurveda classics describe activities and properties of the ingredients of AYUSH 64 in respiratory diseases and also acute infectious diseases with pulmonary complications. The ingredients are effective in diseases of Kapha (कफघ्न), and Vata (वातघ्न), improve *Agni,* (अग्निदीपन), digest *Ama* (आमपाचन), strengthen tissues, relieve symptoms (श्वासघ्न, कासघ्न, ज्वरघ्न, शोथघ्न), and restore health. Based on the clinical experience of physicians who had successfully used AYUSH 64, to treat Influenza like Illness (ILI), a pilot study was recently carried out by CCRAS (56). In this clinical observational study, 28 out of 30 participants recovered satisfactorily and did not require any other drug. It is against the latter experience that it was decided to evaluate AYUSH 64 in the treatment of COVID-19. Studies on the ingredients of AYUSH 64 has shown anti-inflammatory and immunomodulatory activities. In animal study, the alkaloids from *Saptaparna* inhibited the production of inflammatory cytokines TNF-α and IL-8 in the broncho-alveolar lavage fluid from the lung (57).In an in-vitro molecular study, whole plant extracts of *Swertia chirata* inhibited the expression of viral protein (Vpr) of Herpes Simplex Virus Type 1 in a cell culture (Hela cells harbouring the TREX plasmid encoding full-length Vpr) (58, 59).

Dosage: The dose of AYUSH - 64 will be two tablets (500 mg each) twice daily. Each tablet will contain aqueous extracts of *Alstonia scholaris* (100 mg), *Picrorhiza kurroa* (100 mg), *Swertia chirata* (100 mg), and *Caesalpinia crista* (200 mg). However, the Ayurveda clinician can increase the dose (up to six tablets per day) considering the symptoms, dosha dominance, and patient’s need. In such a case the attending Ayurveda physician will document every change in dose everyday with its reason.

**C. Yashtimadhu** ([*Glycyrrhiza glabra*](https://www.sciencedirect.com/topics/pharmacology-toxicology-and-pharmaceutical-science/glycyrrhiza-glabra)): Commonly known as [licorice](https://www.sciencedirect.com/topics/pharmacology-toxicology-and-pharmaceutical-science/liquorice) (family Fabaceae) is one of the most important and widely experienced herbal drug in clinical practice from Ayurveda pharmacopeia. It is popularly also called ‘Mulethi’ in India and has been popularly used since times immemorial as a popular household remedy in Indian home to treat common cold, flu like illness and sore throat. This plant is also popularly used in the Traditional Chinese Medicine to treat cough, colds asthma, and COPD since ancient times (59). (Appendix E)

Yashtimadhu is used as a medicine to strengthen the respiratory system (स्वर्य, कण्ठ्य). It is considered to nourish and contribute to healthy growth and function of tissues and strengthens immune status (बल्य, क्षयापह) in a general way. It is a potent medicine in alleviation of all three Doshas and inflammatory response (शोथघ्न). All these medicinal properties are likely to makes it an attractive choice of medicine to treat COVID-19. There is clinical evidence that this formulation used as a Chinese Medicine prevented severe acute respiratory syndrome (SARS) and H1N1 influenza (Cinatl, J.; Lancet 2003). Literature review shows that there is a renewed clinical and research interest in mainland China on using this formulation in the prevention and treatment of COVID-19 (Liqiang Wang Acta et al Pharmaceutica Sinica B). Yashtimadhu contains several active compounds, including glycyrrhizin, glycyrrhetinicacid, [flavonoids](https://www.sciencedirect.com/topics/pharmacology-toxicology-and-pharmaceutical-science/flavonoids), [isoflavonoids](https://www.sciencedirect.com/topics/pharmacology-toxicology-and-pharmaceutical-science/isoflavonoids), and chalcones. Glycyrrhizin and glycyrrhetinic acid are considered the major constituents for plant activity of clinical therapeutic significance. The extract of liquorice roots is rich in glycyrrhizinate and recently the compound diammonium glycyrrhizinate has been selected in a control study of COVID-19 (*Chinese clinical trial:*<http://www.chictr.org.cn/showprojen.aspx?proj=49131>) (61). Beside this *Glycyrrhiza glabra*, has long been employed against coughs and colds as well as to settle disturbed digestion. The compound diammonium glycyrrhizinate has anti inflammatory activity and has been used to treat liver damage caused by hepatitis B (Jun-ling Ren et al Pharmacological Research). Professor Hong Ding (Wuhan University, China) has recently proposed a combination of diammonium glycyrrhizinate and vitamin C to treat COVID-19 and clinical trials have recently been approved (*Editorial: Nature Plants 2020*).

Thus, there is a good rationale to select Yashtimadhu as a potential research drug to treat COVID-19 in this protocol.

Dosage: The dose of *Glycyrrhiza glabra* will be two tablets (300 mg each) twice daily.

Several details of the standardization and ingredients of the Study Ayurvedic Formulations are described in Appendix to this protocol (See Content for Appendix C, D and E). However, the Ayurveda clinician can increase the dose (up to six tablets per day) considering the symptoms, dosha dominance and patient’s need. In such a case the attending Ayurveda physician will document every change in dose everyday with its reason.

# 4. OBJECTIVE OF THE STUDY

**PRIMARY**

To compare the efficacy and safety of a combination regimen of Standard of Care (SOC) plus a selected standardized Ayurvedic drug (adjuvant) in the management of mild to moderate COVID-19 with that of standalone SOC (active control).

**SECONDARY**

1. To determine the therapeutic effect of a combination of SOC plus a selected standardized Ayurvedic drug on surrogate markers of COVID-19 disease severity and complications, and recovery pertaining to broad based domain of acute phase reactants, pro-inflammatory cytokines, imaging assessment, anti-oxidant activity, organ dysfunction and damage
2. To determine the early and late post-recovery health benefits of a combination of selected standardized Ayurvedic drug plus SOC using qualitative measure of health status (WHO Quality of life assessment questionnaire) and health related behaviour, habits and fitness questionnaire
3. To describe the clinical profile of COVID-19 with special reference to early symptoms, severity of disease, complications, course of disease, diagnostic investigations, biochemical and imaging abnormalities, patterns of recovery
4. To determine the predictors of early and late recovery from COVID-19 using a therapeutic combination of SOC plus a selected standardized Ayurvedic drug

# 5. STUDY DESIGN

Fig 1 shows the study flow and the overview of study design and timelines, and principal study events including clinical examinations, laboratory and other investigations.

Each of the three selected and standardized Ayurveda drugs i.e. **AYUSH 64 (A-64), Sanshamani Vati Plus (SV +)** and **Yashtimadhu (MYT)** will be assessed as add on or adjunct treatment in three different drug trials of two arms each for efficacy and safety under a common current protocol.

Each of the drug trial will be a randomized, prospective, open label, parallel efficacy, active control two arm multicentre study. Each of the three Ayurveda drugs is being considered as an adjuvant drug to Standard of Care (SOC). The SOC and Ayurveda drug combination is the experimental interventional arm which will be compared to the active control arm of standalone SOC. The total duration of each of the three drugs’ study is 12 weeks and this period includes time to clinical recovery (defined later in text) and a period of follow up to complete 12 week of observation following admission to the hospital. The nature of SOC and any follow up medication will be decided by the treating physician. Some variation in SOC between different study sites and even between patients is possible. The study investigators will meticulously record the details of SOC in the study case record form of each patient. The dose of Ayurvedic drugs is as per standard Ayurveda practise and decided by consensus amongst experts in the AYUSH working group for this patient. The total sample size of each drug study is 140 patients with 70 patients randomized to each arm.

**Fig 1: Study Flow diagram showing Study Events and Timelines**

Screening of Voluntary and Consenting In-patients suffering from Mild to Moderately Severe COVID-19 for Eligibility as per Inclusion-Exclusion Criteria

3 Ayurveda formulation drug trial studies under a common protocol

(Separate study for each AYUSH 64 / Yashtimadhu / Samshamai Vati Plus)

Randomize Eligible Hospitalized Patients

(Open Label, Parallel Efficacy, Active Control Standard of Care (SOC), Two Arm Study)

Selected Ayurveda Formulation (Adjuvant) Versus SOC (Active Control)

Time Points

Clinical Assessment

Investigations

Baseline

Case Record Form (CRF), Medical History Clinical Examination, Ayurveda Assessment, Health Status (WHOQs), QoL (HR-BHF)

Diagnostic (PCR), Serology (IgM, IgG), Routine Hematology Profile, Special Assays, X-Ray Chest & Imaging, ECG

Week 4

Brief Clin Exam, Ayu Exam, Health Status (WHO- Qs) & QoL (HR-BHF Qs), Adverse Events (AEs)

Diagnostic (PCR), Serology (IgM, IgG), Routine haematology Body Profile, Special Assays

Week 8

Brief Clin Exam, Ayu Exam, Health Status (WHO- Qs) & QoL (HR-BHF Qs), Adverse Events (AEs)

Diagnostic (PCR), Serology (IgM, IgG), Routine haematology Body Profile, Special Assays

Week 12

Completion Outcome Assessment, CRF Clin Exam, Ayu Exam, WHO- Qs & QoL (HR-BHF Qs), AEs

Diagnostic (PCR), Serology (IgM, IgG), Routine haematology Body Profile, Special Assays

***Further Assessment Time Points**: (i) During Hospitalization: Daily monitored vital parameters, disease symptoms, disease progression and recovery, and investigations carried out as per protocol till DISCHARGE; Laboratory and Imaging investigations as per protocol but the study investigator and treating physician can do any additional test as per their judgment (ii)Post DISCHARGE: Daily Telephonic Contact using a special mobile phone application for any relapse or any other symptoms or complications or any other Issue (iii) Discharge: Complete all formalities as required for study completion (iv) Skip week 4 or week 8 end point evaluation within 2 weeks of DISCHARGE

The total study period is 12 weeks; Primary Efficacy is ‘Clinical Recovery’ as described in the protocol; Study participant developing severe COVID 19 or severe complication and requiring special critical support/ ICU/ ventilator support will be withdrawn from the study but followed up to record outcome; Both Ayurvedic and Allopathic Physicians will jointly examine and monitor study participants in addition to the hospital treating physician.

Patients will be selected from the outpatient or inpatient facility in COVID-19 medical centres/hospitals. The study will be explained to the volunteer patients and those willing to sign the informed consent will be screened for eligibility as per the inclusion and exclusion criteria in the protocol. Patient suffering from severe COVID-19 will be excluded (see exclusion criteria below).

# CONSENT, SELECTION, SCREENING, and ENROLLMENT

Study patients will be served on first come first serve basis as per the protocol.

Consenting and eligible patients will be enrolled as soon as possible in a COCID-19 management facility and the study intervention begun. It is important that symptomatic patients suspected for COVID-19 on clinical grounds are quickly evaluated for eligibility. It is important to enroll the patient as close to the onset of symptoms as possible. As described above, this study is dealing with patients of COVID who are ‘non-emergent’ which means mild and moderate disease (pneumonia) which does not require emergency or critical care (see section on inclusion and exclusion criteria).

An ‘Patient Information Sheet’ (Appendix F) in the language understood by the patient will be provided to each patient for study and discussion prior to enrollment. The information fact sheet will describe the overall design of the study and the rationale, benefit and side effect profile of the study drug interventions, and other aspects of the study.

Patients will be selected from the outpatient or inpatient facility in COVID-19 medical centres/ hospitals. The study will be explained to the volunteer patients and those willing to sign the informed consent (Appendix G) will be screened for eligibility as per the inclusion and exclusion criteria in the protocol.

Though it will be preferable to enrol patients with diagnosis confirmed by a positive RT-PCR assay using a nasal or throat swab in a standardized procedure, a patient with a clinical diagnosis of COVID- 19 as described below will be eligible.

Eligible patients will be enrolled using a simple randomization table (provided to the investigator) in a block of 20 patients each to either of the two study arms - SOC plus Ayurveda drug or SOC. As the target sample size is 70 patients in each arm, the first block of randomized allocation will be of 10. The study randomization will be carried out by the investigator.

# CLINICAL EVALUATION INCLUDING AYURVEDIC ASSESSMENT

Both Allopathic and Ayurvedic physicians, and predesignated study personnel will carry out assessment of all study patients as per the study schedule of events and timelines shown in Fig 1. and in Appendix A.

Several of the patient centric assessments including health and quality of life measures will be completed by the study paramedic without the knowledge of the drug allocation of the patient (assessor blind).

Standard study case record forms will be used on patient enrolment (baseline) to record patient data and will included demographics, personal habits, comprehensive medical history and examinations, measures of health and quality of life (Appendix H). The Ayurveda physician will also record the required clinical parameters of the study patient in the Ayurveda Case Record Form (Appendix I).

The routine hospital case record sheet will be used to record the patient’s progress while in hospital. The study investigator and the Ayurvedic physician will extract the relevant data of the patient from the hospital case sheet on a daily basis and enter it into the ‘study progress sheet’. (Appendix J).

On completion of treatment and at time of discharge, the investigators will complete the study completion case record form which will also include parameters on outcome (recovery or death) (Appendix K). The study patients will be provided with a precise follow-up schedule for the post treatment evaluation and during which the patients will be assessed as per schedule of events (Table 1) at weeks 4, 8 and 12 (study completion). It should be noted that some patients may require a longer period to recover that may extend from 3-4 weeks and in which case the assessment at discharge will also comply with the requirements of week-4 time endpoint (see Table 1).

During the study period, the study patient will complete a self-reported questionnaire on COVID 19 related symptoms daily which will be provided in the form of a mobile application. The self-reported questionnaire is enclosed in Appendix L.

All patients withdrawn for any reason will complete a medical examination and complete other documents as required on study completion (see above). If possible, laboratory evaluation as described above for study completion will also be done.

Laboratory and imaging investigations will be carried out as per Table 1 and are further described below.

It should be noted that study investigators will work closely with the hospital physicians in a co-operative and positive manner so as to offer the best possible management to the patient and avoid any unnecessary discomfort of any kind to the patient arising from study participation. It is prudent to state that while the success of clinical drug trials usually depends upon strict compliance with the requirements stated in the study protocol with particular reference to Schedule of Events, see Table 1), the interest of the patient and his/her wellbeing is of topmost priority and the investigators may use their judgment and discretion to modify the study requirements to serve that purpose. However, the latter will be a protocol deviation and clearly recorded by the senior study investigator with an explanatory note on its compulsion in the study completion case record form.

# 8. STUDY DURATION, FOLLOW- UP SCHEDULE AND PROCEDURES

This is summarized in Figure 1 and Table 1. There are several foot notes provided in Table 1 to further clarify a particular event/test procedure and often with particular reference to the role of the study investigator. Several of the clinical events with reference to clinical examination, study record forms and questionnaires, study procedures, laboratory diagnostics and assessments are elaborated in Appendices enclosed with the protocol (Appendix A to S).

It should be noted that this is a study treatment protocol and that the time to outcome in terms of recovery following onset of illness and/or admission to hospital is a varying parameter. The large majority of study patients are expected to recover completely but based on prior knowledge the mortality of COVID-19 in severe case in well documented (see literature review, section above). It is expected that the majority of patients will recover and be discharged to home within 5 days to 15 days of hospitalization.

However, all study patients who recover are required to follow with the study investigators on pre-determined time points as shown in Table 1.

The total duration of the study is 12 weeks and includes the duration of treatment and post clinical recovery or post-hospital discharge period to ensure optimum recovery and regain of health status.

# 9.WITHDRAWAL AND PREMATURE TERMINATION

The patient can withdraw from the study at any point of time without assigning any reason.

The study investigator can also withdraw the patient for reasons arising out of the study drug intervention such as- intervention treatment failure, progression to severe disease, requirement of critical support care (ICU), requirement of persistent oxygen support, need for ventilation, drug related toxicity, protocol violations etc. All patients withdrawing from the study will be requested to complete the formalities (including laboratory evaluation) of study completion case record form and outcome/ hospital discharge. However, they will be encouraged to continue the clinical and other assessments enumerated for post treatment study schedule in Table 1.

There are several other reasons for patient to withdraw from the study such as:

1. Patients who worsen or need ICU admission or ventilator support due to any reason
2. Non-compliance of the treatment regimen
3. Participants not willing to continue the study
4. Drug related toxicity
5. Any serious adverse event that makes the participant unable to do the activities of daily living / any SAE
6. Any other serious medical condition which the participant may acquire during the course of the trial which makes it difficult for the participant to continue in the trial
7. Any other condition which the investigator feels might cause harm to the participant

All adverse events (AE), and in particular serious adverse event (SAE) will be dealt with as described in the section on AE (see below).

The study investigator s will be required to keep an informal contact with such a study patient uptp the study completion at 12 weeks and record post study withdrawal clinical developments. The probable outcomes of such a study patient will be stated in the case record form and include asymptomatic status, days of hospitalization, symptomatic disease and its severity, time to discharge, time to recovery, death, post recovery status as stated in the patient completion case record form and outcome report

# 10. PATIENT POPULATION

The patients will be largely drawn from COVID-19 clinics and outpatients in various medical colleges and hospitals. All patients will be admitted in the hospital for standard care and those willing to participate in the study will be screened and enrolled as described above. Only patients with mild to moderately severe symptoms will be selected as described above. If the patients progress to a severe stage of illness as described below, they will be withdrawn from the study but the investigator will be encouraged to follow the patient ill study completion at week 12 and during this period record the hospital management, course of illness , final outcome and post recovery health status as described in this protocol.

- 1. Inclusion Criteria:

1. Typical Clinical presentation of acute onset febrile illness with cough and a RT_PCR based laboratory confirmation test for COVID-19
2. Patients with either sex, 21to 69years age
3. Patients with Mild to Moderate COVID-19 as described below
4. All patients must agree not to share medication
5. Patients willing to participate and sign an informed consent
   Note: The criteria for mild to moderate COVID-19 are based on the WHO recommendations (See Section 2.5 above with WHO Table on clinical syndromes of COVID-19 and include Mild disease and Uncomplicated Pneumonia but not severe disease which requires any form of intensive care such as invasive techniques for supplemental oxygen or ventilation and life support systems, and absence of any life threatening complications (such as severe respiratory failure, adult respiratory distress syndrome, septicaemia shock and other complications shown in Table 3 above)
   1. Exclusion Criteria
6. Patients suffering from severe COVID-19 Disease as described above in Section 10.1 and judged by a physician; further guidance can be taken to exclude patient in a clinical context of progressive respiratory dysfunction and failure from the following criteria (i) Respiratory distress at room ambience (≥30 breaths per min) (ii) Oxygen saturation at rest ≤93% (peripheral digital arterial oxymetry) and requiring oxygen support for over one hour to normalize (iii) Any of the known COVID-19 complications and emergency procedures which may require shift/admission in intensive care unit such as respiratory failure, adult respiratory distress syndrome, requirement of oxygen support for over 1 hour, requirement of mechanical ventilation, septic shock, or severe non-respiratory organ dysfunction or failure.(62)
   (Adapted and modified from the reference: Yang Liu et al.Lancet Infect Dis 2020 , March 19, 2020 https://doi.org/10.1016/ S1473-3099(20)30232-2)
7. Chronic, Severe, Unstable, Uncontrolled co-existent medical illness such as Diabetes, Hypertension, Cardiac disorders, liver, kidney disorders and lung disorders or other disease of concern which may put the patient at increased risk during the study
8. History of immunosuppression: solid organ or bone marrow transplant, use of immunosuppressive antimetabolic and biologic agents, intrinsic immunodeficiencies, HIV infection.
9. Active cancer diagnosis, on palliative treatment or requiring current therapy with antimetabolic agents, immunotherapy or radiotherapy.
10. Patients on parenteral nutrition
11. Patients with known sensitivity or contraindication to any of the ingredients of study medication
12. History of bleeding haemorrhoids, haemoptysis, acid peptic diseases, ulcers and pulmonary diseases (tuberculosis, asthma, etc)
13. Patients who are likely to worsen or planed ICU admission or ventilator support due to any reason
14. Pregnancy and lactation
15. Participation in a drug interventional clinical drug trial of any nature in the three-month period preceding onset of COVID-19
16. Participation in any other clinical trial of an experimental agent treatment for COVID-19
17. Patients on any kind of Ayurveda treatment or any other alternative and complementary medicinal systems such as Homeopathy, Unani, Siddha and in particular requiring oral therapy of any kind
18. Patients receiving any other test drug, apart from SOC.
19. Physician decision that involvement in the study is not in the patient´s best interest

# 11. EFFECTIVENSS MEASURES

**11.1 Primary Efficacy:**

1. Mean time (days) for clinical recovery (Day of randomization to the Day of clinical recovery (see criteria below] [Time Frame: up to 28 days]
2. Proportion of patients showing ‘clinical recovery’

**Criteria of ‘Clinical Recovery’**:

1. Normal body temperature (≤36.6°C axilla or ≤37.2 °C oral)
2. Absence of cough or mild cough (infrequent, short episodic, non-wheezy, relieved by minimal or no medication, not interfering with routine speech and not related to lying in bed, mild sore throat or nasal congestion)
3. Absence of breathlessness on routine daily self-care chore or respiratory rate less than 30 breaths per minute without supplemental oxygen
4. Absence of any other symptom/sign attributed to COVID-19 illness
5. Normalization of SpO2 by standard peripheral oximetry device (above 95 percent)
6. Recovery should be sustained for at least 48 hours under physician observation
7. Assessed by physician blinded to treatment allocation
8. All of the above criteria ought to be fulfilled

Note: Clinical recovery would be deemed from the first day of satisfying the above criteria

**11.2 Secondary Efficacy:**

1) Rate of patients with negative SARS-CoV-2 on nasal or throat swab in a 2-day continuous real time RT-PCR test beginning from ‘first day of clinical recovery’ (see above) or ‘Day 10 after onset of symptoms depending on whichever of the two time points is first achieved

2) Timelines (days counted from onset of illness) - normal body temperature <37◦C, absence or minimal cough (see ‘clinical recovery’ for the definition), absence of dyspnoea (respiration rate 24/minute or less on room air), onset of clinical pneumonia, pneumonia diagnosed on chest X-Ray or CT scan, time to supplemental oxygen, admit in intensive care unit, mechanical ventilation (non-invasive), mechanical ventilation (invasive), steroid use, respiratory failure, adult respiratory distress syndrome, cytokine storm syndrome, secondary infection, shock, septicaemia shock, hospital discharge, death, negative nose or throat swab confirmatory test, all-cause mortality (Time Frame: up to 28 days).

Note: In case of symptoms, the absence on improvement should be sustained for at least 72 hours or live hospital discharge, whichever comes first.

3) Proportion OF patients developing an event that reflects clinical or otherwise improvement of worsening (the events are similar to those listed under Timelines, see above)

4) Improvement on pulmonary function tests using simple ‘home expiratory spirometer’ device and peripheral pulse oximetry

5) Improvement in selected laboratory parameters: blood haemoglobin, differential and total leukocyte counts, liver enzymes, renal functions, acute phase reactants, serum IL-6 and other selected cytokines, serum muscle enzymes (CK, CPK), serum ferritin, serum d-Dimer, anti-oxidant markers, serum BNP (cardiac function)

6) Serological Protective Antibody Assay (IgM and IgG)

7) Radiological Improvement on digital chest Xray and HRCT chest

8) Drug related: side effects and toxicity, and tolerability

9) Health status: WHO QOL (Appendix M) brief, health related behaviour habit and fitness questionnaire based on visual analogue scale (See Appendix N)

# 12. AYURVEDIC MEASURES

**Baseline**:

a) Prakriti

b) Clinical Features

c) Ayurvedic Disease Subtypes / Stages

**Follow Up / Monitoring**:

a) Clinical Features:

b) Ayurveda examination:

c) Ayurvedic Disease Subtypes / Stages

# 13. LABORATORY & OTHER INVESTIGATIONS

These are common to the each of the three Ayurveda formulation drug trials:

1. Blood/Serum (routine): Haemogram, Platelet count, Total leukocyte differential count, Hemoglobin and ESR, Blood sugar level (Fasting), Liver function test and Liver Enzymes (SGOT,SGPT, Alkaline Phosphatase), Renal functions (Serum creatinine, Blood Urea Nitrogen), Lipid profile (Total cholesterol, HDL cholesterol, LDL cholesterol, Triglycerides, VLDL)
2. Serum (inflammatory markers, organ damage):C-Reactive protein titer, lactate dehydrogenase, ferritin, Pro-Calcitonin, Troponin, D-Dimer,
3. Special tests: Blood for Vitamin D, Vitamin B12, Oxidation Biomarkers (Superoxide Dismutase (SOD), Glutathoine (GSH)
4. Cytokine Panel: Interleukin (IL) IL-2, IL-1 beta, IL-4, IL-6,IL-10, IL-13, IL-17, TNF-α, Monocyte Chemotactic Protein-1 (MCP), Interferon gamma, TGF-bet
5. Serum Protective Antibody Response: IgM and IgG against SARS-CoV-2
6. Routine Urinalysis, Urine Pregnancy Test for women of child bearing potential
7. Confirmatory Diagnostic Test: Nasal and/or Throat swab for real time RT-PCR for SARS-CoV-2 (See Appendix O)
8. Other Investigations: USG Abdomen and Pelvis, ECHO-Color Doppler and 12 Lead ECG

Chest X-ray, HRCT scan Chest

**13.1** Rationale for Cytokine Assay: Cytokine assay can be used to study important anti-viral effects (gamma interferon), immune mediated inflammation (IL-6, anti-TNF, IL-17), TH 1 and TH 2 immune response and antibody producing B cell activity (Il 4, IL 13), activation of immune cells such as macrophage activation (MCP). Intense up regulation and elevation of IL 6 and several other cytokines has been reported by several clinical case series and research in COVID-19 and can guide specific therapy (as in case of use of monoclonal antibody to IL-6 receptor being used to treat Cytokine storm in COVID 19).

Detail methods for diagnostic tests, serology and cytokine assay are described in Appendix O.

# 14. SAFETY ISSUES AND ENDPOINTS, ADVERSE EVENTS

All adverse events will be recorded and meticulously followed till resolution. Serious adverse events will require immediate action and further communication by the study investigator. AE will be classified by the study physician as described in Appendix P and classified as shown in Appendix Q and recorded in the AE form by the investigator along with its grading (Appendix R).

A-priori check list of common drug related side as a ready reckoner will be provided to all investigators so that timely action may be taken if a patient reports any kind of adverse event that may be related to the study intervention. The patients will be encouraged to record any kind of symptom that they may think is due to a drug in their personal diary which will be scrutinized by the study physician at monthly visits and on completion.

The following ‘**Stop rules’** will be applied to withdraw the patient prematurely because of safety concerns:

1. Patients developing severe COVID-19 as described above under the ‘exclusion criteria’
2. Requiring hospitalization for disorders other than COVID-19 will be critically evaluated by the study physician to decide continuity in the study
3. Any adverse clinical or lab profile that in the opinion of the investigator is likely to be due to therapeutic intervention and threatens to deteriorate the wellbeing of the patients

Data Safety Monitoring Board (DSMB) will be established to monitor safety of the participants and assess safety data. The CONSORT extension for HARMS will be used to document and report safety data.

# 15. STUDY PROCEDURES: TIMELINES AND EVENTS SCHEDULE

The overview is shown in Table 1.

15.1 Baseline Visit: (Visit 1)

Patients who meet the criteria for selection, must have the following procedures completed during this visit prior to study entry.

Visit 1 (Baseline)

|  | Providing with Patient Information Fact Sheet for thorough study. |
| --- | --- |
|  | Signing of Informed Consent. |
|  | Complete medical examination form: medical history, comprehensive physical examination, co-morbidity, medication history. |
|  | Symptom Assessment, Vital Parameters |
|  | Record Baseline Symptoms |
|  | Comprehensive Ayurvedic evaluation. |
|  | Laboratory work up- general health and advanced test and cytokines as in Appendix O |
|  | Ultrasound abdomen and Pelvis, Chest X-ray, HRCT chest, |
|  | Colour Doppler and 12 Lead ECG |
|  | Pulse Oximetry |
|  | Health Related-Behaviour Fitness (HR-BHF) VAS questionnaire, Self-Reported Questionnaire (Mobile app) |
|  | Dispense study medication and begin medication log entry |

15.2 Visit 2 (Day 4):

|  | Symptom Assessment, Vital Parameters |
| --- | --- |
|  | Brief Ayurvedic Evaluation |
|  | Health Related-Behaviour Fitness (HR-BHF) VAS questionnaire, Self-Reported Questionnaire (Mobile app) |
|  | Laboratory work up |
|  | Chest X-ray, HRCT chest, |
|  | Pulse Oximetry |
|  | Monitor Adverse Events |

15.3Visit 2 (Day5 up to-Discharge) (Daily Assessment):

|  | Symptom Assessment, Vital Parameters |
| --- | --- |
|  | Brief Ayurvedic Evaluation |
|  | Laboratory work up |
|  | Pulse Oximetry |
|  | Monitor Adverse Events |
|  | Colour Doppler, ECG |
|  | Chest X-ray, and HRCT chest |
|  | Health Related-Behaviour Fitness (HR-BHF) VAS questionnaire, Self-Reported Questionnaire (Mobile app) |
|  | Completion and Outcome Report |

15.4DISCHARGE

|  | Symptom Assessment, Vital Parameters |
| --- | --- |
|  | Brief Ayurvedic Evaluation |
|  | Laboratory work up |
|  | Pulse Oximetry |
|  | Monitor Adverse Events |
|  | Colour Doppler, ECG |
|  | Chest X-ray, and HRCT chest |
|  | Health Related-Behavior Fitness (HR-BHF) VAS questionnaire, Self-Reported Questionnaire (Mobile app) |
|  | Completion and Outcome Report |

15.5. Visit 4 (week 4)

|  | Symptom Assessment, Vital Parameters |
| --- | --- |
|  | Brief Ayurvedic Evaluation |
|  | Laboratory work up |
|  | Pulse Oximetry |
|  | Monitor Adverse Events |
|  | Health Related-Behaviour Fitness (HR-BHF) VAS questionnaire, Self-Reported Questionnaire (Mobile app) |
|  | Drug Compliance, Dispense study medication |

15.6 .Visit 5 (week 8)

|  | Symptom Assessment, Vital Parameters |
| --- | --- |
|  | Comprehensive Ayurvedic Evaluation |
|  | Medical History, Comprehensive Physical examination |
|  | Laboratory work up |
|  | Pulse Oximetry |
|  | Monitor Adverse Events |
|  | Health Related-Behaviour Fitness (HR-BHF) VAS questionnaire, Self-Reported Questionnaire (Mobile app) |
|  | Drug Compliance, Dispense study medication |
|  |  |

15.7. Visit 6 (Week 12)

|  | Symptom Assessment, Vital Parameters |
| --- | --- |
|  | Comprehensive Ayurvedic Evaluation |
|  | Medical History, Comprehensive Physical examination |
|  | Laboratory work up |
|  | Pulse Oximetry |
|  | Monitor Adverse Events |
|  | Health Related-Behaviour Fitness (HR-BHF) VAS questionnaire, Self-Reported Questionnaire (Mobile app) |
|  | Drug Compliance |
|  | Completion Report |

# 16. INVESTIGATIONAL DRUGS AND DOSING

The investigational drugs to be used individually in 3 separate drug trials of 2 arms each in this protocol are Sanshamani Vati Plus, AYUSH 64 and *Glycyrrhiza glabra*. The dosage schedule for each one of them is as follows:

i) AYUSH-64: 500 mg tablet, 2 tablets bid (twice daily)

ii) Yashtimadhu: 300 mg tablet, 2 tablets bid (twice daily)

iii) Sanshamani Vati Plus: Each tablet to contain 300 mg Guduchi plus 75 mg Pippali, 2 tablets bid (twice daily) with warm water.

**Botanical Test Material:** The ingredients of all the test materials used in this clinical study as investigational drug formulations are aqueous botanical extracts. All the botanical test materials (*Tinospora cordifoila, Piper longum, Alstonia scholaris, Picrorhiza kurroa, Swertia Chirata, Caesalpinia crista,* and *Glycyrrhiza glabra*) are manufactured in accordance with highest quality standard requirements. The test material formulation will be manufactured at GMP compliant facility using WHO guidelines on good herbal processing practices for herbal medicines (2017). The test material has been prepared from high quality botanical material carefully procured through approved vendor qualification program and authenticated by In-House taxonomist for each batch. The test material formulation has been tested as per the quality control requirements of Indian Pharmacopeia (2018) to analyze at least two analytical markers by HPLC for consistent fingerprinting and quality of finished products. LCMS profile may be obtained to ensure consistency in ratio of main peaks. The other mandatory testing includes heavy metals analysis, microbiological parameters, aflatoxins, residual solvents, and ash or water content to be carried out In-house for each batch of formulations (tablet) by the quality control department. Current certifications at the facility where test drugs will be prepared include Quality Management System (ISO 9001:2015), Food safety management (ISO22000:2005), NSF-GMP (21 CFR 111 & 117) with Kosher and Halal, and USDA/APEDA organic certifications for liable products. The facility premises contain a separate area for quality assurance, quality control, and research development departments. The manufacturer will produce valid Certificate of Analysis for every batch. The facility where test materials are prepared has well-structured and secured premises guarding the area with CCTV enhanced security systems. In short, the botanical test materials will be manufactured as one batch in accordance with Indian and WHO standards for AYUSH / herbal / botanical drugs. The reference samples of the raw material and extract will be preserved.

The medicines and other interventions being used as standard of care will be documented systematically with its dosage, duration, manufacturing company etc. The variations in the interventions being implemented as standard of care, if any, will be noted in case record form.

**16.1 Compliance:**

The number of tablets and capsules dispensed at each visit will be recorded in the medication log. On each return visit, the patient will be instructed to bring all unused medication and a “pill count” will be performed and recorded in the medication log.

**16.2 Concomitant Medication/Therapy:**

Patients will continue to consume any medication for concomitant illness like diabetes etc. permitted as per exclusion criteria and the under supervision of their primary care physician. Other medications required for any other illness during the study will be taken under supervision of the prescribing physician but the same will be informed to the study investigator.

Concomitant medications are to be kept to a minimum during the study. However, if these are considered necessary for the patient’s welfare and do not interfere with the study medication, they may be allowed at the discretion of the investigator. If the administration of any concomitant treatment is necessary it should be reported in the case record form (preferably: generic name), dosage, duration and indication for use in the patient’s medical records.

# 17. STATISTICAL DESIGN, SAMPLE SIZE ANDANALYSIS, DATA BASE

As described above, there are 3 Ayurveda plant formulations and each will be tested separately for clinical efficacy and safety as an adjunct/add-on therapy to the SOC in patients suffering from mild to moderate COVID-19 in a non-randomized, open label, parallel efficacy, active control study; active control being the arm with only SOC. The study design is exploratory in nature. This protocol will be used by each of the selected Ayurveda formulations in standalone drug trial evaluation study and in different sites.

**17.1 Sample Size:**

A convenience sample size of 70 patients in each of the arm is selected. The total sample size will be 140 patients in each of the 3 studies. The sample size is not based on any power calculations but by consensus within the experts of the Working Group constituted for the purpose of this protocol. It is assumed that the withdrawal rate may be in the range of 15-20%.

**17.2 Statistical Analysis:**

Whenever suitable, both completer and an intent-to-treat analysis with ‘last observation carried forward’ will be performed; for intent-to-treat analysis the patient should have completed at least 4 weeks of intervention and completed the follow up visit at 4 weeks post enrollment.

All statistical processing will be performed using SPSS & BMDP software unless otherwise stated.

The data will be individually analyzed for central tendencies (mean, median), range, standard error, standard deviation and 95% confidence intervals for each intervention arm in each of the groups in the study. Data will be tabulated and graphically shown using standard format and MS Excel and other software programs. Though ANOVA will be used for most of the measures, standard parametric (Student t-test) and non-parametric statistical tests (Mann Whitney statistic, Kruskal Wallis test) will be used depending upon the normality of the data. Fisher’s exact test or Chi Square will be used to test the proportion of patients classified as efficacy failures and those with AE (calculate exact binomial 95% Confidence Interval).

The primary efficacy measures of ‘mean time to clinical recovery’ and ‘proportion of patients with clinical recovery’ in the two arms will be compared using appropriate statistical tests; ANOVA for ‘mean time’ and two proportion z-test for proportion of patients.

Secondary efficacy measures in the two arms will also be compared and Bonferroni correction will be applied for multiple comparisons.

Risk ascertainment will be done for selected variables. Regression analysis (multivariate and step down) will be used to identify predictors for important outcomes. Survival analysis will be carried out to compare the two interventions (HCQ and Ashwagandha) for primary efficacy at different study time points.

Within arm efficacy will be evaluated by a one sample Student t-test.

Significance for each statistical test result will be considered at two-sided p< 0.05.

**17.3 Database:** Data will be collected from all investigation sites using both electronic transfer and paper records. A central facility will be used to enter data into a central data base by specialized personnel and under co-ordination and supervision of the sponsor. Once the data is locked, secure and specific number of copies will be made as directed by the sponsor. An authorized copy will be sent by the sponsor to the pre-designated Biostatistician for the statistical analysis and preparation of the statistical result report.

# 18. MANAGEMENT ISSUES

18.1 **Protocol Violation**:

Any deviation from the protocol is not permitted, except in an emergency situation. The study investigator and the team will be fully conversant with the main provisions of this protocol and exercise utmost care and responsibility while discharging their duty and also ensure utmost compliance by the patients.

If circumstances permit, the investigator attending such an emergency will contact the Chief Principal Investigator at the earliest opportunity so that a decision can be made whether or not to continue the patient in the study.

Any administrative or technical modification, which does not interfere with the patient’s health interests, should be in writing and filed as amendments to the protocol.

The concerned chief investigator will sign these amendments and obtain early clearance from the concerned ‘Ethics Committee’ and the Principal Investigator of the clinical project before finalizing the amendment for execution.

18.2 **Withdrawal of Study Patient:**

Patient may be withdrawn from the study at their own request or at the discretion of the investigator. In all cases, the reasons why the patient is withdrawn will be entered in the CRF. Several likely reasons of withdrawal from study are listed in Section 9 above. As far as possible, a complete final examination must be carried out in all patients who are withdrawn prematurely before the study completion similar to that required on study completion.

18.3 **Safety Issues**: Safety assessments will be based on results of the physical examinations (including vital signs), laboratory tests and adverse events as described above. It is very important that the physician investigators record every adverse event, irrespective of its causality, meticulously in the patient case records. It will also be mandatory and binding for the study physician investigators to keep close contact and co-ordination with the pre-designated principal physician investigators for this purpose and as per instructions from the Sponsor.

Serious adverse events (SAEs) will be tabulated per patient and reported as per GCP guidelines. Each investigation site will submit an updated list of patients who discontinued from the study and a list of patients who experienced SAE to the pre-designated principal investigators of this study as decided by the sponsor.

# 19. ADMINISTERATIVE ISSUES

**19.1 Study Documents**

A Patient Information Sheet (see Appendix H) will be handed over to each patient participant in an easy to understand question answer format and in the language best understood.

Only the patient’s initials / ID number will be used for identification in the study records and database.

Study confidentiality will be maintained. The following documents will be used in the study:

- Patient Case Record Forms (CRF): This will be provided for each study participant. All centers must follow instructions for filling of CRF that have been listed in the beginning of each CRF file.
- Informed Consent in the regional language best understood by the study participant.
- Adverse Event Form
- Serious Adverse Event Form
- Termination Form
- Medication Log
- WHO- Quality of Life

All the centers will be provided with a file in which to organize and retain all study-related documents. All study correspondence should be filed by the investigator in this file. The Site Master File will be available for inspection by the individual monitoring the study in order to ensure that all the relevant documents are present and easily retrievable.

**19.2 Study Participation Cards**

A study pocket sized card (Appendix S) will be provided to each study participant on enrollment and this will be carried on self during the study period. The card will indicate the Patient ID, study medication and contact details of investigator. The patient will be encouraged to show this card to any other medical practitioners while consulting them for any illness.

**19.3. Mobile phone Application**

A special software program as mobile application (AYUSH COV-2 CT App) will be downloaded by the study participant under supervision of the study investigator at the time of enrollment. The App will have a 2-way communication between participant and local investigator-through SMS and phone call. It will also record DAILY the response of the participant to 5 cardinal questions regarding presence and absence of =Fever, Sore throat, Cough, Breathlessness and Suspected Drug Side Effect. In addition, there will be a 6^th^ question- ‘do you wish to speak to investigator’. The participant will complete the response every day between 8-10 pm. The App will send the response daily to the study computer of the local investigator and central monitoring cell (CMC). In case, the study patient fails to complete the response in time, the App will raise a frequent alarm bell pop up response on the mobile phone of the study participant. In case, the response is YES to any of the questions, there will be an alarm bell type visual and audible response raised both the desktop computer and mobile phone of the local investigator and person in charge CMC. This will ensure speedy necessary action by the local site investigator. The App will stay active throughout the study duration.

**19.4 Ethics Committee Approval**: A copy of the formal written notification approving the study will be obtained from the EC duly signed by the chairman or any person so authorized, prior to commencement.

**19.5 Case Record Forms**: All complete CRFs duly signed by the investigator and counter signed by principal investigator must be submitted within one month of the completion of the study to the Principal Investigator.

**19.6 Study Monitoring**

The study will be conducted in accordance with the principles of Good Clinical Practice (GCP) as laid out in the Indian Council of Medical Research (ICMR) 2017 guidelines, International Conference on Harmonization (ICH) 2017 guidelines on GCP and clinical studies and AYUSH/CCRAS guidelines for Ayurveda research (63-69).

The investigator should ensure that sufficient time is allocated for monitoring activities and for the monitor to meet the appropriate site personnel. The sponsor will arrange monitoring visits to each of the participating centers for evaluating the progress of the ongoing study and identify any unexpected problems/difficulties. During these visits special emphasis will be on checking the study documents.

During the monitoring visits, study monitor will:

1. Examine all source data and CRFs for the correctness and consistency of data, protocol compliance and possible adverse events
2. Discuss inconsistencies in the study data or missing data
3. Ensure that the study medication is correctly stored, dispensed and accounted for.
4. Verify information on the CRFs against source data and proper capturing of the Adverse Events
5. Check adherence to the obligations of the investigator (for documentation and reporting)
6. Review consent forms, in particular the date of consent and signature

Source data will include Case Record Forms and/or copy of any other medical records from hospital and/or referral practice considered relevant by the study investigator. Such verification is an essential element of the Quality Control, as it allows the correction of transcription errors and omissions.

An independent Data Safety Monitoring Board (DSMB) will established to evaluate the data on adverse events (AE) at regular intervals. The DSMB may also consider a need for an interim review to ensure participant safety and emerging outcomes.

**19.7 Record Retention**

All documents relating to the study, including the protocol and the local or core data sheets are the confidential property of the Sponsor and should be regarded as such. Unused CRFs should be returned to Sponsor at the end of the study.

The investigator will retain one copy of the study records (e.g., laboratory printouts, ECG printouts, CRF copies, etc.) and the documentation for least 5 years and await instructions of their final disposal from the sponsor. If the investigator retires or changes employment, custody of the records may be transferred to another suitable person who will accept responsibility for those records. Notice of such transfer will be given in writing to the Sponsor

There will be no destruction or unauthorized alteration of any source records or Case Record Forms.

The Principal Investigator will retain all documentation pertaining to this study in the Medical Records Unit for as long as the study treatment is available for human consumption.

**19.8 Other Documentation**

All the investigators will supply the Principal Investigator with the following:

1. A current signed and dated CV for the investigator and his/her entire study team.
2. Documentation of Independent Ethics Committee’s approval. This should be accompanied by the EC working procedures and the EC Membership List. All these documents should be signed by the EC chairperson or designate.
3. Any local laboratory reference ranges used during the study
4. Copy of the EC approved consent forms (English and local versions)

**19.9 Protocol Amendments**

Should any changes be required to the signed final protocol, a protocol amendment must be prepared and agreed by the Investigators and the respective Ethics Committees before implementation.

Deviations from the protocol should only be made in a medical emergency or as a part of the protocol amendment. In an event of a medical emergency, the investigator must notify the Principal Investigator and the appropriate Ethics Committee within 24 hours.

All the amendments to the protocol must be submitted to the appropriate Ethics Committee before implementation.

**19.10 Window Period**

A 3-5 day window period may be allowed in either side of the scheduled visit for exceptional or any other anticipated reasons at the discretion of the investigator.

**19.12 Standardization of study procedures/Standard Operating Procedures (SOP)**

Following the approval of this protocol by the regulatory body, video-based meetings and discussions will take place with the study team members including investigators to fully comprehend this protocol and ensure uniformity in administration of the various clinical and other procedures required to carry out this study. SOP’s specifically targeting these issues in greater detail are prepared and will be sent to the respective study teams. The SOP’s will complement this protocol

# 20. ETHICAL CONSIDERATIONS

**20.1 Declaration of Helsinki**

The study will be conducted in accordance with the Declaration of Helsinki (1964), as amended in South Africa (1996), Edinburgh (2000) and Washington (2002). (Enclosed Appendix T) (68).

**20.2 Ethics Committee**

The investigator must submit this protocol, plus relevant consent forms and a patient information sheet, for independent review by an Institutional Ethics Committee, and the protocol must be reviewed and approved by this Committee before the start of the study. The Ethics Committee should be constituted as per the guidelines issued by ICMR and endorsed by DCGI (63).

The patient enrollment will not start until satisfactory evidence of ethical approval is obtained in writing.

Amendments to the protocol will be submitted for ethical review before implementation.

**20.3 Regulatory clearances:**

The regulatory clearances will be obtained by the sponsor of the study.

All the guidelines and directions described in Declaration of Helsinki, WHO, ICH-GCP (Jan ’97 version) and DCGI, AYUSH (Ethical Guidelines for Biomedical Research on Human Patients) will be followed (63-69).

This protocol will be registered with CTRI after approval from the Ethics Committee.

**20.4 Funding agency/Sponsor:**

Ministry of AYUSH GOI and CSIR GOI are the principal sponsors and funding agency.

**20.5 Informed Consent**

This is prepared along the ICMR guidelines on GCP 2017) (63).

The investigator must explain the study fully to the patient participating in the study. An information sheet giving details of the study will be provided to the patient for study and discussion if any with the investigator. Sufficient time will be given to the patient to make a voluntary decision to participate. Patient will be encouraged to ask questions and clear all doubts in a face to face interview with the investigator. After fully satisfying with the various provisions of the research study the patient will be asked to sign and date the consent form and this will be then be considered as an informed consent. All consent forms should be signed and dated by the investigator. If the patient is unable to read or write, the consent forms should be signed and dated by the investigator and an impartial witness, to indicate that the patient has fully understood the information and has consented freely.

Consent forms will be reviewed retained by the investigator and reviewed by the study monitor.

**20.6 Confidentiality of Patients:**

All the investigators and their research staff will preserve the confidentiality of the patients taking part in this study. Any information revealed to the Institutional Review Board during the course of this study will be considered confidential. All study documents and report forms, laboratory specimens, and other records available to anyone other than the primary investigator and the research staff will be identified by a coded number to maintain the confidentiality of the patient.

# 21. FINANCING AND INSURANCE

AYUSH-CSIR is the sponsor of the study, has sole responsibility of financing the study.

Adequate Insurance will be obtained for the study participants including the study patients for the required duration of time.

AYUSH will provide insurance coverage with respect to any liability caused by the investigational products and or study related procedures in connection with the clinical study. The terms and conditions will apply as specified in the insurance policy document.

The quantum of compensation for the Serious Adverse Events leading to the death will be given to the patient’s nominee as per recommendations of the EC, DSMB and CDSCO’s expert committee and as per current applicable regulatory guidelines (69).

# 22. ACKNOWLEDGEMENT

Several colleagues, experts, peers, mentors and friends have contributed substantially to the preparation of this protocol and we state them in no particular order. We wish to state that the current on-going spread of the COVID 19 pandemic necessitated a speedy process. And this challenge was met by the collective effort of a big team of selfless and generous men and women dedicated to science and Nation. It was not easy to work in such a cohesive and co-ordinated manner during this lockout period. We cannot possibly thank every individual and several were behind the stage. We thank one and all.

We have received invaluable encouragement from the Honourable Union Minister Dr Harsh Vardhan, MH&FW and Honourable Minister Shri Shripad Naik, MoA GOI. We specially thank Secretary, MoA Vaidya Rajesh Kotecha for the foresighted vision to set a research agenda for AYUSH in COVID-19 and constituted an ‘Interdisciplinary AYUSH Research and Development Task Force’. We thank Dr. Shekhar Mande, Director General CSIR and his colleagues for constant encouragement and support. We thank Ministry of AYUSH and CSIR for giving us an opportunity to participate in this national endeavour to find effective solutions to control the menace of COVID-19.

We thank AYUSH for this tremendous opportunity to participate in this national endeavour to find effective solutions to control the menace of SARS-CoV-2. Several members of the ‘Task Force’ and ‘Working Group’ are the authors of this protocol. A working group was set up to address the requirement of Clinical trials for prophylaxis and management chaired by Dr G G Gangadharan, Dr Arvind Chopra and Dr Rama Jayasundar. We acknowledge the contribution of ideas, suggestions, relevant material and guidance from several members namely-, Dr Ram Manohar, Dr SR Narahari, Dr PLT Girija, and Dr N Mehrotra.

We wish thank few distinguished scientists for enriching discussions right from conceptualization stage. Interactions with these experts helped us to quickly formulate this project. We wish to specially mention interactions with Dr Vijay Raghavan, Dr Vinod Paul, Dr. (Lt.Gen.) Madhuri Kanitkar, Dr N.K. Ganguly, Dr. V.M. Katoch, Dr. Soumya Swaminathan, Dr Vasantha Mutthusamy, Dr Nandini Kumar, Dr Madhu Dikshit who offered very good suggestions and constructive comments regarding overall concept. We specially thank Dr Mohan Gupte for more insights to epidemiology, statistics and study design; Dr Ram Vishwakarma, Dr Dr Vishwajananani Sattigeri, and Dr Lal Hingorani for invaluable guidance towards the botanicals and quality of test materials.

We thank DG ICMR for facilitating technical guidance and support. During the preparation of the protocol, there were several interactions with experts from ICMR, GOI and we thank them wholeheartedly. We thank ICMR for technical guidance and support. During the preparation of the protocol, there were several interactions with experts from ICMR namely Prof YK Gupta, Dr MUR Naidu, Dr R Rao, Dr Vijay Kumar. We are indebted to them for reviewing some of the core issues of study design and offering guidance. We specially thank Prof YK Gupta for providing guidance and holding several video conference sessions to discuss the various issues with this research project. In addition to reviewers from ICMR, we specially thank other eminent independent reviewers (Appendix U) who offered constructive comments in very short time which has immensely helped in improvising the quality of the protocol. We are grateful to all reviewers for valuable time and constructive comments, which helped in improvising quality of the protocol.

We cannot thank enough our ‘always available colleague’ from AYUSH Dr JLN Sastry, for his excellent administrative handling of meetings and Ayurveda inputs towards this protocol.

Several of our younger colleagues have also earned our gratitude and namely- CRD Pune: Dr Rahul Patil, Dr Hardik Rathod ;CCIH members: Dr Arti Nagarkar, Dr Preeti Chavan-Gautam, Dr Swapnil Borse, Dr Santosh Dixit, Mr Aksah Saggam, Ms Vedika Bhat; Scientists from Serum Institute of India Dr Suresh Jadhav, Dr Sunil Gairola, Dr Manish Gautam, Dr Dada Patil.

Finally, we wish to thank some of our parent institutions. Arthritis Research Care Foundation Center for Rheumatic Diseases and School of Health Sciences, Savitribai Phule Pune University, Kasturba Health Society, Mumbai, CSIR-IIIM Jammu, CCRAS, AIIA Delhi, provided important logistic and infrastructural material support and spared important staff to assist in the protocol preparation.

# 23. PROTOCOL ACCEPTANCE AND INVESTIGATORS SIGNATURE

A Randomized, Open Label, Parallel Efficacy, Active Control, Multi-Centre Exploratory Drug Trial to Evaluate Efficacy and Safety of an Ayurvedic Formulation as Adjunct Treatment to Standard of Care for the Management of Mild to Moderate COVID-19 patients

| By: _________________________________ |
| --- |
| Print Name: |
| Title: Principal Investigator & Clinical Co-ordinator |
| Date: ________________________________ |

By signing below on this document as a Principal Investigator, I agree to follow this protocol without deviation and in accordance with the rules and regulations regarding ethics, medical research and experiments in India.

By: _____________________________

Principal Investigator

Print name: ________________________Dated ________________

# 24. REFERENCES

1. COVID-19 Map - Johns Hopkins Coronavirus Resource Center . 2020 [cited 2020 Apr 20]. Available from: <https://coronavirus.jhu.edu/map.html>
2. Zhou P, Yang X Lou, Wang XG, Hu B, Zhang L, Zhang W, et al. A pneumonia outbreak associated with a new coronavirus of probable bat origin. Nature. 2020 Mar 12;579(7798):270–3.
3. Kelso JK, Milne GJ, Kelly H. Simulation suggests that rapid activation of social distancing can arrest epidemic development due to a novel strain of influenza. BMC Public Health. 2009;9.
4. Cantey PT, Chuk MG, Kohl KS, Herrmann J, Weiss P, Graffunder CM, et al. Public health emergency preparedness: Lessons learned about monitoring of interventions from the national association of county and city health official’s survey of nonpharmaceutical interventions for pandemic H1N1. J Public Heal ManagPract. 2013 Jan;19(1):70–6.
5. Macdonald G, Moen AC, St. Louis ME. The national inventory of core capabilities for pandemic influenza preparedness and response: An instrument for planning and evaluation. Influenza Other Respi Viruses. 2014 Mar;8(2):189–93.
6. Coronavirus: The Government’s ‘contain and mitigate’ strategy explained . 2020 [cited 2020 Apr 20]. Available from: <https://www.telegraph.co.uk/global-health/science-and-disease/coronavirus-governments-contain-mitigate-strategy-explained/>
7. Qualls N, Levitt A, Kanade N, Wright-Jegede N, Dopson S, Biggerstaff M, et al. Community Mitigation Guidelines to Prevent Pandemic Influenza — United States, 2017. MMWR RecommReports . 2017 Apr 21 [cited 2020 Apr 20];66(1):1–34. Available from: <http://www.cdc.gov/mmwr/volumes/66/rr/rr6601a1.htm>
8. Essential Commodities Order, 2020 Notification. Gazette of India. 2020;26(D):26–7. [cited 2020 Apr 20] Available at <https://www.mohfw.gov.in/pdf/218645.pdf>
9. Zou L, Ruan F, Huang M, Liang L, Huang H, Hong Z, et al. SARS-CoV-2 viral load in upper respiratory specimens of infected patients. Vol. 382, New England Journal of Medicine. Massachussetts Medical Society; 2020. p. 1177–9.
10. Wölfel R, Corman VM, Guggemos W, Seilmaier M, Zange S, Müller MA, et al. Virological assessment of hospitalized patients with COVID-2019. Nature. 2020 Apr 1;
11. Guan W, Ni Z, Hu Y, Liang W, Ou C, He J, et al. Clinical Characteristics of Coronavirus Disease 2019 in China. N Engl J Med. 2020 Feb 28;
12. Hu Z, Song C, Xu C, Jin G, Chen Y, Xu X, et al. Clinical characteristics of 24 asymptomatic infections with COVID-19 screened among close contacts in Nanjing, China. Sci China Life Sci. 2020;
13. Wu F, Zhao S, Yu B, Chen YM, Wang W, Song ZG, et al. A new coronavirus associated with human respiratory disease in China. Nature. 2020 Mar 12;579(7798):265–9.
14. Chen N, Zhou M, Dong X, Qu J, Gong F, Han Y, et al. Epidemiological and clinical characteristics of 99 cases of 2019 novel coronavirus pneumonia in Wuhan, China: a descriptive study. Lancet. 2020 Feb 15;395(10223):507–13.
15. Xydakis MS, MobarakiPD,Holbrook EH, Geisthoff UW, Bauer C, Hautefort C, et l.Smell and taste dysfunction in patientswith COVID-19.Lancet Infect Dis 2020, published Online April 15, 2020. Available at https://doi.org/10.1016/S1473-3099(20)30293-0.Accessed on 06052020
16. Wang D, Hu B, Hu C, Zhu F, Liu X, Zhang J, et al. Clinical Characteristics of 138 Hospitalized Patients with 2019 Novel Coronavirus-Infected Pneumonia in Wuhan, China. JAMA - J Am Med Assoc. 2020 Mar 17;323(11):1061–9.
17. Huang C, Wang Y, Li X, Ren L, Zhao J, Hu Y, et al. Clinical features of patients infected with 2019 novel coronavirus in Wuhan, China. Lancet. 2020 Feb 15;395(10223):497–506.
18. Lee EYP, Ng MY, Khong PL. COVID-19 pneumonia: what has CT taught us? Vol. 20, The Lancet Infectious Diseases. Lancet Publishing Group; 2020. p. 384–5.
19. Clinical management of severe acute respiratory infection when COVID-19 is suspected. 2020;1–21. [cited 2020 Apr 20] Available from: <https://www.who.int/publications-detail/clinical-management-of-severe-acute-respiratory-infection-when-novel-coronavirus-(ncov)-infection-is-suspected>
20. Revised Guidelines on Clinical Management for COVID-19. 2020;(March). Available from: <https://www.mohfw.gov.in/pdf/RevisedNationalClinicalManagementGuidelineforCOVID1931032020.pdf>
21. Schrezenmeier E, Dörner T. Mechanisms of action of Hydroxychloroquine and chloroquine: implications for rheumatology. Vol. 16, Nature Reviews Rheumatology. Nature Research; 2020. p. 155–66.
22. Colson P, Rolain JM, Lagier JC, Brouqui P, Raoult D. Chloroquine and Hydroxychloroquine as available weapons to fight COVID-19. International Journal of Antimicrobial Agents. Elsevier B.V.; 2020. p. 105932.
23. Ferner RE, Aronson JK. Chloroquine and Hydroxychloroquine in covid-19. BMJ . 2020 [cited 2020 Apr 21];369:m1432. Available from: http://www.bmj.com/lookup/doi/10.1136/bmj.m1432
24. Chopra A, Saluja M, Venugopalan A. Effectiveness of chloroquine and inflammatory cytokine response in patients with early persistent musculoskeletal pain and arthritis following chikungunya virus infection. Arthritis Rheumatol. 2014;66(2):319–26.
25. Yao X, Ye F, Zhang M, Cui C, Huang B, Niu P, et al. In Vitro Antiviral Activity and Projection of Optimized Dosing Design of Hydroxychloroquine for the Treatment of Severe Acute Respiratory Syndrome Main point : Hydroxychloroquine was found to be more potent than chloroquine at inhibiting SARS-CoV-2 in vit. Clin Infect Dis. 2020;2:1–25.
26. Gao J, Tian Z, Yang X. Breakthrough: Chloroquine phosphate has shown apparent efficacy in treatment of COVID-19 associated pneumonia in clinical studies. Vol. 14, BioScience Trends. International Advancement Center for Medicine and Health Research Co., Ltd.; 2020.
27. Chen Z, Hu J, Zhang Z, Jiang S, Han S, Yan D, et al. Efficacy of hydroxychloroquine in patients with COVID-19: results of a randomized clinical trial. medRxiv. 2020 Apr 10;2020.03.22.20040758.
28. Molina JM, Delaugerre C, Goff J Le, Mela-Lima B, Ponscarme D, Goldwirt L, et al. No Evidence of Rapid Antiviral Clearance or Clinical Benefit with the Combination of Hydroxychloroquine and Azithromycin in Patients with Severe COVID-19 Infection. Médecine Mal Infect. 2020 Mar 30
29. Joshua B, Daniel K, Ryan F, Kim Le, Xihui Lin. Clinical Outcomes of HCQ in Hospitalized patients with COVID-19 - A quasi-Randomized Comparative Study. N Engl J Med. 202o
30. Cox E, Antierens A, Bavari S, Carson G, Cavaleri M, Fowler R, et al. Consultation on Monitored Emergency Use of Unregistered and Investigational Interventions for Ebola Virus Disease ( EVD ). 2016;1–6. Available from: <http://www.who.int/ethics/publications/infectious-disease-outbreaks/en/>
31. Baden LR, Rubin EJ. Covid-19 — The Search for Effective Therapy. N Engl J Med. 2020 Mar 18;
32. Kim AHJ, Sparks JA, Liew JW, Putman MS, Berenbaum F, Duarte-García A, et al. A Rush to Judgment? Rapid Reporting and Dissemination of Results and Its Consequences Regarding the Use of Hydroxychloroquine for COVID-19. Ann Intern Med. 2020 Mar 30;
33. Bergman S, Cennimo D, Miller M, Olsen K. Treatment of Coronavirus Disease 2019 (COVID-19): Investigational Drugs and Other Therapies. Medscape . 2020; Available from: <https://emedicine.medscape.com/article/2500116-overview>
34. “Solidarity” clinical trial for COVID-19 treatments . 2020 [cited 2020 Apr 20]. Available from: <https://www.who.int/emergencies/diseases/novel-coronavirus-2019/global-research-on-novel-coronavirus-2019-ncov/solidarity-clinical-trial-for-covid-19-treatments>
35. Landscape of COVID-19 candidate vaccines. 2020;(March). Available from: <https://www.who.int/blueprint/priority-diseases/key-action/novel-coronavirus-landscape-ncov.pdf>
36. Advisory on the use of Hydroxychloroquine as prophylaxis for SARS-CoV-2 infection. 2020. [cited 2020 Apr 20] Available from: <https://www.mohfw.gov.in/pdf/AdvisoryontheuseofHydroxychloroquinasprophylaxisforSARSCoV2infection.pdf>
37. Rathi S, Ish P, Kalantri A, Kalantri S. Hydroxychloroquine prophylaxis for COVID-19 contacts in India. Lancet Infect Dis . 2020 Apr [cited 2020 Apr 20];0(0). Available from: <https://linkinghub.elsevier.com/retrieve/pii/S1473309920303133>
38. Aylward B (WHO), Liang W (PRC). Report of the WHO-China Joint Mission on Coronavirus Disease 2019 (COVID-19). WHO-China Jt Mission Coronavirus Dis 2019 . 2020;2019(February):16–24. Available from: <https://www.who.int/docs/default-source/coronaviruse/who-china-joint-mission-on-covid-19-final-report.pdf>
39. London AJ, Omotade OO, Mello MM, Keusch GT. Ethics of randomized trials in a public health emergency. Diemert DJ, editor. PLoSNegl Trop Dis . 2018 May 17 [cited 2020 Apr 21];12(5):e0006313. Available from: <https://dx.plos.org/10.1371/journal.pntd.0006313>
40. Ayurveda’s immunity boosting measures for self care during COVID-19 crisis. 2020; [cited 2020 Apr 21] 1–4. Available from: <https://www.ayush.gov.in/docs/123.pdf>
41. Chopra A, Saluja M, Tillu G. Ayurveda-modern medicine interface: A critical appraisal of studies of Ayurvedic medicines to treat osteoarthritis and rheumatoid arthritis. J Ayurveda Integr Med. 2010;1(3):190–8.
42. Patwardhan B. Integrity of Ayurveda. Vol. 7, Journal of Ayurveda and Integrative Medicine. Elsevier B.V.; 2016. p. 189–90.
43. Patwardhan B, Chavan-gautam P, Gautam M, Tillu G, Chopra A. Ayurveda rasayana in prophylaxis of COVID-19. Curr Sci. 2020;19(Figure 1):1–3.
44. Balasubramani SP, Venkatasubramanian P, Kukkupuni SK, Patwardhan B. Plant-based Rasayana drugs from Ayurveda. Vol. 17, Chinese Journal of Integrative Medicine. 2011. p. 88–94.
45. Gogte V M. DravyagunaVidnyan. Pune: VaidyamitraPrashan. 2008
46. Kalikar MV, Thawani VR, Varadpande UK, Sontakke SD, Singh RP, Khiyani RK. Immunomodulatory effect of Tinosporacordifolia extract in human immuno-deficiency virus positive patients. Indian J Pharmacol. 2008;40(3):107-10.
47. Sharma V, Kaushik S, Pandit P, Dhull D, Yadav JP, Kaushik S. Green synthesis of silver nanoparticles from medicinal plants and evaluation of their antiviral potential against chikungunya virus. ApplMicrobiolBiotechnol. 2019;103(2):881-891.
48. Advisory published by Ministry of AYUSH, D.O. No. S. 16030/18/2019 – NAM, dated 6 March 2020.
49. Pharmacological Investigations of certain Medicinal Plants and Compound Formulations used in Ayurveda and Siddha; Central Council for Research in Ayurveda & Siddha (CCRAS), 1996
50. Kazim M, Puri SK, Dutta GP, Narasimham MV. Evaluation of Ayush-64 for blood schizontocidal activity against rodent and simian malaria parasites. Indian J Malariol. 1991;28(4):255-8.
51. H Ali. "Ayush -64"--a New Anti Malarial Herbal Compound. Indian J PatholMicrobiol. 1996; 39 (5), 499-500
52. http://www.ccras.nic.in/sites/default/files/viewpdf/IEC_Communication/Ayush%2064.pdf (Assessed on 7 April 2020).
53. Sharma KD, Kapoor ML, Vaidya SP et al. A Clinical Trial of ‘AYUSH 64" (A Coded Antimalarial Medicine) in Cases of Malaria, Jour. Res. Ay. Sid., 1981, 2 (4), 309-326.
54. Anonymous. Management of Chikungunya through Ayurveda and Siddha, A Technical Report, CCRAS, Dept of AYUSH, MoHFW, Govt of India, 2009
55. N Srikanth et al. Effect of ‘AYUSH-64’ in the treatment of Malaria. Seminar for Developing an interaction between CCRAS and Pharmaceutical Industry, CCRAS, Department of AYUSH, Ministry of Health & Family Welfare, Govt. of India New
56. Gundeti M, Bhurke L, Mundada P, Murudkar S, Surve A, Sharma R. AYUSH 64, a polyherbal Ayurvedic formulation in Influenza like Illness: results of a pilot study. J Ayurveda Integr Med. 2020 (In press).
57. Zhao YL et al. Effect of total alkaloids from Alstoniascholaris on airway inflammation in rats., Ethnopharmacol. 2016 Feb 3;178:258-65
58. Verma H, Patil P R, Kolhapure R M, et al. Antiviral activity of the Indian medicinal plant extract, Swertiachirata against herpes simplex viruses: A study by in-vitro and molecular approach. Indian J Med Microbiol 2008;26:322-326
59. So-Yeun Woo, NwetNwet Win, WyineMyat Noe Oo, et al. Viral protein R inhibitors from Swertiachirata of Myanmar. Journal of Bioscience and Bioengineering, 2019, 128 (4), 445-449.
60. Ren JL, Zhang AH, Wang XJ.Traditional Chinese medicine for COVID-19 treatment.Pharmacol Res. 2020;155:104743. doi: 10.1016/j.phrs.2020.104743.
61. TCM Clinical Studies registered in Chinese Clinical Trial Registry, http://www.chictr.org.cn (Data search on 29 March 2020)
62. Yang Liu, Li-Meng Yan, Lagen Wan, Tian-Xin Xiang, Aiping Le, Jia-Ming Liu et al. Viral dynamics in mild and severe cases of COVID-19 Lancet Infect Dis 2020 , March 19, 2020 https://doi.org/10.1016/ S1473-3099(20)30232-2)
63. National Ethical Guidelines For Biomedical And Health Research Involving Human Participants. Available at <https://main.icmr.nic.in/sites/default/files/guidelines/ICMR_Ethical_Guidelines_2017.pdf>). Accessed on 28 April2020
64. [https://admin.ich.org/sites/default/files/inline-files/ICH-E8_GlobalMeeting_SummaryReport_2020 _0220_0.pdf](https://admin.ich.org/sites/default/files/inline-files/ICH-E8_GlobalMeeting_SummaryReport_2020%20_0220_0.pdf)). Accessed on 28 April 2020
65. <https://admin.ich.org/sites/default/files/2019-04/ICH>:_Reflection_paper_GCP_Renovation_Jan_2017 _Final.pdf. Accessed on 28 April 2020
66. General guidelines for clinical evaluation of Ayurvedic interventions. Available at <http://www.ccras.nic.in/sites/default/files/viewpdf/Publication/CCRAS_Guideline%20of%20Clinical_Evaluation.pdf>. Accessed on 29 April 2020
67. https://www.academia.edu /2377559/AYUSH_ GCP_ GUIDELINES. Accessed on 20 April 2020
68. Declaration of Helsinki (Update) <https://www.who.int/bulletin/archives/79(4)373.pdf> Accessed on 05 April 2020
69. Adverse Events and Quantum of Compensation. Available at <https://cdsco.gov.in/opencms/opencms/en/Notifications/documents/>. Accessed on 10 April 2020

# APPENDIX

## Appendix A: Schedule of Events

**Table 1: SCHEDULE OF EVENTS:**

| VISIT | Baseline | D 4 | Day 4 Discharge | Discharge | Week 4 | Week 8 | Week 12 | Remarks |
| --- | --- | --- | --- | --- | --- | --- | --- | --- |
| Informed Consent,  Selection Criteria, Demographics, Disease Features, Baseline Symptoms | X |  |  |  |  |  |  |  |
| Medical History and Comprehensive Physical Examination | X |  |  | X |  | X | X |  |
| Symptom, Vital Parameters | X |  |  |  |  |  |  |  |
| Adverse Events **^a^** |  | X | X | X | X | X | X | X |
| AyurvedicEvaluation**^b^** | X |  | X |  | X | X | X |  |
| Pulse Oximetry**^c^** | X | X | X | X | X | X | X |  |
| USG Abdomen and Pelvis | X |  |  |  |  |  |  |  |
| Color Doppler and 12 Lead ECG | X |  |  | X |  |  |  |  |
| Chest X-ray | X | X | X | X |  |  |  |  |
| HRCT Chest | X | X |  | X |  |  |  |  |
| Pregnancy Test | X |  |  |  |  |  |  |  |

| VISIT | Baseline | Day 4 | Day 4- Discharge | Discharge | Week 4 | Week 8 | Week 12 | Remarks |
| --- | --- | --- | --- | --- | --- | --- | --- | --- |
| Hb, TLC, DLC, Platelet, Urine ®**^d^** | X |  | X |  | X | X | X |  |
| ESR and CRP**^e^** | X |  | X |  | X | X | X |  |
| LFT, BUN, SrCreatinine**^f^** | X |  | X |  | X | X | X |  |
| Lipid Profile | X |  |  |  | X |  | X |  |
| LDH, Ferritin**^g^** | X |  | X |  | X |  | X |  |
| IL-6 Pro-cal**^h^** | X |  | X |  | X |  | X |  |
| CK, BNP, Troponin,  D-Dimer **^i^** | X |  | X |  | X |  | X |  |
| Na, K, Cl, Fe, Zn, Mn, Vitamin D , B12 | X |  |  | X |  |  | X |  |
| Oxidation Biomarkers  -SOD, GSH | X | X |  | X |  |  | X |  |
| Cytokine Panel**^J^** | X |  |  | X |  |  | X |  |
| Serum Immune Response**^k^** | X | X | X | X | X | X | X |  |
| Health Related-Behaviour Fitness (HR-BHF) VAS questionnaire, Self Reported Questionnaire (Mobile app)**^L^** | X | X | X | X | X | X | X |  |
| Dispense study medication**^m^** | X |  |  |  |  |  |  |  |
| Completion report |  |  |  | X |  |  | X |  |

Footnotes:

1. Symptom Assessment, vital parameters and monitoring of adverse events to be performed daily by Physician
2. Daily Ayurvedic Monitoring till discharge by Ayurvedic Physician and subsequently for 4, 8 and 12 week.
3. Pulse oximetry to be performed daily

d-f: These tests should be performed at Baseline and once in 3 days till discharge and in addition as per discretion of the treating physician

g-iThese tests should be performed at Baseline and once in 3 day till discharge, week 4 and week 12 and in addition as per discretion of the treating physician

j Cytokine Panel includes IL-2, Il-4, IL-6, IL-10, TNF-α, IL-1β, IL-13, MCP, Gamma Interferon, to be performed at Baseline, Day 5, Day 10, Day 15

Day 20/Discharge, Week 8, Week 12.

k. Serum Immune Response includes IgG, IgM, and will be performed at Baseline, Day 2, Day 4, Day 6, Discharge, Week 4, Week 8, and Week 12.

L .Health Related -Behaviour Habit Fitness , HR-BHF, WHO-QOL Bref, Mobile Application for recording COVID-19 symptoms daily

m. Study medication will be dispensed every 4 weeks, at baseline, week 4 and week 12

Note:

i)The treating physician can ask for any investigations anytime, if need arises and as per clinical judgement

ii)If the discharge takes place within 2 weeks of a particular timeline (week 4 or week 8) then the events/investigations for that particular timeline will merge with those required at time of discharge (DO NOT repeat again for that timeline)

## Appendix B: Summary of SOLIDARITY Study

WHO SPONSORED INTERNATIONAL DRUG TRIAL ON

MEDICAL MANAGEMENT OF COVID 19 (SOLIDARITY STUDY)- SUMMARY

(<https://www.who.int/emergencies/diseases/novel-coronavirus-2019/global-research-on-novel->

coronavirus-2019-ncov/solidarity-clinical-trial-for-covid-19-treatments)

There are no specific anti-viral drugs available to treat COVID-19. However, there are several drugs being used clinically as unproven therapy in an emergency situation but with some rationale and/ or insufficient evidence. The ’SOLIDARITY STUDY’ trial will evaluate additional treatments for hospitalized patients suffering from COVID 19 being managed with the local Standard of Care (SOC). The patients will be enrolled in a randomized, parallel efficacy, open label, active control multi-centric study. All study procedures including enrollment and assessment will use a web based program. The study sites can recruit any number of patients depending upon the intensity of the epidemic.The study interventional experimental drugsare four anti-viral agents-Remdesivir, Chloroquine/Hydroxychloroquine, Lopinavir (plus Ritonavir) andInterferon (β1a). Study patients will be randomized depending upon the number of study interventional drugs available at any particular site. The study interventional drug will be added to SOC in interventional arms and will be compared to a study arm wherein patients are managed by ‘standalone’ SOC; comparative study design.The study design allows optimum flexibility and discretion to the participating countries and investigators so to adapt and cater to local situation and requirements.

The primary objective is to provide reliable estimates on any effects of these anti-viral

treatments on in-hospital mortality in moderate and severe cases of COVID-19.

The secondary objectives are to assess any effects of these anti-viral treatments on hospital duration and receipt of ventilation or intensive care, and to identify any serious adverse reactions.

Eligibility: consenting adults (age ≥18) who are hospitalized with definite COVID-19 (No testing method mentioned) and are already receiving any of the study drugs, without any known allergy or contra-indications to any of them (in the view of the physician responsible for their care),and without anticipated transfer within 72 hours to a non-study hospital. A patient will not eligible for the trial if it is believed have a significant contra-indication to any one of the study drugs (e.g., serious chronic liver or heart disease or pregnancy) by the treating physician.

The dosage schedule of the intervention drugs is- (i) Remdesivir: daily infusion for 10 days) (ii) Chloroquine or Hydroxychloroquine (155 mg base tablet): 4 tab start and repeat after 6 hours, and maintain thereafter with 2 tablets administered every 12 hours to complete a total of 20 doses (iii) Lopinavir with Ritonavir: Orally twice daily for 14 days (iv) Lopinavir with Ritonavir (as in iii above) plus Interferon (Daily injection for 6 days).

All serious adverse and unexpected reactions must be reported through the study website within 24 hours of occurrence. A possible major protocol deviation would be substantial over-dosing (study drug) and this will need to be reported within 24 hours.

Patients are free to withdraw from study treatment at any time, but could still remain in the study; in this case it would be important to report discharge from hospital or death (while on treatment for COVID 19). All patients withdrawing from the study be offered the local standard of care (but not be reported on).

Analysis will relate to the outcome of a randomly allocated treatment (i.e. intent-to-treat). The primary analyses will assess any effects of treatment allocation on the all-cause in-hospital mortality; individualizes as per severity of disease,

At all times the patient’s medical team remains will be solely responsible for decisions about the care and safety of the study patient. Hence, the medical team is fully authorised to take all decisions concerning the study drugs or any other aspect of the drug trial.

Data monitoring: A global Data and Safety Monitoring Committee will keep the accumulating drug safety results and major outcome results under regular review.

Adaptive design: The WHO may decide to add novel treatment arms while the trial is in progress. Conversely, the WHO may decide to discontinue some treatment arms based on the interim analysis report of the ‘Global Data and Safety Monitoring Committee report’ with special regard to mortality.

Participating countries or groups of hospitals may want to collaborate in making further measurements or observations (virology and other biomarkers assay, serial lung imaging, documentation of other disease aspects). Though the latter may be valuable, it is not the core aspect of the ‘Solidarity Study’.

The Co-Sponsors of this study are the National Ministry of Health in each participating country and WHO. The study drugs will be available at no cost from the study Sponsors, but the study does not cover any other aspect of patient care.

WHO has established a global clinical trial liability insurance (for individuals suffering serious adverse reactions arising from the use of the investigational therapeutics for COVID-19 as part of the Solidarity Trial) that will cover all countries that participate in the Trial. Coverage is provided for serious adverse reactions following the use of an unlicensed therapeutic in all countries, except the OECD, EFTA (Norway, Switzerland, Iceland and Liechtenstein) and the European Union. The territorial scope of the policy (for the filing of claims) is worldwide. Compensation covered by the insurance would be paid directly to the individuals concerned.

Study Outcome Interpretation: The evidence on mortality must be strong enough to influence national international strategy on management of COVID- 19.

## Appendix C: Sanshamani Vati Plus: Ingredients and Related Research

**Background**

Although the rationale is included in the protocol document, a detailed information about research on Ayurveda formulations are discussed in following sections.

The Medanta Hospital in Delhi has implemented a traditional formulation of Ashtang Sangraha (SanshamaniVati Plus) in a state-of-the-art super-specialty hospital. The SanshamaniVati is dried aqueous extract of *Tinosporacordifoila* (TC) indicated in VKJ and immunocompromised conditions. Its immune modulating function has been effectively used HIV and chikungunya (1,2). The advisory by the AYUSH Ministry for COVID-19 has also recommended *SanshamaniVati* [equivalent of 200 ml decoction of *Tinosporacordifolia* and 4 grams *Piper longum* powder / day orally] for improving immunity (3). SamshamaniVati Plus is a formulation made up of aqueous extract of *Tinosporacordifolia* (TC) and fine powder of *Piper longum* (PL). This combination is specific to VKSJ and is mentioned in Ashtanga Hrudaya. This has been selected as therapeutic intervention considering its specificity to the clinical picture of COVID-19, the known immune-enhancing effect of TC and effects of PL in respiratory system. *SamshamaniVati* will be given as 500 mg. thrice daily with warm water for 2-3 weeks.

***Tinospora cordifolia***:

*Tinosporacordifolia* (Menispermaceae) is an Ayurvedic medicinal plant, commonly known as Guduchi or Amrita, is distributed throughout the Indian subcontinent and some parts of China. It is categorized as ‘Rasayana’ in Ayurveda and is used as a tonic and to treat diabetes, skin, heart diseases, jaundice, rheumatoid arthritis, allergies, etc.

It is a rich source of alkaloids, furano diterpenoids, clerodane norditerpenoids, sesquiterpenoids, phenolics, lignans, sterols, aliphatic compounds, polysaccharides, essential oil and fatty acids. The alkaloids (e.g. berberine), bitter compounds (tinosporin, tinosporic acid and tinosporol) and lipids have been found to exhibit medicinal effects (4,5).

A diterpenoid, tinosporin, has shown activity against HIV, HTLV and other viral diseases (6).The plant has been studied extensively for its immunomodulating activities. The active principles of *Tinosporacordifolia* were found to cause significant increase in IgG antibodies along with macrophage activation in guinea pigs (7). The plant has been proposed to possess immense potential for use against novel H1N1 flu since it is potent immunostimulant (8). A study on *Tinosporacordifolia* and CpG ODN (TLR21 Agonist) against the very virulent, infectious Bursal Disease Virus in SPF Chicks reveals that both these drugs can be used as prophylactic agents and as adjuvants in poultry for viral diseases. Both the drugs demonstrated immunomodulatory potential via the TLR mediated pathway (9). Antiviral prospective of *Tinosporacordifolia* on HSV-1 was studied using vero cell line. Viral titration was carried out followed by virucidal assay and it was concluded that *T. cordifolia* inhibits the growth of HSV by 61.43% at 10TCID_50_ (10).A study conducted to explore anti-viral activity of the plant against Hepatitis-A virus concluded that the ethanolic extract of the whole plant showed a dose dependent anti-HAV activity and it was maximum at 50 μg/mL with 56.24% of virus viability and it was comparable to standard drug camptothecin where it shows 43.01% of viability (11).

In yet another study to determine the activity of aqueous and methanolic extracts of *Tinosporacordifolia* against Salmonella typhimurium. The methanolic extract treatment resulted in increased survival and reduced bacterial load in S. typhimurium-infected mice (12).

The *Tinosporacordifolia*extract (TCE) when tested in Human Immuno-Deficiency Virus Positive Patients significantly affected the symptoms of HIV. TCE treatment caused significant reduction in eosinophil count and hemoglobin percentage. 60% patients receiving TCE and 20% on placebo reported decrease in the incidence of various symptoms associated with disease (13).

A review on herbal plants as immunomodulators mentioned presence of Immunomodulatory Protein (ImP) is present in Tinospora. It was purified from dry stem powder extract and was shown to possess lymphoproliferative and macrophageactivating properties (14). Another review carried out to explore the antioxidant, immunomodulatory and anti-cancer potential of Tinospora. The review states that the stem, root and bark parts of the plant have many active principles mainly in alcoholic and aqueous extracts that have shown prominent antioxidant, immunomodulatory and anticancer activities. The most active principles viz. berberine, palmetin, G1-4A, α-D glucan, clerodane furane diterpene glycoside, epoxy clerodane diterpene and immunomodulatory protein, are reported to be effective against different diseases in either individual or synergistic manner (15).

*T. cordifolia*also has potential to enhance the phagocytosis and pinocytosis by macrophages (16). The aqueous extract of *Tinosporacordifolia* when administered for 10 days at a dose of 100 mg/kg orally leads to significant increase in CFU-GM (Colony Forming Units of the granulocyte-macrophage series) activity in the serum of mice. This suggests that activation of macrophages by *Tinosporacordifolia* leads to increase in GM-CSF which leads to leucocytosis and improved neutrophil function (17). In another study it was found that pre-treatment with Tinosporacordifolias lead to protection against mortality induced by intra-abdominal sepsis following caecal ligation in rats. It was reported that treatment in rats had resulted in significant leucocytosis and predominant neutropenia. It has been also observed that it stimulated the macrophages as evidenced by an increase in the number and percentage phagocytosis of S. aureaus by peritoneal macrophages in rats. Syringin, Cordiol, Cordioside, Cordifoliosides A&B were identified as the active principle responsible for the anti-compliment and immunomodulatory activities (18).

***Piper longum*:**

*P. longum*(Piperaceae), an indigenously growing plant is an important component of Ayurvedic medicine. Traditionally, it is used for treating respiratory tract infections, chronic gut related pain, gonorrhea, menstrual pain, tuberculosis, and arthritic conditions (19).There is a large phytochemical compound base which includes phenanthrenes, phenylpropanoic acids, unsaturated hydrocarbons, diarylheptanoids, piperidines, oxanes, organooxygen compounds, aristolactams, furanoid lignans, cinnamic acids, pteridines, phenol ethers, alkaloids, carboxylic acids, saturated hydrocarbons, pyridines, organic nitrogen compounds, polycyclic hydrocarbons, naphthalenes, isoflavonoids, benzodioxoles, prenol lipids, fatty acyls, steroids, phenols and benzene derivatives. The pharmacological properties of this plant include anticancer, antioxidant, anti-inflammatory, hepatoprotective, immunomodulatory, antimicrobial, anti-platelet, anti-hyperlipidemic, analgesic, antidepressant, anti-amoebic, anti-obesitic, radio-protective, cardio-protective and anti-fungal activities (20).

Coronavirus disease 2019 (COVID-19) is characterised by an over exuberant inflammatory response (21). When COVI-19 infects the upper and lower respiratory tract it can cause mild or highly acute respiratory syndrome with consequent release of pro-inflammatory cytokines, including interleukin (IL)-1β and IL-6 (22).It has been established that cell adhesion molecules are the key players in this process. The migration of the leukocytes to the site of inflammation is regulated partly by the expression of cell adhesion molecules, viz. intercellular adhesion molecule-1 (ICAM-1), vascular cell adhesion molecule-1 (VCAM-1) and E-selectin. Chloroform extract of *P. longum* has been proved to exhibit 70 % inhibition of TNF-α induced ICAM-1 and VCAM-1 expression on human umbilical vein endothelial cells (HUVECs), at 17.5 μg/ml concentration and E-selectin at 15 μg/ml followed by hexane and ethanol extracts, which showed around 40 % inhibition (23).Piperine, a phytoconstituent of *P.longum*, has been also reported to inhibit the TNF-α induced expression of cell adhesion molecules by inhibiting nuclear factor-κB (NF-κB) activation in endothelial cells (24, 25, 26). Piperine also acts as a powerful superoxide scavenger with IC50 of 1.82 mM and a 52 % inhibition of lipid peroxidation (27).

*Piper longum* and piperine, both also display efficient immunomodulatory and anti tumor activity. Alcoholic extract of the Piper fruits was 100% toxic at a concentration of 500 microg/ml to Dalton's lymphoma ascites (DLA) cells and 250 microg/ml to Ehrlich ascites carcinoma (EAC) cells. Piperine was found to be cytotoxic towards DLA and EAC cells at a concentration of 250 microg/ml. Administration of *Piper longum* extract and piperine increased the total WBC count to 142.8 and 138.9%, respectively, in Balb/c mice. The number of plaque-forming cells also enhanced significantly by the administration of the extract (100.3%) and piperine (71.4%) on 5^th^ day after immunization (28).

Ethanol extract of Piper has displayed anti-HBV activity on Hep G2 cell line. Piperine was also found to have remarkable inhibitory HBV activity, against the secretion of Hepatitis B virus surface antigen (HBsAg) and Hepatitis B virus e antigen (HBeAg) with the Selectivity Index (SI) value of 15.7 and 16.8 respectively (29).

In C57BL/6 mice injected with B16F-10 melanoma cells, intraperitoneal administration of methanolic extract of Piper (10 mg/dose/animal) significantly inhibited (50.6%) the number of tumor-directed capillaries induced by injected cells. Further, the extract could differentially regulate level of the cytokines (30).

**References:**

Kalikar MV, Thawani VR, Varadpande UK, Sontakke SD, Singh RP, Khiyani RK. Immunomodulatory effect of Tinosporacordifolia extract in human immuno-deficiency virus positive patients. Indian J Pharmacol. 2008;40(3):107-10.

Sharma V, Kaushik S, Pandit P, Dhull D, Yadav JP, Kaushik S. Green synthesis of silver nanoparticles from medicinal plants and evaluation of their antiviral potential against chikungunya virus. ApplMicrobiolBiotechnol. 2019;103(2):881-891.

Advisory published by Ministry of AYUSH, D.O. No. S. 16030/18/2019 – NAM, dated 6 March 2020.

Chadha YR. (1948) The Wealth of India. Publication and Information Directorate, CSIR, New Delhi, 33

Singh D, Chaudhari PK. Chemistry and Pharmacology of Tinosporacordifolia. Nat Prod Communication 2017;12(2):299-308.

Chetan B, Nakum A. Use of natural compounds, chitin and tinosporin for the treatment of the targeted viruses (retroviruses) (HIV-1, HIV-2) all subgroups, HTLV and other viral disease. Indian patent App 2010; IN 2010MU01350 A 20100730.

Fortunatov MN. Experimental use of phytoncides for therapeutic and prophylactic purpose. VoprosyPediatrii I OkhranyMaterinstva 1952;20(2):55–58.

Arora R, Chawla R, Marwah R et al. Potential of complementary and alternative medicine in preventive management of Novel H1N1 flu Pandemic: Thwarting potential disasters in the bud. Evidence based Complementary and Alternative Medicine 2011; article id 586506:1-16

Sachan Swati, Dhama K et al. Immunomodulatory Potential of Tinosporacordifolia and CpG ODN (TLR21 Agonist) against the Very Virulent, Infectious Bursal Disease Virus in SPF Chicks. Vaccines 2019;7(106):1-22

Pruthivish R, Gopinath SM. Antiviral prospective of Tinosporacordifolia on HSV-1. Int. J. Curr. Microbiol. App. Sci 2018;7(1): 3617-3624

Maddi R, Kandula VL, Vallepu B, Navuluri H, Kolluri H, Vunnam DT. Preliminary Phytochemical Analysis and Invitro Anti–viral Activity of Ethanolic extract of Whole plant of Tinosporacordifolia (Thunb.) Miers against Hepatitis-A Virus. Int. J. Sci. Res. in Biological Sciences 2018;5(3):51-55.

Sultan Alsuhaibani, Masood A. Khan. Immune-Stimulatory and Therapeutic Activity of Tinosporacordifolia: Double-Edged Sword against Salmonellosis. Journal of Immunology Research 2017; Article ID 1787803:1-9

KalikarMV,ThawaniVR,Varadpande UK et al. Immunomodulatory effect of Tinosporacordifolia extract in human immuno-deficiency virus positive patients. Indian Journal of Pharmacology 2008; 40(3):107-110

Singh N, Tailang M, Mehta SC. A review on herbal plants as Immunomodulators. IJPSR 2016;7(9): 3602-3610.

Verma R, Khan AB. Antioxidant, Immunomodulatory and Anticancer potential of TinosporaCordifolia - A Review. Int J Pharm Biol Sci.2018; 8(3):54-59.

More P, Pai K. Effect of Tinosporacordifolia (Guduchi) on the phagocytic and pinocytic activity of murine macrophages in vitro. Indian J Exp Biol 2017; 55:21-26.

Thatte UM, Rao SG, Dahanukar SA. Tinosporacordifolia induces colony stimulating activity in serum. J Postgrad Med 1994;40(4):202-203.

Thatte UM, Dahanukar SA. Immunotherapeutic modification of experimental infections. Ind Med plants Phytoter Res 1989; 3:43-49.

Sarvesh Kumar, Shashwat Malhotra, Ashok K et al. Anti-Inflammatory and Antioxidant Properties of Piper Species: A Perspective from Screening to Molecular Mechanisms. Curr Top Med Chem. 2015; 15(9): 886–893

Chowdhary N, Singh V, Piper longum: A review of its phytochemicals and their network pharmacological evaluation. PLOS One 2017

Stebbing J, Phelan A, Griffin I, etal. Covid- 19: combining antiviral and anti- inflammatory treatments. The Lancet Infect Dis 2020;20(4):400-402

Conti P, Ronconi G, Caraffa A, et al. Induction of Pro-Inflammatory Cytokines (IL-1 and IL-6) and Lung Inflammation by Coronavirus-19 (COVI-19 or SARS-CoV-2): Anti-Inflammatory Strategies. J BiolRegulHomeost Agents 2020 ;34(2):1

Singh N, Kumar S, Singh P, etal. Piper longum Linn. Extract inhibits TNF-alpha-induced expression of cell adhesion molecules by inhibiting NFkappaB activation and microsomal lipid peroxidation. Phytomedicine, 2008 15(4): 284–91

Kumar S; Singhal V; Roshan R; Sharma A; Rembhotkar GW; Ghosh B Piperine inhibits TNF alpha induced adhesion of neutrophils to endothelial monolayer through suppression of NF kappa B and I kappa B kinase activation. Eur. J. Pharmacol, 2007; 575(1–3): 177–86

Reddy AC and Lokesh BR, Studies on spice principles as antioxidants in the inhibition of lipid peroxidation of rat liver microsomes. Mol. Cell Biochem, 1992; 111(1–2): 117–24

Ku SK, Kim JA, and Bae JS, Piper longuminine downregulates endothelial protein C receptor shedding in vitro and in vivo. Inflammation, 2014 37(2): 435–42

Mittal R and Gupta RL, In vitro antioxidant activity of piperine. Methods Find Exp. Clin. Pharmacol, 2000; 22(5): 271–4

Sunila ES, Kuttan G. Immunomodulatory and antitumor activity of Piper longum Linn. and piperine. J Ethnopharmacol. 2004 ;90(2-3): 339-46

Jiang ZY, Liu WF, Zhang XM, et al. Anti-HBV active constituents from Piper longum. Bioorg Med Chem Lett. 2013;23(7):2123-7

Sunila ES, Kuttan G. Piper longum inhibits VEGF and proinflammatory cytokines and tumor-induced angiogenesis in C57BL/6 mice. International Immunopharmacology 2006;6(5):733-741

## Appendix D: AYUSH 64: Ingredients and Related Research

**Background**

Central Council for Research in Ayurvedic Sciences, an apex body for research and development in Ayurveda in India under the Ministry of AYUSH has developed a poly-herbal drug ‘AYUSH 64’ through extensive pharmacological, toxicological /safety and clinical studies.

 ‘AYUSH 64’ consists of- *Saptaparna* (*Alstoniascholaris* R. Br.) Bark Aqueous Extract 100 mg.,*Katuki* (*Picrorhizakurroa*Royle ex. Benth) Root Extract 100 mg., *Kiratatikta*(*SwertiaChirata*Pexbex. Karst) Whole-plant Extract 100 mg.,*Kuberaksha*(*Caesalpinia crista* L.) Seed powder 200 mg.

**Ayurveda Pharmacological profile of the plants in AYUSH 64**

| Name of the plant | *Rasa* | *Guna* | *Virya* | *Vipaaka* | *Karma* |
| --- | --- | --- | --- | --- | --- |
| *Saptaparna*  *(Alstoniascholaris*R. Br.)  (1,2,3,4) | *Tikta, Kashaya* | *Sara, Ushna, Snigdha, Deepan, Laghu* | *Ushna* | *Katu* | *Shoolahara* (relieves pain)*, Gulmahara*(relieves bloating)*, Krimihara*(wormicidal)*, Hrudya* (cardiac tonic)*, Shwashara* (userful in asthma)*, Vranahara*(wound healer)*, Asradoshahara*(useful in blood related diseases)*, Jantuhara*(antihelminthic)*, Tridoshaghna*(pacifies *kapha, pitta* and *vata*), *Kasahara* (useful Bronchitis)*Kushthaghna*(useful in skin disorders), *Jvaraghna* (Antipyretic), *SaraktaPravahikahar* (useful in bloody dysentery), *Vataraktahara* (*useful in Gout*), *Grahanihara*(useful in IBS like conditions)*Udardaprashaman*(relieves urticaria), *Dantakrimihara* (useful in dental caries),*Vishaghna*(Antitoxic) |
| Katuki  (*Picrorhizakurroa*Royle ex. Benth) (5) | *Tikta* | *Laghu, Ruksha* | *Shita* | *Katu* | *Bhedana* (causes purgation)*, Deepana* (improves digestion)*, Hrudya*(cardiac tonic)*, Jwarahara*(useful in fevers)*, Vishamjvaranashini*( useful in recurrent fevers)*Kaphapittahara*(pacifies *Kapha*and *pitta), Pramehaghna*(useful in urinary disorders/ diabetes)*, Shwasa-Kaasaghna* (useful in asthma, cough)*, Dahaghna*(relieves burning sensation)*, Kushthaghna* (userful in skin disorders), *Lekhana*(therapeutic scrapping),*Krimihara*(wormicidal), *Arochakaghna* (useful in tastelessness)*Asrajit*(useful in blood disorders)*, Stanyashodhan*(improves quality of breast milk) *Hikkanigrahana* (relieves hiccups) |
| *Kiratatikta*  *(SwertiaChirata*Pexbex. Karst) (6) | *Tikta* | *Laghu, Ruksha* | *Shita* | *Katu* | *Sannipatajwarahara*(useful in chronic and recurrent fevers)*, Shwasahara*(userful in asthma)*, Kaphapittahara*(pacifies *Kapha*and *pitta), Asradoshahara*(useful in blood related diseases)*, Dahashamana*(relieves burning sensation)*, Kasahara* (useful in cough)*, Shothahara*(relieves inflammation)*, Trishnashamaka*(relieves thirst)*, Kushthahara (*useful in skin disorders)*, Vranahara*(wound healer)*, Krimihara*(wormicidal), *Stanyashodhan*(improves quality of breast milk), *Jvaraghna* (Antipyretic), *Raktapittahara* (useful in bleeding disorders) |
| *Kuberaksha* (*Caesalpinia crista* L.) (6,7) | *Katu, Tikta, Kashaya* | *LaghuRuksha* | *Ushna* | *Katu* | *Kaphavatahara*(pacifies *Kapha*and *vata), Deepana*(improves digestion)*, Shoolaghna*(relieves pain)*, Gulmanaashaka*(relieves bloating)*, Kriminashaka*(wormicidal)*, Kushthanaashaka (*useful in skin disorders)*, Pramehajit*(useful in urinary disorders/ diabetes)*, Pittarshanaashaka*(useful in haemorrhoids)*, Vamihara*(antiemetic), *Sramsana*(laxative),*Shothahara*(anti-inflammatory),*Vranaropana*(wound healer)*,Yakrutplihaghni*(useful in Liver & spleen related disorders), *Vataghna*(pacifies vata), *KaphajaShlipadahara*(useful in filariasis) |

All the four herbs are having *Tikta rasa* which is *Amapachak*(digest the *Ama* i.e. undigested form of food) and hence *Jvaraghna*. The combination effect of these herbs is *Jwarahara*(relieves fever)*, Sannipatajwarahara* (relieves recurrent fevers), *Krimihara*(wormicidal),*Jantuhara*(antihelminthic),and*Shothahara*(anti-inflammatory)making this as potent combination against condition like ILI having the symptoms of cough, cold, headache, fever etc.

**Preclinical pharmacological and toxicological /safety studies AYUSH 64 and its ingredients**

**I.AYUSH 64 FORMULATION**

1. In albino mice, oral administration of Ayush -64 at doses of 250-750mg/kg for five days exhibited significant anti-malarial property. (8)
2. The experimental studies of AYUSH 64 have shown that it was safe and non-toxic in a dose of 500 mg/kg of body weight for 12 weeks. (9, 10)
3. Antiviral activity of Chirakin (marketed name of AYUSH 64 by Zandu) against Chikungunya virus was investigated at department of Molecular Virology Laboratory, Rajiv Gandhi Centre for Biotechnology, Thiruvananthapuram, by Protection and Plaque reduction assay. The efficacy of the compound was expressed in terms of Activity index and Selectivity index in protection assay and Vero cells were infected with CHIKV (MOI 0.1) and treated with an optimum concentration of Chirakin (25μg/ml). Chirakin treated cells and the reduction in virus yield was determined by plaque assay in Verocells. In this study Chirakin shows antiviral activity against Chikungunya virus. Its activity is better than Ribavirin in protection assays, but in plaque reduction assays, both perform almost equally with a 2 log10 reduction in virus numbers. (11)

**II.STUDIES ON INGREDIENTS**

***Kiratatikta*(*Swertiachirata Buch-ham)***

1. Inhibits the expression of Viral protein R (an attractive target for HIV disease) in Hela cells harbouring the TREx plasmid encoding full-length Vpr (TREx-HeLa-Vpr cells). (12)
2. Inhibited HSV-1, plaque formation at more than 70% level and viral dissemination (13)
3. Suppressive effects on inflammatory mediators by blocking the expression of COX-2 and phosphorylation of Akt, IKK-β, MAPK and NF-κB, activation in LPS-stimulated macrophages (14)
4. a study on experimental arthritis in rats suggested amelioration of oxidative and inflammatory stress thus once again implying immunomodulatory effect of the leaves of the said plant. Post treatment with the leaves of the said plant the animal patients showed marked reduction in inflammation as well as arthritic changes. (15)
5. Have antipyretic (16), antimalarial (17) & Hepatoprotective properties. (18)

***Saptapama*(A*lstoniascholaris R.Br* - Stem Bark)**

1. Inhibited the carrageenan-induced inflammation in the rat paw oedema study model (19)
2. Controls Malarial fever by virtue of its strong schizonticidal activity (20)
3. Anti- HSW and anti-adenovirus activity of indole alkaloids from leaves of *Alstoniascholaris*, with significant inhibitory activity against herpes simplex virus (HSV) and adenovirus (21)
4. Enhance DNA repair capacity. (22)
5. in vitrotests, alkaloidsexhibited inhibition of inflammatorymediators (COX-1, COX-2 and 5-LOX), which is accordant with results on animal model. (23)
6. Potent antiplasmodial activity against P. falciparum (24, 25)

***Katuki (Picrorhiza kurroa RoyluxBenth– Root)***

1. Anti-inflammatory effect by l3-adrenergic blockade (26)
2. Stimulate the cell-mediated and humoral components of the immune system as well as phagocytosis in experimental animals (27, 28)
3. Improves the immune system by increasing the proliferation of lymphocytes and cytokine levels (IL-4 and IFN-gamma) in serum, in HA titre, DTH, PFC, phagocytic index and CD4/CD8 population. (29)
4. Another study demonstrates the antioxidant and free radical scavenging activity of the leaf extract of the same plant. (30)
5. Significantly inhibit growth of Plasmodium falciparum (31, 32)

***(KuberakshaCaesalpinia crista Linn. – Seed)***

1. Protection against red blood cell (RBC) haemolysis and DNA damage (33)
2. Immuno-stimulatory: increase in hemagglutinating antibody titre and a change in delayed-type hypersensitivity (34)
3. Two weeks after challenge with Pseudomonas aeruginosa, the *Caesalpinia* treated animals showed a significant bacterial clearance from the lungs, with less severe incidence of lung abscess (35)
4. Exhibited activity against the vaccinia virus (36)
5. In an in vivo experimental study, Neutrophil adhesion test, hemagglutinating antibody (HA) titre, delayed-type hypersensitivity (DTH) response, phagocytic activity and cyclophosphamide-induced myelosuppression were demonstrated to be positively activated pointing towards a promise in immunomodulation (37)
6. Possesses Antimalarial (38) & Hepatoprotective (39) activity.

**CLINICAL STUDIES OF AYUSH 64**

1. The report on management of Chikungunya through Ayurveda and Siddha has documented the efficacy of ‘AYUSH-64’. (Anonymous. Management of Chikungunya through Ayurveda and Siddha, A Technical Report, CCRAS, Dept of AYUSH, MoHF&W, Govt of India, 2009)
2. Clinically effective in cases of Microfilariasis. (40)
3. 81% curative effect on Plasmodium vivax, drug is more effective in ring stage than in Gamete stage. In the cases of mixed infection of P. vivax & P. falciparrum the curative effect was found to be 75% after longer therapy. (41)
4. Clinically shown good effect in the management of microfilaraemia (42, 43)
5. In a study with about 4500 participants, Ayush-64 was found safe and nontoxic with good antimalarial activity and also observed that by giving Ayush-64 to all fevers along with anti- mosquito measures, there was a decline in the infectivity rate. (44)
6. A double-blind study demonstrated comparative efficacy of AYUSH 64 to the chloroquine /primaquine as the standard modern control in sixty cases of P. Vivax malaria. (45)
7. In a prospective, open-label, nonrandomized, single group, single-centre pilot study with pre-test and post-test design one-week intervention of ‘AYUSH 64’ in a dose of 3gms/day effectively helped to recover from Influenza Like Illness  symptoms and return to normal life early with reduced frequency of usage of acetaminophen/ antihistaminic. No adverse effects were found during the study (46).
8. A pilot study done on 112 participants produced 97.14% result with prophylactic treatment in P.F. Malaria (47)

**Note: The drug /drug and drug herb interactions may not be so relevant as the formulation is not NCE /and possess herbs processed as per Standard procedures of Ayurveda, however such studies may be taken up simultaneously**

**Thus AYUSH 64 may prove to be a promising solution in combating COVID-19 in India.**

**References:**

1. *Dhanwantari Nighantu* – *ChaaaandanaadiVarga* 3/86,87
2. *Kaiyadeva Nighantu – AushadhiVarga* 1/953,954,955
3. *Madanpala Nighantu – VatadiVarga* 5/57

*Bhavaprakash Nighantu Poorvakhanda, Mishra prakaran, VataadiVarga* 6/61

*Bhavaprakash Nighantu Poorvakhanda, Mishra prakaran, HaritakyaadiVarga* 2/134, 135

Bhavaprakash Nighantu Poorvakhanda, Mishra prakaran, GuduchyadiVarga 4/107, 108

*RajnighantuShalmalyaadiVarga* 8/62, 63

Pharmacological Investigations of certain Medicinal Plants and Compound Formulations used in Ayurveda and Siddha; Central Council for Research in Ayurveda & Siddha (CCRAS), 1996

1. Ayush-64 A New Ayurvedic Anti- Malarial Compound, Central Council for Research in Ayurveda&Siddha (CCRAS) 1987

Pharmacological Investigations of certain Medicinal Plants and Compound Formulations used in Ayurveda and Siddha; Central Council for Research in Ayurveda & Siddha (CCRAS), 1996

CK Katiyar. Technical note on Chirakin Tab

So-Yeun Woo, NwetNwet Win, WyineMyat Noe Oo, Hla Ngwe, Takuya Ito, Ikuro Abe, and Hiroyuki Morita. Viral protein R inhibitors from Swertiachirata of Myanmar. J. Biosci. Bioeng. 2019; 128(4):445-49, https://doi.org/10.1016/j.jbiosc.2019.04.006

Verma H, Patil P R, Kolhapure R M, et al. Antiviral activity of the Indian medicinal plant extract, Swertiachirata against herpes simplex viruses: A study by in-vitro and molecular approach. Indian J Med Microbiol 2008; 26:322-326

Hu Tian-Yong et.al. Anti-inflammation action of xanthones from Swertiachirayita by regulating COX-2/NF-κB/MAPKs/Akt signaling pathways in RAW 264.7 macrophage cells. Phytomedicine 55 (2019) 214–221.

1. Lad H, Bhatnagar D. Amelioration of oxidative and inflammatory changes by Swertiachirayita leaves in experimental arthritis. Inflammopharmacology. 2016 Dec;24(6):363-375. Epub 2016 Oct 14.
2. S. Bhargava et al, Sci Pharm. Antipyretic Potential of Swertiachirata Buch Ham. Root Extract2009; 77; 617–623.
3. G. Praveen Bhat et.al., in vitro antimalarial activity of extracts of three plants used in the traditional medicine of india, Am. J. Trop. Med. Hyg., 65(4), 2001, pp. 304–308

R. Nagalekshmi et al. Hepatoprotective activity of Andrographis Paniculata and SwertiaChirayita Food and Chemical Toxicology 49 (2011) 3367–3373

ManjeshwarShrinathBaliga. Review of the Phytochemical, Pharmacological and Toxicological Properties of AlstoniaScholaris Linn. R. Br (Saptaparna). Chin J Integr Med. 2011. DOI: 10.1007/s11655-011-0947-0

Gami B, ParabiaF:, Evaluation of acetone extract of three Indian medicinal plants for schizoticidal properties in Plasmodium falciparum. International Journal of Pharmacy & Technology 2011; 3: 1373-1384.

Zhang L , Zhang CJ , Zhang DB , Wen J, Zhao XW , Li Y, Gao K: An unusual indole alkaloid with anti-adenovirus and anti-HSV activities from Alstoniascholaris. Tetrahedron Letters 2014; 55: 1815–1817.

Dhruti Mistry &MeonisPithawala (2017): Protective effect of Alstoniascholaris Linn. R. Br. Against Bleomycin induced chromosomal damage in cultured human lymphocytes, in vitro, Drug and Chemical Toxicology, DOI: 10.1080/01480545.2017.1329316

1. Jian-Hua Shang, Xiang-Hai Cai, TaoFeng, Yun-Li Zhao, Jing-Kun Wang, Lu-Yong Zhang et al.Pharmacological evaluation of Alstoniascholaris: Anti-inflammatory and analgesic effects. J Ethnopharmacol 2010; 129(2):174-8

Ravikumar et.al/In vitro antiplasmodial activity of Kani herb Alstoniascholaris against Plasmodium falciparum, Innovative Journal of Medical and Health Science 5: 4 July - August (2015) 166 – 169.

1. Manoj Gandhi et.al., Preliminary evaluation of extracts of alstoniascholaris bark for in vivo antimalarial activity in mice, Journal of Ethnopharmacology, 29 (1990) 51- 5’7

Pandey BL & Das PK (1989). Immunopharmacological Studies on PicrorhizakurroaRoyle ex Benth. Part IV: Cellular Mechanisms of Anti-Inflammatory Action. Indian J PhysiolPharmacol 33(1): 28-30.

M.L.Sharma, C.S.Rao, P.L.Duda. Immunostimulatory activity of Picrorhizakurroa leaf extract. Journal of Ethnopharmacology. 1994;41(3): 185-192. https://doi.org/10.1016/0378-8741(94)90031-0

Hussain A, Shadma W, Maksood A, Ansari SH. Protective effects of Picrorhizakurroa on cyclophosphamide-induced immunosuppression in mice. Pharmacognosy Res. 2013 Jan;5(1):30-5. doi: 10.4103/0974-8490.105646. PMID: 23598922; PMCID: PMC3579017.

Amit Gupta at.al. Immunomodulatory activity of biopolymeric fraction RLJ-NE-205 from Picrorhizakurroa. International Immunopharmacology, 2006; 6(10):1543-9. DOI: 10.1016/j.intimp.2006.05.002

1. Kant K, Walia M, Agnihotri VK, Pathania V, Singh B. Evaluation of Antioxidant Activity of Picrorhizakurroa (Leaves) Extracts. Indian J Pharm Sci. 2013 May;75(3):324-9. doi: 10.4103/0250-474X.117438. PMID: 24082348; PMCID: PMC3783750.

Abdul Mannan et.al.,Antimalarial activity of three Pakistani medicinal plants, Pak. J. Pharm. Sci., Vol.24, No.4, October 2011, pp.589-591

H. S. Banyal, R. Devi, N. Devi, Picrorhizakurrooa Royal Ex Benth exhibits antimalarial activity against Plasmodium bergheiVincke and Lips, 1948 Asian Journal of Biological Sciences, 7 (2014), 72-75.

Kumar RS, Narasingappa RB, Joshi CG, Girish TK, Danagoudar A. Caesalpinia Crista Linn. Induces protection against DNA and membrane damage. Phcog Mag 2017;13: S250-7.

Shukla Shruti. In Vivo Immunomodulatory Activities of Aqueous Extract of CaesalpiniaBonducella Seed, Pharmaceutical Biology (Formerly International Journal of Pharmacognosy). 2010;48(2):227-230

Arif T et. Al. In vitro and in vivo antimicrobial activities of seeds of Caesalpiniabonduc (Lin.) Roxb. J Ethnopharmacol. 2009 May 4;123(1):177-80. doi: 10.1016/j.jep.2009.02.040. Epub 2009 Mar 9.

Dhar ML, Dhar MM, Dhawan BN, Mehrotra BN, Ray C. Screening of Indian plants for biological activity: I. Indian J Exp Biol, 6(4), 1968, 232-247.

1. Shukla S, Mehta A, John J, Mehta P, Vyas SP, Shukla S. Immunomodulatory activities of the ethanolic extract of Caesalpiniabonducella seeds. J Ethnopharmacol. 2009 Sep 7;125(2):252-6. doi: 10.1016/j.jep.2009.07.002. Epub 2009 Jul 14.
2. Surya Kant KALAUNI et.al., Antimalarial Activity of Cassane- and Norcassane-Type Diterpenes fromCaesalpinia crista and Their Structure–Activity Relationship, Biol. Pharm. Bull. 29(5) 1050—1052 (2006)
3. Rhitajit Sarkar et.al., Hepatoprotective Potential of Caesalpinia crista against Iron-Overload-Induced Liver Toxicity in Mice, Evidence-Based Complementary and Alternative Medicine Volume 2012, Article ID 896341, 9 pages

T. Seshagiri Rao et. Al. Clinical Trial of Ayush-64 in sieepada. Jour. Res. Ay. Sid., Vol III, No. 1 & 2 PP-9-12

K. D. Sharma et.al. A Clinical Trial of Ayush 64" (A Coded Antimalarial Medicine) in Cases of Malaria. Jour. Res. Ay. Sid., Vol. II. No. 4

P.N. Pandey and Prem Kishore. Effect of Ayush-64 and SaptaparnaghanaVati on Microfilaraemia. J.R.A.S. Vol. XII, No. 3-4, pp. 145-150

P.N. Pandey et.al. An Epidemiological Survey on Microfilaraemia in the Villages Around Bhubaneswar with Therapeutic Effect of Ayush-64. J.R A.S. Vol. XII, No. 1-2, pp. 33·40

D.Bhatia. Role of Ayush- 64 In Malaria Epidemic. JR.A.S. Vol. XVIII, No. 1-2, (1997) pp.71-76

M.V. Chari et. Al. A Double-Blind Clinical Trial with Ayush-64 an Ayurvedic Drug in P. vivax Malaria. Jour. Res. Ay. Sid. Vol. VI. No.1, 3 & 4 pp. 105-116

Gundeti MS et al. Pilot evaluation of safety and efficacy of an Ayurvedic formulation (AYUSH 64) for accelerating the recovery in cases of Influenza like Illness. in press- JAIM

J.L.N Sastry. An indigenous herbal formulation to combat Falciparum Malaria. J of Indian system of medicines and Homeopathy. 1999

## Appendix E: Yashtimadhu ([*Glycyrrhiza glabra*](https://www.sciencedirect.com/topics/pharmacology-toxicology-and-pharmaceutical-science/glycyrrhiza-glabra)) – A Research Update

SARS-CoV-2 is an envelope, positive-sense, single-stranded RNA β-corona virus and shown similarity with severe acute respiratory syndrome (SARS) and Middle East respiratory syndrome (MERS) viruses. Since its origin, scientists and physicians are making efforts to understand its entry and pathogenicity, and the most notable findings regarding its entry into host revealed out, where several SARS-CoV-2 enters into cells through binding of its spike proteins with host cell receptors, angiotensin-converting enzyme 2 (ACE2) and leads to down regulation of the ACE2 receptor. This leads to viral entry and replication, as well as severe lung injury. The occurrence of COVID-19 elevated intense attention not only within India but internationally. Therefore, there is an urgent need for its management and prevention.

[*Glycyrrhiza glabra*](https://www.sciencedirect.com/topics/pharmacology-toxicology-and-pharmaceutical-science/glycyrrhiza-glabra) (family Fabaceae), commonly known as [licorice](https://www.sciencedirect.com/topics/pharmacology-toxicology-and-pharmaceutical-science/liquorice), one of the important an herbaceous perennial Ayurvedic medicinal has been used as a [flavoring agent](https://www.sciencedirect.com/topics/pharmacology-toxicology-and-pharmaceutical-science/flavoring-agent) in foods and medicinal remedies for thousands of years. Licorice root has been widely used around the world to treat cough since ancient times. It contains active compounds, including glycyrrhizin, glycyrrhetinic acid, [flavonoids](https://www.sciencedirect.com/topics/pharmacology-toxicology-and-pharmaceutical-science/flavonoids), [isoflavonoids](https://www.sciencedirect.com/topics/pharmacology-toxicology-and-pharmaceutical-science/isoflavonoids), and [chalcones](https://www.sciencedirect.com/topics/pharmacology-toxicology-and-pharmaceutical-science/chalcone-derivative). Glycyrrhizin and glycyrrhetinic acid are considered to be the main active components and are potent inhibitors of [cortisol](https://www.sciencedirect.com/topics/pharmacology-toxicology-and-pharmaceutical-science/hydrocortisone) metabolism, due to their steroid-like structures. The root of this plant has been used for cough, colds, asthma, and COPD (Ram et al., 2011).

Beside this Licorice is a very well known herb in traditional Chinese medicine (TCM). In China, it is called “gancao” (meaning “sweet grass”) and has been recorded in the Shennong's Classic of Materia Medica around 2100 BC. Threfore, plant has been widely used for prevention and treatment of infections all over the world including in China for prevention of COVID-19. There is clinical evidence of Chinese Medicine on the prevention of severe acute respiratory syndrome (SARS) and H1N1 influenza (Cinatl, J.; Lancet 2003), and Chinese Medicine prevention programs issued by health authorities in China since the COVID-19 outbreak were retrieved from different databases and websites (Liqiang Wang Acta et al PharmaceuticaSinica B)

The extract of liquorice roots rich in glycyrrhizinate and compound diammonium glycyrrhizinate were conscripted to control COVID-19 (*Chinese clinical trial:*<http://www.chictr.org.cn/showprojen.aspx?proj=49131>). Beside this *Glycyrrhiza glabra*, has long been employed against coughs and colds as well as to settle disturbed digestion, while one of its compound diammonium glycyrrhizinate has antiinflammatory activity and is used to treat liver damage caused by hepatitis B (Jun-ling Ren et al Pharmacological Research). Professor Hong Ding of Wuhan University has proposed a combination of diammonium glycyrrhizinate and vitamin C as a COVID-19 therapy. This approach became popular through social media and reporting in publications such as the Health Times (JiankangShibao). It has not been officially recommended, but clinical trials have recently been approved (*Editorial: Nature Plants 2020*).

CSIR-IIIM has strong natural product chemistry. It has its own experimental farms at different locations in Jammu and Kashmir. It has got one of the largest herbarium and crude drug repository. It has in-house expertise and infra structure to enable botanical/phytopharmaceutical drug development. It has successfully completed phase I clinical trial of one of the botanical lead while several IND candidates are in pipeline. Therefore, in this direction, CSIR-IIIM has proposed to develop *Glycyrrhiza glabra* based AYUSH lead for management of COVID-19.

Therefore, this project is proposed to develop and commercialize the aqueous extract based formulations of *Glycyrrhiza glabra* root as product for management/treatment of Corona virus disease 2019 (COVID-19) under regulatory regime of AYUSH.

**Rationale of the study supported by cited literature**

*Glycyrriza glabra* has been widely used as a cough-relieving medicinal herb from ancient times in Indian System of Medicine as well as in Tradition Chinese Medicine. There is clinical evidence of Chinese Medicine on the prevention of severe acute respiratory syndrome (SARS) and H1N1 influenza (Cinatl, J.; Lancet 2003), and Chinese Medicine prevention programs issued by health authorities in China since the COVID-19 outbreak were retrieved from different databases and websites (Liqiang Wang Acta et al PharmaceuticaSinica B)

The extract of liquorice roots rich in glycyrrhizinate and compound diammonium glycyrrhizinate were conscripted to control COVID-19 (*Chinese clinical trial:*<http://www.chictr.org.cn/showprojen.aspx?proj=49131>). Beside this *Glycyrrhiza glabra*, has long been employed against coughs and colds as well as to settle disturbed digestion, while one of its compound diammonium glycyrrhizinate has antiinflammatory activity and is used to treat liver damage caused by hepatitis B (Jun-ling Ren et al Pharmacological Research). Professor Hong Ding of Wuhan University has proposed a combination of diammonium glycyrrhizinate and vitamin C as a COVID-19 therapy. This approach became popular through social media and reporting in publications such as the Health Times (JiankangShibao). It has not been officially recommended, but clinical trials have recently been approved (*Editorial: Nature Plants 2020*).

The plant *Glycyrrhiza glabra* is also a major constituent in different poly herbal formulations/ therapeutic effect used in preliminary clinical trials in management of corona virus disease (COVID-19) (Yang et al. 2020; <http://www.chictr.org.cn/showprojen.aspx?proj=49131>, Jun-ling et al, 2020, Editorial: Nature, 2020).

This indicates that the plant has very high potential to reduce the symptoms of COVID-19 like severe acute respiratory syndrome (SARS) and influenza, cold and coughs and may prove as an add-on candidate for in the field of management of COVID-19. The liquorice roots are also used traditionally as well as commercialized herbal extracts for different therapeutics; therefore, the aqueous extract is preferred for developing an AYUSH product.

In this proposal, the aqueous extract will be prepared and standardized based on the isolated four markers from roots of *Glycyrrhiza glabra* for product development in the area of COVID-19 as per AYUSH regulatory guidelines.

**Current status of research and development in the patient (both international and national status)**

Traditionally, medicinal plants have been used for many years by different cultures round the world for the management of infection. In the current situation, investigation on herbal/botanical medicines has become progressively important in the search for a new, effective and safe therapeutic agent for the treatment/management of COVID-19 infection. Based on the traditional knowledge, use of *Glycyrrhiza glabra* around the world to treat cough since ancient times and used as major ingredient in different polyherbal formulations in China as well as use of its major marker diammonium glycyrrhizinate and vitamin C in combination as a proposed therapy for COVID-19 the plant has promising indications in management of COVID-19.

The extract is rich in various secondary metabolites including glycyrrhizin, glycyrrhetinic acid, [flavonoids](https://www.sciencedirect.com/topics/pharmacology-toxicology-and-pharmaceutical-science/flavonoids), [isoflavonoids](https://www.sciencedirect.com/topics/pharmacology-toxicology-and-pharmaceutical-science/isoflavonoids), and [chalcones](https://www.sciencedirect.com/topics/pharmacology-toxicology-and-pharmaceutical-science/chalcone-derivative). Glycyrrhizin and glycyrrhetinic acid are considered to be the main active components and are potent in management of COVID-19. The compound diammonium glycyrrhizinate were conscripted to control COVID-19 (*Chinese clinical trial:*<http://www.chictr.org.cn/showprojen.aspx?proj=49131>).

Further the aqueous extract is non toxic and safe with proposing efficacy in COVID-19. Now we want to develop AYUSH product from the plant lead by preparation cGMP extract with its CMC and formulations following the AYUSH route.

**The relevance and expected outcome of the proposed study**

**Preliminary work done so far**

Scientists of IIIM Jammu are working on the plant in one of CSIR project i.e. Phytopharmaceutical Mission. Therefore, IIIM Jammu is having raw material around 200 kg in hand and has prepared the aqueous extract of root with extractive value >20% at lab scale which was also successfully scaled up at our cGMP plant.

Two cGMP batches have been prepared of the aqueous extract at our facility. Further CMC of the plant material/the extract including standardization of the extract is going on at our QC/QA department. The isolation of 4 markers i.e. Glycyrrhizic Acid, Glycyrrhetinic acid, Glabridin and Glabrol shown in Figure 1 in major quantity along with some other 5 minor compounds has been completed.

Fig 2: Structure of major markers isolated in gram quantity from *Glycyrrhiza glabra*

*Based on the preliminary work undertaken on Glycyrrhiza glabra at CSIR-IIIM, Jammu and its use in China for Covid-19, it is proposed to develop AYUSH product of G. glabra through this project in collaboration with Medanta Hospital.*

**References**

1. Kavita Gulati, Nishant Rai, Sulekha Chaudhary and Arunabha Ray. Nutraceuticals in Respiratory Disorders
2. Gulati, K., Rai, N., Chaudhary, S., & Ray, A. (2016). *Nutraceuticals in Respiratory Disorders. Nutraceuticals, 75–86.* doi:10.1016/b978-0-12-802147-7.00006-1
3. Ram, A., Balachandar, S., Vijayananth, P., & Singh, V. P. (2011). Medicinal plants useful for treating chronic obstructive pulmonary disease (COPD): Current status and future perspectives. Fitoterapia, 82(2), 141–151. doi:10.1016/j.fitote.2010.09.005
4. Yang Yang, Md Sahidul Islam, Jin Wang, Yuan Li and Xin Chen. Traditional Chinese Medicine in the Treatment of Patients Infected with 2019-New Coronavirus (SARS-CoV-2): A Review and Perspective. International Journal of Biological Sciences 2020; 16(10): 1708-1717. doi: 10.7150/ijbs.45538
5. Hoffman, M. Kleine-Weber H., Schroeder S., Krüger N., Herrler T., Erichsen S., Schiergens T. S., Herrler G., Wu N. H., Nitsche A., Müller M. A., Drosten C., Pöhlmann S. SARS-CoV-2 Cell Entry Depends on ACE2 and TMPRSS2 and Is Blocked by a Clinically Proven Protease Inhibitor". *Cell.* Retrieved **2020**-03-05 DOI: <https://doi.org/10.1016/j.cell.2020.02.052>.
6. Editorial: Redeploying plant defences; Nature Plants 2020 VOL 6, 177 13 <https://doi.org/10.1038/s41477-020-0628-0>
7. A randomized, open, controlled trial for diammonium glycyrrhizinate enteric-coated capsules combined with vitamin C tablets in the treatment of common novel coronavirus pneumonia (COVID-19) in the basic of clinical standard antiviral treatment to evaluate the safety and efficiency <http://www.chictr.org.cn/showprojen.aspx?proj=49131>
8. Cinatl, J.; Morgenstern, B.; Bauer, G.; Chandra, P.; Rabenau, H.; Doerr, H.W. Glycyrrhizin, an active component of liquorice roots, and replication of sars-associated coronavirus. Lancet 2003, 361, 2045–2046.
9. Liqiang Wang, Rui Yang Bochuan Yuan Ying Liu Chunsheng Liu The antiviral and antimicrobial activities of licorice, a widely-used Chinese herb. Acta PharmaceuticaSinica B 2015;5(4):310–315
10. Jun-ling Ren, Ai-Hua Zhang, Xi-Jun Wang.Traditional Chinese medicine for COVID-19 treatment.PharmacologicalResearch[Volume 155](https://www.sciencedirect.com/science/journal/10436618/155/supp/C), 2020, 104743
11. R.A. Isbrucker, G.A. Burdock. Risk and safety assessment on the consumption of Licorice root (Glycyrrhiza sp.), its extract and powder as a food ingredient, with emphasis on the pharmacology and toxicology of glycyrrhizin. Regulatory Toxicology and Pharmacology 46 (2006) 167–192.
12. Yu-Jin Kwon, Da-Hye Son, Tae-Ha Chung, and Yong-Jae Lee.

## Appendix F: Patient Information Sheet

AYUSH COVID-19 PATIENT PROTOCOL 2020

PATIENT INFORMATION FACT SHEET

STUDY DOCTOR:_________________________________

INSTITUTION ADDRESS:___________________________________________________

__________________________________________________________________________

Phone Number:________________________

Protocol Title:

A Randomized, Open Label, Parallel Efficacy, Active Control, Multi-Centre Exploratory Drug Trial to Evaluate Efficacy and Safety of an Ayurvedic Formulation as Adjunct Treatment to Standard of Care for the Management of Mild to Moderate COVID-19 patients

Protocol Number: ________________________

1) What is a Research Study?

You have been requested to participate in a clinical research study. This study will test an Ayurvedic experimental drug for the treatment of Mild and Moderate COVID-19.

There is no specific medicine yet with clear cut evidence for treatment of COVID-19.

COVID- 19 is a new virus that has caused a rapid spread of infection all over the World and is called a pandemic. The virus usually attacks lungs and airway passage. Patients often suffer from fever, cough and breathing difficulties. Many individuals will recover from the virus infection without suffering any symptoms but can transmit the virus to their contacts. The majority of patients will suffer from mild to moderate symptoms and will also recover. Some patients with mild to moderate disease may progress to severe stage and require special critical care including oxygen and ventilators and other supportive therapy. A small number of patients suffering from severe diseases may die due to severelung and other complications. Elderly people and those suffering from overweight, diabetes, hypertension and heart ailments run a higher risk of severe disease. COVID-19 is a highly infectious disease and rapidly spreads from man to

The Patient Information sheet and Informed Consent describe the study to you. Your study doctor will discuss this form with you. Your study doctor will reply to all the queries you have about the information on this form

If you agree to participate, you will be told to sign and date this form. You will be handed over a signed and dated copy of this form to keep in your records. Your decision to take part in this study is absolutely voluntary. At any point of time during the procedure of obtaining informed consent, the study participant can withdraw from the process and refuse to sign.

This project is sponsored by the Ministry of AYUSH, Government of India.

2) What is the purpose of this study?

Currently the Standard of Care is mostly symptomatic treatment and antibiotics are added to prevent secondary infections. If the patient deteriorates and this is mostly due to lung complications, critical care with intravenous fluids, oxygen and ventilator may be required. Patients may also need medicines to maintain blood pressure and adequate circulation as part of standard of care. The purpose of this study is to improve the current standard of care by adding a standardized Ayurveda formulation. But this needs evidence and in this research study the clinical benefit of adding a standardized Ayurveda formulation to standard of care will be assessed by comparing with patients only receiving standard of care. One of the 3 selected Ayurveda formulations (SamshamaniVati Plus, AYUSH 64 and Yashtimadhu will be added to the standard of care. It is expected that such a combination of standard of care plus Ayurveda formulation will lead to improved and early recovery from COVID 19 and also prevent complications and progression to severe stage. It is expected that this new approach will also reduce the mortality from COVID 19.

3) How does this study work?

If you agree to join this study, you will be one of the approximately 140 volunteering patients in this study. The study will take place in _________centers in India.

This is a randomized, prospective, open label, parallel efficacy, multicentric, two arm study to compare the effectiveness of a combination of selected Ayurveda herbal drug and standard of care (SOC) to that of only SOC. The Ayurveda drug is being considered as an add-on therapy.This means that you and the treating doctors will know whether you are receiving the Ayurvedic drug. By randomized it means that a computer program will decide based on some kind of a by chance selection calculations (like throwing a dice) whether you will be treated by only standard of care or you will be treated by standard of care plus Ayurvedic formulation.

4) How long will you be on the study?

The total study duration is of 12 weeks which includes a period of admission into hospital. The hospitalization may last from few days to few weeks. Most of the patients recover within 20 days. After discharge from hospital you will continue to be monitored for complete recovery and good health as an outpatient till you complete 12 weeks.

5) What are your responsibilities?

You have voluntarily consented to participate in this study. The study investigators and doctors and their team members will take utmost care to look after you well. In case you suffer any kind of discomfort or illness or have a query any time during the study period, the study team will respond to you in an appropriate timely courteous manner. However, please study this document carefully and participate wholeheartedly. Please adhere to all the advise given to you by the study investigator and doctors from time to time. Though you have consented with your free will and knowledge, you may choose not to take part or may leave the study at any point of time.

If you choose to participate in this study, there are some rules you should follow.

- Throughout this study women should not become pregnant
- Women must not breastfeed throughout this study
- It is very essential that you inform your study doctor all of the information you know regarding your health, symptoms and medications, even the over the counter medications, vitamins and herbal products, that you have taken in the past, are presently taking. If you do not let the study doctor know everything, you maybe putting your health at risk.
- You are not permitted to take specific medication while taking study drugs.
- You must follow all instructions given to you while you are taking part in this study. If you are unsure about anything please ask your study doctor
- You need to undergo specific tests or procedures as applicable to the study
- You should not simultaneously participate in any other clinical drug project/trial

6) What are the study drug details?

Please remember that if you agree to participate in this research program you will be given only one of the 3 Ayurveda formulations as per the study selected for your particular hospital. All the 3 drugs have been popularly used in Ayurveda practice over a long period and are considered generally safe. There are no reports of any serious drug side effects or any kind of reaction in clinical experience of Ayurvedic physicians. But still caution is required and you will be carefully monitored for any drug side effect. Some of the specific details of each of the 3 Ayurveda formulations is described below.

i) SamshamaniVati Plus: This drug is a combination of 2 plants namely

Tinosporacordifolia (Guduchi) and Piper longum (Pippali).Both these plants are very well studied and documented for clinical use to recover from viral infections like cold and flu and also improve the immunity of the person. This particular combination has been described in the treatment of flu like febrile illness with cough and other lung symptoms. COVID-19 is also an acute febrile illness with sore throat, cough and breathing difficulty and thus SamshamaniVati plus may help in recovery from COVID 19.
Some reports show its benefit in other viral illnesses such as chikungunya. TheMiinistry of Ayush has also issues an advisory for the benefit of public to improve their immunity status using drugs like SanshamaniVatiand this will help in prevention against COVID 19 and may help recovery in case of actual infection.

Dosage:

SamshamaniVati Plus will be given as two tablets (each containing 300 mg aqueous extracts of Tinospora cordifolia and 75 mg aqueous extracts of Piper longum) twice daily with warm water.

ii)AYUSH-64: The AYUSH-64 is a multi-plant formulation. The plants extracts are known to reduce inflammation and strengthen the immune system. It is also known to relieve fever of different cause and also when complicated by sore throat and cough and lung problems. It has extensive experience from treating 13,000 patients during the malaria epidemic of 1984. It has been used for liver disorders. The Ministry of Ayush has also recommended the use of AYUSH-64 as a preventive measure for COVID 19.

Dosage:

The dose of AYUSH - 64 will be two tablets (500 mg each) twice daily. Each tablet will contain aqueous extracts of Alstoniascholaris (100 mg), Picrorhizakurroa(100 mg), Swertiachirata(100 mg), and Caesalpinia crista (200 mg).

iii)Yashtimadhu (Glycyrrhiza glabra): Commonly known as licorice, it is one of the important Ayurvedic medicinal plant that has been widely used around the world including Traditional Chinese Medicine to treat cough, colds asthma, and chronic pulmonary disease since ancient times. In Indian houses, it is called ‘Muleti’ and is popularly used since ancient times.

It is used as a medicine to strengthening lungs and airways. It is a potential medicine for treatment of COVID 19. In fact, it is already being used popularly to obtain relief in COVID 19 in China.

Dosage: The dose of Glycyrrhiza glabra will be two tablets (300 mg each) twice daily.

7) Can I take other medicines?

Medication prescribed for any other co-existent illness (e.g diabetes, Hypertension etc) or fresh common ailments like other infectionsaetccan be taken as prescribed by your family doctor. But you will be requested to show the prescription to the study investigator so that this can be recorded in your case record. You will be contacted by the study nurse and study doctor to check your daily progress.

8) How often will I be seen and what all will be done by the physician?

The study physicians will examine you at regular intervals after you join the study. In the beginning while you are admitted in the hospital you will be seen and examined daily till you recover and discharged. Following this you will be seen as per study schedule every 4 weeks till you have completed 3 months. During each visit when you are ill with COVID 19, the doctor will perform several laboratory tests to find out your health status and recover. Some tests will be done to detect any complication taking place in any organ like lung, liver, kidney, heart and circulation. During the study some special tests will be done to study the responses in the immune system and blood circulation. The clinical examinations will be routine but focused on COVID 19 and lung function specially. Your body temperature and body weight will be recorded often. You will also be required to take X-Ray chest and in need be a CT scan of the chest. Other routine investigations will include ultrasonography of your abdomen and an electrocardiography (ECG) for the heart- a color Doppler may be done to check your heart. You will be asked for any drug related side effect from time to time during the study. Your symptom assessment, vital parameters and monitoring of adverse events will be performed daily till discharge by the study doctor and recorded in your study progress sheet. The Ayurvedic Physician will also monitor you daily. After discharge you will also be followed and advised on a mobile phone application to be downloaded for this purpose.

9) How long will I be in the study?

The maximum duration of the study will be 12 weeks followed by a post treatment follow up at 12 weeks after your admission in the hospital. However, your Ayurveda treatment will be discontinued if it is found that:

• Your disease worsens or you need ICU admission or ventilator support due to any reason

• Non-compliance of the treatment regimen

• You withdraw consent

• You suffer any serious adverse event

• Any other serious medical condition which you may acquire during the course of the trial which makes it difficult for you to continue in the trial

• Any other condition which the investigator feels might cause harm to you.

• You are not taking the study medication regularly

• You are unable to tolerate the study medication

• New Information becomes available that indicates the study treatment is no longer in your best interest

No matter which group you are assigned to, and even if you stop treatment early, we would like to keep track of your medical condition for at least 6 months to see long-term effects of study treatments in an informal manner on telephone or a personal visit.

10) What investigations will be done?

• Special test will be carried out on joining the study and daily till discharge or as required by the treating physician depending on your progress. Special blood tests will also be carried out to study immune mediated inflammatory response by cytokine assay (such as gamma interferon, IL-2, IL-6, IL 4, anti-TNF, IL 13, IL-10, MCP etc). Biomarkers such as Superoxide Dismutase (SOD), Glutathoine (GSH), Serum Immune response tests (IgG and IgM) for COVID-19. Considering good progress and early recovery, 10-15 ml of blood will be required every 2-3 days over a period of 10-14 days for blood tests in the study-this will also include check blood hemoglobin to make sure that you don’t suffer from anemia or any other complication. This is often done during hospitalization of acute infections lasting 7-10 days. In case your disease becomes serious with complications you will need more blood to be taken for blood tests.

• Other investigations such as Ultrasound of Abdomen and Pelvis, Color Doppler, Chest Radiograph, HRCT chest, ECG will be performed.

On joining and completion of study your blood will be checked for routine parameters of health such as hemoglobin, blood sugar, liver and kidney function tests, cholesterol and other related fractions, tests to detect any inflammatory response (ESR, C-Reactive protein, serum ferritin)

.

11) How will my blood and other test samples be disposed? Will you preserve them for any future research?

All blood samples and other study related samples and specimen collected during the duration of the study will be disposed of as per current rules and regulations on the patient. However, sometimes blood and or serum samples are preserved for future medical research with full protection of the confidentiality of the identity of the patient concerned. This kind of future medical research will also adhere to regulatory and ethic clearance as per the law of the land. In case you do not wish to let your blood sample and other study related body samples be preserved for future medical research please notify the study investigator and the same will be stated on this informed consent.

12) What will happen if my disease worsens?

If the treating Physician realizes your disease is worsening you maybe shifted to the ICU for further management of your disease. The study medication will be stopped in such situation.

13) What are the risks of Standard of Care mentioned in this study?

Most of the patients suffering from COVID 19 in this study will only require symptomatic treatment for cough and fever. If the disease progresses you may need antibiotics and several other potent medicines to ensure your recovery. In the ICU many drugs are used to maintain breathing and blood circulation. These are all modern medicines which are approved by the suitable regulatory agency for treatment. They are used to treat the disease and its complications effectively but sometimes the disease is very severe and the patient may die. The risk of sideeffects with modern medicine is certainly present and becomes high if high doses of drugs are used for longer periods or in emergency setting of an ICU. Therefore, though there are many risks of drug side effects in different body systems when using modern medicine in serious or emergency situation, the treating physician will take utmost care to safeguard against drug related side effects and even treat them quickly by changing medicine or using other medicines.

14) Can I get pregnant during the study?

Regarding pregnancy, it is advisable that if you are in the reproductive age group please avoid pregnancy. It is best that safe birth control and other contraceptive methods are used after consulting a specialist Gynecologist. There is always a risk of any medicine during pregnancy. Also, there is not enough data on the chances of any adverse effects of the Ayurveda study drug on mother and foetus during pregnancy and the new born child.

15) What are the contraceptive methods advised to prevent pregnancy?

The preferred birth control and other contraceptive methods are as follows:

Intrauterine device (IUD)

Tubal sterilization

Vasectomy in male partner

Participants who use a hormonal contraceptive as one of their birth control methods should have the same method for a minimum of 3 months before the first dose of study drug.

Acceptable hormonal methods (every hormonal method must be used with a barrier method like a condom, preferably a male condom)

Oral contraceptives

Injectable progesterone

Contraceptive vaginal ring

Acceptable barrier methods (should be used with a hormonal method)

Male or female condom with or without spermicide

16) In this study I will be under care of Ayurvedic or Allopathic doctor?

You will be under the combined care of Allopathic and Ayurvedic and both will examine you daily till discharge.

17) Will I be paid to be part of this study?

You will be remunerated to participate in this study and at each pre-arranged schedule visit you will be paid Rs 750/- towards your travel and meal.

18)What are the dietary restrictions and other precautions that i should observe?

Please remember:

• Take your meals on time

Eat balanced diet with plenty of water and fresh fruits. There is no restriction on milk intake. Avoid fasting and eating food from outside sources that may not be hygienic and upset your body system and especially stomach. You may like to avoid excess spices, tea and coffee. Also avoid any use of tobacco and alcohol.

19) How will you look after my confidentiality?

Your name will not be used in any of the papers used for the study. Only the code number assigned to you and your initials will be used throughout the study for identification purposes. No outside person except authorized representatives will have direct access to the study documents. In any case, your personal data will be kept confidential to the extent permitted by Indian laws and regulations. If the results are published your name will not be used.

20) What are the costs that i have to bear in this study?

The study medication will be given to you free of charge as long as you are in the study. Also you will not be charged for any of the tests or consultations that are required for the study. Overall, the study is free of cost.

In case you develop any other illness or any study trial related illness/toxicity/injury you will be advised to seek admission in a general ward of the Government hospital, such a patient will be entitled to free medical management as long as needed or till such time it is established that the injury is not connected to the clinical trial, whichever is earlier.

In case of a patient’s trial related injury or death, sponsor will pay for the medical management and financial compensation for such trial related injury or death. Such medical management will cover the treatment required to treat the trial related injury. A trial related injury or death is the injury/death directly caused to the patient by the administration of the study drug under investigation or by the study procedures included in the protocol to which the patient would not have been exposed otherwise than by his/her involvement in this study. Compensation of such trial related injury or death should be for the reasons as mentioned under Rule 122-DAB (5) of the Drugs and Cosmetics Rules, 1945. The sponsor will provide adequate insurance to cover any kind of financial liability that may arise out of participation in this study.

21) WHOM DO I CALL IF I HAVE QUESTIONS OR PROBLEMS?

Till in this study, you will be under the supervision of Dr ________________________. If at any time you feel that you are having problems or side effects or if you have a study related injury, please get in touch with your study doctor. The telephone number to contact your study doctor or other study personnel is:

DR _________________________________Mobile Number:_____________________________

This consent form and study have been approved by the Institutional Ethics Committee. The EC is a group of scientific and non-scientific people who supervise research that is done on people. They follow the guidelines and rules of Central Drug Standard Control Organization. For any queries your rights as a research participant, please contact;

Member Secretary Name: _____________________________________________________

Name and Address of Ethics Committee:__________________________________________

___________________________________________________________________________

22) WHERE CAN I FIND MORE INFORMATION ABOUT THIS STUDY?

As required by the Indian Law:

A description of this study will be posted on the website <http://ctri.nic.in/Clinicaltrials/login/php>. This website will not have information that can identify you. You can see this website any time.

##

## Appendix G: Informed Consent Form

**Informed Consent Form**

Patient No: Patient Initials:

Study Title: **(*AN AYUSH- ICMR CLINICAL RESEARCH PROGRAM)***

A Randomized, Open Label, Parallel Efficacy, Active Control, Multi-Centre Exploratory Drug Trial to Evaluate Efficacy and Safety of an Ayurvedic Formulation as Adjunct Treatment to Standard of Care for the management of Mild to Moderate COVID-19 Patients

***Study Number: AYUSH-CSIR-TRT-01***

Patient Initials: Name: Date of Birth (dd/mm/yyyy)/Age: Address of the Participant:

Qualification:

Occupation: (Student/Self Employed/Service/Housewife/Others)-Please mark on appropriate

If Others (Please mention):

Annual Income of the participant:

Nominee details: (for the purpose of compensation in case of trial related death)

Name of nominee (s):- Address of the nominee(s):

Relation of the Participant with

nominee(s):

The details of the study (AYUSH-CSIR-TRT-01) have been explained to me by the Investigator in the language that I understand.

My signature on this consent form means the following:

Patient Initials

| (I) | I verify that I have studied and understood the Patient Information Fact Sheet dated for the above study and have had the chance to ask  questions. The study has been fully explained to me and all my questions have been answered satisfactorily | [ ] |
| --- | --- | --- |
| II) | I fully understand the implications, requirements and risks of this project | [ ] |
| III) | I understood that my involvement in the study is voluntary and that I am free to leave at any time without giving any reasons, without my medical care or legal rights getting affected | [ ] |
| IV) | I authorize the Investigator to carry out all necessary laboratory tests and other investigations required by the study. My blood and other related specimens may be used for future research study if relevant to my current ailment. | [ ] |
| V) | I have understood that the sponsor of this trial, others working on behalf of the sponsor, the ethics committee and the regulatory authorities will not require my permission to see my health records both in respect of the present study and any further research that may be carried out in relation to it, even if I withdraw from the trial. I agree to this access. However I understand that my identity will not be disclosed in any information provided to the third parties | [ ] |
| VI) | I agree to my taking part in the above study | [ ] |

Date of Informed Consent Process:

Name of the Patient: Date:

Signature of the Patient (Left hand thumb impression if participant is illiterate or cannot sign)

Name of Legally Acceptable Representative (If Applicable)

Signature: Date:

Relationship to participant (e.g Parent, Guardian, etc)

Name of the person who conducted the informed consent discussion:

Name: Date:

Signature of the Investigator

Impartial Witness Name (If Applicable)

Date: Signature of Witness

Copy of this Patient Information Fact Sheet and completed ICF shall be provided to the participant

*(The legally acceptable representative signature must be added if the patient cannot sign for themselves. The relationship between the patient and the legally acceptable representative must be stated*

*The Impartial witness signature must be added if the patient and legally acceptable representative cannot read or write.)*

## Appendix H: Patient Case Record Form

**AYUSH COVID-19 PATIENT PROTOCOL 2020**

**PATIENT CASE RECORD FORM**

**PATIENT NAME________________________________________________**

**ADDRESS: □URBAN □RURAL**

**____________________________________________________________**

**________________________________________________________________**

**________________________________________________________________**

**CONTACT NUMBER:___________________________________________**

**EMERGENCY CONTACT NUMBER : ____________________________**

**STUDY SITE LOCATION**________________________________________

**DATE OF INFORMED CONSENT__________LANGUAGE OF INFORMED CONSENT ________**

**STUDY GROUP:** □ **STANDARD OF CARE** □**AYURVEDA**

Name of Drugs_____________________________________________________

ADMISSION DATE TO HOSPITAL___________________________________

STATUS OF DISEASE ON ADMISSION: □ MILD □MODERATE

Note: Please check ‘INCUSION and EXCLUSION CRITERIA’ on page 4

**AYUSH COVID-19 PATIENT PROTOCOL 2020**

**CASE RECORD FORM**

**INITIAL**

**Id:______________**

PATIENT NO: ________________ PATIENT INITIALS _____________

SURNAME: ________________FIRSTNAME___________________MIDDLENAME ______________ AGE ________years; □ HOSPITAL SITE :_________________________________________________

SEX: □ M □ F, Birth Date: ________________ WT: _______kg, HT: _________ cm, BMI__________

OCCUPATION: □LABOUR □ DESK JOB □FIELD WORK □HOUSE WORK □POLICE □NURSE □DOCTOR □MEDIA□CLEANING STAFF □ OTHERS__________________________________________________

EDUCATION: □NIL □<5,□5-12 □ABOVE 12 □GRADUATE□> POST GRADUATE □OTHERS________

STATUS: □SINGLE □MARRIED□□WIDOWED□DIVORCED □OTHERS_______________

PERSONAL HISTORY: □ SMOKING; □ DAILY □ INFREQUENT □ MORE THAN 6 MONTHS

□TOBACCO ;□DAILY □ INFREQUENT □ MORE THAN 6 MONTHS

□ ALCOHOL; □ DAILY □ INFREQUENT □ MORE THAN 6 MONTHS

Does any other close member of the family has COVID-19 □ Yes □ No; If Yes

Describe:________________________________________________________________________

Were you patiented to Quarantine? □ Yes □ No; If Yes, where____________________________

Number of days quarantined________________________________________________________

ALLERGY TO DRUGS: □NIL □ YES; □ SULFA□ PENICILLIN □ ANY OF THE STUDY DRUG INGREDIENTS

Present Illness:

Did you take any medicine in the past 7 days □ Yes □ No; If Yes _________________________

**____________________________________________________________________________________**

**HISTORY OF PRESENT ILLNESS:**

DATE OF ONSET:____________________________

SYMPTOMS ON DAY ONE: □ FEVER □ SORE THROAT □DRY COUGH □ SHORTNESS OF BREATH □ OTHERS_________________________________________________________

_______________________________________________________________________________

CONCOMITANT ILLNESS (INDICATE DURATION)

| **DISEASE** | **DURATION** | **DISEASE** | **DURATION** |
| --- | --- | --- | --- |
| **□** HYPERTENSION |  | **□** ACID PEPTIC DISEASE |  |
| **□** DIABETES |  | **□** PILES |  |
| **□** HYPERLIPIDEMIA |  | **□**LIVER DISEASE |  |
| **□** CARDIAC DISEASE |  | **□**NEUROLOGICAL DISEASE |  |
| **□**CHRONIC LUNG DISEASE |  | **□** RENAL DISEASE |  |
| **□**OBESITY |  | **□** OTHERS |  |

CONCOMITANT THERAPY:

| Indication | Drug  (Generic name) | Strength / Route | Frequency | Start Date | End Date |
| --- | --- | --- | --- | --- | --- |
|  |  |  |  |  |  |
|  |  |  |  |  |  |
|  |  |  |  |  |  |
|  |  |  |  |  |  |

**VITAL SIGNS:**

Temp: ______Pulse rate: _____Respiratory Rate : _______Blood Pressure: ________mm Hg

**BASELINE SYMPTOMS: (OF ANY ETIOLOGY)**

|  | NO | YES | IF YES, DESCRIBE |
| --- | --- | --- | --- |
| Headache |  |  |  |
| Disturbed sleep |  |  |  |
| Anorexia |  |  |  |
| Oral Ulcers |  |  |  |
| Retrosternal Burning or Discomfort |  |  |  |
| Poor Digestion |  |  |  |
| Eructation |  |  |  |
| Constipation |  |  |  |
| Altered Bowel Habits |  |  |  |
| Body ache |  |  |  |
| Tingling numbness |  |  |  |
| Itching |  |  |  |
| Skin rash |  |  |  |
| Irregular menses |  |  |  |

Physical Examination (Please tick)

|  | Normal | Abnormal | If Abnormal, please comment |
| --- | --- | --- | --- |
| General Appearance | **□** | **□** |  |
| Skin | **□** | **□** |  |
| Head, Neck & Thyroid | **□** | **□** |  |
| Eyes | **□** | **□** |  |
| Nose and Throat | **□** | **□** |  |
| Lymph Nodes | **□** | **□** |  |
| LUNGS | **□** | **□** |  |
| Cardiovascular | **□** | **□** |  |
| Abdomen | **□** | **□** |  |
| Central Nervous System | **□** | **□** |  |
| Others – Specify | **□** | **□** |  |
|  | **□** | **□** |  |
|  | **□** | **□** |  |

Remarks: ________________________________________________________________________

________________________________________________________________________________

________________________________________________________________________________________________________________________________________________________________________________________________________________________________________________

Completed by: __________________________

Signed _________________________________

Dated : _________________________________

Please see Inclusion Exclusion Criteria on reverse

**INCLUSION CRITERIA:**

|  |  | YES | NO |
| --- | --- | --- | --- |
| 1 | Typical Clinical presentation of acute onset febrile illness with cough and a RT_PCR based laboratory confirmation test for COVID-19 |  |  |
| 2 | Typical Clinical presentation of acute onset febrile illness with sore throat and dry cough with or without shortness of breath in a patient from a known ‘hot spot’ area or in close contact with a confirmed COVID 19 case with a negative laboratory test for COVID 19 and H1N1 influenza |  |  |
| 3 | Patients with either sex, 18 to 75 years age |  |  |
| 4 | Patients with mild-moderately severe disease |  |  |
| 5 | All patients must agree to refrain from donating blood while on study drug and for 28 days after discontinuation from this study treatment |  |  |
| 6 | All patients must agree not to share medication |  |  |
| 7 | Patients willing to participate and sign an informed consent |  |  |


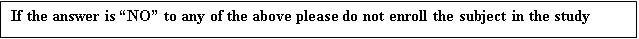


**EXCLUSION CRITERIA:**

|  |  | YES | NO |
| --- | --- | --- | --- |
| 1 | Patients suffering from severe COVID-19 Disease as judged by a physician and fulfilling at least two of the following three criteria* (i) Respiratory distress at room ambience (≥30 breaths per min) (ii) Oxygen saturation at rest ≤93% (peripheral digital arterial oxymetry) and requiring oxygen support for over one hour to normalize (iii) Any of the known COVID-19 complications and emergency procedures which may require shift/admission in intensive care unit such as respiratory failure, adult respiratory distress syndrome, requirement of oxygen support for over 1 hour, requirement of mechanical ventilation, septic shock, or severe non-respiratory organ dysfunction or failure. |  |  |
| 2 | Chronic, Severe, Unstable, Uncontrolled co-existent medical illness such as Diabetes, Hypertension, Cardiac disorders, liver, kidney disorders and lung disorders or other disease of concern which may put the patient at increased risk during the study |  |  |
| 3 | History of immunosuppression: solid organ or bone marrow transplant, use of immunosuppressive antimetabolic and biologic agents, intrinsic immunodeficiencies, HIV infection. |  |  |
| 4 | Active cancer diagnosis, on palliative treatment or requiring current therapy with antimetabolic agents, immunotherapy or radiotherapy. |  |  |
| 5 | At least one fever every 24 hours for > 72h |  |  |
| 6 | Patients on parenteral nutrition |  |  |
| 7 | Patients with known sensitivity or contraindication to any of the ingredients of study medication |  |  |
| 8 | History of bleeding haemorrhoids, haemoptysis, acid peptic diseases, ulcers and pulmonary diseases (tuberculosis, asthma, etc) |  |  |
| 9 | Patients who are likely to worsen or planed ICU admission or ventilator support due to any reason |  |  |
| 10 | Pregnancy and lactation |  |  |
| 11 | Participation in a drug interventional clinical drug trial of any nature in the 3 month period preceding onset of COVID-19 |  |  |
| 12 | Participation in any other clinical trial of an experimental agent treatment for COVID-19 |  |  |
| 13 | Patients on any kind of Ayurveda treatment or any other alternative and complementary medicinal systems such as Homeopathy, Unani, Siddha and in particular requiring oral therapy of any kind. |  |  |
| 14 | Physician decision that involvement in the study is not in the patient´s best interest |  |  |


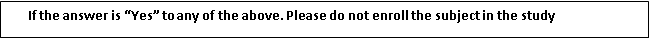


## Appendix I: Ayurveda Case Record Form

**ASSESSMENT AT BASELINE**

1. **Prakriti Questionnaire (Short)**

| **Question** | **Options** | **Dosha** | **Weight** |
| --- | --- | --- | --- |
| The body parts look like (appearance of body parts) | Dry / Cracked / furrowed | V | 2 |
|  | Delicate / tender | P | 2 |
|  | Shapely / beautiful | K | 2 |
| The size of the forehead | Broad and tall forehead | K | 2 |
| The chest (massiveness of chest) | Massive / huge / large chest (ribs hidden, fleshy) | K | 2 |
| The appearance of joints | Well Knit (Well Formed) and Nicely Covered with Flesh | K | 2 |
| Texture of body muscles on touch (to examine the belly of the muscles) | Rigid | V | 2 |
|  | Lax / supple | P | 2 |
|  | Built Well (Without Laxness) / Firm | K | 2 |
|  | Fleshy | K | 2 |
| Colour of the sclera (The colour of the white part of the eyes) | Misty | V | 2 |
|  | Greyish | V | 2 |
|  | Dull | V | 2 |
|  | Reddish eyes (get red immediately in Sunrays; or after alcohol consumption; or after getting angry) | P | 2 |
|  | Clean white, bluish colored eyes with reddishness in the corners | K | 2 |
| The size of the eyes | Small / sunken / narrow | V | 2 |
|  | Medium | P | 0 |
|  | Big, Wild (Wide open) | K | 2 |
| Skin texture (on touch) | Rough and hard | V | 2 |
|  | Soft and supple / loose | P | 2 |
|  | Soft and tight, velvety smooth / silky | K | 2 |
| The temperature of the skin at the room temperature | Cold with dryness (on hands, legs, forehead) | V | 2 |
|  | Warm / hot | P | 2 |
|  | Cold without dryness | K | 2 |
| Early hair loss or early balding | Early hair loss (baldness) | P | 4 |
|  | Excessive and or early graying of hair | P | 4 |
| Overall nature of appetite | Irregular hunger-sometimes intense hunger, sometimes not | V | 2 |
|  | Strong hunger (Sharp and intense) | P | 2 |
|  | Less and less sharp hunger | K | 2 |
| Capacity to skip meals (Tolerance to hunger) | Can tolerate skipping meal easily | K | 4 |
| Stools | Large Quantity | P | 2 |
|  | Very Quick Emptying of Bowels | P | 2 |
| Perspiration (Quantity and incidence) | Excessive and quick sweating (sweating even with less exposure to heat or sunrays) | P | 6 |
|  | Less sweating (sweats only if very hot climate) | K | 2 |
| Movements and activities | Fast / hurried / untidy actions / restless movements (habit / style to move restlessly the neck, hands, lips, eyes, shoulders, head, eye brows, tongue - all or one of these) | V | 4 |
|  | Slow, thoughtful and few movements, deliberate style of activities | K | 2 |
| Speech (speaking style) | Fond of Chit-Chatting All the Time (Chattering / Excessive Talking) | V | 2 |
|  | Fast / Hasty / Fumbling Style of Speaking | V | 2 |
|  | Slow, Rich with Moments of Silence | K | 2 |
| Temperament | Anxious, not composed | V | 1 |
|  | Short tempered | P | 0.5 |
|  | Straight forward | P | 0.5 |
|  | Composed | K | 1 |
| Memory (quality and span) | Quick to remember | V | 0.5 |
|  | Slow to remember | K | 0.5 |
|  | Forgets quickly / accuracy less | V | 0.5 |
|  | Forgets slowly / accuracy more | K | 0.5 |

1. **Did you have any of the following as preventive measure?** (mark if yes)

Warm water Decoction Herbal powder Specific diet

0

0

Yoga Breathing exercise Gargles Milk + Turmeric

0

0

0

Other measures _________________________________________________________

1. **Clinical features**

Heaviness *(Gaurava)* Weakness *(Daurbalya)* Fatigue *(Klama)*

0

0

Vertigo *(Bhrama)* Sleepiness (*Nidradhikya*) Bodyache*(Angamarda)*

0

0

Indigestion *(Avipaka)* Tastelessness *(Aruchi)* Anorexia *(Anannaabhilaashaa)*

0

0

Loss of appetite *(Agnimaandya)* Headache *(Shirahshoola)* Anosmia *(Gandha)*

0

0

Cough *(Kasa)* Rhinorrhea *(Pratishyaya)* Fever *(Jvara)*

0

0

Breathlessness *(Shvasa)* Headache *(Shirahshool)* Fever with chills (ShitpurvakJvara)

0

0

Sore throat *(Gala Sanrambha)* Dysgeusia *(Aasyavairasya)*

0

Alertness Energetic Cheerful Calm

0

0

Depression Anxiety Forgetfulness Lack of enthusiasm

0

0

Insomnia Early awakening Lightness in head Drowsiness

0

0

Increased appetite Pain in abdomen Hyperacidity Nausea

0

0

Diarrhea Dysentery Constipation Vomiting

0

0

0

Excess salivation Dryness of mouth Mouth ulcers Stomatitis

0

0

Joint pains Muscle pains Stiffness Backache

0

0

Tremors Skin rash Itching Sweating

0

0

1. **Clinical assessment**

1. How many times do you eat every day? --------- times
2. Do you exercise regularly? Yes No ……… times in last week
3. Do you feel sensation of hunger? Yes No Unable to answer
4. How was your sleep yesterday? Sound sleep Disturbed sleep Unable to answer
5. Is your tongue quoted? Yes No Unable to answer
6. Please describe your bowel movements: Constipation Regular Irregular
7. How is your urine? Dark Yellow Pale-Yellow Colorless Burning

1. **Ayurvedic Disease Subtypes / Stages**

Vyadhi: Vata Kaphaj Jvara / Other______________________________________­­­­­­_________

Vyadhi Sankara / Upadrava: _____________________________

Dosha Status: Vata Pitta Kapha Sannipatik / ­­___________

Avastha: Sama / Nirama

Dushya: Rasa Rakta Mamsa

Meda Asthi Majja Shukra

Adhishthan: Pranavaha Annavaha Rasavaha Raktavaha

Other_____­­­­­­­­­­­­­­­­­­­­­­­­­­­­­­­­­­­­____________________________________________________

1. **Vyadhibala:** Heena Madyama Uttam
2. **Rugnabala:** Heena Madyama Uttam
3. **Ancillary measures:**

Diet related: _______________________________________________________________

Behavioral changes: _________________________________________________________

**ASSESSMENT AT FOLLOW UP**

1. **Symptoms at follow up**

Heaviness *(Gaurava)* Weakness *(Daurbalya)* Fatigue *(Klama)*

0

0

Vertigo *(Bhrama)* Sleepiness (*Nidradhikya*) Bodyache*(Angamarda)*

0

0

Indigestion *(Avipaka)* Tastelessness *(Aruchi)* Anorexia *(Anannaabhilaashaa)*

0

0

Loss of appetite *(Agnimaandya)* Headache *(Shirahshoola)* Anosmia *(Gandha)*

0

0

Cough *(Kasa)* Rhinorrhea *(Pratishyaya)* Fever *(Jvara)*

0

0

Breathlessness *(Shvasa)* Headache *(Shirahshool)* Fever with chills ShitapurvakJvara)

0

0

Sore throat *(Gala Sanrambha)* Dysgeusia *(Aasyavairasya)*

0

Alertness Energetic Cheerful Calm

0

0

Depression Anxiety Forgetfulness Lack of enthusiasm

0

0

Insomnia Early awakening Lightness in head Drowsiness

0

0

Increased appetite Pain in abdomen Hyperacidity Nausea

0

0

Diarrhea Dysentery Constipation Vomiting

0

0

0

Excess salivation Dryness of mouth Mouth ulcers Stomatitis

0

0

Joint pains Muscle pains General stiffness Backache

0

0

Tremors Skin rash Itching Sweating

0

0

1. **Clinical assessment**

1. How many times do you eat every day? --------- times
2. Do you exercise regularly? Yes No ……… times in last week
3. Do you feel sensation of hunger? Yes No Unable to answer
4. How was your sleep yesterday? Sound sleep Disturbed sleep Unable to answer
5. Is your tongue quoted? Yes No Unable to answer
6. Please describe your bowel movements: Constipation Regular Irregular
7. How is your urine? Yellow Pale-Yellow Colorless Burning

1. **Ayurvedic Disease Subtypes / Stages**

Vyadhi: Vata Kaphaj Jvara / Other______________________________________­­­­­­_________

Vyadhi Sankara / Upadrava: _____________________________

Dosha Status: Vata Pitta Kapha Sannipatik / ­­___________

Avastha: Sama / Nirama

Dushya: Rasa Rakta Mamsa

Meda Asthi Majja Shukra

Adhishthan: Pranavaha Annavaha Rasavaha Raktavaha

Other_____­­­­­­­­­­­­­­­­­­­­­­­­­­­­­­­­­­­­____________________________________________________

1. **Vyadhibala:** Heena Madyama Uttam
2. **Rugnabala:** Heena Madyama Uttam
3. **Ancillary measures:**

Diet related: _______________________________________________________________

Behavioral changes: _________________________________________________________

## Appendix J: Daily Progress Report

**AYUSH COVID-19 PATIENT PROTOCOL 2020**

**DAILY PROGRESS REPORT:**

**Patient Number______________________ Patient Initials:__________________ Date of Admission______________________________**

| **SYMPTOMS** | **D1** | **D2** | **D3** | **D4** | **D5** | **D6** | **D7** | **D8** | **D9** | **D10** | **D11** | **D12** | **D13** | **D14** | **D15** | **D16** | **D17** | **D18** | **D19** | **D20** |
| --- | --- | --- | --- | --- | --- | --- | --- | --- | --- | --- | --- | --- | --- | --- | --- | --- | --- | --- | --- | --- |
| **FEVER** |  |  |  |  |  |  |  |  |  |  |  |  |  |  |  |  |  |  |  |  |
| **COUGH** |  |  |  |  |  |  |  |  |  |  |  |  |  |  |  |  |  |  |  |  |
| **SORE THROAT** |  |  |  |  |  |  |  |  |  |  |  |  |  |  |  |  |  |  |  |  |
| **DYSPNOEA** |  |  |  |  |  |  |  |  |  |  |  |  |  |  |  |  |  |  |  |  |
| **TASTE LOSS** |  |  |  |  |  |  |  |  |  |  |  |  |  |  |  |  |  |  |  |  |
| **SMELL LOSS** |  |  |  |  |  |  |  |  |  |  |  |  |  |  |  |  |  |  |  |  |
| **MYALGIA** |  |  |  |  |  |  |  |  |  |  |  |  |  |  |  |  |  |  |  |  |
| **CHILLS** |  |  |  |  |  |  |  |  |  |  |  |  |  |  |  |  |  |  |  |  |
| **NAUSEA** |  |  |  |  |  |  |  |  |  |  |  |  |  |  |  |  |  |  |  |  |
| **VOMITING** |  |  |  |  |  |  |  |  |  |  |  |  |  |  |  |  |  |  |  |  |
| **DIARRHOEA** |  |  |  |  |  |  |  |  |  |  |  |  |  |  |  |  |  |  |  |  |
| **PNEUMONIA** |  |  |  |  |  |  |  |  |  |  |  |  |  |  |  |  |  |  |  |  |
|  |  |  |  |  |  |  |  |  |  |  |  |  |  |  |  |  |  |  |  |  |
|  |  |  |  |  |  |  |  |  |  |  |  |  |  |  |  |  |  |  |  |  |

**REMARKS:__________________________________________________________________________________________________________________________________________________________________________________________________________________________________________________**

**AYUSH COVID-19 PATIENT PROTOCOL 2020**

**Patient Number______________________ Patient Initials:__________________ Date of Admission______________________________**

| **VITALS** | **D1** | **D2** | **D3** | **D4** | **D5** | **D6** | **D7** | **D8** | **D9** | **D10** | **D11** | **D12** | **D13** | **D14** | **D15** | **D16** | **D17** | **D18** | **D19** | **D20** |
| --- | --- | --- | --- | --- | --- | --- | --- | --- | --- | --- | --- | --- | --- | --- | --- | --- | --- | --- | --- | --- |
| **PULSE** |  |  |  |  |  |  |  |  |  |  |  |  |  |  |  |  |  |  |  |  |
| **BP** |  |  |  |  |  |  |  |  |  |  |  |  |  |  |  |  |  |  |  |  |
| **RR** |  |  |  |  |  |  |  |  |  |  |  |  |  |  |  |  |  |  |  |  |
| **SPaO2 (OXI)** |  |  |  |  |  |  |  |  |  |  |  |  |  |  |  |  |  |  |  |  |
| **COMPLICATIONS** | | | | | | | | | | | | | | | | | | | | |
| **OXYGEN** |  |  |  |  |  |  |  |  |  |  |  |  |  |  |  |  |  |  |  |  |
| **INTUBATION** |  |  |  |  |  |  |  |  |  |  |  |  |  |  |  |  |  |  |  |  |
| **VENTILATOR** |  |  |  |  |  |  |  |  |  |  |  |  |  |  |  |  |  |  |  |  |
| **ICU** |  |  |  |  |  |  |  |  |  |  |  |  |  |  |  |  |  |  |  |  |
| **STEROID** |  |  |  |  |  |  |  |  |  |  |  |  |  |  |  |  |  |  |  |  |
| **ARF** |  |  |  |  |  |  |  |  |  |  |  |  |  |  |  |  |  |  |  |  |
| **ARDS** |  |  |  |  |  |  |  |  |  |  |  |  |  |  |  |  |  |  |  |  |
| **SHOCK** |  |  |  |  |  |  |  |  |  |  |  |  |  |  |  |  |  |  |  |  |
| **SEPTICEMIA** |  |  |  |  |  |  |  |  |  |  |  |  |  |  |  |  |  |  |  |  |
|  |  |  |  |  |  |  |  |  |  |  |  |  |  |  |  |  |  |  |  |  |

**REMARKS____________________________________________________________________________________________________________________**

## Appendix K: Study Completion and Outcome Report

**AYUSH COVID-19 PATIENT PROTOCOL 2020**

**STUDY COMPLETION AND OUTCOME REPORT**

Id:______________

PATIENT NO: ________________ PATIENT INITIALS:______________________ DATE:____________________

VITAL SIGNS:

Temp: ________ Pulse rate: _______Respiratory Rate : _______Blood Pressure: ________mm Hg

Physical Examination (Please tick):

|  | Normal | Abnormal | If Abnormal, please comment |
| --- | --- | --- | --- |
| General Appearance | **□** | **□** |  |
| Skin | **□** | **□** |  |
| Head, Neck & Thyroid | **□** | **□** |  |
| Eyes | **□** | **□** |  |
| Nose and Throat | **□** | **□** |  |
| Lymph Nodes | **□** | **□** |  |
| Cardiovascular | **□** | **□** |  |
| Lungs | **□** | **□** |  |
| Abdomen | **□** | **□** |  |
| Central Nervous System | **□** | **□** |  |
| Others – Specify | **□** | **□** |  |
|  | **□** | **□** |  |

Patient Patient’s Global Assessment of Tolerability to Therapy (4 grades):

□ Excellent tolerability

□ Good Tolerability

□ Average tolerability

□ Poor tolerability

**PROBABLE OUTCOMES:**

□ Infection free

COVID-19 TEST:□NEGATIVE □POSITIVE

CHANGE IN DISEASE STATE: □MILD TO MODERATE; □MODERATE TO SEVERE

BODY TEMPERATURE: NORMAL □YES □NO

COUGH :□MILD □ ABSENT

BREATHLESSNESS: □MILD □ABSENT

SpO2 NORMAL □YES □NO

SHIFTED TO ICU: □YES □NO

□Withdrawal (Non- COVID 19 related)

□DEATH

□Others _____________________________________________________________________

Observation ends 12 weeks after the start of investigational product **□ Yes □ No**

If ‘No’ indicate the Date /Visit No. of early cessation of the study :___________________

**Therapy Interruptions:**

Therapy with the investigational drug may be interrupted when adverse events occur.

□ Yes □ No

If answered ‘Yes’ please give duration of therapy interruptions.

Therapy Interruptions:

From : __________________ to _____________________

From : __________________ to _____________________

**Reason for early cessation of study :**

Therapy is to be discontinued : [ Please tick √ as appropriate]

□ Therapy refusal/ if a patient withdraws his/her consent.

□ If treatment fails to achieve the desired effect and the investigator considers another therapy to be more effective for medical reasons

□ Significant protocol deviation *

□ Serious adverse event

□ Significant Laboratory abnormality in the opinion of the investigator

□Loss to follow-up

□ Poor compliance*

□ Pregnancy

□ Intercurrent medical problem*

□ Death

* Please clarify in remarks

#### In the event of study discontinuation Comments

(Please specify in detail under remark)

_____________________________________________________________________________

Date of the latest treatment under the study _____________________

Date and time of the latest contact between

Investigator and patient ______________________

________________________________________________________________________________

Remarks of the Investigator: ________________________________________________________________________________

Signature of investigator with date : __________________________________________________

## Appendix N: Self-Reported Questionnaire

**AYUSH COVID-19 PATIENT PROTOCOL 2020**

**SELF REPORTED QUESTIONNAIRE**

PATIENT NO: ________________PATIENT INITIALS :_____________DATE:____________________

1. Do you feel Feverish? □ Yes □ No
2. Do You have sore throat □Yes □No
3. Do you have Nasal stuffiness/Sneezing/Runny Nose? □Yes □No
4. Do You have Cough □Yes □No
5. Did you Suffer from any symptom that could be due to the study drug □Yes □No

If Yes, ____________________________________________________________________

________________________________________________________________________

## Appendix M: WHO Quality of Life Instrument

**AYUSH COVID-19 PATIENT PROTOCOL 2020**

**WHO- Quality of Life Instrument**

**WHOQOL-BREF**

PATIENT NO: ________________PATIENT INITIALS :_____________DATE:__________

Before you begin we would like to ask you a few general questions about yourself: By circling the correct answer or by filling in the space provided.

What is your **gender** ? Male Female

What is you date of **birth**? _______/_______/_______

Day / Month / Year

What is the highest **education** you received ? None at all

Primary school

Secondary school

Tertiary

What is your marital status ? Single Separated

Married Divorced

Living as married Widowed

Are you currently ill ? Yes No

If something is wrong with your health what do you think it is? ________________ illness. Problem

**Instructions**

This assessment asks how you feel about your quality of life. Health, or other areas of your life. **Please answer all the questions.** If you are unsure about which response to give, **please choose the one** that appears most appropriate. This can often be your first response.

Please keep in mind your standard, hopes, pleasures and concerns. We ask what you think about life in **the last two weeks.** Please read each question, asses your feeling, and circle the number on the scale for each question that gives the best answer for you.

|  | |  | **Very poor** | **Poor** | **Neither poor or good** | **Good** | **Very good** |
| --- | --- | --- | --- | --- | --- | --- | --- |
| 1(G1) | | How would you rate your quality of life? |  |  |  |  |  |
|  | |  |  |  |  |  |  |
|  | |  | **Very dissatisfied** | **dissatisfied** | **Neither satisfied nor dissatisfied** | **Satisfied** | **Very satisfied** |
| 2(G4) | | How satisfied are you with your health ? |  |  |  |  |  |
|  | | | | | | | |
| The following questions ask about how much you have experienced certain things in the last two weeks. | | | | | | | |
|  | |  | **Not at all** | **A little** | **A moderate amount** | **Very much** | **As extreme amount** |
| 3(F1.4) | | To what extent do you feel the physical pain prevents you from doing what you need to do ? |  |  |  |  |  |
| 4( F11.3) | | How much do you need any medical treatment to function in your daily life ? |  |  |  |  |  |
| 5(F4.1) | | How much do you enjoy life ? |  |  |  |  |  |
| 6(F24.2) | | To what extent do you feel your life to be meaningful? |  |  |  |  |  |
|  | |  |  |  |  |  |  |
|  | |  | **Not at all** | **A little** | **A moderate amount** | **Very much** | **Extremely** |
| 7(F5.3) | | How well are you able to concentrate ? |  |  |  |  |  |
| 8(F16.1) | | How safe do you feel in your daily life? |  |  |  |  |  |
| 9(F22.1) | | How healthy is your physical environment ? |  |  |  |  |  |
| The following question ask about how completely you experience or were able to do certain things in the last two weeks. | | | | | | | |
|  |  | | **Not at all** | **A little** | **Moderately** | **Mostly** | **Completely** |
| 10(F2.1) | Do you have enough energy for everyday life ? | |  |  |  |  |  |
| 11(F7.1) | Are you able to accept your bodily appearance ? | |  |  |  |  |  |
| 12(F18.1) | Have you enough money to meet your needs? | |  |  |  |  |  |
| 13(F20.1) | How available to you is the information that you need in your day-to-day life | |  |  |  |  |  |
| 14(F21.1) | To what extent do you have the opportunity for leisure activities ? | |  |  |  |  |  |
|  |  | |  |  |  |  |  |
|  | | | | | | | |
|  |  | | **Very poor** | **Poor** | **Neither poor nor good** | **Good** | **Very good** |
| 15(F9.1) | How well are you able to get around ? | |  |  |  |  |  |
| The following question ask to you say how good or satisfied you have felt about various aspects of your life over the last two weeks | | | | | | | |
|  | |  | **Very dissatisfied** | **Dissatisfied** | **Neither satisfied nor dissatisfied** | **Satisfied** | **Very satisfied** |
| 16(F3.3) | | How satisfied are you with your sleep |  |  |  |  |  |
| 17(F10.3) | | How satisfied are you with your ablity to perform daily living activities ? |  |  |  |  |  |
| 18(F12.4) | | How satisfied are you with your capacity for work ? |  |  |  |  |  |
| 19(F6.3) | | How satisfied are you with yourself? |  |  |  |  |  |
| 20(F13.3) | | How satisfied are you with your personal relationships? |  |  |  |  |  |
| 21(F15.3) | | How satisfied are you with your sex life? |  |  |  |  |  |
| 22(F14.4) | | How satisfied are you with the support you get from your friends ? |  |  |  |  |  |
| 23(F17.3) | | How satisfied are you with the conditions of your living place? |  |  |  |  |  |
| 24(F19.3) | | How satisfied are you with your access to health services ? |  |  |  |  |  |
| 25(F23.3) | | How satisfied are you with your transport? |  |  |  |  |  |
|  | |  | **Never** | **Seldom** | **Quite often** | **Very often** | **Always** |
| 26(F8.1) | | How often do you have negative feelings such as blue mood, despair, anxiety, depression? |  |  |  |  |  |

Did someone help you to fill out this form ? ___________________________________________

How long did it take to fill this form out? _____________________________________________

Do you have any comments about the assessment ?______________________________________

## Appendix N: Health Related - Behavior Habit Fitness

**AYUSH COVID-19 PATIENT PROTOCOL 2020**

**HEALTH RELATED - BEHAVIOUR HABIT FITNESS**

**(HR-BHF)**

PATIENT NO: ________________PATIENT INITIALS :_____________DATE:__________

**Please put a vertical line across the scale to indicate your extent of the health related problem**

1. **Please consider the past ONE WEEK while answering. We want you to give your overall assessment.**
2. GENERAL HEALTH: How would you rate your overall general health?

100

0

Very Poor Very Good

1. How much is your anxiety ?

100

0

Very anxious No anxiety at all

1. Fatigue: How much do you feel tired in your daily routine?

100

0

Nil Feel very tired

1. How much is your Energy level?

100

0

Low energy Feel Energetic during my daily routine

1. How is your Bowel movement (passing stools/faeces)?

100

0

Highly irregular and unsatisfying Satisfactory and normal bowel habit

1. Are you stressed?

100

0

No Stress Maximum Stress

1. How happy are you?

100

0

Very Sad Very Happy

1. How do you rate your sleep?

100

0

Normal Bad and unable to sleep properly

1. How much is your appetite for food?

100

0

Nil Very Good

**THANK YOU FOR YOUR PARTICIPATION**

## Appendix O: Laboratory Diagnosis and Cytokine Assay

**LABORATORY DIAGNOSIS AND CYTOKINE ASSAY**

**The following laboratory investigations are proposed to be carried out as per the protocol on prophylaxis against COVID-19 in high risk health care providers and other risk prone individuals in the community**:

1. Diagnostic: nose/throat swab to detect COVID 19 using RT-PCR
2. Serology: specific anti-COVID IgM (ELISA) and IgG antibodies (chromatography)
3. Cytokine Assay: gamma Interferon, anti-TNF apha & beta, IL-6, IL-4, IL-13, IL-17, MCP
4. Routine Laboratory tests: detailed in protocol and in a separate Appendix

**Laboratory Diagnosis of Covid-19:**

Specific diagnosis of Covid-19 is done by specific molecular tests to determine one or several nucleic acid targets specific to SARS–CoV-2on respiratory samples (throat swab/ nasopharyngeal swab/ sputum/ endotracheal aspirates and bronchoalveolar lavage. The virus may also be detected in the stool and in the blood in severe cases. The tests that are currently used for diagnosis of SARS–CoV-2 identify viral RNA through nucleic acid amplification, usually using real time Reverse Transcriptase - Polymerase Chain Reaction (rRT-PCR) (1). Routine confirmation of cases of COVID-19 is based on detection of unique sequences of virus RNA by Nucleic Acid Amplification Test (NAAT) such as rRT-PCR with confirmation by nucleic acid sequencing when necessary. The viral genes targeted so far include the N, E, S and RdRP genes. The most common samples that are being tested are swabs taken from the nasopharynx and/or oropharynx. Nasopharangeal swabs are considered somewhat more sensitive than those taken from the oropharynx. (2). In case both are collected, the two swabs may be combined and tested to increase viral RNA extraction. Swabs are transported in universal viral transport medium immediately after sample collection to preserve viral nucleic acid. Samples taken from sputum, endotracheal aspirates, and bronchoalveolar lavage may have greater sensitivity than upper respiratory tract specimens (3). Inadequate sample collection may result in a false-negative test. All samples undergo RNA extraction followed by qualitative RT-PCR for target detection.The ability of a negative RT-PCR assays to rule out COVID-19 on the basis of upper respiratory tract samples obtained at a single time point remains unclear. Conversely, if a patient has had a positive test result, it is recommended obtaining at least 2 negative upper respiratory tract samples, collected at intervals of 24 hours or longer, to document SARS–CoV-2 clearance (4).

Indian Council for Medical Research – National Institute of Virology (ICMR-NIV) has completed evaluation of 20 non- US FDA EUA/CE IVD kits.

As per ICMR guidelines, currently first line screening for COVID-19 is being done using E gene Assay. All positive samples are sent to the reference laboratory (ICMR-NIV, Pune) for confirmatory testing. It is only after confirmatory test comes positive, that the sample is declared positive. Confirmatory assays available currently at ICMR-NIV are:

- ORF 1b
- RdRp gene assay
- E gene assay
- N gene assay

**Serology:**

Testing for different immunoglobulin (Ig) subclasses is common in serodiagnostics. As antibodies of IgM subclass are usually the first to develop following a primary challenge, IgM is considered to be a parameter of the early phase of infection. Serologic tests that identify antibodies (such as IgA, IgM, and IgG) to SARS–CoV-2 from clinical specimens such as whole blood, serum or plasma such as enzyme-linked immunosorbent assays, are less complex than molecular tests and have the potential to be used for diagnosis in certain situations especially when combined IgM ELISA assay with PCR for each patient compare with a single quantitative PCR(5). However, their utility for diagnosing acute infections is probably limited around the time of symptom onset, because the viral shedding and transmission risk seem to be highest at the pre-symptomatic or symptomatic stage (2). Antibody responses to infection may take a few days to weeks to be reliably detectable (5). Negative results would not exclude SARS–CoV-2 infection, particularly among those with recent exposure to the virus (6).

Serological surveys can aid investigation of an ongoing outbreak and retrospective assessment of the attack rate or extent of an outbreak. In cases where NAAT assays are negative and there is a strong epidemiological link to COVID-19 infection, paired serum samples (in the acute and convalescent phase) could support diagnosis once validated serology tests are available. Serum samples can be stored for these purposes. Also, rapid screening of SARS-CoV-2 carriers who are symptomatic or asymptomatic, in hospitals, clinics and diagnostic laboratories can be done using IgM-IgG combined assays that has shown relatively good specificity and sensitivity (7). Serology detection is also likely to play a role in determining the immunity of health care workers as the outbreak progresses.

**Cytokines:**

RATIONALE FOR CYTOKINE RESEARCH: Cytokine assay can be used to study important immune events of anti-viral (gamma interferon), pro-inflammatory (IL-6, anti-TNF, IL-17), TH 1 and TH 2 response and B cell activity (Il 4, IL 13), macrophage activation (MCP). Intense up regulation and elevation of IL 6 has been extensively reported by several clinical case series and research and a cause of ‘cytokine storm’ that often predates progressive severity and mortality. Other cytokines are also reported to be elevated. All these events are important in the pathophysiology of COVID 19 and may predate the onset of clinical infection. Also, it is important to know the baseline assay of these cytokines in healthy population. There is a dire need to generate normative data in the Indian population and this can be done in this research protocol. Without the latter, it may be difficult to evaluate modest elevations of cytokines. Although this is a protocol for prophylaxis, several patients may show elevations due to basal environmental conditions, asymptomatic COVID 19 infection, and pre-infection up regulated innate immune response. Also, it is expected that some study participants will contract COVID 19 infection and go on to develop the illness and require medical management. It may be useful to know the pre-illness cytokine status of these individuals and this may also be repeated during the illness to evaluate the immune response.

Cytokines play an important role in immunopathology of viral infections in which a rapid and coordinated innate immune response is often seen as the first line of defense against viral infection. However, dysregulated and excessive immune responses may cause immune imbalance leading to severe systemic inflammatory disorders. Pro-inflammatory responses have reported to play an important role in the pathogenesis of several viral infections (8) including Human Coronavirus (HCoVs)(9). A cytokine profile resembling secondary hemophagocyticlymphohistiocytosis (sHLH) is reported to be associated with COVID-19 disease severity, characterised by increased levels of cytokines and chemokines such as interleukin (IL)-2, IL-7, granulocytecolony stimulating factor, interferon-γ inducible protein 10, monocyte chemoattractant protein 1, macrophage inflammatory protein 1-α, and tumour necrosis factor-α (10). Elevated ferritin levels along with elevated interleukin-6 levels have been reported as significant predictors of fatality in a retrospective, multicentre study of 150 confirmed COVID-19 cases in Wuhan, China, suggesting that mortality might be due to virally driven hyperinflammation (11).High levels of expression of IL-1B, IFN-γ, IP-10, and monocyte chemoattractant protein 1 (MCP-1) have been reported in patients with COVID-19 (10). Positive correlation between serum levels of IL-2R and IL-6 (Cytokine storm) and severity of diseases has been seen in patients with COVID-19 (12). In COVID-19, the inflammatory cytokine storm is closely related to the development and progression of Acute Respiratory Distress Syndrome.

Cytokines can be estimated in the serum of patients by several assays such as ELISA or multiplex assays.  Advances in laboratory technologies including flow cytometrics, Luminex bead-based assays, planar multiplex assays. Although the multiplex platforms and ELISA-based systems exhibit distinct strengths and weaknesses, conventional quantitative ELISA assays are very convenient in clinical settings. Several sandwich ELISA assays using monoclonal antibodies against various cytokines are commercially available wherein the microwell plates are coated with monoclonal antibodies against the cytokine to be estimated in the serum.

**Covid-19 tests, times & utility.**

| **Type of Test** | **When to test** | **Whom to test** | **Action Required if tested positive** |
| --- | --- | --- | --- |
| Nucleic Acid Amplification Test (NAAT) by rRT-PCR | Viral load peaks at the end of first week after infection, just before and as symptoms are developing. It may be intermittent, so a single negative swab result can be misleading and tests may need to be repeated | Individuals with suspected current infection with SARS-CoV-2 | Quarantine to prevent transmission and monitor course of illness |
| Antibody detection (IgM and/or IgG by rapid lateral chromatography methods | Specific IgM antibodies start to become detectable after 4-5 days, with positive IgM in ~70% of symptomatic patients by 8-14 days and ~90% of total antibody tests positive by 11-24 days.  IgG reactivity is thought to reach >98% after several weeks, but duration of the response of this antibody is not yet know. | Individuals suspected with recent exposure to SARS-CoV-2 in arRT-PCR negative test result and who is asymptomatic. | Facilitate contact tracing & surveillance.  Identify those potentially immune to SARS-CoV-2. |

References

1. Corman VM, Landt O, Kaiser M, Molenkamp R, Meijer A, Chu DKW, et al**.** Detection of 2019 novel coronavirus (2019-nCoV) by real-time RT-PCR. Euro Surveill. 2020;25. [PMID: 31992387] doi:10.2807/1560-7917.ES.2020.25.3.2000045.
2. Zou L, Ruan F, Huang M, Liang L, Huang H, Hong Z, Yu J, Kang M, Song Y, Xia J, Guo Q, Song T, He J, Yen H-L, Peiris M, Wu J. 2020. SARS-CoV-2 viral load in upper respiratory specimens of infected patients. N Engl J Med 382:1177–1179. doi:10.1056/NEJMc2001737.
3. **Wang W, Xu Y, Gao R, Lu R, Han K, Wu G, Tan W.** Detection of SARS-CoV-2 in different types of clinical specimens. JAMA. 2020. [PMID: 32159775] doi:10.1001/jama.2020.3786
4. **European Centre for Disease Prevention and Control.** Novel coronavirus (SARS-CoV-). Accessedat <https://www.ecdc.europa.eu/sites/default/files/documents/COVID-19-Discharge-criteria.pdf>. Last accessed 14 April 2020.
5. Guo L, Ren L, Yang S, Xiao M, Chang, Yang F, et al. Profiling early humoral response to diagnose novel coronavirus disease (COVID-19). Clin Infect Dis. 2020. [PMID: 32198501] doi:10.1093/cid/ciaa310.
6. Matthew P. Cheng, Jesse Papenburg ,Michaël Desjardins,SanjatKanjilal, Caroline Quach, Michael Libman, Sabine Dittrich,Cedric P. Yansouni*.* Diagnostic Testing for Severe Acute Respiratory Syndrome–Related Coronavirus-2: A Narrative Review. Ann Intern Med. 2020; [Epub ahead of print 13 April 2020]. doi: <https://doi.org/10.7326/M20-1301>
7. Zhengtu Li, Yongxiang Yi, XiaomeiLuo, NianXiong, Yang Liu, ShaoqiangLiet al. J Med Virol 2020. Doi: 10.1002/jmv.25727. Online ahead of print.
8. Venugopalan A, Ghorpade R, Chopra A.Cytokines in acute chikungunya. [PLoS One](https://www.ncbi.nlm.nih.gov/pmc/articles/PMC4208842/). 2014; 9(10): e111305.  doi: [10.1371/journal.pone.0111305](https://dx.doi.org/10.1371%2Fjournal.pone.0111305)
9. Qing Ye, Bili Wang, Jianhua Mao. Cytokine Storm in COVID19 and Treatment, Journal of Infection (2020), doi: <https://doi.org/10.1016/j.jinf.2020.03.037>.
10. Huang C, Wang Y, Li X, Ren L, Zhao J, Hu Y, et al. Clinical features of patients infected with 2019 novel coronavirus in Wuhan, China. Lancet 2020; 395: 497–506.
11. Ruan Q, Yang K, Wang W, Jiang L, Song J. Clinical predictors of mortality due to COVID-19 based on an analysis of data of 150 patients from Wuhan, China. Intensive Care Med 2020; published online March 3. DOI:10.1007/s00134-020-05991-x).
12. Chen L, Liu HG, Liu W, Liu J, Liu K, Shang J, et alAnalysis of clinical features of 29 patients with 2019 novel coronavirus pneumonia. Chin J TubercRespir Dis. 2020;43.

## Appendix P: Classification of Adverse Events

**CLASSIFICATION OF ADVERSE EVENTS**

Adverse Events (AE):

All observed or volunteered adverse events regardless of treatment group or suspected causal relationship to study drug will be recorded on the AE Form. All AE’s must be classified according to their severity as Mild, Moderate and Severe.

For all AE, the investigator must pursue and obtain information adequate both to determine the outcome of the adverse event and to assess whether it meets the criteria for classification as a Serious AE.

Follow-up of the adverse event even after the date of therapy discontinuation, is required if the AE or its sequelae persist. Follow-up is required until the event or its sequelae resolve or stabilize at a level acceptable to the Investigator.

*Please note that AE are not limited only to adverse drug reactions. Any noxious event and or a medically important deviations from normality will be recorded as AE.*

Criteria for determining category of relationship of AE to trial treatment will be as follows:

1. Not related: AE’s which are definitely attributed to other causes.

2. Unlikely: (must satisfy at least two)

- It does not follow a reasonable temporal sequence from administration of the test drug
- It could readily have been produced by the patients clinical state, environmental or toxic factors, or other modes of therapy administered to the patient
- It does not follow a known pattern of response to the test drug
- It does not reappear or worsen when the drug is re-administered

3. Possible: (must satisfy at least two criteria) An AE may be considered possibly related if or when

- It follows a temporal sequence from administration of the drug
- It could not readily have been produced by the patient’s clinical state, environmental or toxic factors.
- It follows a known pattern of response to the test drug

4. Probable: (must satisfy three of the following)

- It follows a reasonable temporal sequence from administration of the drug
- It could not be reasonably explained by the known characteristics of the patient’s clinical state, environmental or toxic factors, or other modes of therapy administered to the patient
- It decreases or disappears on cessation or reduction in dose.
- It follows a known pattern of response to the test drug

5. Definitely (must satisfy all four criteria)

- It follows a reasonable temporal sequence from administration of the drug
- It could not be reasonably explained by the known characteristics of the patient’s clinical state, environmental or toxic factors, or other modes of therapy administered to the patient
- It decreases or disappears on cessation or reduction in dose and recurs with re-exposure to drug.
- It follows a known pattern of response to the test drug

f) Serious Adverse events (SAE):

All serious adverse events which occur during the study until the last follow-up visit required by the protocol, regardless of treatment group or suspected relationship to drug, must be reported immediately by telephone \ fax to Principal Investigator***,*** .

SAE include those that suggest a significant hazard, such as events which:

- are fatal
- result in permanent disability
- require in – patient hospitalization or a hospital stay
- are life – threatening
- a congenital anomaly or drug overdose

It should be emphasized that, regardless of the above criteria, any additional adverse experience which the investigator considers serious should be immediately reported.

For all serious adverse events, the investigator is obligated to pursue and provide information as requested, in addition to that on the case record form. In general, this will include a description of the adverse event in sufficient detail to allow for a complete medical assessment. The investigator should ensure that information on such cases is reported by telephone or by other means and information entered in the case record form is accurate and consistent.

## Appendix Q: Adverse Event Form

**YUSH COVID-19 PATIENT PROTOCOL 2020**

**ADVERSE EVENT FORM**

PATIENT NO: ________________PATIENT INITIALS :_____________DATE:____________________

**Adverse Event Term:** _____________________________________________________________

| AE Serious | □ Yes □ No | |
| --- | --- | --- |
| Start Date______________________ End Date ___________________Ongoing□ Yes □ No | | |
| Related to study treatment : Ashwagandha | | □ Yes □ No |
| Related to study treatment – Hydroxychloroquine | | □ Yes □ No |
| Related to study Procedures | | □ Yes □ No |
| Action taken with study treatment : Ashwagandha  Dose not changed □; Drug Interrupted □; Drug Withdrawn□; Not Applicable □ | | |
| Action taken with study treatment: Hydroxychloroquine  Dose not changed □; Drug Interrupted □; Drug Withdrawn□; Not Applicable □ | | |
| Other Action taken :None □; Medication required □; Other Treatment Required □; Permanent Study discontinuation □ Details of treatment/medication: _________________________________________________________________________________ | | |
| AE toxicity Grade: Grade 1 □; Grade 2 □; Grade 3 □; Grade 4 □ | | |

**Appendix R: Grading of Adverse Events**

**GRADING OF ADVERSE EVENTS**

| Grade | Adjective | Description |
| --- | --- | --- |
| Grade 1 | Mild | Asymptomatic or mild symptoms ; clinical or diagnostic observations only ; intervention not indicated. |
| Grade 2 | Moderate | Local or non-invasive intervention indicated; limiting age - appropriate instrumental ADL |
| Grade 3 | Severe | Severe or medically significant but not immediately life - threatening; hospitalization or prolongation of hospitalization indicated; disabling; limiting self -care ADL |
| Grade 4 | Life Threatening | Urgent intervention indicated |
| Grade 5 | Death | Death related AE |

## Appendix S: Patient Information Card

**AYUSH COVID-19 PATIENT PROTOCOL 2020**

**PATIENT INFORMATION CARD**

Patient No _____________ Patient Initials: _____________ ___

**PLEASE CARRY THIS CARD WITH YOU AT ALL TIMES**

THIS PATIENT IS PARTICIPATING IN A CLINICAL TRIAL

A Randomized, Open Label, Parallel Efficacy, Active Control, Multi-Centre Exploratory Drug Trial to Evaluate Efficacy and Safety of an Ayurvedic Formulation as Adjunct Treatment to Standard of Care for the Management of Mild to Moderate COVID-19 patients

***INSTITUTION NAME:__________________________________________________________***

***______________________________________________________________________________***

***INSTITUTION ADDRESS:_____________________________________________________***

***____________________________________________________________________________________________________________________________________________________________***

***EMERGENCY 24 HOUR PHONE :_______________________________________________***

PRESCRIPTION ON DISCHARGE:

## Appendix T: Declartion of Helsinki

**
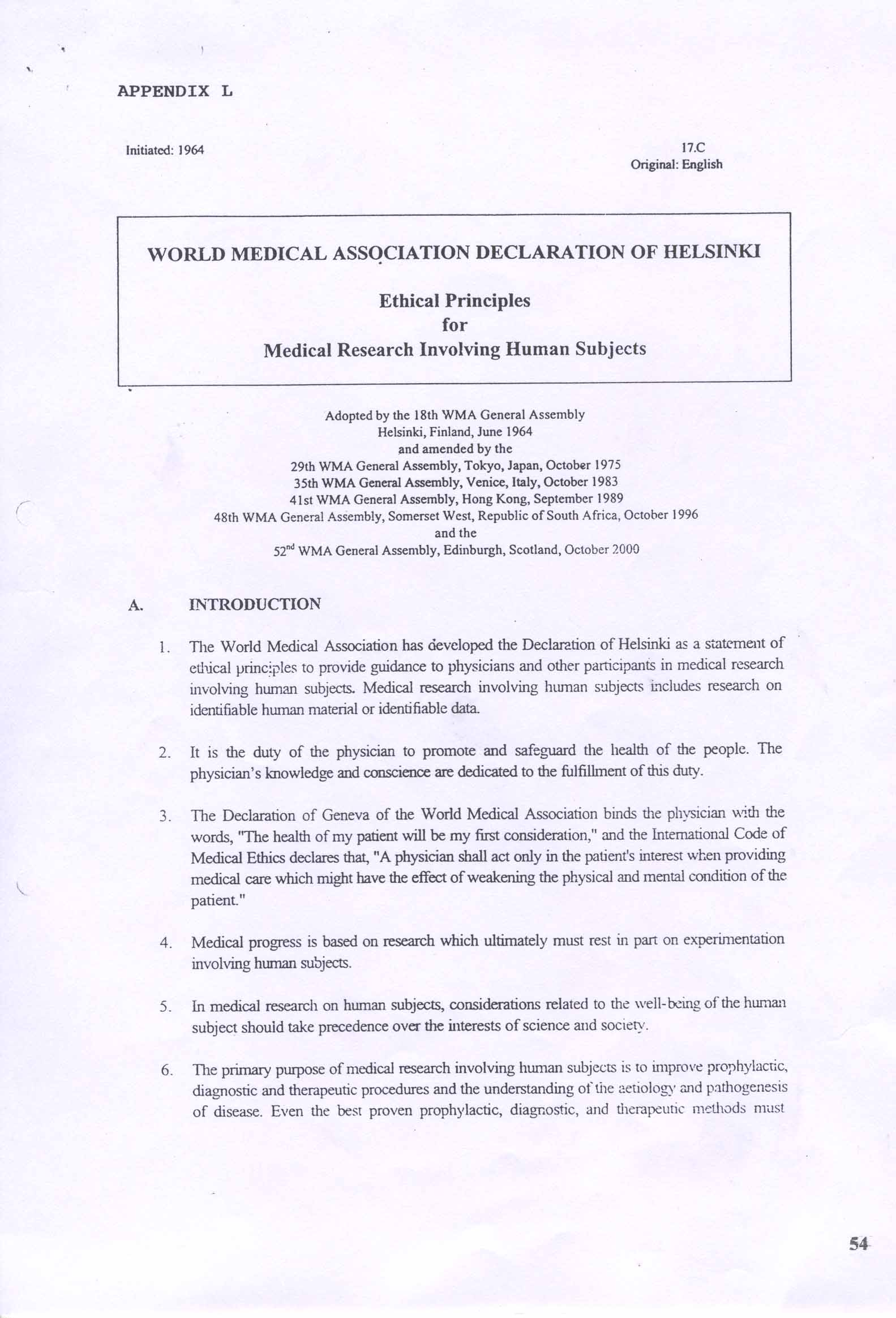
**

**
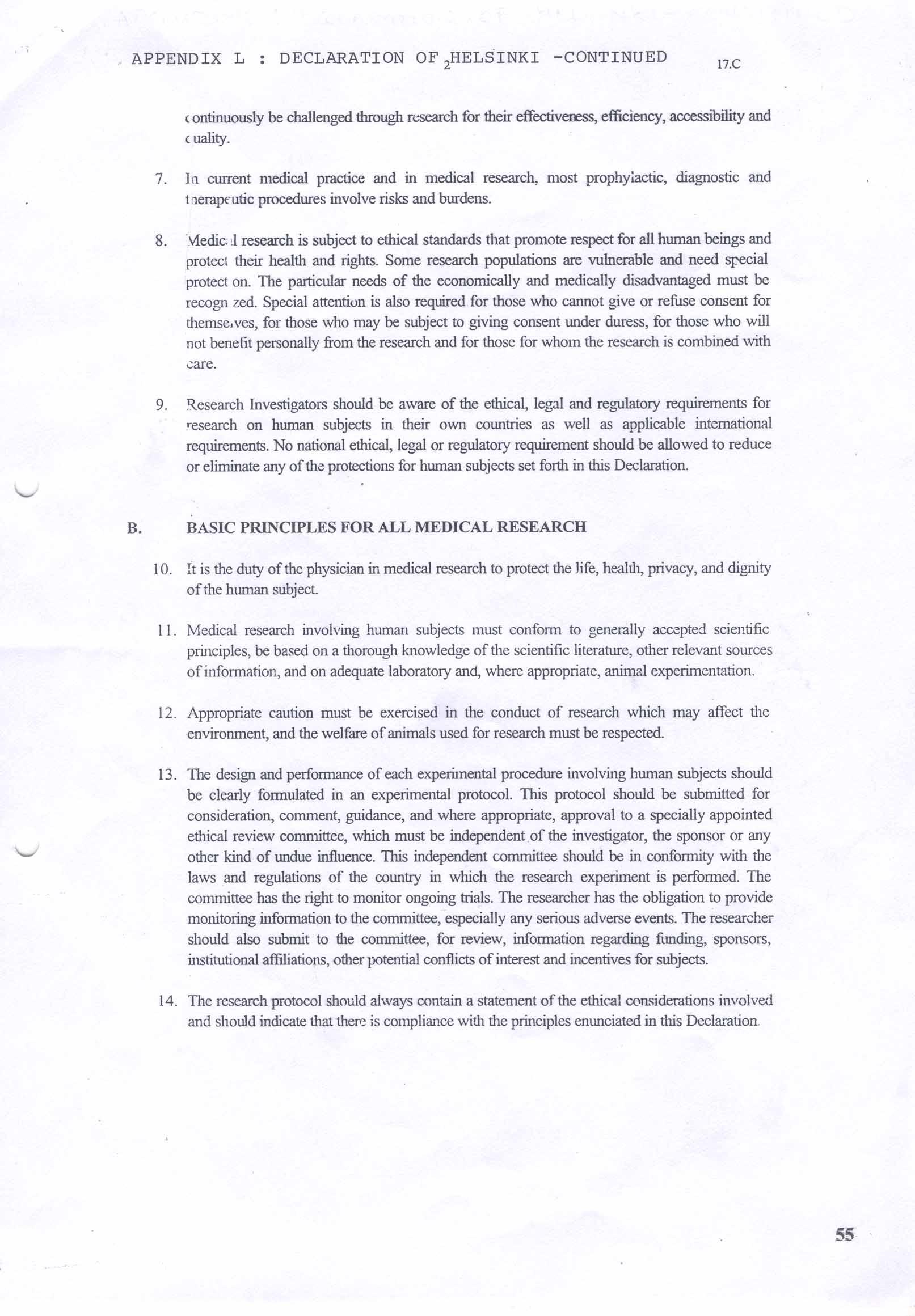
**

**
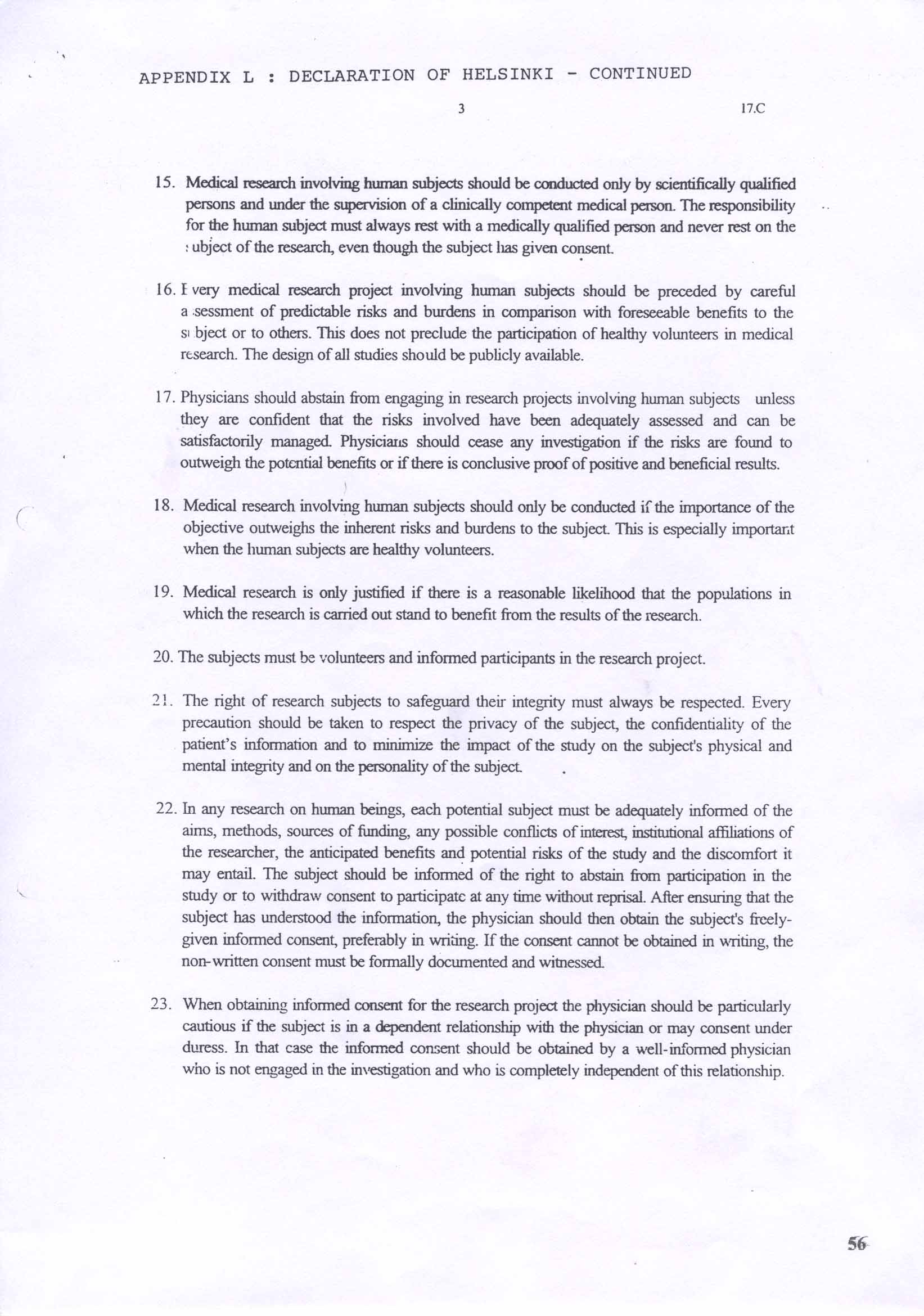
**

**
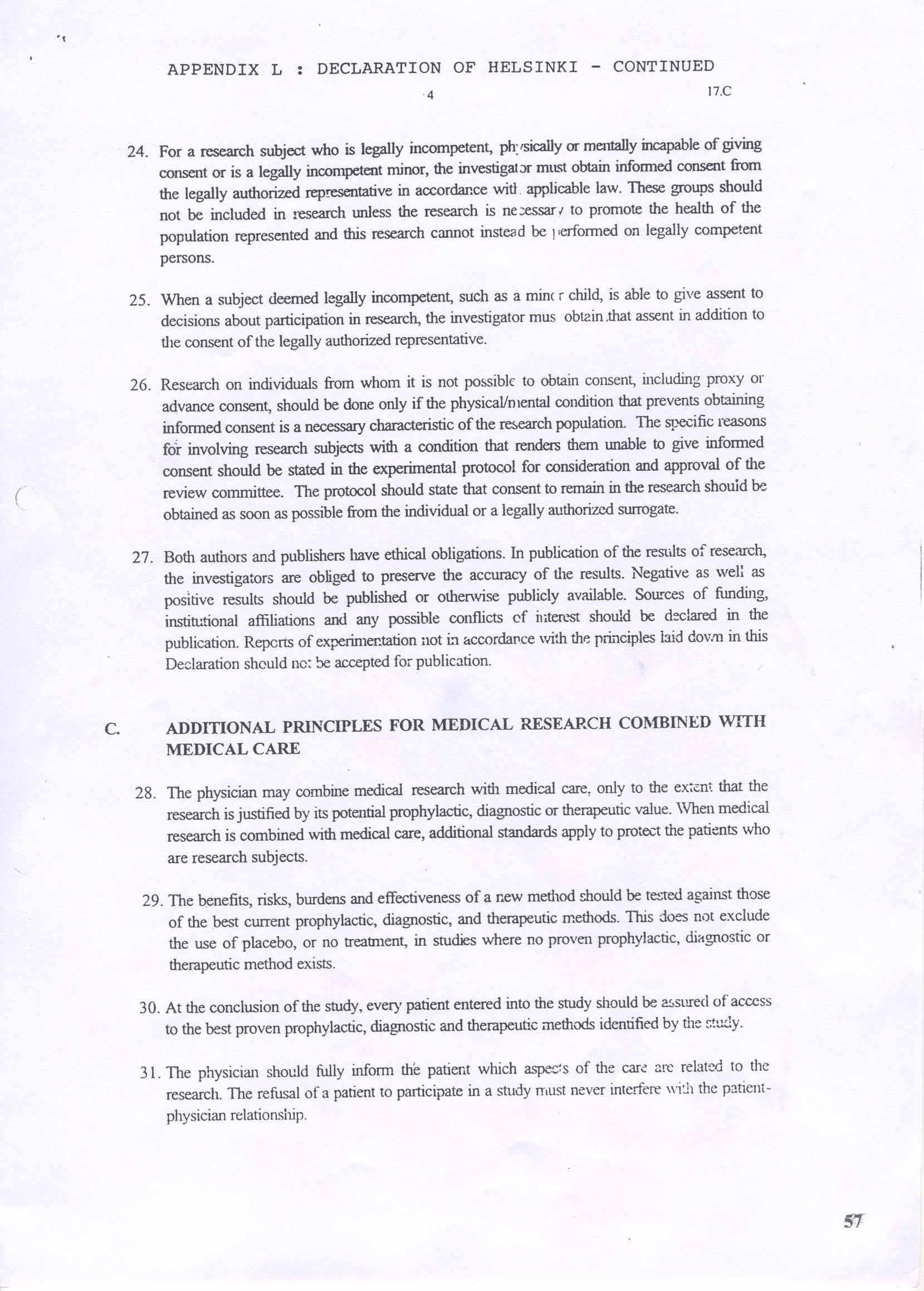
**

**
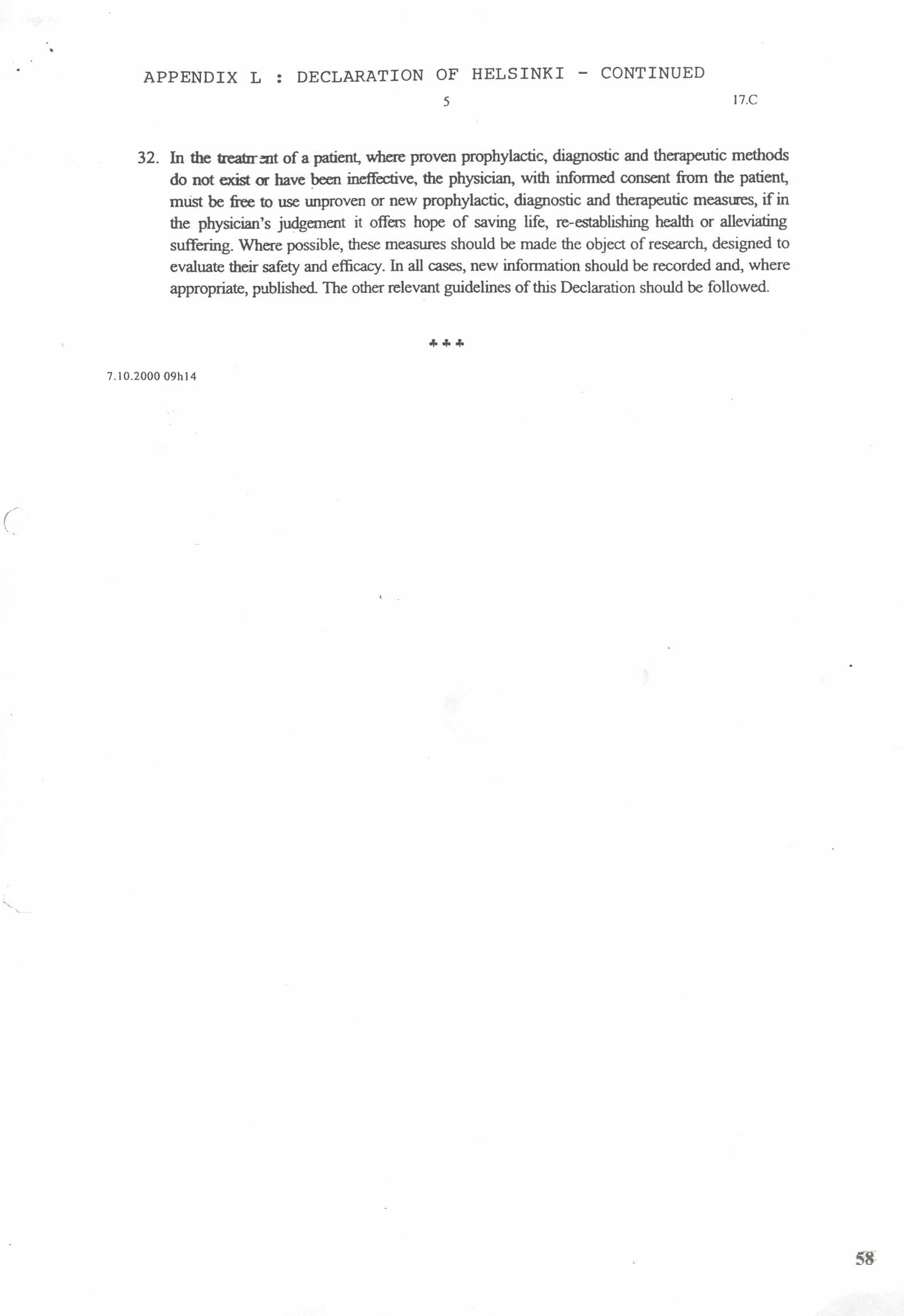
**

## Appendix U: List of Protocol Reviewers

|  | **Reviewer** | **Address** | **Comments** |
| --- | --- | --- | --- |
| **1** | Prof. Gurdip Singh, Padma Shri | M.D. (Ayu), Ph.D., Triple Gold Medalist, Director PG Studies SDM College of Ayurveda, Hassan  Formerly Professor & Dean, Gujarat Ayurveda University Jamnagar | Received |
| **2** | Prof Yogesh Kumar Chawla, Padma Shri | BC Roy Awardee, Former Director, Post Graduate Institute of Medical Education and Research, Chandigarh, India | Received |
| **3** | Prof SK Maulik | Professor, Dept of Pharmacology, All India Institute of Medical Sciences, New Delhi | Received |
| **4** | Prof MUR Naidu | Former Dean, Faculty of Medicine, Professor and Head, Clinical Pharmacology and Therapeutics, The Nizam's Institute of Medical Sciences, Hyderabad, India | Received |
| **5** | Prof MS Baghel | Former Vice Chancellor, Gujarat Ayurveda University, Jamnagar | Received |
| **6** | Prof RN Mishra | Dept of Clinical Immunology, Sanjay Gandhi Post Graduate Institute of Medical Sciences, Lucknow | Received |
| **7** | Dr Rajagopala S | Associate Professor and Head | Received |
| **8** | ICMR Reviewer | Prof. YK Gupta, Former HoD, Pharmacology & Dean AIIMS, Delhi Dr R. Hemlatha, Director, ICMR-NIN, Hyderabad Dr Vishnu Rao, Director, ICMR-NIMS, Delhi Dr Dinesh Kumar, Scientist-G, ICMR-NIN, Hyderabad Dr Vijay Kumar, Scientist G and Head BMS, ICMR  Dr Madhavi Eerike, Scientist D, ICMR Dr Jerin Jose Cherian, Scientist D, ICMR Dr. Monika Pahuja, Scientist C, ICMR | Received |
