# Supplementary material for: Co-administration of AYUSH 64 as an adjunct to standard of care in mild and moderate COVID-19: A randomized, controlled, multicentric clinical trial
Source: PLoS One. 2023 Mar 16;18(3):e0282688. doi: 10.1371/journal.pone.0282688 (PMC10019690; doi:10.1371/journal.pone.0282688)
Supplement: S3 File — (DOCX) [file pone.0282688.s003.docx]

Coadministration of AYUSH 64 as an adjunct to Standard of Care in mild and moderate COVID-19: A randomised, controlled, multicentric clinical trial

**S3 File. Composition, Chemistry, Manufacturing and Controls of AYUSH-64**

**Table S3.1: Composition of AYUSH-64 (standard proprietary Ayurvedic drug); contents of a 500 mg tablet**

| **S.No.** | **Name of the ingredient** | **Botanical name** | **Part used** | **Quantity** |
| --- | --- | --- | --- | --- |
| 1 | Saptaparna (Aqueous extract) | *Alstonia scholaris* | Bark | 100 mg |
| 2 | Kutaki (Aqueous extract) | *Picrorhiza kurroa* | Rhizome | 100 mg |
| 3 | Kiratatikta (Aqueous extract) | *Swertia chirata* | Whole plant | 100 mg |
| 4 | Latakaranja powder | *Caesalpinia crista* | Seed | 200 mg |

**Table S3.2:** Quality Standard (Specifications) of AYUSH-64 (standard proprietary Ayurvedic drug) and its ingredients for QC Analysis of AYUSH-64 and its ingredients

| **Sr** | **Test Parameters** | **Ingredients** | | | | **Formulation** |
| --- | --- | --- | --- | --- | --- | --- |
|  |  | ***Saptaparna* (Aqueous Extract)** | ***Kutaki***  **(Aqueous Extract)** | ***Chiraita***  **(Aqueous Extract)** | ***Latakaranja* (Seed Powder)** |  |
| 1 | Loss on drying | Not more than 9% | Not more than 6% | Not more than 8% | - | Not more than 6% |
| 2 | pH (1% Sol) | 4.5-6.5 | 4.0-7.0 | 5.0-7.0 | - | 4.0-6.5 |
| 3 | Total Ash | Not more than 12% | Not more than 5% | Not more than 15% | Not more than 5% | Not more than 25.0% |
| 4 | Acid insoluble Ash | Not more than 2% | Not more than 1% | Not more than 2% | Not more than 1% | Not more than 8.0% |
| 5 | Alcohol soluble extractive | Not less than 3% | Not less than 3% | Not less than 12% | Not less than 26% | Not less than 5.0 % |
| 6 | Water Soluble extractive | Not less than 85% | Not less than 80% | Not less than 80% | Not less than 4% | Not less than 30.0% |
| 7 | Heavy Metals  (Max. limit) | Lead 10 ppm  Arsenic 3.0 ppm  Mercury 1.0 ppm  Cadmium 0.3 ppm | | | | |
| 8 | Microbial Count | Total Microbial plate count = < 10^5^cfu/gm  Yeast and mould count = <10^3^cfu/gm | | | | |
| 9 | Specific Pathogens | *Escherichia* coli.**-** absent  *Salmonella* spp. *-* absent  *Staphylococcus aureus -* absent  *Pseudomonas -* absent | | | | |
| 10 | Aflatoxins | B1 = Not more than 0.5%  B2= Not more than 0.1 %  G1= Not more than 0.5%  G2= Not more than 0.1 % | | | | |
| 11 | Pesticide Residue | As per Annexure-I | | | | |

Note- Quality control and safety parameters of the ingredients and the formulation are complied with Ayurvedic Pharmacopoeia of India (API) limits/ In-house limits.

Fig S3.1 : HPTLC Chemo Profiling and UV spectrophotometric analysis of Ayush-64 (standard proprietary Ayurvedic drug) & its raw ingredients.

| 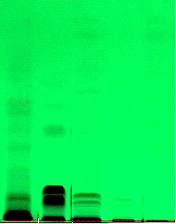 | 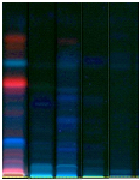 | 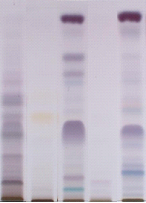 |
| --- | --- | --- |
| 1 2 3 4 5 1 2 3 4 5 1 2 3 4 5  UV 254 UV 366 under white Light after derivatization | | |

Track 1- Saptaparna , Track 2-Katuki , Track 3-Ayush 64, Track 4-Kiratatikta, Track 5-Latakaranja

(b)

| 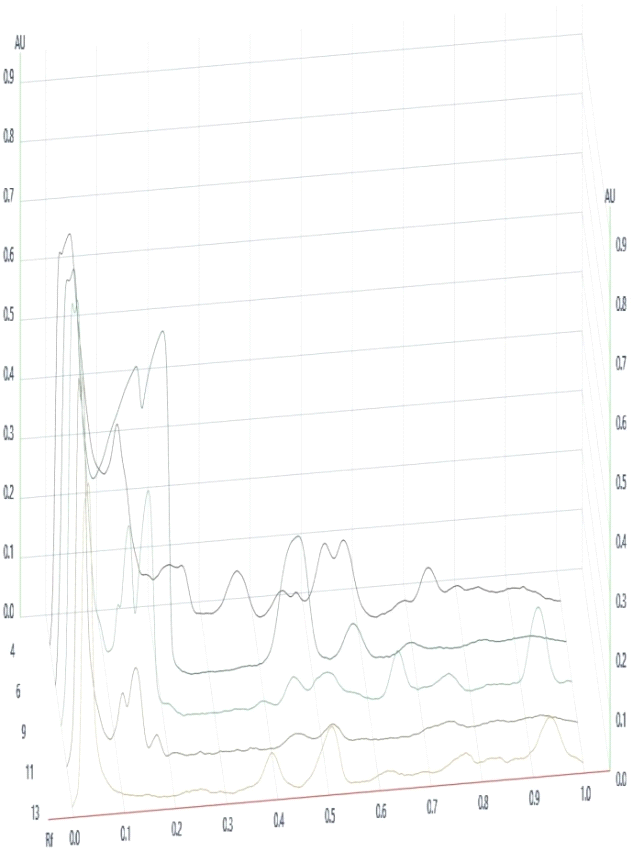 | 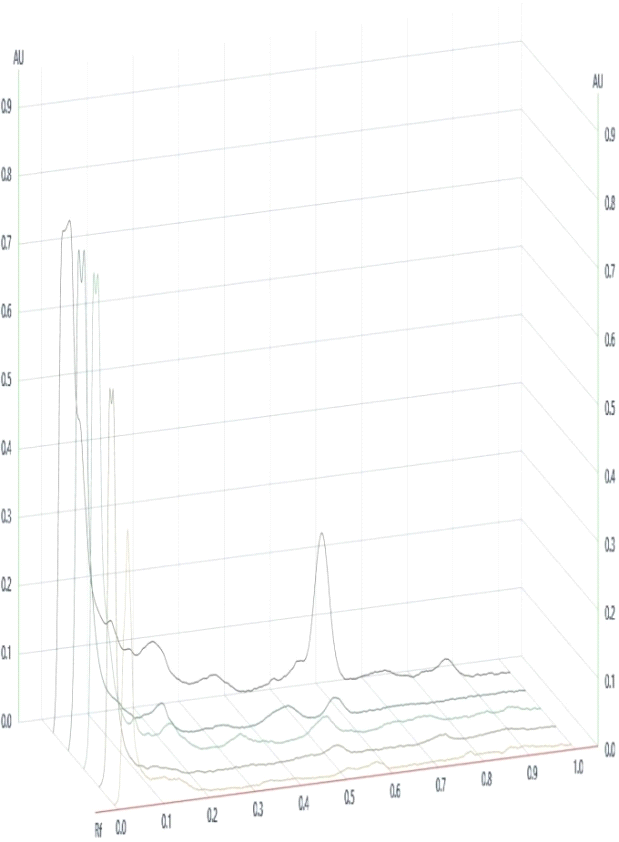 |
| --- | --- |

**3D Densiometric HPTLC Profile under UV 254 nm. 3D Densiometric HPTLC Profile under UV 366 nm.**

**Table S3.3:** Pesticide Residue of ingredients of AYUSH 64 (standard proprietary Ayurvedic drug) as per Indian pharmacopeia

| **Substance (s)** | **Permissible Limit prescribed in API** (in ppm) |
| --- | --- |
| Alachlor | 0.02 |
| Aldrin and Dieldrin (sum of) | 0.05 |
| Azinphos-methyl | 1.0 |
| Bromopropylate | 3.0 |
| Chlordane (sum of cis-,Trans- and Oxythlordane) | 0.05 |
| Chlorfenvinphos | 0.5 |
| Chlorpyrifos | 0.2 |
| Chlorpyrifos-methyl | 0.1 |
| Cypermenthrin (and Isomers) | 1.0 |
| DDT (sum of p, p-DDT, o, p-DDT, p, p-DDE and p, p-TDE) | 1.0 |
| Deltamenthrin | 0.5 |
| Diazinon | 0.5 |
| Dichlorvos | 1.0 |
| Dithiocarbamates (as CS2) | 2.0 |
| Endosulfan (sum of isomers and endosulfansulphate) | 3.0 |
| Endrin | 0.05 |
| Ethion | 2.0 |
| Fenitrothion | 0.5 |
| Fenvalerate | 1.5 |
| Fonofos | 0.05 |
| Heptachlor (sum of Heptachlor and Heptachlorepoxide) | 0.05 |
| Hexachlorbenzene | 0.1 |
| Hexachlorocyclohexane isomers (other than ɤ) | 0.3 |
| Lindane (ɤ- Hexachlorocyclohexane) | 0.6 |
| Malathion | 1.0 |
| Methidathion | 0.2 |
| Parathion | 0.5 |
| Parathion-methyl | 0.2 |
| Permethrin | 1.0 |
| Phosalone | 0.1 |
| PiperonylButoxide | 3.0 |
| Pirimiphos-methyl | 4.0 |
| Pyrethrins (sum of) | 3.0 |
| Quintozene (sum of pentachloroaniline and methyl pentachlorophnylsulphide) | 1.0 |

^$^ Limit of Detection = 10 ppb (GC-MS MS)
