# Supplementary material for: Co-administration of AYUSH 64 as an adjunct to standard of care in mild and moderate COVID-19: A randomized, controlled, multicentric clinical trial
Source: PLoS One. 2023 Mar 16;18(3):e0282688. doi: 10.1371/journal.pone.0282688 (PMC10019690; doi:10.1371/journal.pone.0282688)
Supplement: S4 File — (DOCX) [file pone.0282688.s004.docx]

Coadministration of AYUSH 64 as an adjunct to Standard of Care in mild and moderate COVID-19: A randomised, controlled, multicentric clinical trial

**S4 File. Health and Quality of Life Questionnaires** **(WHO-QOL & HR-BHF)**

**Text Box S4. 1: A summary of the WHO Quality of Life (QOL)-Bref Questionnaire used in the randomized controlled study to evaluate the co-administration of AYUSH 64 (Ayurvedic drug) with standard of care in mild - moderate patients of COVID-19.**

WHO QOL-Bref (Dec 1996 version) has 27 questions belonging to four major domains. Each question was answered on a 5-item categorical response Likert scale and each category was scored on a 1 to 5 scale in an ascending disorder: very poor, poor, neither poor nor good, good, and very good (22). The 4 domains pertained to physical health (such as activities of daily living, sleep, work capacity), psychological health ( such as bodily image, positive feelings, negative feelings, self- esteem, spirituality), social relationships (such as personal relations, social support, sexual activity) and environmental wellbeing (such as finances, physical safety, health and social care, home environment, transport). The range of score for each of the domains varies- physical health (7-35), psychological health (6-30), social relationships (3-15) and environmental well- being (8- 40); range of score shown in parenthesis.

Administration: A hard copy of the questionnaire was administered to each participant for self-reporting by a designated study para medic at time points described in the study protocol. A window period of two weeks is allowed to obtain the optimum response. The subject was asked to choose the likely response to each question as per experience and place a tick mark in the adjacent box showing the score of the response. Suitable local translations of the questionnaire in Hindi and Marathi language were used by the patients to record their response in the current study under reference.

Scoring: The score for 3 questions were reversed to follow the ascendancy order of the remaining questions. Higher scores meant better quality of life as per instructions of the questionnaire. The score of each question was added by the designated paramedic to calculate the score for each domain (manually calculated sum of raw scores in each domain) and the total score. The score was recorded in the questionnaire and entered into the study database.

Reference: WHOQOL-BREF : Introduction, Administration, Scoring And Generic Version Of The Assessment. Field Trial Version, December 1996. Available at https://www.who.int/mental_health/media/en/76.pdf. Accessed on 26 May 2021

**Text Box S4.2: Health Related-Behaviour, Habit and Fitness Questionnaire (HR-BHF, CRD, Pune 2020 version)**

CONFIDENTIAL

ARTHRTIS RESEARCH CARE FOUNDATION-CENTRE FOR RHEUMATIC DISEASES PUNE INDIA HEALTH RELATED-BEHAVIOUR HABIT FITNESS (HR-BHF)

PARTICIPANT NO: PARTICIPANT ID Age Gender

PARTCIPANT NAME (OPTIONAL) Mobile (optional) Address (General location, include name of village/city & district)

DATE:

Note: The participant is permitted to stay anonymous and not provide any contact details- only ID will suffice.

This is a research study. It will be used in clinical practice after testing its usefulness and acceptance by community and doctors.

Please put a vertical line across the scale to indicate your extent of the health-related problem

1. **Please consider YOUR EXPERIENCE OVER THE PAST ONE WEEK while answering. We want you to give your overall assessment.**
2. **GENERAL HEALTH: How would you rate your overall general health? 0 100**

Very Poor Very Good

1. **How much is your anxiety?**

0 _100

Very anxious No anxiety at all

1. **Fatigue: How much do you feel tired in your daily routine?**

0 _100

Nil Feel very tired

1. **How much is your Energy level?**

0 _100

Low energy Feel Energetic during my daily routine

1. **How is your Bowel movement (passing stools)?**

0 100

Highly irregular and unsatisfying Satisfactory and normal bowel habit

1. **Are you stressed?**

0 _100

No Stress Maximum Stress

1. **How happy are you?**

0 100

Very Sad Very Happy

1. **How do you rate your sleep?**

0 100

Normal Bad and unable to sleep properly

1. **How much is your appetite for food?**

0 100

Nil Very Good

REMARKS

# THANK YOU FOR YOUR PARTICIPATION. You may contact us at +912026344099/26345624 or Email: [crdp5624@gmail.com](mailto:crdp5624@gmail.com)

***and visit us at*** [***www.rheumatologyindia.org***](http://www.rheumatologyindia.org/)

Note that the above illustration is not drawn to scale.

**Text Box S4. 3: Development and Validation of Health Related-Behaviour, Habit and Fitness Questionnaire (HR-BHF CRD Pune 2020 version)**

HR-BHF was developed by Centre for Rheumatic Disease CRD), Pune in Feb 2020 to address the various complaints of general health related physical and mental health issues which are often associated with chronic disorders and often ignored by the doctors. But patients consider them important and they effect outcome. The latter was recognized by the rheumatologist (AC) in CRD. A new quality of life (QOL) assessment instrument namely HR-BHF (CRD Pune 2020) was developed. Inadvertently, this exercise coincided with the COVID 19 pandemic. We also speculated HR-BHF to be useful in assessing recovery in COVID 19. A summary along with the instrument is presented (results not yet published).

An inventory of 23 questions was created based on the information gathered from patients suffering from chronic arthritis and attending CRD outpatient from Dec 2019 to Feb 2020. A local CRD expert group of 3 physicians, 4 paramedics, 10 healthy community (5 senior citizens) and 12 patients decided to reduce the number of questions 9 after deliberate discussions and consensus. There were 14 women members in the expert group. The group opined that the questionnaire is likely to capture some vital aspects of human behaviour, habits and fitness (more mental than physical) and improve patient satisfaction. The face validity and suitable translations (local language) were confirmed by this expert group.

The 9 questions pertained to general health, anxiety, fatigue, energy level, bowel habits, stress, happiness, sleep and appetite (food). Patients marked the answer on a visual analogue scale (VAS) to indicate a measure of the difficulty or health status. VAS was a 100 mm horizontal bland line (no tick marks) anchored at 0 and 100 mm at either end for the extreme outcome. The patient drew a a short vertical mark on the VAS to record response and the intersection point was accurately measured from ‘0’ to provide the score. Addition of individual scores led to a composite HR-BHF score (range 0-900). The optimum response (best of health) for 5 questions (general health, energy happiness, bowel habit and appetite) was 100 and for 3 questions (anxiety, stress, depression) it was ‘0’ and this was decided after due consideration of the way patients understand the question.

Subsequently, an in-house evaluation of performance of HR-BHF was performed by AC and colleagues. 403 consenting participants [103 patients of chronic rheumatoid arthritis (RA) and 300 healthy community (HC] completed the questionnaire in face to face interview with a paramedic. 72.8% RA and 88.3% HC did not report ‘any difficulty’ in answering the questions; remaining had ‘some difficulty’. 100% RA and 98.4% HC reported HR-BHF to be ‘Useful to very useful’. The mean VAS score was 56.4 mm for general health, 43.6 mm for anxiety, 45.9 mm for fatigue, 55.2 mm for energy, 67.6 mm for good bowel clearance, 34.1 mm for stress, 67.9 mm for happiness, 38.8 mm for sleep and 68.7 mm for appetite in the RA group; correspondingly it was 81.7 mm, 34.7 mm, 27.9 mm, 77.1 mm, 76.7mm, 20.1 mm, 79.4mm, 33.5 mm, and 81.8 mm in the HC and significantly different (p<0.05, Student t test) for all measures except sleep. In the correlation matrix, general health was shown positively correlated with energy, good bowel clearance, happiness and good appetite and negatively correlated with anxiety, fatigue, stress and sleep. The correlation between each of the item questions varied from -0.126 to 0.503. 8 questions (HR-BHF) could explain 32.7% (adjusted R^2^ ) variation in the score of ‘general health’ (dependent variable, 9^th^ question in HR-BHF) in a multivariable regression model; data from the total cohort of 403 participants was used. The evaluation results were consistent with the content validity and clinical usefulness.

HR-BHF was used in the current drug trial of AYUSH 64 (Ayurvedic drug) and standard of care in mild and moderate COVID-19 as per protocol. The score at several time points was analysed to show response to intervention and recovery. The results of HR-BHF were also consistent with the standard WHO-QOL Bref questionnaire (See main text Table 8 and supplement material Table 5) .

The HR-BHF questionnaire is shown in supplement material Box 2

.

**Table S4.1: Comparison of individual question score (mean ± standard deviation) in Health Related- Behaviour, Habit and Fitness (HR-BHF, CRD Pune 2020 version) questionnaire between AYUSH 64 plus standard of care (SOC) and SOC : A** **randomized controlled study to evaluate the co-administration of AYUSH-64 with Standard of Care (SOC) in mild - moderate symptomatic COVID-19 (n=139)**

| Variable | Baseline (n=139) | | Discharge  (n=137) | Week 4  (n=129) | Week 8  (n=127) | Week 12  (n=120) |
| --- | --- | --- | --- | --- | --- | --- |
| HR-BHF-General Health | | | | | | |
| AYUSH plus | 60.85 ± 13.5 | 76.69 ± 10.0 | | 81.27 ± 9.8 | 84.87 ± 8.9 | 89.31 ± 8.6 |
| SOC | 58.29 ± 17.5 | 73.88 ± 13.3 | | 79.97 ± 12.0 | 83.57 ± 10.4 | 86.53 ± 9.2 |
| HR-BHF -Anxiety | | | | | | |
| AYUSH plus | 46.05 ± 21.1 | 67.02 ± 23.1 | | 61.75 ± 32.0 | 65.19 ± 33.7 | 68.74 ± 34.8* |
| SOC | 43.48 ± 22.4 | 69.22 ± 16.9 | | 58.95 ± 32.3 | 61.59 ± 33.1 | 57.79 ± 36.1 |
| HR-BHF-Fatigue | | | | | | |
| AYUSH plus | 48.06 ± 19.5 | 24.60 ± 14.0* | | 26.89 ± 17.1 | 21.02 ± 19.1 | 15.08 ± 13.4* |
| SOC | 51.91 ± 18.7 | 32.5 ± 18.5 | | 28.07 ± 18.7 | 22.09 ± 18.2 | 21.69 ± 19.4 |
| HR-BHF-Energy | | | | | | |
| AYUSH plus | 54.34 ± 18.2 | 72.26 ± 13.7 | | 77.63 ± 14.9 | 82.23 ± 13.1 | 87.65 ± 10.8 |
| SOC | 57.5 ± 17.0 | 71.98 ± 9.6 | | 77.55 ± 13.4 | 79.93 ± 14.2 | 83.62 ±15.2 |
| HR-BHF-Bowel movement | | | | | | |
| AYUSH plus | 60.97 ± 19.6 | 74.44 ± 12.0 | | 79.68 ± 16.1 | 83.94 ± 10.0 | 85.11 ±13.8 |
| SOC | 64.6 ± 19.2 | 76.38 ± 10.8 | | 78.34 ±15.6 | 80.66 ± 14.7 | 82.74 ± 12.1 |
| HR-BHF-Stress | | | | | | |
| AYUSH plus | 44.15 ± 20.6 | 21.13 ± 14.0* | | 19.81 ± 15.4 | 17.37 ± 15.1 | 15.69 ± 16.6 |
| SOC | 45.69 ± 21.1 | 26.47 ± 16.2 | | 22.29 ± 17.5 | 21.52 ± 17.6 | 18.09 ± 17.1 |
| HR-BHF-Happiness | | | | | | |
| AYUSH plus | 56.29 ± 21.7 | 79.44 ± 15.1** | | 83.92 ± 12.2** | 86.02 ± 10.3** | 88.58 ± 8.1** |
| SOC | 57.84 ± 20.3 | 60.52 ± 31.2 | | 58.59 ± 31.2 | 61.52 ± 35.6 | 61.28 ± 36.9 |
| HR-BHF-Sleep | | | | | | |
| AYUSH plus | 48.89 ± 20.5 | | 32.82 ± 21.8 | 29.73 ± 28.0 | 29.16 ± 28.0 | 28.16 ± 31.3 |
| SOC | 54.36 ± 22.0 | | 30.17 ± 18.5 | 33.1 ± 30.4 | 29.5 ± 26.8 | 32.91 ± 31.4 |
| HR-BHF questionnaire -Appetite | | | | | | |
| AYUSH plus | 62.69 ± 17.2 | | 76.13 ± 12.5 | 83.06 ± 13.3 | 85.95 ± 8.0 | 87.65 ± 8.3* |
| SOC | 63.88 ± 18.5 | | 74.31 ± 10.5 | 80.67 ± 14.9 | 83.57 ±11.2 | 83.48 ± 12.3 |
| Note: (1) *: p<0.05, **p<0.01 (Mann Whitney statistic) on comparison of the two study groups at study time point  (2) See Supplement File 4 Text Box SF 4.2 and 4.3 (above) for details. HR-BHF contained nine questions- General health, anxiety, fatigue, energy, bowel habit, stress, happiness, sleep and appetite. Each question was scored from 0-100. Low scores for fatigue, stress and sleep indicate better response; High scores for general health, appetite, energy, anxiety, bowel habit, happiness indicate better response; See main text for further details  (3)n : number of study participants | | | | | | |
