# Supplementary material for: Co-administration of AYUSH 64 as an adjunct to standard of care in mild and moderate COVID-19: A randomized, controlled, multicentric clinical trial
Source: PLoS One. 2023 Mar 16;18(3):e0282688. doi: 10.1371/journal.pone.0282688 (PMC10019690; doi:10.1371/journal.pone.0282688)
Supplement: S5 File — (DOCX) [file pone.0282688.s005.docx]

Coadministration of AYUSH 64 as an adjunct to Standard of Care in mild and moderate COVID-19: A randomised, controlled, multicentric clinical trial

**S 5 File. SOC drugs, Site Specific, Efficacy, and Radiological data**

**Table S5.1:** **Drugs with dosages used in the Standard of Care (SOC) treatment: a randomized controlled study to evaluate the co-administration of AYUSH-64 with Standard of Care (SOC) in mild - moderate symptomatic COVID-19 .**

| SOC Medicine  (Tab: Tablet) | Dosage |
| --- | --- |
| Tab Azithromycin | 500 mg od x3-5 days |
| Tab Doxycycline | 100 mg bid x 5 days |
| Tab Hydroxychloroquine sulphate | 400 mg od x 5 days (1st day bid) |
| Tab Zincovit (Zinc plus Vitamin B complex) | 50-100 mg (zinc content( od |
| Tab Vitamin C | 500 mg bid |
| Tab Multivitamin | Composite |
| Tab Vitamin D3 | 400 iu od, (with calcium citrate 500 mg) |
| Tab Pantoprazole | 20-40 mg od |
| Tab Paracetamol | 500-650 mg ,1-2 times on need basis |
| Tab Cetrizine | 5-10 mg od x 3-5 days |
| Tab Ivermectin | 12 mg od X 5 days |
| Injection Dexamethasone | 4-8 mg i/v stat |
| Injection Low molecular weight heparin analogue (clexane) | 40 mg s/c od x5-10 days |
| Oxygen intermittent | 2-4 l/min, nasal face mask |

**Table S5.2: Site specific data on some clinical variables (timelines) and individual standard of care drugs use (number of study participants and proportion %) in a randomized controlled study to evaluate the co-administration of AYUSH-64 with Standard of Care (SOC) in mild - moderate symptomatic COVID-19 (See main text for details)**

|  | Mumbai (n=60) | Nagpur (n=30) | Lucknow (n=49) |
| --- | --- | --- | --- |
| **Onset-Symptom to RT-PCR assay (days, mean, standard deviation)** | | | |
| AYUSH plus | 7 ± 5.6 | 0.5 ± 1.5 | 4.3 ± 2.8 |
| Standard of Care | 6.2 ± 4.5 | 1.5 ± 3.3 | 4.8 ± 3.8 |
| **RT-PCR assay to Hospitalization (days, mean, standard deviation)** | | | |
| AYUSH plus | 2.5 ± 1.1 | 2.0 ± 0.1 | 3.0 ± 0.8 |
| Standard of Care | 2.6 ± 0.8 | 2.0 ± 0.1 | 3.2 ± 0.7 |
| **Hospitalization to randomization (days, mean, standard deviation)** | | | |
| AYUSH plus | - 1. ± 0.6 | 1.3 ± 0.5 | 1.6 ± 1.1 |
| Standard of Care | 1.1 ± 0.4 | 1.5 ± 1.2 | 1.8 ± 0.9 |
| **Tablet Azithromycin** | | | |
| AYUSH plus | 30 (50) | 12 (40) | 6 (12.2) |
| Standard of Care | 28 (46.6) | 15 (50) | 6 (12.2) |
| **Tablet Hydroxychloroquine Sulphate** | | | |
| AYUSH plus | 14 (23.3) | 14 (46.6) | 1 (2.0) |
| Standard of Care | 08 (13.3) | 15 (50) | 1 (2.0) |
| **Zincovit (zinc plus Vitamin B complex) Tablet** | | | |
| AYUSH plus | 11 (18.3) | 12 (40) | 25 (51) |
| Standard of Care | 02 (3.3) | 15 (50) | 25 (51) |
| **Vitamin C Tablet** | | | |
| AYUSH plus | 30 (50) | 14 (46.6) | 25 (51) |
| Standard of Care | 29 (48.3) | 15 (50) | 25 (51) |
| **Tablet Pantoprazole** | | | |
| AYUSH plus | 28 (46.6) | 13 (43.3) | 25 (51) |
| Standard of Care | 26 (43.3) | 14 (46.6) | 25 (51) |
| **Tablet Paracetamol** | | | |
| AYUSH plus | 19 (31.6) | 15 (50) | 25 (51) |
| Standard of Care | 15 (25) | 15 (50) | 25 (51) |
| **Tablet Cetrizine** | | | |
| AYUSH plus | 0 | 13 (43.3) | 0 |
| Standard of Care | 1 (1.6) | 14 (46.6) | 0 |
| **Intermittent Oxygen** | | | |
| AYUSH plus | 9 (15) | 0 | 0 |
| Standard of Care | 5 (8.3) | 1 (3.3) | 0 |

**Table S5.3 - Result of a General Linear [Mixed Effects] Model with ‘Time from Randomization to Clinical Recovery’ as dependent variable in 134 patients completing the study intervention at different study sites as per protocol (Qualifiers) -A randomized control study to evaluate the efficacy of AYUSH 64 plus standard of care (An Output from SPSS statistical software analysis, See main text for details)**

| **Tests of Between-Subjects Effects-** **Dependent Variable: Time from Randomization to Clinical Recovery** | | | | | | |
| --- | --- | --- | --- | --- | --- | --- |
| Source | | Type III Sum of Squares | df | Mean Square | F | Sig. |
| Intercept | Hypothesis | 7308.439 | 1 | 7308.439 | 40.724 | .023 |
|  | Error | 360.793 | 2.010 | 179.464^a^ |  |  |
| GROUP | Hypothesis | 121.228 | 1 | 121.228 | 72.191 | .001 |
|  | Error | 6.403 | 3.813 | 1.679^b^ |  |  |
| Site | Hypothesis | 375.117 | 2 | 187.558 | 147.349 | .007 |
|  | Error | 2.546 | 2 | 1.273^c^ |  |  |
| GROUP * Site | Hypothesis | 2.546 | 2 | 1.273 | .125 | .883 |
|  | Error | 1302.832 | 128 | 10.178^d^ |  |  |
| \| **Table S5.4 - Results of a General Linear [Mixed Effects] Model with ‘Time from Onset of symptoms to Clinical Recovery’ as dependent variable in 134 patients completing the study intervention at different study sites as per protocol (Qualifiers) -A randomized control study to evaluate the efficacy of AYUSH 64 plus standard of care (An Output from SPSS statistical software analysis, See main text for details)Tests of Between-Subjects Effects-** **Dependent Variable: Time from onset of symptoms to Clinical Recovery** \| \| \| \| \| \| \| \| --- \| --- \| --- \| --- \| --- \| --- \| --- \| \| Source \| \| Type III Sum of Squares \| df \| Mean Square \| F \| Sig. \| \| Intercept \| Hypothesis \| 23215.792 \| 1 \| 23215.792 \| 138.282 \| .007 \| \| Error \| 340.885 \| 2.030 \| 167.888^a^ \|  \|  \| \| GROUP \| Hypothesis \| 147.229 \| 1 \| 147.229 \| 11.256 \| .059 \| \| Error \| 32.046 \| 2.450 \| 13.080^b^ \|  \|  \| \| Site \| Hypothesis \| 349.183 \| 2 \| 174.592 \| 14.101 \| .066 \| \| Error \| 24.763 \| 2 \| 12.381^c^ \|  \|  \| \| GROUP * Site \| Hypothesis \| 24.763 \| 2 \| 12.381 \| .447 \| .640 \| \| Error \| 3543.299 \| 128 \| 27.682^d^ \|  \|  \| | | | | | | |

**Table S5.5:** **Primary efficacy measure (randomization to clinical recovery) in all study participants completing study intervention (including disqualifiers):** **A randomized controlled study to evaluate the co-administration of AYUSH-64 with Standard of Care (SOC) in mild - moderate symptomatic COVID-19 (n=137)- Intention to treat analysis**

| Time line  (days) | Mumbai (n=58) | | Nagpur (n=30) | | Lucknow (n=49) | | Total Study  (n=137)# | |
| --- | --- | --- | --- | --- | --- | --- | --- | --- |
|  | AYUSH plus (n=29) | SOC (n=29) | AYUSH plus (n=15) | SOC (n=15) | AYUSH plus (n=24) | SOC (n=25) | AYUSH plus (n=68) | SOC (n=69) |
| Randomization to Clinical Recovery  Mean ± SD | 6.79  ±2.11 | 8.45  ±3.75 | 8.80  ±1.01 | 11.33  ±4.86 | 4.50  ±1.56 | 6.40  ± 4.03 | 6.43  ±2.36 | 8.33  ±4.44 |
| 95 % CI of Difference between Means | -3.23 to -0.09 | | - 5.04 to -0.02 | | -3.62 to - 0.17 | | - 3.09 to - 0.71 | |
| Between Groups Comparison-P by ‘t’ test | 0.04 | | 0.067 | | 0.036 | | 0.002 | |
| *Statistically significant p(0.05  NS Not statistically significant p>=0.05  a. Student’s ‘t test, two independent samples, unequal variance  b. Note: n: number of participants; AYUSH plus: AYUSH 64 plus SOC; 137 participants completed randomization treatment phase and recorded primary efficacy measure as per protocol and includes 3 participants who was disqualified based on protocol deviation; See Text for details | | | | | | | | |

**Table S5.6: Comparing radiological data (assessor blind) derived from skiagram of the chest in the two study groups:** **A randomized controlled study to evaluate the co-administration of AYUSH-64 with Standard of Care (SOC) in mild - moderate symptomatic COVID-19 (n=86) study participants enrolled in two study sites- Shows proportion (percent) of study participants**

| Radiological Feature | AYUSH 64 plus Standard of Care (n=43) | Standard of Care  (n=43) |
| --- | --- | --- |
| Overall Radiological abnormalities consistent with COVID-19 on randomization | 62.8% | 62.8% |
| Radiological abnormalities of definite pneumonia consistent with COVID-19 on randomization | 18.6% | 30.2% |
| Radiological abnormalities consistent with COVID-19 and not completely resolved at the time of complete recovery (clinical)/hospital discharge | 25.6% | 34.9% |
| Radiological abnormalities of pneumonia consistent with COVID-19 and not completely resolved at the time of complete recovery (clinical)/hospital discharge | 62.5% | 84.6% |
