# Supplementary material for: Co-administration of AYUSH 64 as an adjunct to standard of care in mild and moderate COVID-19: A randomized, controlled, multicentric clinical trial
Source: PLoS One. 2023 Mar 16;18(3):e0282688. doi: 10.1371/journal.pone.0282688 (PMC10019690; doi:10.1371/journal.pone.0282688)
Supplement: S6 File — (DOCX) [file pone.0282688.s006.docx]

Coadministration of AYUSH 64 as an adjunct to Standard of Care in mild and moderate COVID-19: A randomised, controlled, multicentric clinical trial

**S6 File. Additional Data-Adverse Events**

**Table S6.1: Number of Adverse Events at all endpoints by System organ classification and Preferred term in a randomized controlled study to evaluate the co-administration of AYUSH-64 with Standard of Care (SOC) in mild - moderate symptomatic COVID-19 (n=139)**

|  |  | SOC (n=70) | | | | AYUSH plus (n=69) | | | |
| --- | --- | --- | --- | --- | --- | --- | --- | --- | --- |
| System organ classification (WHO) | Preferred term | During Hosp. | Week 4 | Week 8 | Week 12 | During Hosp | Week 4 | Week 8 | Week 12 |
| Cardiac | Raised Blood Pressure | - | - | - | - | - | 1 | - | - |
| Ear and labyrinth | Ear Ache | - | - | - | 1 | - | - | - | - |
| Gastrointestinal | Gastritis | - | 1 | - | - | - | - | - | - |
|  | Abdominal Discomfort | - | - | 1 | - | - | 1 | - | - |
|  | Diarrhea | - | 1 | - | - | - | 3 | 1 | - |
|  | Constipation | - | - | - | 1 | - | - | 1 | 1 |
|  | Epigastric Pain | - | - | - | - | - | - | 1 | - |
|  | Hyperacidity | - | - | - | - | - | 1 | 1 | - |
|  | Abdominal Pain | - | 1 | - | - | - | - | - | - |
| Hepatobiliary | Raised SGOT/SGPT | - | - | - | 1 | - | - | - | - |
| Infections and infestations | Fever | - | 1 | - | 1 | - | 1 | - | 4 |
|  | Malaria (P Vivax) | 1 | - | - | 1 | - | - | - | - |
|  | Cellulitis | 1 | - | - | - | - | - | - | - |
|  | Sore throat | - | 2 | - | 2 | - | 1 | - | - |
| Musculoskeletal and connective tissue | Neck Pain | - | - | - | 1 | - | - | 1 | 1 |
|  | Backache | - | - | - | 1 | 1 | - | - | - |
|  | Leg Pain | - | - | - | - | - | - | - | 1 |
|  | Ankle Pain | - | - | - | - | - | - | - | 1 |
|  | Joint Pain | - | - | - | - | - | 1 | - | - |
| Skin and subcutaneous tissue | White patches hands | - | - | 1 | - | - | - | - | - |
|  | Itching | - | - | - | - | - | - | 1 | - |
|  | Eczema | - | - | - | - | - | 1 | - | - |
| Respiratory, thoracic and mediastinal |  |  |  |  |  |  |  |  |  |
|  | Cough | - | 1 | 1 | - | - | 1 | - | - |
|  | Breathlessness | - | 1 | 2 | 2 | - | 4 | 2 | - |
|  | Loss of smell + Loss of Taste + Sore Throat + Breathlessness | - | 1 | - | - | - | - | - | - |
| Nervous system | GB Syndrome | - | - | - | - | 1 | - | - | - |
|  | Vertigo | - | - | - | - | 1 | 1 | - | - |
| Renal and urinary | Burning Micturition | - | - | 1 | - | - | - | - | - |
| Endocrine | High Blood Glucose levels | - | 3 | - | 3 | - | 2 | - | 4 |
| Investigations | Increased Triglycerides, LDL | - | 1 | - | - | - | - | - | - |
| Others | Weakness | - | 4 | 2 | - | - | 1 | - | 1 |
|  | Chills | - | - | - | 1 | - | - | - | - |
|  | Myalgia | - | 2 | 2 | 2 | - | 1 | 1 | - |
|  | Headache | - | - | - | - | - | 1 | 2 | - |
| Total |  | 2 | 19 | 10 | 17 | 3 | 21 | 11 | 13 |
| Note: Hosp:hospitalization; Note: AYUSH plus: AYUSH 64+ SOC: n: number of participants; SGOT/SGPT: serum glutamaseoxalacetate, serum gluatamase; No AE recorded for disorders of blood and lymphatic, immune system, metabolism and nutrition, psychiatric, reproductive system and breast, eye, vascular system, congenital familial and genetic, injury poisoning and procedural complications, and surgical and medical procedures; See text for detail | | | | | | | | | |
