# Supplementary material for: Co-administration of AYUSH 64 as an adjunct to standard of care in mild and moderate COVID-19: A randomized, controlled, multicentric clinical trial
Source: PLoS One. 2023 Mar 16;18(3):e0282688. doi: 10.1371/journal.pone.0282688 (PMC10019690; doi:10.1371/journal.pone.0282688)
Supplement: S7 File — (DOCX) [file pone.0282688.s007.docx]

Coadministration of AYUSH 64 as an adjunct to Standard of Care in mild and moderate COVID-19: A randomised, controlled, multicentric clinical trial

**S7 File. Additional Data- Laboratory Results**

**Table S7.1: Laboratory investigations [Data expressed as Mean ± SD] in a randomized controlled study to evaluate the co-administration of AYUSH-64 with Standard of Care (SOC) in mild - moderate symptomatic COVID-19: per protocol completer analysis**

| ***Blood /serum assay*** | | | | | |
| --- | --- | --- | --- | --- | --- |
| **Haemoglobin (g/dl)** | | | | | |
| Timepoints | Baseline (n=139) | On Discharge  (n=137) | Week 4  (n=129) | Week 8  (n=127) | Week 12  (n= 120) |
| AYUSH plus | 13.62 ± 1.42 | 13.34 ± 1.49 | 13.18 ± 1.62 | 13.54 ± 1.61 | 13.53 ± 1.78 |
| SOC | 13.80 ± 1.62 | 13.41 ± 1.90 | 13.67 ± 1.29 | 13.80 ± 1.45 | 13.94 ± 1.39 |
| **Red Blood Cells – Total (million/mm^3^)** | | | | | |
| AYUSH plus | 4.71 ± 0.59 | 4.69 ± 0.54 | 4.56 ± 0.55 | 4.65 ± 0.56 | 4.77 ± 0.63 |
| SOC | 4.69 ± 0.68 | 4.81 ± 0.70 | 4.64 ± 0.52 | 4.71 ± 0.55 | 4.81 ± 0.54 |
| **White Blood Cells – Total (cells/ mm^3^)** | | | | | |
| AYUSH plus | 5920.69 ± 2008.8 | 6781.39 ± 1513.6 | 7156.6 ± 1634.0 | 7228.57 ± 1334.6 | 6827.27 ± 1747.7 |
| SOC | 6828.30 ± 2085.8 | 6650 ± 1911.0 | 6888 ± 1426.7 | 7024.49 ± 1292.2 | 7001.92 ± 1450.8 |
| **Platelets (lakhs/ mm^3^)** | | | | | |
| AYUSH plus | 2.73 ± 1.31 | 3.88 ± 1.63 | 2.60 ± 0.98 | 2.60 ± 0.87 | 2.63 ± 0.86 |
| SOC | 2.55 ± 1.48 | 3.34 ± 1.55 | 2.39 ± 1.00 | 2.33 ± 0.93 | 2.48 ± 0.93 |
| **Erythrocyte sedimentation rate**  **(mm/hour)** | | | | | |
| AYUSH plus | 50.19 ± 38.03 | 42.98 ± 36.22 | 31.63± 26.21 | 26.77± 22.36 | 21.10 ± 21.05 |
| SOC | 46.86 ± 37.37 | 42.68 ± 34.19 | 21.07± 14.27 | 23.77± 15.10 | 15.75 ± 10.76 |
| ***Liver Function Tests*** | | | | | |
| **Total Bilirubin** (mg/dL) | | | | | |
| AYUSH plus | 0.64 ± 0.28 | 0.61 ± 0.27 | 0.70 ± 0.30 | 0.75 ± 0.47 | 0.79 ± 0.37 |
| SOC | 0.78 ± 0.51 | 0.64 ± 0.35 | 0.80 ± 0.34 | 0.82 ± 0.35 | 0.88 ± 0.39 |
| **Total Proteins** (mg/mL) | | | | | |
| AYUSH plus | 7.12 ± 0.59 | 8.33 ± 3.42 | 7.20 ± 0.51 | 7.23 ± 0.57 | 7.34 ± 0.67 |
| SOC | 7.09 ± 0.66 | 7.07 ± 0.57 | 7.20 ± 0.52 | 7.16 ± 0.52 | 7.16 ± 0.74 |
| **Serum Globulin** (g/dL) | | | | | |
| AYUSH plus | 3.14 ± 0.49 | 3.20 ± 0.35 | 3.12 ± 0.39 | 3.14 ± 0.39 | 3.10 ± 0.44 |
| SOC | 3.08 ± 0.61 | 3.13 ± 0.52 | 3.07 ± 0.40 | 3.08 ± 0.39 | 2.99 ± 0.50 |
| **Serum Albumin** (g/dL) | | | | | |
| AYUSH plus | 3.99 ± 0.39 | 3.89 ± 0.35 | 4.07 ± 0.30 | 4.09 ± 0.28 | 4.24 ± 0.38 |
| SOC | 4.0 ± 0.41 | 3.89 ± 0.41 | 4.14 ± 0.31 | 4.08 ± 0.23 | 4.15 ± 0.39 |
| **Albumin/Globulin ratio** | | | | | |
| AYUSH plus | 1.34 ± 0.38 | 1.23 ± 0.19 | 1.32 ± 0.20 | 1.32 ± 0.17 | 1.38 ± 0.20 |
| SOC | 1.36 ± 0.33 | 1.31 ± 0.24 | 1.47 ± 0.59 | 1.36 ± 0.18 | 1.40 ± 0.21 |
| **Aspartate aminotransferase** (units/L) | | | | | |
| AYUSH plus | 42.96 ± 32.33 | 36.91 ± 28.39 | 28.64± 14.29 | 27.48± 14.02 | 30.44 ± 18.15 |
| SOC | 43.93 ± 28.49 | 37.99 ± 24.78 | 33.26± 22.32 | 29.47± 11.35 | 31.41 ± 18.79 |
| **Alkaline Phosphatase** (units/L) | | | | | |
| AYUSH plus | 106.45 ± 63.37 | 85.33 ± 40.38 | 103.78± 45.26 | 116.93± 66.48 | 108.38 ± 53.72 |
| SOC | 113.80 ± 68.58 | 91.25 ± 53.91 | 111.29 ± 51.17 | 116.78 ± 63.57 | 110.37 ± 61.62 |
| **Renal function test** | | | | | |
| **Serum Creatinine** (mg/dL) | | | | | |
| AYUSH plus | 1.02 ± 0.23 | 0.96 ± 0.50 | 0.99 ± 0.23 | 1.02 ± 0.23 | 1.01 ± 0.24 |
| SOC | 1.00 ± 0.31 | 1.08 ± 0.68 | 0.92 ± 0.22 | 0.93 ± 0.19 | 1 ± 0.29 |
| **Blood Urea Nitrogen** (mg/dL) | | | | | |
| AYUSH plus | 14.97 ± 8.28 | 10.74 ± 7.25 | 14.53± 12.17 | 17.09± 13.09 | 15.70 ± 10.16 |
|  | 17.25 ± 10.82 | 10.91 ± 7.05 | 15.97± 10.02 | 17.43± 10.50 | 17.02 ± 9.86 |
| **Blood Sugar Level** (mg/dL) | | | | | |
| AYUSH plus | 112.50 ± 37.54 | 120.11 ± 54.21 | 124.51 ± 59.30 | 96.61± 18.49 | 98.97 ±15.40 |
| SOC | 114.17 ± 35.23 | 125.37±34.90 | 122.39± 58.25 | 92.99± 10.83 | 92.26±11.09 |
| Note: AYUSH plus: AYUSH 64 + SOC; No significant difference between the groups for any of the assay shown above at significant p<0.05, ANOVA; n; number of participants; study patients with diabetes excluded for blood sugar data shown above. See text for detail. | | | | | |
