# Supplementary material for: Co-administration of AYUSH 64 as an adjunct to standard of care in mild and moderate COVID-19: A randomized, controlled, multicentric clinical trial
Source: PLoS One. 2023 Mar 16;18(3):e0282688. doi: 10.1371/journal.pone.0282688 (PMC10019690; doi:10.1371/journal.pone.0282688)
Supplement: S8 File — (DOCX) [file pone.0282688.s008.docx]

Coadministration of AYUSH 64 as an adjunct to Standard of Care in mild and moderate COVID-19: A randomised, controlled, multicentric clinical trial

**S 8 File.** **Selected Raw Data - Efficacy, Withdrawals, Adverse events**

**TABLE 1: AYUSH 64 DRUG TRIAL- RAW DATA OF STUDY PARTICIPANTS (N=140) FOR PRIMARY EFFICACY MEASURE AND SELECTED TIMELINES: A RANDOMIZED CONTROLLED STUDY COMPARING AYUSH 64 PLUS STANDARD OF CARE AND STANDARD OF CARE IN MILD-MODERATE COVID-19**

| Patient ID | Randomisation Group A (AYUSH 64 + SOC) or B (SOC/Standard of Care) | Age in years | Gender (Male/Female | Site (1/2/3) | Time-Randomization to clinical recovery days | Time-Symptom onset to clinical recovery days | | | REMARK |
| --- | --- | --- | --- | --- | --- | --- | --- | --- | --- |
| 1701001S006 | A | 38 | M | 1 | 7 | 15 | | | - |
| 1701001S007 | A | 33 | M | 1 | 9 | 16 | | | - |
| 1701001S011 | A | 59 | M | 1 | 7 | 20 | | | - |
| 1701001S013 | A | 55 | F | 1 | 7 | 15 | | | - |
| 1701001S018 | A | 42 | M | 1 | 6 | 12 | | | - |
| 1701001S019 | A | 36 | F | 1 | 10 | 28 | | | - |
| 1701001S022 | A | 48 | F | 1 | 5 | 33 | | | - |
| 1701001S025 | A | 32 | M | 1 | 5 | 7 | | | - |
| 1701001S026 | A | 47 | M | 1 | 7 | 14 | | - | |
| 1701001S027 | A | 20 | F | 1 | 5 | 8 | | - | |
| 1701001S030 | A | 45 | M | 1 | 7 | 20 | | - | |
| 1701001S031 | A | 26 | M | 1 | 4 | 14 | | - | |
| 1701001S032 | A | 46 | M | 1 | 5 | 8 | | - | |
| 1701001S037 | A | 28 | M | 1 | 8 | 18 | | - | |
| 1701001S039 | A | 32 | M | 1 | 6 | 9 | | - | |
| 1701001S043 | A | 68 | F | 1 | 4 | 6 | | - | |
| 1701001S044 | A | 48 | M | 1 | 5 | 15 | | - | |
| 1701001S047 | A | 43 | M | 1 | 8 | 19 | | - | |
| 1701001S048 | A | 48 | M | 1 | 7 | 17 | | - | |
| 1701001S051 | A | 30 | F | 1 | 6 | 13 | | - | |
| 1701001S052 | A | 52 | M | 1 | 5 | 12 | | - | |
| 1701001S053 | A | 48 | M | 1 | 10 | 21 | | - | |
| 1701001S054 | A | 35 | F | 1 | 7 | 17 | | - | |
| 1701001S056 | A | 52 | M | 1 | 6 | 12 | | - | |
| 1701001S059 | A | 65 | M | 1 | 13 | 19 | - | | |
| 1701001S061 | A | 44 | M | 1 | 6 | 8 | - | | |
| 1701001S062 | A | 22 | M | 1 | 10 | 20 | - | | |
| 1701001S065 | A | 48 | M | 1 | 4 | 12 | - | | |
| 1701001S002 | B | 55 | M | 1 | 9 | 13 | - | | |
| 1701001S009 | B | 46 | M | 1 | 11 | 17 | - | | |
| 1701001S010 | B | 48 | M | 1 | 14 | 16 | - | | |
| 1701001S014 | B | 56 | M | 1 | 8 | 14 | - | | |
| 1701001S015 | B | 33 | M | 1 | 9 | 18 | - | | |
| 1701001S016 | B | 56 | M | 1 | 10 | 19 | - | | |
| 1701001S017 | B | 53 | M | 1 | 7 | 15 | - | | |
| 1701001S020 | B | 43 | M | 1 | 6 | 13 | - | | |
| 1701001S021 | B | 36 | M | 1 | 11 | 28 | - | | |
| 1701001S028 | B | 34 | M | 1 | 7 | 9 | - | | |
| 1701001S029 | B | 27 | F | 1 | 4 | 11 | - | | |
| 1701001S033 | B | 28 | M | 1 | 4 | 7 | - | | |
| 1701001S034 | B | 67 | F | 1 | 5 | 10 | - | | |
| 1701001S035 | B | 31 | M | 1 | 5 | 14 | - | | |
| 1701001S036 | B | 26 | M | 1 | 7 | 29 | - | | |
| 1701001S038 | B | 51 | M | 1 | 21 | 30 | - | | |
| 1701001S040 | B | 29 | M | 1 | 7 | 11 | - | | |
| 1701001S041 | B | 66 | M | 1 | 14 | 17 | - | | |
| 1701001S045 | B | 33 | M | 1 | 5 | 15 | - | | |
| 1701001S049 | B | 56 | M | 1 | 5 | 19 | - | | |
| 1701001S050 | B | 39 | M | 1 | 11 | 25 | - | | |
| 1701001S055 | B | 47 | M | 1 | 13 | 19 | - | | |
| 1701001S057 | B | 28 | F | 1 | 6 | 15 | - | | |
| 1701001S058 | B | 51 | M | 1 | 7 | 15 | - | | |
| 1701001S060 | B | 39 | M | 1 | 6 | 12 | - | | |
| 1701001S063 | B | 44 | M | 1 | 6 | 11 | - | | |
| 1701001S064 | B | 40 | M | 1 | 7 | 13 | - | | |
| 1701001S068 | B | 48 | M | 1 | 9 | 24 | - | | |
| 1701001S069 | B | 60 | M | 1 | 11 | 18 | - | | |
| 1701002S001 | A | 25 | M | 2 | 5 | 17 | - | | |
| 1701002S004 | A | 43 | M | 2 | 7 | 14 | - | | |
| 1701002S009 | A | 31 | M | 2 | 5 | 11 | - | | |
| 1701002S008 | A | 29 | M | 2 | 4 | 8 | - | | |
| 1701002S010 | A | 24 | M | 2 | 7 | 12 | - | | |
| 1701002S012 | A | 46 | F | 2 | 7 | 12 | - | | |
| 1701002S015 | A | 26 | M | 2 | 7 | 11 | - | | |
| 1701002S016 | A | 24 | M | 2 | 5 | 11 | - | | |
| 1701002S019 | A | 58 | F | 2 | 5 | 8 | - | | |
| 1701002S021 | A | 53 | M | 2 | 4 | 17 | - | | |
| 1701002S022 | A | 41 | M | 2 | 3 | 16 | - | | |
| 1701002S026 | A | 54 | M | 2 | 4 | 12 | - | | |
| 1701002S030 | A | 59 | M | 2 | 3 | 9 | - | | |
| 1701002S033 | A | 31 | M | 2 | 5 | 12 | - | | |
| 1701002S035 | A | 48 | M | 2 | 2 | 8 | - | | |
| 1701002S036 | A | 49 | F | 2 | 2 | 7 | - | | |
| 1701002S038 | A | 48 | M | 2 | 4 | 9 | - | | |
| 1701002S039 | A | 55 | F | 2 | 3 | 12 | - | | |
| 1701002S041 | A | 51 | M | 2 | 6 | 11 | - | | |
| 1701002S042 | A | 30 | M | 2 | 4 | 13 | - | | |
| 1701002S043 | A | 59 | M | 2 | 4 | 11 | - | | |
| 1701002S045 | A | 41 | M | 2 | 3 | 6 | - | | |
| 1701002S047 | A | 69 | M | 2 | 6 | 18 | - | | |
| 1701002S002 | B | 48 | M | 2 | 14 | 21 | - | | |
| 1701002S003 | B | 70 | F | 2 | 11 | 30 | - | | |
| 1701002S005 | B | 35 | M | 2 | 4 | 9 | - | | |
| 1701002S006 | B | 37 | M | 2 | 10 | 15 | - | | |
| 1701002S007 | B | 35 | F | 2 | 10 | 14 | - | | |
| 1701002S011 | B | 21 | M | 2 | 7 | 12 | - | | |
| 1701002S013 | B | 40 | M | 2 | 7 | 12 | - | | |
| 1701002S017 | B | 28 | M | 2 | 5 | 10 | - | | |
| 1701002S018 | B | 43 | M | 2 | 20 | 28 | - | | |
| 1701002S020 | B | 40 | M | 2 | 8 | 9 | - | | |
| 1701002S023 | B | 44 | M | 2 | 6 | 19 | - | | |
| 1701002S025 | B | 23 | M | 2 | 5 | 12 | - | | |
| 1701002S027 | B | 59 | M | 2 | 5 | 22 | - | | |
| 1701002S028 | B | 47 | M | 2 | 3 | 11 | - | | |
| 1701002S029 | B | 46 | M | 2 | 3 | 11 | - | | |
| 1701002S031 | B | 45 | F | 2 | 4 | 11 | - | | |
| 1701002S032 | B | 55 | M | 2 | 4 | 11 | - | | |
| 1701002S034 | B | 29 | M | 2 | 2 | 11 | - | | |
| 1701002S037 | B | 56 | M | 2 | 5 | 10 | - | | |
| 1701002S040 | B | 53 | F | 2 | 3 | 10 | - | | |
| 1701002S044 | B | 50 | M | 2 | 5 | 8 | - | | |
| 1701002S046 | B | 34 | M | 2 | 5 | 7 | - | | |
| 1701002S048 | B | 33 | F | 2 | 5 | 12 | - | | |
| 1701002S049 | B | 48 | M | 2 | 6 | 14 | - | | |
| 1701002S050 | B | 29 | M | 2 | 3 | 8 | - | | |
| 1701003S004 | A | 20 | M | 3 | 8 | 11 | - | | |
| 1701003S005 | A | 22 | M | 3 | 10 | 11 | - | | |
| 1701003S007 | A | 43 | M | 3 | 9 | 13 | - | | |
| 1701003S009 | A | 37 | M | 3 | 9 | 12 | - | | |
| 1701003S010 | A | 44 | M | 3 | 9 | 11 | - | | |
| 1701003S011 | A | 43 | M | 3 | 9 | 11 | - | | |
| 1701003S012 | A | 55 | M | 3 | 9 | 10 | - | | |
| 1701003S015 | A | 40 | F | 3 | 9 | 10 | - | | |
| 1701003S017 | A | 55 | F | 3 | 8 | 12 | - | | |
| 1701003S018 | A | 59 | M | 3 | 11 | 15 | - | | |
| 1701003S021 | A | 55 | M | 3 | 9 | 11 | - | | |
| 1701003S023 | A | 31 | M | 3 | 7 | 8 | - | | |
| 1701003S024 | A | 20 | F | 3 | 9 | 10 | - | | |
| 1701003S025 | A | 57 | F | 3 | 9 | 11 | - | | |
| 1701003S029 | A | 44 | M | 3 | 7 | 10 | - | | |
| 1701003S002 | B | 40 | M | 3 | 11 | 12 | - | | |
| 1701003S003 | B | 35 | M | 3 | 28 | 31 | - | | |
| 1701003S006 | B | 21 | M | 3 | 11 | 11 | - | | |
| 1701003S008 | B | 52 | M | 3 | 11 | 15 | - | | |
| 1701003S013 | B | 42 | M | 3 | 12 | 14 | - | | |
| 1701003S014 | B | 48 | M | 3 | 8 | 14 | - | | |
| 1701003S016 | B | 49 | M | 3 | 9 | 11 | -- | | |
| 1701003S019 | B | 21 | M | 3 | 7 | 9 | - | | |
| 1701003S020 | B | 40 | F | 3 | 9 | 11 | - | | |
| 1701003S022 | B | 37 | M | 3 | 11 | 18 | - | | |
| 1701003S026 | B | 62 | M | 3 | 11 | 14 | - | | |
| 1701003S027 | B | 24 | F | 3 | 10 | 11 | - | | |
| 1701003S028 | B | 45 | F | 3 | 10 | 12 | - | | |
| 1701003S030 | B | 43 | M | 3 | 9 | 20 | - | | |
| 1701001S003 | A | 52 | F | 1 | 8 | 23 | Disqualified for PP | | |
| 1701002S014 | A | 62 | M | 2 | 3 | 11 | Disqualified for PP | | |
| 1701003S001 | B | 67 | F | 3 | 13 | 14 | Disqualified for PP | | |
| 1701001S046 | A | 46 | M | 1 | - | - | Disqualified for ITT & PP | | |
| 1701002S024 | A | 32 | M | 2 | - | - | Disqualified for ITT & PP | | |
| 1701001S012 | B | 45 | M | 1 | - | - | Disqualified for ITT & PP | | |
|  | Randomization Group A (ADD ON)=AYUSH 64 PLUS STANDARD OF CARE | | | | | | | | |
|  | Randomization Group B = Standard of care | | | |  |  | | | |

**TABLE 2: AYUSH 64 DRUG TRIAL- RAW DATA OF STUDY PARTICIPANTS (N=140) FOR ADVERSE EVENTS: A RANDOMIZED CONTROLLED STUDY COMPARING AYUSH 64 PLUS STANDARD OF CARE AND STANDARD OF CARE IN MILD-MODERATE COVID-19**

| **Sub No** | **Randomization group** | **AE Term** | **Start Date** | **End Date** |
| --- | --- | --- | --- | --- |
| 1701003S012 | 2 | DECREASED SPO2 | 01.07.2020 | 28.07.2020 |
| 1701003S040 | 2 | MALARIA (P. Vivax) | 10.07.2020 | 17.-7.2020 |
| 1701001S046 | 1 | BILATERAL LOWER LIMB WEAKNESS WITH TINGLING SENSATION AND NUMBNESS (? GBS) | 16.07.2020 | ONGOING ( STUDY DURATION COMPLETED) |
| 1701001S059 | 1 | DECREASED SPO2 | 24.07.2020 | 01.08.2020 |
| 1701001S013 | 1 | HIGH BLOOD GLUCOSE LEVELS (FBS=233.4) | 27.07.2020 | ONGOING ( STUDY DURATION COMPLETED) |
| 1701001S069 | 2 | DECREASED SPO2 | 31.07.2020 | 05.08.2020 |
| 1701003S020 | 2 | HIGH BLOOD GLUCOSE LEVELS (FBS=221) | 31.07.2020 | ONGOING ( STUDY DURATION COMPLETED) |
| 1701001S061 | 1 | ABDOMINAL DISCOMFORT | 03.08.2020 | 06.08.2020 |
| 1701001S061 | 1 | INCREASED BLOOD PRESSURE (148/100 MM OF HG) | 03.08.2020 | ONGOING ( STUDY DURATION COMPLETED) |
| 1701001S036 | 2 | GASTRITIS | 04.08.2020 | 07.08.2020 |
|  | 2 | FEVER | 10.08.2020 | 13.08.2020 |
| 1701001S047 | 1 | FEVERISHNESS | 10.08.2020 | 12.08.2020 |
| 1701001S016 | 2 | ABDOMINAL DISCOMFORT | 14.08.2020 | 17.08.2020 |
| 1701001S036 | 2 | RAISED SGOT/SGPT LEVLES | 27.08.2020 | 05.10.2020 |
| 1701001S060 | 2 | High blood glucose levels (FBS=324); HBa1C = 11.9 | 29.08.2020 | ONGOING ( STUDY DURATION COMPLETED) |
|  | 2 | INCREASED TRIGLYCERIDE LEVEL, LDL CHOLESTEROL AND VLDL CHOLESTEROL | 29.08.2020 | ONGOING ( STUDY DURATION COMPLETED) |
| 1701001S061 | 1 | BOWEL DISCOMFORT/ UNSATISFACTORY BOWEL EVACUATION | 16.09.2020 | 18.09.2020 |
| 1701001S058 | 2 | HIGH BOOD GLUCOSE LEVELS ( FBS=138.5) | 17.09.2020 | ONGOING ( STUDY DURATION COMPLETED) |
| 1701001S016 | 2 | HIGH BLOOD GLUCOSE LEVELS | 17.07.2020 | ONGOING ( STUDY DURATION COMPLETED) |
| 1701001S031 | 1 | FEVERISHNESS | 15.09.2020 | 17.09.2020 |
| 1701001S019 | 1 | FEVERISHNESS | 10.09.2020 | 12.09.2020 |
| 1701001S037 | 1 | EPIGASTRIC PAIN | 09.09.2020 | 11.09.2020 |
| 1701001S037 | 1 | HYPERACIDITY ( PAIN IN ABDOMEN WITH RETROSTERNAL BURNING) | 29.07.2020 | 30.07.2020 |
| 1701001S037 | 1 | vertigo - mild, intermittently 5- 6 days after discharge | 30.07.2020 | 09.09.2020 |
| 1701001S055 | 2 | BURNING MICTURITION | 11.09.2020 | 14.09.2020 |
| 1701001S036 | 2 | FEVER WITH CHILLS, HEADACHE, VOMITING AND ABDOMINAL DISCOMFORT ( MALARIA - P VIVAX POSITIVE) | 12.09.2020 | 16.09.2020 |
| 1701001S047 | 1 | FEVER WITH CHILLS WITH HEADACHE | 09.10.2020 | 11.10.2020 |
| 1701001S047 | 1 | HIGH BLOOD GLUCOSE LEVELS ( FBS= 132/ PPBS =250.1) | 12.10.2020 | ONGOING ( STUDY DURATION COMPLETED) |
| 1701001S055 | 2 | FEVERISHNESS WITH GEN. BODYACHE AND GE. WEAKNESS | 06.10.2020 | 09.10.2020 |
| 1701001S053 | 1 | HIGH BLOOD GLUCOSE LEVELS ( RISING TREND/ UNCONTROLLED) | 08.10.2020 | ONGOING ( STUDY DURATION COMPLETED) |
| 1701001S044 | 1 | FEVER WITH CHILLS | 10.10.2020 | 13.10.2020 |
| 1701001S044 | 1 | HIGH BLOOD GLUCOSE LEVELS ( UNCONTROLLED) | 14.10.2020 | ONGOING ( STUDY DURATION COMPLETED) |
| 1701001S043 | 1 | HIGH BLOOD GLUCOSE LEVELS ( RISING TREND/ UNCONTROLLED) | 11.08.2020 | ONGOING ( STUDY DURATION COMPLETED) |
| 1701001S017 | 2 | HIGH BLOOD GLUCOSE LEVELS ( RISING TREND/ UNCONTROLLED) | 24.09.2020 | ONGOING ( STUDY DURATION COMPLETED) |
| 1701001S059 | 1 | HIGH BLOOD GLUCOSE LEVELS ( RISING TREND/ UNCONTROLLED) | 13.10.2020 | ONGOING ( STUDY DURATION COMPLETED) |
| 1701001S019 | 1 | GEN. BURNING SENSATION WITH LOOSE STOOLS ( 3-4 TIMES/DAY) | 27.07.2020 | 01.08.2020 |
| 1701001S021 | 2 | HIGH BLOOD GLUCOSE LEVELS ( FBS = 128.4) | 28.09.2020 | ONGOING ( STUDY DURATION COMPLETED) |
| 1701001S027 | 1 | semi loose stools ( 3 - 4 times daily) | 14/07/2020 | 08-04-2020 |
|  |  |  |  |  |
| **Sub No** | **Randomisation Group** | **AE term** | **Start Date** | **End Date** |
| 1701003S004 | 1 | Headache | 2 days | Not Known |
| 1701003S007 | 1 | Hyper acidity | 3 days | Not Known |
| 1701003S009 | 1 | Headache | 3 days | Not Known |
| 1701003S017 | 1 | Loose motions | 3 days | Not Known |
| 1701003S025 | 1 | Weakness | 11-10-2020 | 15-10-2020 |
| 1701003S029 | 1 | Headache | 3 days | Not Known |
| 1701003S001 | 2 | Weakness | 07-08-2020 | 15-07-2020 |
| 1701003S003 | 2 | Cellulitis | 05-07-2020 | 23-07-2020 |
|  | 2 | Weakness | 27.07.2020 | 05.08.2020 |
| 1701003S008 | 2 | Weakness | 19-07-2020 | 25-07-2020 |
| 1701003S013 | 2 | Weakness | 27-07-2020 | 08-02-2020 |
| 1701003S022 | 2 | Neck pain | 2 days | Not Known |
| 1701003S026 | 2 | Cough | 3 days | Not Known |
| 1701003S028 | 2 | Backache | 7 days | Ongoing (Study duration completed) |
| 1701003S030 | 2 | Earache | 12-10-2020 | 17-10-2020 |
|  |  |  |  |  |
| **Sub No** | **Randomisation Group** | **AE term** | **Start Date** | **End Date** |
| 1701002S039 | 1 | weakness | reported at WEEK 4 visit | Not Known |
|  | 1 | itching | reported at WEEK 8 visit | Not Known |
| 1701002S012 | 1 | Back pain | reported at RANDOMIZATION VISIT | Not Known |
|  | 1 | vertigo | reported atRANDOMIZATION VISIT | Not Known |
|  | 1 | Neck Pain (cervical spondilytis) | reported at WEEK 8 visit | Not Known |
|  | 1 | Neck Pain (cervical spondilytis) | reported at WEEK 12 visit | OnGoing -Study completed |
|  | 1 | ankle pain | reported at WEEK 12 visit | OnGoing -Study completed |
| 1701002S015 | 1 | Breathlessness (dyspnoea) | reported at WEEK 4 visit | Not Known |
| 1701002S019 | 1 | joint pain | reported at WEEK 4 visit | Not Known |
|  | 1 | myalgia | reported at WEEK 4 visit | Not Known |
|  | 1 | constipation | reported at WEEK 12 visit | Not Known |
| 1701002S030 | 1 | diarrhoea | reported at WEEK 4 visit | Not Known |
|  | 1 | Breathlessness (dyspnoea) | reported at WEEK 4 visit | Not Known |
|  | 1 | myalgia | reported at WEEK 8 visit | Not Known |
| 1701002S036 | 1 | Breathlessness (dyspnoea) | reported at WEEK 4 visit | Not Known |
|  | 1 | eczema | reported at WEEK 4 visit | Not Known |
|  | 1 | Breathlessness (dyspnoea) | reported at WEEK 8 visit | Not Known |
|  | 1 | pain in legs | reported at WEEK 12 visit | Not Known |
| 1701002S039 | 1 | cough | reported at WEEK 4 visit | Not Known |
|  | 1 | Breathlessness (dyspnoea) | reported at WEEK 8 visit | Not Known |
| 1701002S041 | 1 | Breathlessness (dyspnoea) | reported at WEEK 4 visit | Not Known |
| 1701002S047 | 1 | soreness of throat | reported at WEEK 4 visit | Not Known |
| 1701002S003 | 2 | abdominal pain | 16/07/2020 | Breathlessness |
|  | 2 | Breathlessness (dyspnoea) | 16/07/2020 | 31/07/2020 |
|  | 2 | Breathlessness (dyspnoea) | 14/08/2020 | 31/08/2020 |
| 1701002S005 | 2 | cough | reported at WEEK 4 visit | Not Known |
|  | 2 | weakness | reported at WEEK 4 visit | Not Known |
|  | 2 | weakness | reported at WEEK 8 visit | Not Known |
| 1701002S007 | 2 | DIARRHOEA | reported at WEEK 4 visit | Not Known |
|  | 2 | Myalgia | reported at WEEK 4 visit | Not Known |
|  | 2 | CHILLS | reported at WEEK 12 visit | ONGOING ( STUDY DURATION COMPLETED) |
|  | 2 | MYALGIA | reported at WEEK 12 visit | ONGOING ( STUDY DURATION COMPLETED) |
| 1701002S018 | 2 | myalgia | reported at WEEK 8 visit | Not Known |
|  | 2 | myalgia | reported at WEEK 12 visit | ONGOING ( STUDY DURATION COMPLETED) |
| 1701002S020 | 2 | WHITE PATCHES ON SKIN OF HANDS | reported at WEEK 8 visit | Not Known |
| 1701002S023 | 2 | sore throat | reported at WEEK 4 visit | Not Known |
| 1701002S027 | 2 | myalgia | reported at WEEK 4 visit | Not Known |
|  | 2 | myalgia | reported at WEEK 8 visit | Not Known |
| 1701002S028 | 2 | Breathlessness (dyspnoea) | reported at WEEK 12 visit | ONGOING ( STUDY DURATION COMPLETED) |
| 1701002S032 | 2 | constipation | reported at WEEK 12 visit | ONGOING ( STUDY DURATION COMPLETED) |
|  | 2 | myalgia, sore throat | reported at WEEK 4 visit | Not Known |
|  | 2 | sore throat | reported at WEEK 12 visit | ONGOING ( STUDY DURATION COMPLETED) |
| 1701002S040 | 2 | Breathlessness (dyspnoea), sore throat | reported at WEEK 8 visit | Not Known |
|  | 2 | fatigue, breathlessness | reported at WEEK 12 visit | ONGOING ( STUDY DURATION COMPLETED) |
| 1701002S049 | 2 | smell loss, sorethroat, loss of taste, breathlessness | reported at WEEK 4 visit |  |
|  | 2 | myalgia, sore throat | reported at WEEK 12 visit | ONGOING ( STUDY DURATION COMPLETED) |

**TABLE 3: AYUSH 64 DRUG TRIAL- RAW DATA OF STUDY PARTICIPANTS (N=140) FOR WITHDRAWAL AND DROP OUTS: A RANDOMIZED CONTROLLED STUDY COMPARING AYUSH 64 PLUS STANDARD OF CARE AND STANDARD OF CARE IN MILD-MODERATE COVID-19**

| Sr. No | Sub No | Control/Test (C/T) | Reason for Drop Out/Discontinuation | Time Withdrawn |
| --- | --- | --- | --- | --- |
| 1 | 1701001S025 | Test (Add on) | Lost to follow up (Repeated calls for follow up not attended) | Post Hospital Discharge |
| 2 | 1701001S046 | Test (Add on) | Withdrawn from study due to Occurrence of AE (not related to study drug) (GBS?) | Post Randomization |
| 3 | 1701001S065 | Test (Add on) | Lost to follow up (Repeated calls for follow up not attended) | 12 Week |
| 4 | 1701001S051 | Test (Add on) | Lost to follow up (Repeated calls for follow up not attended) | Post Hospital Discharge |
| 5 | 1701002S001 | Test (Add on) | Lost to follow up (Repeated calls for follow up not attended) | Post Hospital Discharge |
| 6 | 1701002S008 | Test (Add on) | Lost to follow up (Repeated calls for follow up not attended) | Post Hospital Discharge |
| 7 | 1701002S024 | Test (Add on) | Withdrew consent (Not willing to participate) Did not take study drug | Post Randomization |
| 8 | 1701002S042 | Test (Add on) | Lost to follow up (Repeated calls for follow up not attended) | Post Hospital Discharge |
| 9 | 1701001S009 | Control (SOC) | Lost to follow up (Repeated calls for follow up not attended) | Post Hospital Discharge |
| 10 | 1701001S012 | Control (SOC) | Withdrawn from Study due to Occurrence of SAE (not related to study drug) (Decreased SPO2 levels) | Post Randomization |
| 11 | 1701001S020 | Control (SOC) | Lost to follow up (Repeated calls for follow up not attended) | 8 Week |
| 12 | 1701001S029 | Control (SOC) | Lost to follow up (Repeated calls for follow up not attended) | 4 Week |
| 13 | 1701001S033 | Control (SOC) | Lost to follow up (Repeated calls for follow up not attended) | 4 Week |
| 14 | 1701001S038 | Control (SOC) | Lost to follow up (Repeated calls for follow up not attended) | 12 Week |
| 15 | 1701001S040 | Control (SOC) | Lost to follow up (Repeated calls for follow up not attended) | 4 Week |
| 16 | 1701001S068 | Control (SOC) | Lost to follow up (Repeated calls for follow up not attended) | 12 Week |
| 17 | 1701002S034 | Control (SOC) | Lost to follow up (Repeated calls for follow up not attended) | Post Hospital Discharge |
| 18 | 1701002S006 | Control (SOC) | Lost to follow up (Repeated calls for follow up not attended) | Post Hospital Discharge |
| 19 | 1701002S044 | Control (SOC) | Lost to follow up (Repeated calls for follow up not attended) | Post Hospital Discharge |
| 20 | 1701003S002 | Control (SOC) | Lost to follow up (Repeated calls for follow up not attended) | 8 Week |
