# Supplementary material for: Co-administration of AYUSH 64 as an adjunct to standard of care in mild and moderate COVID-19: A randomized, controlled, multicentric clinical trial
Source: PLoS One. 2023 Mar 16;18(3):e0282688. doi: 10.1371/journal.pone.0282688 (PMC10019690; doi:10.1371/journal.pone.0282688)
Supplement: Attachment — Submitted filename: Response to Editor_Referee.docx [file pone.0282688.s009.docx]

**RESPONSE TO COMMENTS FROM THE EDITOR AND REVIEWERS:**

1.	Please ensure that your manuscript meets PLOS ONE's style requirements, including those for file naming. The PLOS ONE style templates can be found at 
[https://journals.plos.org/plosone/s/file?id=wjVg/PLOSOne_formatting_sample_main_body.pdf](https://nam12.safelinks.protection.outlook.com/?url=https%3A%2F%2Fjournals.plos.org%2Fplosone%2Fs%2Ffile%3Fid%3DwjVg%2FPLOSOne_formatting_sample_main_body.pdf&data=05%7C01%7C%7C9d5905228d12432e08c808da449198c9%7C84df9e7fe9f640afb435aaaaaaaaaaaa%7C1%7C0%7C637897689419727070%7CUnknown%7CTWFpbGZsb3d8eyJWIjoiMC4wLjAwMDAiLCJQIjoiV2luMzIiLCJBTiI6Ik1haWwiLCJXVCI6Mn0%3D%7C3000%7C%7C%7C&sdata=yqZxBZCAlJ9KVqSzceF7HNFY%2Fod7aXSjPHUa%2BXI%2FDZA%3D&reserved=0) and 
[https://journals.plos.org/plosone/s/file?id=ba62/PLOSOne_formatting_sample_title_authors_affiliations.pdf](https://nam12.safelinks.protection.outlook.com/?url=https%3A%2F%2Fjournals.plos.org%2Fplosone%2Fs%2Ffile%3Fid%3Dba62%2FPLOSOne_formatting_sample_title_authors_affiliations.pdf&data=05%7C01%7C%7C9d5905228d12432e08c808da449198c9%7C84df9e7fe9f640afb435aaaaaaaaaaaa%7C1%7C0%7C637897689419727070%7CUnknown%7CTWFpbGZsb3d8eyJWIjoiMC4wLjAwMDAiLCJQIjoiV2luMzIiLCJBTiI6Ik1haWwiLCJXVCI6Mn0%3D%7C3000%7C%7C%7C&sdata=N1FAZQLD%2BPu7Skhb7ntJUXgvZvPGMQoyTvczG1QoceE%3D&reserved=0)

**Response**: The manuscript was revised with due adherence to the style and other guidelines contained in the instructions to the authors and above mentioned references.

2. We note that the grant information you provided in the ‘Funding Information’ and ‘Financial Disclosure’ sections do not match.

When you resubmit, please ensure that you provide the correct grant numbers for the awards you received for your study in the ‘Funding Information’ section.

**Response:** Both the funding information and financial disclosure section have been revised in view of consistency and further description. The grant reference of the study issued by CCRAS/Ministry of AYUSH Government of India is added.

3. Thank you for stating the following in the Competing Interests section: "None of the authors have any financial conflict of interest regarding this study. The authors declare their relationship related to study as per the International Committee of Medical Journal Editors. Bhushan Patwardhan is Chairman, Interdisciplinary Ayush Research and Development Task Force on Covid-19. Kuldeep Chaudhary, Alok Srivastava, Govind Reddy, Manohar Gundeti, BCS Rao, Babita Yadav, Narayanam Srikanth work in Central Council for Research in Ayurvedic Sciences (CCRAS), Ministry of AYUSH (MoA), Government of India (GOI), New Delhi. Ashwinikumar Raut was a consultant for the study. Sanjay Tamoli was involved as a CRO. AYUSH-64 is a proprietary formulation of CCRAS."

Please confirm that this does not alter your adherence to all PLOS ONE policies on sharing data and materials, by including the following statement: "This does not alter our adherence to  PLOS ONE policies on sharing data and materials.” (as detailed online in our guide for authors [http://journals.plos.org/plosone/s/competing-interests](https://nam12.safelinks.protection.outlook.com/?url=http%3A%2F%2Fjournals.plos.org%2Fplosone%2Fs%2Fcompeting-interests&data=05%7C01%7C%7C9d5905228d12432e08c808da449198c9%7C84df9e7fe9f640afb435aaaaaaaaaaaa%7C1%7C0%7C637897689419727070%7CUnknown%7CTWFpbGZsb3d8eyJWIjoiMC4wLjAwMDAiLCJQIjoiV2luMzIiLCJBTiI6Ik1haWwiLCJXVCI6Mn0%3D%7C3000%7C%7C%7C&sdata=PctSbaWv3pgbhlGVUKY%2F%2BUM53%2BZH4pOdG1CSLGocFg0%3D&reserved=0)).  If there are restrictions on sharing of data and/or materials, please state these. Please note that we cannot proceed with consideration of your article until this information has been declared.

Please include your updated Competing Interests statement in your cover letter; we will change the online submission form on your behalf.

**Response:** The relevant sections have been carefully revised and also contain the required statement on adherence to PLOS ONE policies. Efficacy and adverse event data is now enclosed as a supporting document as per the minimal data sharing policy. Current limitations on data sharing and future access to all data is also described in the relevant section.

A mention of above is also made in the covering letter.

4. In your Data Availability statement, you have not specified where the minimal data set underlying the results described in your manuscript can be found. PLOS defines a study's minimal data set as the underlying data used to reach the conclusions drawn in the manuscript and any additional data required to replicate the reported study findings in their entirety. All PLOS journals require that the minimal data set be made fully available. For more information about our data policy, please see [http://journals.plos.org/plosone/s/data-availability](https://nam12.safelinks.protection.outlook.com/?url=http%3A%2F%2Fjournals.plos.org%2Fplosone%2Fs%2Fdata-availability&data=05%7C01%7C%7C9d5905228d12432e08c808da449198c9%7C84df9e7fe9f640afb435aaaaaaaaaaaa%7C1%7C0%7C637897689419727070%7CUnknown%7CTWFpbGZsb3d8eyJWIjoiMC4wLjAwMDAiLCJQIjoiV2luMzIiLCJBTiI6Ik1haWwiLCJXVCI6Mn0%3D%7C3000%7C%7C%7C&sdata=8%2FCQdHZz1Rh1uPVZOUPet3RzzC9xAHuyATsv9vulSRY%3D&reserved=0).

Upon re-submitting your revised manuscript, please upload your study’s minimal underlying data set as either Supporting Information files or to a stable, public repository and include the relevant URLs, DOIs, or accession numbers within your revised cover letter. For a list of acceptable repositories, please see [http://journals.plos.org/plosone/s/data-availability#loc-recommended-repositories](https://nam12.safelinks.protection.outlook.com/?url=http%3A%2F%2Fjournals.plos.org%2Fplosone%2Fs%2Fdata-availability%23loc-recommended-repositories&data=05%7C01%7C%7C9d5905228d12432e08c808da449198c9%7C84df9e7fe9f640afb435aaaaaaaaaaaa%7C1%7C0%7C637897689419727070%7CUnknown%7CTWFpbGZsb3d8eyJWIjoiMC4wLjAwMDAiLCJQIjoiV2luMzIiLCJBTiI6Ik1haWwiLCJXVCI6Mn0%3D%7C3000%7C%7C%7C&sdata=5hq7rLNDhuEh0SBHYPNVeLLeDXUolcRhvgMcaHu1D20%3D&reserved=0). Any potentially identifying patient information must be fully anonymized.

Important: If there are ethical or legal restrictions to sharing your data publicly, please explain these restrictions in detail. Please see our guidelines for more information on what we consider unacceptable restrictions to publicly sharing data: [http://journals.plos.org/plosone/s/data-availability#loc-unacceptable-data-access-restrictions](https://nam12.safelinks.protection.outlook.com/?url=http%3A%2F%2Fjournals.plos.org%2Fplosone%2Fs%2Fdata-availability%23loc-unacceptable-data-access-restrictions&data=05%7C01%7C%7C9d5905228d12432e08c808da449198c9%7C84df9e7fe9f640afb435aaaaaaaaaaaa%7C1%7C0%7C637897689419727070%7CUnknown%7CTWFpbGZsb3d8eyJWIjoiMC4wLjAwMDAiLCJQIjoiV2luMzIiLCJBTiI6Ik1haWwiLCJXVCI6Mn0%3D%7C3000%7C%7C%7C&sdata=CkCbmmD5bBHY1EzM80Vvpe3UOBSFBxUISRHsf9St4jc%3D&reserved=0). Note that it is not acceptable for the authors to be the sole named individuals responsible for ensuring data access.

We will update your Data Availability statement to reflect the information you provide in your cover letter.

**Response**: The data availability section now contains updated information on access to study data. Minimal data set to support the efficacy outcome and adverse events is now enclosed as a supporting document.

This is also mentioned in the covering letter.

5. We note that you have included the phrase “data not shown” in your manuscript. Unfortunately, this does not meet our data sharing requirements. PLOS does not permit references to inaccessible data. We require that authors provide all relevant data within the paper, Supporting Information files, or in an acceptable, public repository. Please add a citation to support this phrase or upload the data that corresponds with these findings to a stable repository (such as Figshare or Dryad) and provide and URLs, DOIs, or accession numbers that may be used to access these data. Or, if the data are not a core part of the research being presented in your study, we ask that you remove the phrase that refers to these data.

**Response:** The phrase ‘data not shown’ has been deleted from the manuscript with appropriate modification of the relevant text. Supporting files are now upgraded to contain all relevant data to support all observations and results in the main text.

6. Your ethics statement should only appear in the Methods section of your manuscript. If your ethics statement is written in any section besides the Methods, please delete it from any other section.

**Response:** The ethics statement is now shifted to the methods section.

**Comments to the Author**

1. Is the manuscript technically sound, and do the data support the conclusions?

The manuscript must describe a technically sound piece of scientific research with data that supports the conclusions. Experiments must have been conducted rigorously, with appropriate controls, replication, and sample sizes. The conclusions must be drawn appropriately based on the data presented.

Reviewer #1: Partly

Reviewer #2: Yes

**Response:** The manuscript is appropriately revised to strengthen the study data and conclusions.

2. Has the statistical analysis been performed appropriately and rigorously?

Reviewer #1: I Don't Know

Reviewer #2: Yes

**Response:** Section on statistical design and analysis has been revised and made more comprehensive. The statistical results are now described with greater detail. Some more statistical analysis was carried out and results added as per the advice of the Reviewers.

3. Have the authors made all data underlying the findings in their manuscript fully available?

The [PLOS Data policy](https://nam12.safelinks.protection.outlook.com/?url=http%3A%2F%2Fwww.plosone.org%2Fstatic%2Fpolicies.action%23sharing&data=05%7C01%7C%7C9d5905228d12432e08c808da449198c9%7C84df9e7fe9f640afb435aaaaaaaaaaaa%7C1%7C0%7C637897689419727070%7CUnknown%7CTWFpbGZsb3d8eyJWIjoiMC4wLjAwMDAiLCJQIjoiV2luMzIiLCJBTiI6Ik1haWwiLCJXVCI6Mn0%3D%7C3000%7C%7C%7C&sdata=B%2FVHD9IUWgEiDgdOboW9aYhVXFxdkdJ0HizjrtXY4Lw%3D&reserved=0) requires authors to make all data underlying the findings described in their manuscript fully available without restriction, with rare exception (please refer to the Data Availability Statement in the manuscript PDF file). The data should be provided as part of the manuscript or its supporting information, or deposited to a public repository. For example, in addition to summary statistics, the data points behind means, medians and variance measures should be available. If there are restrictions on publicly sharing data—e.g. participant privacy or use of data from a third party—those must be specified.

Reviewer #1: No

Reviewer #2: Yes

**Response**: Raw data to support the efficacy analysis and adverse events is now enclosed as a supporting file.

4. Is the manuscript presented in an intelligible fashion and written in standard English?

PLOS ONE does not copyedit accepted manuscripts, so the language in submitted articles must be clear, correct, and unambiguous. Any typographical or grammatical errors should be corrected at revision, so please note any specific errors here.

Reviewer #1: Yes

Reviewer #2: No

**Response:** The revision has been checked for English Language use by a professional in native English language. The corrections are marked in a revision file.

Editor: Upon re-submitting your revised manuscript, please upload your study’s minimal underlying data set as either Supporting Information files or to a stable, public repository and include the relevant URLs, DOIs, or accession numbers within your revised cover letter

**Response:** Minimal data set is uploaded as a supporting document. A new description with details of contact to obtain further access is also provided under section on ‘data availability’

**Reviewer #1**:

1)The authors do not provide line numbers, so it is quite tedious to give specific edits. However, there are a good number of minor errors in English language usage throughout the manuscript. Given the standard to which the authors aspire, these should be corrected. There are verb tense issues in several places as well.

**Response:** The line numbers are now provided in the revision. The entire text is reviewed by a native English language person and errors in grammar etc corrected.
2) The authors do not provide raw data for evaluation. This seems indicated in what could be thought of as a somewhat contentious area.
**Response:** This is now provided as a supporting document and further description for access to data also mentioned in the section on ‘data availability’.

3)The authors write "drugs such as hydroxychloroquine fell into disrepute". This is needless editorializing on a matter where there appears to be at least some good faith disagreement. There are certainly many western authors who would say the same about non-western systems of medicine, so it seems pointless to engage in such statements. It seems ironic that hydrochloroquine is then listed as part of standard of care later.
**Response:** The point is well taken and the sentence modified to indicate that the widespread use of HCQS in the earlier part of the pandemic in India was grossly restricted when several drug trials did not shown unequivocal evidence. Since submission of this manuscript in mid 2021, the Government of India has removed HCQS from the ‘standard of care’ and the same is now reflected in the revision.

4) The authors write "It is now known that the exuberant and dysregulated immune response in COVID-19 leads to life threatening complications." Please provide citations and appropriately scope this statement with an estimate of the incidence of these in general populations.

**Response:** A citation is provided. There are no truly suitable prospective population studies to estimate the incidence of severe or life-threatening complications in the general population. However, we provide an estimate from hospital-based studies and a recent study published in PLOS ONE [: Grant MC, Geoghegan L, Arbyn M, Mohammed Z, McGuinness L, Clarke EL, et al. (2020) The prevalence of symptoms in 24,410 adults infected by the novel coronavirus (SARSCoV-2; COVID-19): A systematic review and meta-analysis of 148 studies from 9 countries. PLoS ONE 15(6): e0234765].

5) The authors write "We used manually calculated raw scores (summation) for each domain". Why is "manually" stipulated here? Were computations performed by individuals and then entered into case report forms (CRFs)?
**Response**: This was with reference to WHO Quality of Life Instrument- BREF. We followed the instructions contained in the WHO publication for calculating the domain and total score. Reference is provided in the main text. There were 27 questions belonging to 4 major domains. Each question was answered using a 5-point Likert response scale. Each of the 5 points of response (such as very poor, poor, neither poor nor good, good, and very good) were scored 1 to 5 - 1 was equivalent to very poor and 5 was equivalent to very good. The score for 3 questions reversed to follow the ascendancy order of the remaining questions so that higher scores meant better quality of life. A hard copy of the questionnaire was provided to each of the study subject for completion as per time points in the study protocol. The subject was asked to choose the likely response to each question and place a tick mark in the adjacent box showing the score of the response. The score of each question was added by the designated paramedic manually to calculate the score for each domain (manually calculated sum of raw scores in each domain); recorded in the questionnaire. The score for each domain and total for each participant was entered into the Excel based data sheet.

6)The authors write "A convenience study sample of 140 participants was finalized by AC and SS and considered adequate to address the study research questions." This sounds quite ad hoc. What process was used to establish the convenience sample? What sampling biases were introduced by this process?
**Response:** We have rewritten the section.

To find possible answers to address the study research questions and considering the logistics, a convenience study sample of 140 participants was felt to be adequate by AC (first author) and SS (Biostatistician co-author)”. This was also on the basis of available time & resources and relevant experience available then to carry out this trial. No formal estimation of required sample size for this study was attempted.

7) The statistical methods are described poorly, with a mix of jargon and software thrown together. Please describe the study endpoints and their analyses a little more clearly, perhaps grouping as seems fit to save tedious listing.
**Response:** Please see the answer above in question No 6 above

8)The actual structure of the analysis of variance (ANOVA) performed is unclear. Also, see below. The authors should provide a description that includes fixed effects, interaction terms, random effects (if any), and R-side correlation (if any). Linear model analysis must be accompanied by an assessment of model fit diagnostics, and corrective measures should be described in advance and described in the methods as necessary. All contrasts or estimates to be obtained should be described.

**Response:** The statistical analysis and results are revised and shown in Table 2 and the primary efficacy measure result was revised as per the original plan. Being a 2 arm study, we adhere to the Student’s ‘t- test for independent samples and Mann Whitney statistic. However, to respond to the ‘random effect’ of sites, we have performed a general linear (mixed effect ) model for the primary efficacy measure and enclosed the results (SPSS output) in the supplement file (S5 File).

9) Generally, the analysis should be performed on both an intent-to-treat (ITT) and a per-protocol basis. The ITT analysis could be placed in a supplementary document.
**Response:** The results of an ITT analysis on 137 participants who completed the randomization treatment phase are enclosed in the supplement file (S5 File)

10) Usual format for tables such as Table 1 are to footnote the p-values with the specific tests used.

**Response:** The format for Tables and foot notes is revised.

11)Looking at Table 2 makes it clear that the data follow one of two structures:

* Fixed effect for treatment.
* Random effect for site.
* Potential random interaction of treatment and site.
* Repeated measures in cases where serial measurements are taken on a patient over time.
This structure can be handled easily within the linear mixed effects model framework. A consistent model should be used in all cases. If repeated measures are used, probably an unstructured correlation matrix will be most sensible and most flexible.
**Response:** Please see the answer to Question No 8. We have enclosed the results of a general linear (mixed effects) model.

12) In Table 3 the authors use the footnote "*" to indicate "no difference"---the same footnote used before to indicate statistical significance. This is pointlessly confusing. Please use the standard approach that everyone else uses when reporting such results.
**Response**: The Tables and foot notes have been revised to ensure uniform method of reporting data and in particular the ‘footnote’

13) In Table 4 the authors note "(anchored with best and worst response)". What does this mean? This procedure is not described in the methods section.
**Response:** The error is regretted. This pertains to a visual analogue scale and a a elaborate description is provided on the scoring technique of the two health and quality of life questionnaires used in the ‘methods section’. Table 4 and S4 File is modified accordingly.

14) Please modify Table 5 to industry norms.
**Response:** The suggestion is now incorporated and Table 5 is revised to show the AE are now classified as per symptom, sign and diagnosis along with the System organ classification .

15) The authors write "Despite serious concerns, the study arms were well matched on several measures including SOC." What are these concerns? Please demonstrate that these concerns are not an issue or please appropriately caveat the results of this trial.
**Response:** The word ‘serious’ was a wrong choice of word, and we acknowledge the error. It should be ‘important concerns. Accordingly, the text has been now been modified. There were several important a-priori concerns for the current drug trial study such as challenges of a drug trial during the upsurge of pandemic, fear and reluctance of COVID patients to participate, delay in the diagnosis and enrolment, different standards of care (COVID-19) at study sites, open label nature of AYUSH 64 intervention and placebo response, bias in the assessment of clinical recovery, and surreptitious use of AYUSH 64 and other Ayurvedic drugs. Several concerns were adequately addressed by the diligence of the study team, randomization procedure and treatment under direct observation (hospitalization phase). The study arms were well matched (Table 1). It seemed prudent to emphasize that hospitalization in the current study was responsible for good compliance and capture of robust data. The latter also made it possible to carry out a daily physical assessment of study participants by a competent COVID-19 physician to identify and endorse clinical recovery in a timely and systematic manner. Intense monitoring was also crucial to document the safety and tolerability of AYUSH 64. Importantly there were no clinically obvious drug interactions with the modern drugs. 16) The authors write "We believe that the current study has boldly addressed the need for evidence-based medicine to treat mild and moderate COVID-19." Doesn't this seem rather overblown?
Response: Yes. In hind sight the statement could be interpreted as an exaggeration. The statement is modified and reads ‘We believe that the current study has been able to provide some degree of evidence based medicine for the treatment mild and moderate COVID-19 (non emergency) using an integrative approach with an Ayurvedic drug (AYUSH 64) combined with standard modern medicine care’.

17) The authors write "Several limitations were imposed by the chaotic and tragic pandemic situation. We encountered uncertainties and often contradictory advice regarding SOC and other COVID-19 related health matters in the social and news media. During the first pandemic year, the patients were reluctant to seek medical care for fear of being stigmatized and this probably delayed the treatment for several patients as shown by the timelines in Table 1. Though, the primary efficacy was assessed by the attending physician in a blinded manner, a placebo response to some extent cannot be ruled out. Ayurveda is endearing to the Indian community. In view of absence of a-priori data, we settled for a convenience sample size for this study." This further points up the need for a *very* clear description of how exactly this sample was selected. This is critical to the manuscript. Aside from that, this paragraph is overly dramatic and chatty. Everyone working in clinical trials had many, many issues during this time.
**Response:** We readily agree with the comment and incorporate the suggestion in the discussion . However, the issue at stake is complex and we have tried to express that in brief. Though it may be general knowledge , it may be appropriate to submit to the reader how challenging and complex drug trials were during the pandemic. The latter was more so during the initial six months or so during which time the current study was launched. It is prudent to add that some issues connected with drug trials were more regional than global. We believe that it is appropriate to state ‘ "Several limitations were imposed by the pandemic situation. We encountered uncertainties and often contradictory advice regarding SOC and other COVID-19 related health matters in the social and news media. During the first pandemic year, the patients were reluctant to seek medical care for fear of being stigmatized and this probably delayed the treatment for several patients as shown by the timelines in Table 1.’ We have further provided an informative reference from a recent PLOS ONE publication [: Chen Z, Chen L, Chen H (2021) The impact of COVID-19 on the clinical trial. PLoS ONE 16(5): e0251410]

With reference to the second half of the paragraph regarding physician centric issues and selection of participants, we acknowledge the need to elaborate the subject of selection of trial participants in the methods section. We first briefly introduce the then Government policy on treatment of COVID-19 which was followed. A new elaborate section on ‘Selection, Screening and Eligibility, and Management’ is now added under the methods section.

A description of the ‘convenience sample size’ used in the current study is provided above under Question number 6 above. This is also clarified under the statistical design and analysis section of the manuscript.

18) Please discuss the following potential limitations in much more detail:
* The study was open label and unblinded.
* The study sample was drawn as a "convenience sample".
Both of these issues have important implications for the interpretability of the results of the trial.

**Response**: Agreed. Both the limitations were mentioned in the discussion section and are now modified for better clarity. The subject of sample size is also mentioned above in Question serial number 6 above.

**Reviewer #2:** The reviewer’s comments on the article, ” Coadministration of AYUSH 64 as an adjunct to Standard of Care in mild and moderate COVID-19: A randomised, controlled, multicentric clinical trial” are as follows:
1) This is a multi-cantered clinical trial on AYUSH 64 as an adjunct to standard of care. The authors concluded that the AYUSH 64 group had shorter time to clinical recovery from randomization, higher proportion of recovery in the first week, and better performance in general health, quality of life, fatigue, anxiety, stress, sleep and other psychosocial metrics.

**Response:** This was the essence of the current study. It was a randomized controlled study. There were limitations too which were mostly related to convenience sample size and non-blinding nature intervention and several difficulties imposed by the COVID-19 pandemic on patient logistics and monitoring. All said and done, we believe that the outcome in favour of the Ayurvedic medicine AYUSH 64 to treat mild and moderate cases of COVID-19 along with standard care is encouraging. There is now community based observational data to support its standalone use also in such uncomplicated COVID-19 situations.

2) In general, native-speaker English editing would be suggested since some sentences/words were difficult to understand. Besides, there were some points needed to explain in detail.
**Response:** The point is well taken. We have corrected the manuscript with expert professional help for several grammatical and other errors and this is visible in the marked revision copy. Further, the sections on introduction, patient selection, trial procedures, and statistical design and analysis have been described in greater detail. Information on Remdesivir and other anti-COVID 19 drugs is also revised. Also, the discussion has been modified accordingly.

3)Abstract
In Results, please mention mean±95% CI days of 2 group.

**Response**: We had incorporate the suggestion in the abstract.
4) Introduction
Reference 7 should be re-indexed. The authors may mention remdesivir, that is a commonly cited repurposing drug for COVID-19.

**Response**: yes. A reference has been added
5) The authors may add some descriptions about the mechanisms that how AYUSH 64 works.

**Response**: A brief description about the likely mechanism of action of AYUSH 64 is added in the discussion .

AYUSH 64 was not a classic Ayurveda formulation but contains medicinal plants described in classic texts and used by Ayurveda practitioners for several centuries in the Indian subcontinent. These medicinal plants were considered therapeutically useful to treat fever, pain and inflammation of varying aetiology and several medical disorders that include cough and breathing disorders and asthma. Several experimental studies, both in vitro and animal, have demonstrated some antiviral (herpes simplex, adenovirus and Chikungunya virus), anti-inflammatory and beneficial immunological effects of the medicinal plants in AYUSH 64. In a more recent in-silico molecular docking study, several ingredients of AYUSH 64 (and Akuammicine N-Oxide from Alstonia scholaris in particular) were found to bind well with the main protease enzyme of the SARS-CoV-2 and may well be an important mechanism of action. An uncontrolled exploratory clinical study of 38 patients suffering from influenza like illness and treated with AYUSH 64 for one week along with standard care showed no safety concerns and uncomplicated recovery and probably a lesser need of paracetamol and anti-allergic drugs.

6) Methods
Is the sentence, “the duration of study was 12 weeks”, meant that for each participant, the treatment interval was 12 weeks? Please make the sentence clear.

**Response**: Thanks for this important observation. Each individual participant completed 12 weeks of study period and we have clarified the same in the text under methods.
7) Since AYUSH 64 is popularly used to treat acute onset febrile respiratory illnesses for over three decades in India, how did the authors prevent the participants to buy AYUSH 64 over the counter or on-line?

**Response:** This is an important comment and we have provided an elaborate description under the ‘strengths and limitations’ section of the discussion.

We were aware of a possibility of confounding by unauthorized use of drugs to treat COVID-19 by the study participants. All patients were counselled in detail prior to enrolment. A strict vigil and close monitoring during hospitalization phase was ensured by a study dedicated staff as per protocol. Following discharge, a close contact was kept with study participants using a special mobile phone app (see study methods) and telephonic reminders. However, it was difficult to vouch for total avoidance of other Ayurvedic drugs and or AYUSH 64 by study participants especially prior to enrolment or after discharge.

It is prudent to add that as per the Government policy in vogue then, all study participants were admitted in Government run COVID dedicated hospitals for treatment irrespective of the severity of the illness. The latter ensured a strict direct observation of the study participants and compliance with all methods and procedures. Both the intervention groups were well matched for several variables at randomization baseline though our recorded did not show any prior Ayurvedic drug use. The study intervention was unblinded. It is unlikely that any drug other than that prescribed by the current study was used during in-patient treatment. Following discharge, patients were followed till week 12 completion and examined monthly. During the latter phase, patients from the AYUSH 64 plus standard care arm continued AYUSH 64 but not the patients from previous standard arm care.

Ayurvedic drugs and Ashwagandha (Withania somnifera) and Guduchi (Tinospora cordifolia) in particular were used extensively both for prophylaxis and treatment of COVID-19 by the India population. Ayurvedic drugs were available over the counter and did not require a prescription. To begin with, AYUSH 64 was a lesser-known drug for use in COVID-19 in the earlier phase of the pandemic but in time was included in the Ayurveda drug promotional campaign by Government agencies. The current drug trial was begun on 18 June 20 and completed on 25 Oct 20.

We have also described the measures taken by us to safeguard the study.

Several measures were taken to safeguard against a surreptitious use of Ayurvedic drugs in the current study. Participants with a history of prior use of Ayurvedic drugs or any CAM were excluded. As mentioned above, the treatment during hospitalization was directly observed. A strict vigil was maintained by the nursing and paramedical staff who also recorded the consumption of AYUSH 64 as per protocol. Participants were closely monitored and examined during the follow up phase. A special mobile app was used for daily monitoring (see methods section above). Participants were repeatedly counselled against self-treatment. We believe that the surreptitious use of Ayurvedic medicines, if at all, was negligible in the current study and that the unauthorized use of AYUSH 64 was unlikely. On the other hand, the better health related outcome observed in the AYUSH Plus group during the pre-planned follow was indeed encouraging (Table 4).

8)In addition, how did the authors evaluate the compliance of both groups after discharge from hospitals?
**Response:** Patients randomized to AYUSH 64 plus standard care arm continued AYUSH 64 to complete 12 weeks of administration as per protocol. AYUSH 64 was provided free of cost to each patient and consumption monitored. Patients from the controlled arm of standard care continued non-specific treatment with drugs such as vitamins as per physician judgement and were not prescribed AYUSH 64. Patients were examined monthly and for long COVID symptoms and quality of life issues. In addition, patients were closely monitored using telephonic reminders and a specially designed mobile phone app (see methods). Some of the measures are described in the answer to Question No.7 above

9)Why and when did the study results be disseminated to the public through electronic social and print media, described in the section of subheading patient and public involvement?

**Response:** We have incorporated the excellent suggestion.A press release was issued by CCRAS, Ministry of AYUSH, Government of India, in May 2021 after completion of the current study. This was followed by a press meeting with selected leading news media to announce important results of the current study by the first author and other co-authors and experts . The management guidelines of COVID-19 issued by CCRAS from time to time were also updated with the key study results on the efficacy and safety of AYUSH 64. Simultaneously, all the principle investigators informed the outcome of the current study to each participant telephonically and those desirous were called for a meeting with the study physician.

10) In supplementary material, the protocol mentioned 3 regimen: i)AYUSH-64: 500 mg tablet, 2 tablets bid (twice daily) ii)Yashtimadhu: 300 mg tablet, 2 tablets bid (twice daily) iii)Sanshamani Vati Plus: Each tablet to contain 300 mg Guduchi plus 75 mg Pippali, 2 tablets bid (twice daily). Were the regimen ii, and iii used in this study?

**Response:** We regret this confusion caused by the lack of clarity in the beginning of the ‘methods section’. We have now modified the introduction section to describe the initiative of AYUSH CCRAS Government of India under which three potential Ayurvedic drugs including AYUSH 64 were selected for independent randomized controlled drug trial studies using a different study team and study sites. However, there was a common protocol for methods which was registered in the Clinical Trial Registry of India and is enclosed as supplementary file (S2 File). All this is further clarified in the beginning of the ‘methods section’.

Here we only report the drug trial of AYUSH-64.

To complete the answer, the other two drug trial studies were also completed in 2021 but the results are still under review with the sponsor.

11) Results- For CXR, moderate abnormalities in 2% patients in the AYUSH plus and 13% in the SOC were noted. Did the authors think patients in SOC had higher proportion of severe illness than those in AYUSH plus group? On day 28, CXR showed mild abnormalities in 22% patients AYUSH plus and 21% patients SOC. Did the authors think that the residual abnormal CXR findings resolve later, after patients recovered clinically? Did the authors follow up the resolution of CXR findings?

**Response:** We first regret the error in the data caused by lack of stringent review of the earlier radiographic data that was extracted from the case record forms and was not properly recorded in all cases. Also we found post submission, that no XRays were printed in one site and only the screening results were recorded. We thank you for the excellent comment and we now incorporate a more elaborate analysis and description.

Soon after submission, we completed a reassessment of the radiographic evaluation in the current study by a newly appointed independent radiologist. We have now revised the relevant sections pertaining to radiographic assessment.

At the outset, we concede that (i) Chest X-Rays were carried out only for diagnosis of atypical COVID-19 pneumonia and to ensure that there was no worsening on hospital discharge (ii) no standard protocol or scoring system was followed (iii) following discharge, X-Rays were only taken if the patient had a relapse of respiratory symptoms (iv) High resolution computed tomography scan was not taken as a routine or used to confirm resolution and absence of any complication such as pulmonary fibrosis (v) uncomplicated radiographic pneumonitis was labelled as moderate COVID-19 by the radiologist (vi) Digital X-Rays were taken at two sites (Mumbai and Nagpur) only and which are now used for detail analysis and inclusion in the current revised manuscript.

All digital X-Rays (90) were blinded for treatment allocation for the analysis report in the revision.

The revision contained the corrected results shown in S5 File (Supplement file).

In specific response to the Reviewer’s query, we agree that the radiographic severity (definite pneumonitis) of COVID-19 seems to be somewhat more in the standard care group though it may not be of clinical significance. Also, there were other caveat that we explain. We also agree with the reviewer’s observation that radiographic resolutions may take much longer than clinical recovery and we have included a citation from a research study. In hindsight, we should have followed participants with abnormal X-Rays during hospitalization with repeated radiological evaluation to document radiographic resolution, but we were discouraged by small numbers of participants with abnormal X-Ray on discharge and logistics and study budget. Both the groups were well balanced and patients were closely monitored during randomization treatment and during follow up. Only in one case, there was rapid clinical and radiologic deterioration of respiratory status warranting ICU care and oxygen administration and withdrawal from the study-the patient made complete recovery and belonged to standard care intervention arm in the study..

As regards a more serious nature of illness in the SOC group we submit the following statement in the discussion section. But also add that in view of the limitations, the reader needs to exercise caution while interpreting the radiographic results

More participants in the SOC arm showed definite radiographic pneumonitis (30% versus 19%) and also a higher proportion of failure of resolution (85% versus 63%) at the time of clinical recovery/hospital discharge; one patient with mild radiographic disease in SOC rapidly developed rapidly progressive respiratory distress and required oxygenation and intensive care for recovery. All this may suggest a more serious disease in the SOC arm but this does not seem to have been the case as shown by several other clinical variables, serum biomarkers and overall standard of care drugs used and the treatment response in the current study (Table 1. Table 3, and S5 File). However, it is important to concede that neither a uniform standard protocol nor CT scan imaging was used to evaluate chest skiagrams and/or resolution and thus any interpretation of the current findings of resolution may be premature and requires utmost caution. Radiographic abnormalities may persist beyond clinical recovery and take a longer time for complete resolution [38]. Also, there is insufficient data on prospective evaluation of conventional radiography in COVID-19 [38].

12) In Table 2, * and ** deserved footnotes

Response: Table 2 has been revised, also in view of comments of Reviewer 1.
13) In the subheading of adverse events in Table 5, the authors may change system organ classification to symptoms or diagnosis for clarification.

Response: Earlier we used the WHO organ classification system. The AE are not mentioned as symptoms or diagnosis
14)Discussions- The authors described “In our experience, despite sound medical advice, a large proportion of mild and moderate uncomplicated cases are admitted in the hospital and clog the system. AYUSH 64 plus SOC seemed to have significantly reduced the duration of hospitalization.” Please explain the government policy for admission and isolation of COVID-19 patients.

**Response:** We have revised our statement. Firstly, all COVID-19 cases were admitted in the hospital during the early pandemic as per the recommendations of the Government. This policy was quickly revised as the burden was overwhelming and mild and moderate cases were to monitored in a domiciliary setting or quarantine facility.. However, in the current study, all patients were admitted as per Government recommendations for COVID-19 but seemed to have improved the quality of clinical monitoring and capture of robust data. The results of the current study show that addition of AYUSH hastened clinical recovery and reduced the hospital period. Mild and moderate COVID-19 does not require hospitalization unless there are risk factors and/or likelihood of disease progression or complications. In the study we only treated mild and moderate cases of COVID-19 and the observations of the current study cannot be extrapolated to severe and complicated cases.
15) Discussion-Since AYUSH 64 is popular in India and the study results was disseminated to the public through electronic social and print media, as per the authors’ description, could AYUSH 64 be so popular and could be bought over the counter? Did the authors consider the influence and deviation by SOC group who had taken AYUSH 64?
Did the authors consider that patients may take AYUSH 64 at home without admission in the future? For AYUSH 64 is popularly used to treat acute onset febrile respiratory illnesses for over three decades in India, why did the authors emphasize they need to be medically supervised?

**Response**: The above comment is well taken and accordingly the current revision contains appropriate clarifications and references on the subject. Please refer to the response described in Question serial 6 , 7 and 9 which also addresses a large part of this question. Undoubtedly, Ayurvedic drugs including AYUSH 64 were available across-the-counter without any restrictions. None of the participant patients stated having taken Ayurvedic drug prior to hospital admission but this cannot be vouched for veracity. Patients may have taken drugs from their general practitioner without knowledge of the name or nature (drug) as is often the practise in India. Any effect of AYUSH 64 or Ayurvedic drugs taken prior to admission hopefully will be balanced in the two study treatment arms by randomization. Post discharge, the arm with continued AYUSH 64 did better than the arm without AYUSH 64 in several measures of quality of life and did not differ for long COVID-19 or any other COVID related complication. It is difficult to exclude with certainty the effect of a surreptitious use of Ayurvedic drug or AYUSH 64 by some participants in the post-discharge follow up phase but this would not be of relevance to at least the primary efficacy measure. Compliance during the study is discussed in response to Question number 8.
