# Supplementary material for: Co-administration of AYUSH 64 as an adjunct to standard of care in mild and moderate COVID-19: A randomized, controlled, multicentric clinical trial
Source: PLoS One. 2023 Mar 16;18(3):e0282688. doi: 10.1371/journal.pone.0282688 (PMC10019690; doi:10.1371/journal.pone.0282688)
Supplement: Attachment — Submitted filename: comments of AYUSH 64 in COVID-19.docx [file pone.0282688.s010.docx]

This manuscript described coadministration of AYUSH 64 as an adjunct to Standard of Care in mild and moderate COVID-19 in India: A randomized, controlled, multicentric clinical trial.

The study explored the efficacy of AYUSH 64, a standard polyherbal Ayurvedic drug in mild and moderate COVID-19. The result presents AYUSH 64 in combination with SOC hastened recovery, reduced hospitalization, and improved health in COVID-19. It was considerably safe and well-tolerated.

In general, this is a fair-written manuscript. Other points in this manuscript needed to be clarified are listed below:

Major revisions:

1. In Method, line 145 and 220: open label study is one of selection bias in your study, and your primary endpoint and secondary points are subjective, not scientific or medical specific term. How did you measure RT-PCR of SARS CoV-2? Roche Cobas? What is the definition of negative? Did every center use the same machine? or central lab was provided? If not, you might have information bias in analyzing outcome.
2. Standard of care, line 202: there are several listed medications in supplement file, and dexamethasone was proved effective treatment in COVID-19, except remdesvir. Could you provide the proportion of dexamethasone used in two arms? I think the data would impact the outcome.
3. In Discussion, strength and limitation, line 566: A convenience study sample of 140 participants was felt to be adequate by AC (first author) and SS (Biostatistician co-author)”, and no formal estimation of required sample size for this study was attempted. I think that the authors might write in paragraph of limitations of conclusion, because sampling bias or selection bias is possible. Moreover, the authors sould list some bias in terms of limitations, not to describe the chaos situation when the study was conducted during the pandemic.

Minor revisions:

1. Discussion, line 470: completer analysis should be corrected as per protocol analysis.
2. Reference 50 and 52, line 925 and 935: volume and page are missed.
