# Supplementary material for: Co-administration of AYUSH 64 as an adjunct to standard of care in mild and moderate COVID-19: A randomized, controlled, multicentric clinical trial
Source: PLoS One. 2023 Mar 16;18(3):e0282688. doi: 10.1371/journal.pone.0282688 (PMC10019690; doi:10.1371/journal.pone.0282688)
Supplement: Attachment — Submitted filename: Response to Reviewers_0922.docx [file pone.0282688.s011.docx]

PONE-D-21-24749R1

Coadministration of AYUSH 64 as an adjunct to Standard of Care in mild and moderate COVID-19: A randomized, controlled, multicentric clinical trial

PLOS ONE

**Response to Author/Referee Comments on the First Revision dated 02 Sept 2022.**

***Please Note: The author response is shown below each comment as provided by the journal editor.***

**Comments to the Author *and Response***

I) **General:**

1. If the authors have adequately addressed your comments raised in a previous round of review and you feel that this manuscript is now acceptable for publication, you may indicate that here to bypass the “Comments to the Author” section, enter your conflict of interest statement in the “Confidential to Editor” section, and submit your "Accept" recommendation.

Reviewer #1: (No Response)

Reviewer #3: All comments have been addressed

Author: No comment

2. Is the manuscript technically sound, and do the data support the conclusions?

The manuscript must describe a technically sound piece of scientific research with data that supports the conclusions. Experiments must have been conducted rigorously, with appropriate controls, replication, and sample sizes. The conclusions must be drawn appropriately based on the data presented.

Reviewer #1: (No Response)

Reviewer #3: Yes

Author: No comment

3. Has the statistical analysis been performed appropriately and rigorously?

Reviewer #1: (No Response)

Reviewer #3: Yes

Author: No comment

4. Have the authors made all data underlying the findings in their manuscript fully available?

The [PLOS Data policy](https://nam12.safelinks.protection.outlook.com/?url=http%3A%2F%2Fwww.plosone.org%2Fstatic%2Fpolicies.action%23sharing&data=05%7C01%7C%7C37e51dfafcec41e618eb08da93b38e05%7C84df9e7fe9f640afb435aaaaaaaaaaaa%7C1%7C0%7C637984696656455274%7CUnknown%7CTWFpbGZsb3d8eyJWIjoiMC4wLjAwMDAiLCJQIjoiV2luMzIiLCJBTiI6Ik1haWwiLCJXVCI6Mn0%3D%7C3000%7C%7C%7C&sdata=giA8HGx1lNQibgwTSrIEtp73eCeLChJKEMNa2Omb%2BdA%3D&reserved=0) requires authors to make all data underlying the findings described in their manuscript fully available without restriction, with rare exception (please refer to the Data Availability Statement in the manuscript PDF file). The data should be provided as part of the manuscript or its supporting information, or deposited to a public repository. For example, in addition to summary statistics, the data points behind means, medians and variance measures should be available. If there are restrictions on publicly sharing data—e.g. participant privacy or use of data from a third party—those must be specified.

Reviewer #1: (No Response)

Reviewer #3: Yes

Author : Further data has been added to address the comment on the efficacy analysis (below)

5. Is the manuscript presented in an intelligible fashion and written in standard English?

PLOS ONE does not copyedit accepted manuscripts, so the language in submitted articles must be clear, correct, and unambiguous. Any typographical or grammatical errors should be corrected at revision, so please note any specific errors here.

Reviewer #1: (No Response)

Reviewer #3: No

Author: The revision was suitably edited by professional in English language. However, we note (see below) that there are still ‘minor issues with English language usage throughout the manuscript’ according to Reviewer 1. The current revision has been edited by a new professional in English language.

6. Review Comments to the Author

Please use the space provided to explain your answers to the questions above. You may also include additional comments for the author, including concerns about dual publication, research ethics, or publication ethics. (Please upload your review as an attachment if it exceeds 20,000 characters)

Author: we are grateful to the author and the referees for their comments to improve the manuscript

II) **Reviewer #1**: In general, most issues have been dealt with in the response. The manuscript is improved.

The following are less important issues:

1)There still are many minor issues with English language usage throughout the manuscript. These are not critical but do detract from the presentation.

Author: This revision has been checked by a new professional in English language use.

2)Lines 163-164 seem quite odd. Perhaps these are not correctly expressing the authors' desired meaning?

Author: Deleted

3)The statistical methods section is somewhat improved with regard to content. However, the English language usage requires a lot of rewriting.

Author: This has been again checked and revised by the study Biostatistician. We followed the a-priori statistical plan as per protocol. However, some additional analysis was performed in response to the excellent suggestion of the referee.
4) It appears that the authors ran both a fixed effects model (with treatment and site as fixed effects) and a linear mixed effects model (with treatment as a fixed effect and site as a random effect). Is this correct? If so, why? The mixed effects model is arguably better if the authors wish to generalize to "all sites" --- assuming these sites can be thought of as a representative sample if "all sites". The fixed effects model is appropriate if the authors wish to restrict their inference to "only the sites studied".

Author: (a) The data on the primary efficacy measure ‘time from randomization to clinical recovery’ in the study between the two study groups was analysed using Students’ t test and MW statistic and the results are shown in Table 2.

(b) We further performed a linear mixed effect model (treatment as fixed effect and site as random) as recommended by the referee the results were consistent with our earlier primary efficacy analysis. This is mentioned in the text and the detail model output is shown in Supplementary 5 File (Table S5.4).

5)For tabulation, please footnote "not significant" results with "NS" and not with "*". Use the following scheme:

* Statistically significant (p < 0.05)
NS Not statistically significant (p >= 0.05)
a Other footnote that does not relate to statistical significance
b Other footnote that does not relate to statistical significance
etc.
Author: We have used this scheme for the Tables and we thank the referee
6) Table 2 requires some reformatting for readability, in particular the rows with the p-values.

Author: This has been done.

7)I was not able to 100% reproduce the values in the table with the raw data --- perhaps there are some data exclusions that are relevant? However, the results are fairly close.

Author: Please note that the raw data of 136 subjects in the earlier supplement pertained to per protocol analysis. Now we have provided the data of 140 subjects out of which 137 qualify for ITT analysis (3 withdrawn prematurely and did not provide data on clinical recovery).
We have reanalysed both per protocol and ITT analysis using the raw data provided in the supplement. Overall there are no variations from the earlier results..

8) The last column of Table 2 is incorrect. This should show the result of a model that incorporates site as a random variable. A simple t-test is incorrect for the entire data set.

Author: As per protocol, we first carried out a standard head-to-head comparison of the two study intervention groups using Students T test and MW statistic and wish to retain this as the primary efficacy measure analysis . However, we carried out a liner mixed effect model to exclude the effect of study site on primary outcome as suggested by the referee and include the result in the text.
9)Please see the following code which reproduces part of Table 2 as well as demonstrating how to perform the linear mixed effects analysis using the R programming language.

\# Load packages.

library(tidyverse)
library(broom)
library(lme4)
library(car)

\# Read data and set data conventions.

S8 <- read_csv("S8_Raw.csv")

names(S8) <- c("PatientID", "Group", "Age", "Gender", "Site", "RtoCR", "SOtoCR")

S8 <- S8 %>%
mutate(Site = str_c("Site ", Site))

#-------------------------------------------------------------------------------
\# Perform brief evaluation of time from randomization to complete recovery.

\# Plot the data.

S8 %>%
ggplot(aes(x = Group, y = RtoCR)) +
geom_boxplot() +
facet_grid( ~ Site)

S8 %>%
ggplot(aes(x = Group, y = RtoCR)) +
geom_boxplot() +
facet_grid( ~ Site) +
scale_y_log10()

S8 %>% ggplot(aes(x = Age, y = RtoCR, col = Group)) +
geom_point() +
facet_grid(~ Site)

S8 %>% ggplot(aes(x = Gender, y = RtoCR, col = Group)) +
geom_boxplot() +
facet_grid(~ Site)

S8 %>% ggplot(aes(x = SOtoCR, y = RtoCR, col = Group)) +
geom_point() +
facet_grid(~ Site)

\# Perform t-tests within each site.

Stats <- S8 %>%
group_by(Site) %>%
nest() %>%
mutate(
fit = map(data, ~t.test(.x$RtoCR ~ .x$Group)),
tidy = map(fit, tidy)
) %>%
unnest(tidy)

Stats

\# Perform linear mixed effect analysis with site as a random effect.

fit <- lmer(RtoCR ~ Group + (1 | Site), data = S8)
#fit <- lmer(log(RtoCR) ~ Group + (1 | Site), data = S8)

plot(fit)

Anova(fit, test = "F")

\# NOTE Arguably slightly better fit on logarithmic scale with no change in
\# conclusion. It is easiest to interpret on the original scale.

#-------------------------------------------------------------------------------
\# Perform brief evaluation of time from symptom onset to complete recovery.

\# Plot the data.

S8 %>%
ggplot(aes(x = Group, y = SOtoCR)) +
geom_boxplot() +
facet_grid( ~ Site)

S8 %>%
ggplot(aes(x = Group, y = SOtoCR)) +
geom_boxplot() +
facet_grid( ~ Site) +
scale_y_log10()

S8 %>% ggplot(aes(x = Age, y = SOtoCR, col = Group)) +
geom_point() +
facet_grid(~ Site)

S8 %>% ggplot(aes(x = Gender, y = SOtoCR, col = Group)) +
geom_boxplot() +
facet_grid(~ Site)

\# Perform t-tests within each site.

Stats <- S8 %>%
group_by(Site) %>%
nest() %>%
mutate(
fit = map(data, ~t.test(.x$SOtoCR ~ .x$Group)),
tidy = map(fit, tidy)
) %>%
unnest(tidy)

Stats

\# Perform linear mixed effect analysis with site as a random effect.

fit <- lmer(SOtoCR ~ Group + (1 | Site), data = S8)
fit <- lmer(log(SOtoCR) ~ Group + (1 | Site), data = S8)

plot(fit)
Anova(fit, test = "F")

\# NOTE Better fit on logarithmic scale but again with no change in overall
\# conclusion. It is easiest to interpret on the original scale.

Author: We thank the Referee for his very considerate suggestion and providing the code for R programming. We have no experience with R. We have used standard statistical programs mentioned in the text and hope that the same will be accepted.

III) Reviewer #3: This manuscript described coadministration of AYUSH 64 as an adjunct to Standard of Care in mild and moderate COVID-19 in India: A randomized, controlled, multicentric clinical trial.

The study explored the efficacy of AYUSH 64, a standard polyherbal Ayurvedic drug in mild and moderate COVID-19. The result presents AYUSH 64 in combination with SOC hastened recovery, reduced hospitalization, and improved health in COVID-19. It was considerably safe and well-tolerated. In general, this is a fair-written manuscript. Other points in this manuscript needed to be clarified are listed below:

Author: We thank the referee.
Major revisions:
1). (a) In Method, line 145 and 220: open label study is one of selection bias in your study, and (b) your primary endpoint and secondary points are subjective, not scientific or medical specific term.(c) How did you measure RT-PCR of SARS CoV-2? Roche Cobas? What is the definition of negative? Did every center use the same machine? or central lab was provided? If not, you might have information bias in analyzing outcome.

Author:

(a) Agreed and we have included the possibility of a selection bias due to the open label nature of the study and convenience sample in the study limitation. We have further explained the good match for several variables at randomization baseline. Also, the inpatients were directly observed for drug administration, monitoring of symptoms and oximetry, clinical recovery and adverse events by a dedicated study team. The primary efficacy outcome was assessed blindly by a dedicated independent COVID physician.

(b) We agree that the primary and secondary end points are dependent upon the clinical response elicited from the patient and prone to inaccuracy and bias. But in our case, there was no single criteria but a set of criteria to determine ‘clinical recovery’ (CR) within a time frame of 28 days. CR was a combination of resolution of symptoms and normal peripheral oximetry for 48 hours and a negative RT-PCR assay. We wish to add that the concept of CR in clinical drug trials for COVID-19 was still evolving when we designed this study in May-June 2020. Interestingly, there continues to be paucity of data on the clinical and microbial efficacy endpoints and what constitutes CR in controlled drug trial studies of mild to moderate COVID-19 in published literature. Most of the studies have used ‘viral load’ as the primary efficacy as a surrogate marker of clinical improvement. The WHO index of severity of 7 symptoms to indicate improvement at some fixed time points between 7-14 days after intervention were often used as secondary efficacy measure. There is no universally accepted definition of clinical recovery in mild to moderate COVID-19. Some studies have used clinical end points of hospitalization, morbidity or mortality. In this regard, our primary efficacy measure was unique. It was more wholesome, pragmatic and reasonable and based on our early clinical experience with mild moderate cases of COVID 19. It should be noted that we applied the criteria in hospitalized patients where the clinical monitoring was daily, direct and in real time. There may be more subjectivity in a domiciliary setting or when clinical data was obtained telephonically or through digital platform. It is prudent to add, that only one patient in the study progressed to severe stage. Also, during the follow up of 12 weeks after clinical recovery, there were no clinical concerns of any important COVID sequel. However, encouraged by the referee comment, we have included a brief note on this pivotal issue in discussion section.

(c) None of the labs at study sites used Roche Cobas and we did not use a central lab.

This was a Government of India sponsored study carried out at Government approved medical facilities. It was mandatory for the labs to follow the instructions issues by Indian Council of Medical Research (ICMR) for the specific SARS-CoV-2 RT-PCR assay

[Ref: (i) <https://www.icmr.gov.in/ctestlab.html>

(ii) [Advisory_for_Reagents_TestingLabs_v1.pdf (icmr.gov.in)](https://www.icmr.gov.in/pdf/covid/labs/Advisory_for_Reagents_TestingLabs_v1.pdf)

(iii) [Microsoft Word - SARS_CoV2_using_TaqPath_COVID19_ComboKit (icmr.gov.in)](https://www.icmr.gov.in/pdf/covid/labs/SARS_CoV2_using_TaqPath_COVID19_ComboKit.pdf) ].

All kits, reagents and equipment was to conform to the recommendations of ICMR. Since then, we have confirmed that the labs at each of the three study sites followed ICMR guidelines of quality control and standard testing but procured reagents and equipment from different manufacturers. Each of the site lab followed the manufacturer guidelines for reporting the results of the RT-PCR assay. A negative RT-PCR was reported usually after a run of 28-30 cycles.

However, we admit that some information bias is likely in reporting outcome based on RT-PCR assay in the current study and this is now included in the manuscript.

2). Standard of care, line 202: there are several listed medications in supplement file, and dexamethasone was proved effective treatment in COVID-19, except remdesvir. Could you provide the proportion of dexamethasone used in two arms? I think the data would impact the outcome.

Author: We have listed the medications in the supplement file that were accepted as standard of care according to the guidelines for treatment issued by Indian Council of Medical Research and Ministry of Health, Government of India during the pandemic. This is referenced in the paper. However, the drugs were to be used as per severity of the illness and clinical discretion. There were differences in the use of drugs at study sites. Dexamethasone was only used in one subject who developed an acute onset of breathlessness and drop in arterial oxygen; withdrawn from the study.

3. In Discussion, strength and limitation, line 566: A convenience study sample of 140 participants was felt to be adequate by AC (first author) and SS (Biostatistician co-author)”, and no formal estimation of required sample size for this study was attempted. I think that the authors might write in paragraph of limitations of conclusion, because sampling bias or selection bias is possible. Moreover, the authors should list some bias in terms of limitations, not to describe the chaos situation when the study was conducted during the pandemic.

Author: We agree with the comment and accordingly modified the section on limitations and conclusion. We have mentioned the possibility of a selection bias as described in the comment above also/

Minor revisions:
1). Discussion, line 470: completer analysis should be corrected as per protocol analysis.

Author: We have corrected the term
2. Reference 50 and 52, line 925 and 935: volume and page are missed.

Author: The references have been corrected as per the citation provided by the published paper and conforming to PLOS One.
