# Supplementary material for: Co-administration of AYUSH 64 as an adjunct to standard of care in mild and moderate COVID-19: A randomized, controlled, multicentric clinical trial
Source: PLoS One. 2023 Mar 16;18(3):e0282688. doi: 10.1371/journal.pone.0282688 (PMC10019690; doi:10.1371/journal.pone.0282688)
Supplement: Attachment — Submitted filename: PLOS One_Author reponse 201022.docx [file pone.0282688.s012.docx]

PONE-D-21-24749R1

Coadministration of AYUSH 64 as an adjunct to Standard of Care in mild and moderate COVID-19: A randomized, controlled, multicentric clinical trial

PLOS ONE

**Response to Author/Referee Comments on the Second Revision dated 10 Oct 2022.**

***Please Note: The author response is shown below each comment as provided by the journal editor.***

Additional Editor Comments (if provided):

The whole manuscript still contained many grammar errors and missing notes. For examples:

Response: My apologies for these errors. The manuscript has been rechecked by a profession in use of English language and medical writing.
Editor:
1. At line 39, “Significance p <0.05, two sided.” This is not a complete sentence

Response: Corrected
2. In the abstract, “App.”, “CI”, “PP”, abbreviations were presented without preceding full names.

Response: All abbreviations are rechecked and full name provided
3. At line 61, “ the World” should the “the world”

Response: corrected
4. At line 97, HCQS without full names

Response: corrected
5. At line 163, the meanings of “The patients did not assess the burden or the outcome of the intervention.” was not clear

Response: This is deleted and the sections rewritten.
6. At line 171, “ real life PCR” must be “real time”

Response: corrected
7. At line 293, CRO were presented without preceding full name.

Response: A full form was provided in the earlier text
8. For figures and tables:
Table 1 Footnote: (p,0.05) may be p<0.05, like other footnotes in table 2-5.
Table 2 # was not explained in the footnote.

Table 3: * did not explain the comparison groups or arms. Is it significant?
Table 4: ** was not explained in the footnote.
Table 5: In Column 1, is “Investigation” represented anything?
Figure 2: “completer” was suggested change to “per protocol”
Response: All Tables are rechecked and corrections made. Figure 2 is revised
9. At line 417-418, “there was a significant reduction in serum biomarkers of COVID-19 in each of the study groups without any significant difference (Table 3).” This sentence was not clear.

Response: Though the difference from baseline was significant in each of the two groups, there was no significant difference between the groups. This is now further clarified

10. At line 430-432, “ In comparison to SOC, AYUSH Plus showed significant improvement in several domains (physical health, psychological health, social relationship, and environmental well-being) in the WHO QOL BREF and the total HR-BHF score the time of clinical recovery and pre-determined follow-up time points (Table 4).” May the authors check again the completeness of the sentences?

Response: The sentence is further made clear. It now reads ‘In comparison to SOC, AYUSH Plus showed significant improvement in several domains (physical health, psychological health, social relationship, and environmental well-being) in the WHO QOL BREF and the total HR-BHF score at the time of clinical recovery and during follow-up (Table 4).’
